# The global burden, trends and cross-region inequities of non-communicable diseases attributed to ambient particulate matter pollution

**DOI:** 10.3389/fpubh.2025.1682574

**Published:** 2025-11-03

**Authors:** Songsong Wang, Hanpeng Lai

**Affiliations:** ^1^Department of Public Health Monitoring and Evaluation, Yantai Center for Disease Control and Prevention, Yantai, China; ^2^School of Public Health, Yangzhou University, Yangzhou, China

**Keywords:** Global Burden of Disease, ambient particulate matter pollution, non-communicable disease, cross-region inequities, age-standardized rate

## Abstract

**Background:**

Ambient particulate matter poses long-term and geographically uneven health risks, warranting assessment of global burden and regional disparities.

**Methods:**

GBD 2019 data was used to assess age standardized rates of mortality (ASMR) and DALY (ASDR) from five chronic diseases due to ambient particulate matter from 1990 to 2019, focusing on temporal trends and cross-regional disparities using the Socio-demographic Index (SDI).

**Results:**

In 2019, ischemic heart disease (IHD) and stroke accounted for the greatest burdens attributable to ambient particulate matter, with global ASMRs of 30 and 26 per 100,000, respectively, followed by chronic obstructive pulmonary disease (COPD) at 16, tracheal bronchus and lung (TBL) cancer at 6.8, and diabetes mellitus at 4.5. From 1990 to 2019, COPD showed a marked decline with an AAPC on ASMR of −0.47, while diabetes and TBL cancer rose sharply, with AAPCs of 1.57 and 0.75, respectively. ASDR for the five disease had similar patterns. Southeast Asia, East Asia, and Oceania carried the heaviest TBL cancer and stroke burden from 2000 on, North Africa and the Middle East ranked consistently highest for IHD and diabetes mellitus, and South Asia emerged as the global hotspot for COPD after 2005. TBL cancer was concentrated in higher-SDI regions, whereas COPD and diabetes mellitus rose disproportionately in lower-SDI areas. Population growth and aging were the primary drivers of increases across all diseases. Health inequality analysis further showed a general shift of burdens from high- to low-SDI countries, with indices declining from high positive values to lower or even negative values between 1990 and 2019.

**Conclusion:**

Ambient particulate matter continues to drive unequal disease burdens, especially in low- and middle-SDI regions, and more targeted efforts are thus needed.

## 1 Introduction

Air pollution, a major environmental threat to human health, has been responsible for approximately seven million deaths annually worldwide, according to the 2021 World Health Organization (WHO) Global Air Quality Guidelines, with adverse effects from both long- and short-term exposure (1). In the realm of environmental air pollutants, PM2.5, which has an aerodynamic diameter of ≤ 2.5 microns, warrants special attention because an estimated 92% of the global population lives in areas where PM2.5 levels exceed 10 μg/m^3^, recommended annual average concentration established by the WHO based on the air quality guideline in 2005 (2). However, mounting evidence suggests that potential health impacts related to PM2.5 have greatly surpassed earlier scientific understanding. Despite recommended value at 5 μg/m^3^ by the WHO in 2021, new causal modeling has shown a higher risk of all-cause mortality exposed to lower levels of PM2.5 (3). To date, researchers have confirmed that health conditions from PM2.5 exposure include not only respiratory but also cardio-cerebral vascular, nervous, and endocrine diseases (4).

International efforts to reduce PM2.5 pollution have been ongoing but less than ideal. The Global Burden of Disease Study 2019 (GBD 2019) revealed that over the past 10 years, although the summary exposure values of indoor air pollution from solid fuels decreased by a yearly mean of 3.70%, exposure to ambient particulate matter increased by 1.46% annually (5). Moreover, ambient particulate matter pollution intensified its impact on the middle-aged and older population in 2019, ranking as the fifth and sixth contributors to disability-adjusted life years (DALYs) for the age groups 50–74 and 75+, respectively, which was higher than overall seventh position across all ages (5). The regional disparities in the distribution of ambient particulate pollution are undeniable, with the main contributors to global growth since 2011 being South Asia, the Middle East, and Africa, primarily comprised of low- and middle-income countries (LMICs), despite a slowdown in upward trends (6). Given the delayed health effects of particulate matter and the irreversible trend of population aging, there is a need for continuous and enhanced academic focus on its potential adverse outcomes, particularly concerning the associated burden of chronic non-communicable diseases.

Previous literature has thoroughly analyzed the effects of ambient particulate pollution on the total disease burdens globally; however, integrative analyses that simultaneously contrast the attributable burden of multiple chronic non-communicable diseases while accounting for geographic variation remain critically underexplored (7–11). In response, this research systematically compared five representative chronic diseases attributable to particulate matter across regions using GBD 2019 data, aiming to deepen scientific insights and inform evidence-based interventions on the long-term health impacts of environmental exposures.

## 2 Materials and methods

### 2.1 Data sources

The data were selected from GBD 2019,^1^ where disease burden estimates originated from various sources, including population surveys, health registries, and risk surveillance (12). Considering the public accessibility of GBD data and the absence of identifiable information, there was no requirement for ethical approval or informed consent (Detailed strategies for data search were provided in Text S1). The Socio-demographic Index (SDI) was created to represent health development status using three key indicators: total fertility rate for those under 25, average education level for those 15 and older, and per capita income adjusted for distribution lags (13). SDI data were obtained directly from the GBD 2019 estimates provided by the Institute for Health Metrics and Evaluation (IHME) (SDI data for 204 countries and territories were provided in Text S2). Based on the GBD 2019 framework, 204 countries and territories were categorized into five groups of SDI, namely, low (0–0.4658), low-middle (0.4658–0.6188), middle (0.6188–0.7120), high-middle (0.7120–0.8103), and high (0.8103–1.0).

Five chronic non-communicable diseases attributable to ambient particulate matter pollution, i.e., tracheal bronchus and lung (TBL) cancer, ischemic heart disease (IHD), stroke, chronic obstructive pulmonary disease (COPD), diabetes mellitus, all defined by the International Classification of Diseases, 10th Edition (ICD-10) (5) (Text S3 gave accurate codes of the five diseases).

### 2.2 Statistical analysis

In this study, which targeted individuals aged 25 years and older, extending up to 95 + years, rather than the general population, age-standardized mortality rates (ASMRs) and age-standardized DALY rates (ASDRs) were recalculated using the GBD world standard population as a reference. The original standard includes all ages; however, a new standard population was created by extracting ages 25–95+, and the direct age-standardization method was applied (14). Uncertainty intervals (UIs) of 95% for all estimates were calculated through 1,000 iterations of each computation step and derived from the 25th and 975th percentiles of the ordered data. To estimate temporal trends of age-standardized rates over a specific interval, average annual percentage changes (AAPCs) were determined using joinpoint regressions (15). Optimal points, identified by changes in slope, were connected through logarithmic linear models, allowing for a maximum of five joint points (16). The joinpoint regression formula is as follows:


$$E(y|x)=e^{\beta_{0}+\beta_{1}x+\delta_{1}(x-\tau_{1})+\ldots +\delta_{k}(x-\tau_{k})+\ldots }$$

where *k* denoted turning points, *τ_k_* denoted unknown turning points, *β*_0_ denoted constant, *β*_1_ denoted regression coefficient, and *δ_k_* denoted regression coefficient of the *k*th piecewise function. Changes in disease burden were decomposed to quantify the contributions of population growth, population aging, and epidemiological change, with population growth reflecting increasing population size, aging reflecting shifts in the population age structure, and epidemiological change reflecting variations in disease-specific rates independent of population size and age (17). The slope index of inequality was calculated by regressing national rates across all age groups on an SDI-related relative position scale, defined by the midpoint of the cumulative range of the population ranked by SDI. The concentration index was calculated by integrating the area beneath the fitted Lorenz concentration curve, using the cumulative fraction of burden and the cumulative relative distribution of the population ranked by SDI. All analyses were conducted using R version 4.3.3 software (Institute for Statistics and Mathematics, Austria). A two-sided *p* < 0.05 was considered statistically significant.

## 3 Results

### 3.1 Trends of TBL cancer due to ambient particulate matter

**Figure 1:**
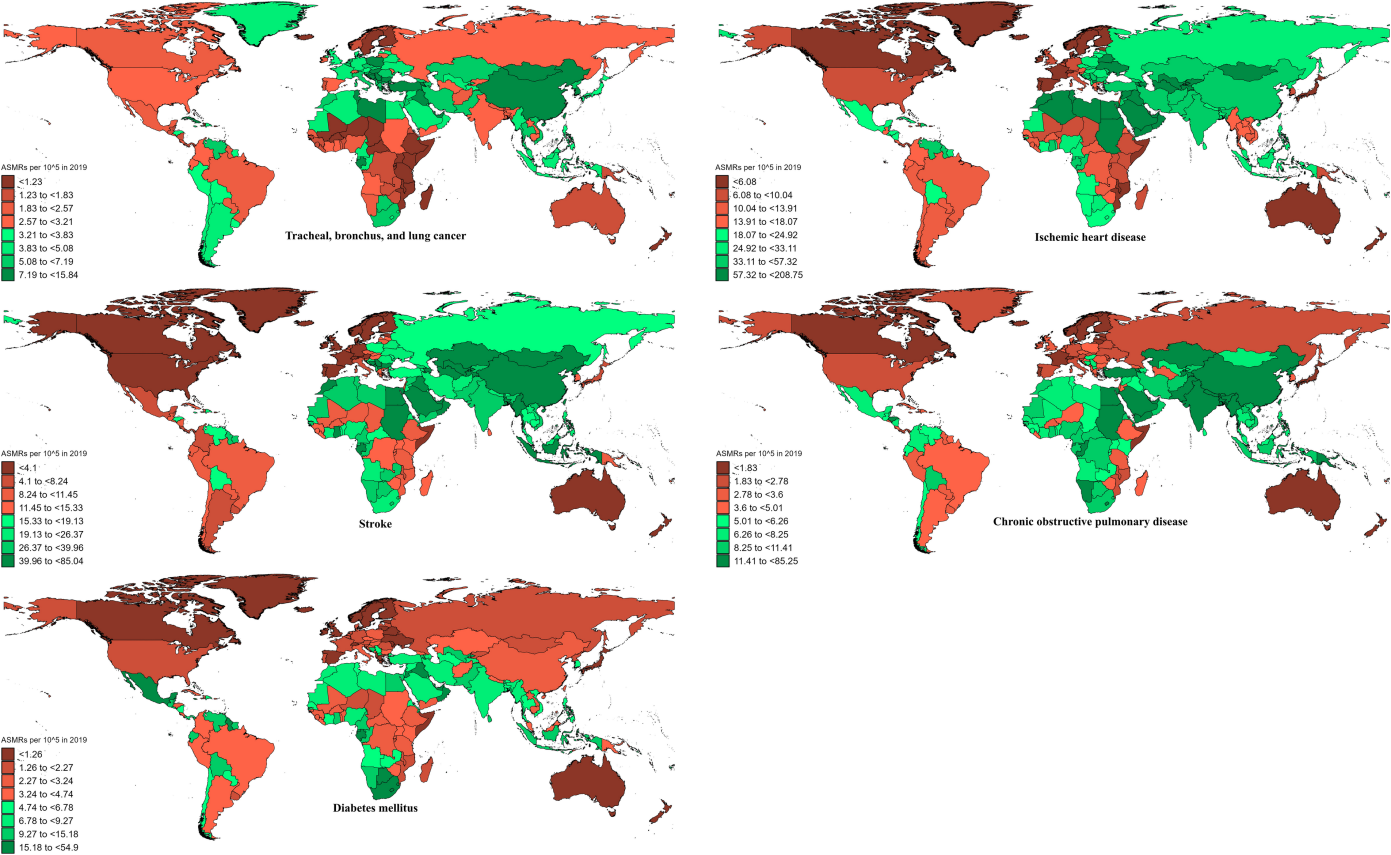
ASMRs of ambient particulate matter-attributed non-communicable diseases in 2019.

**Figure 2:**
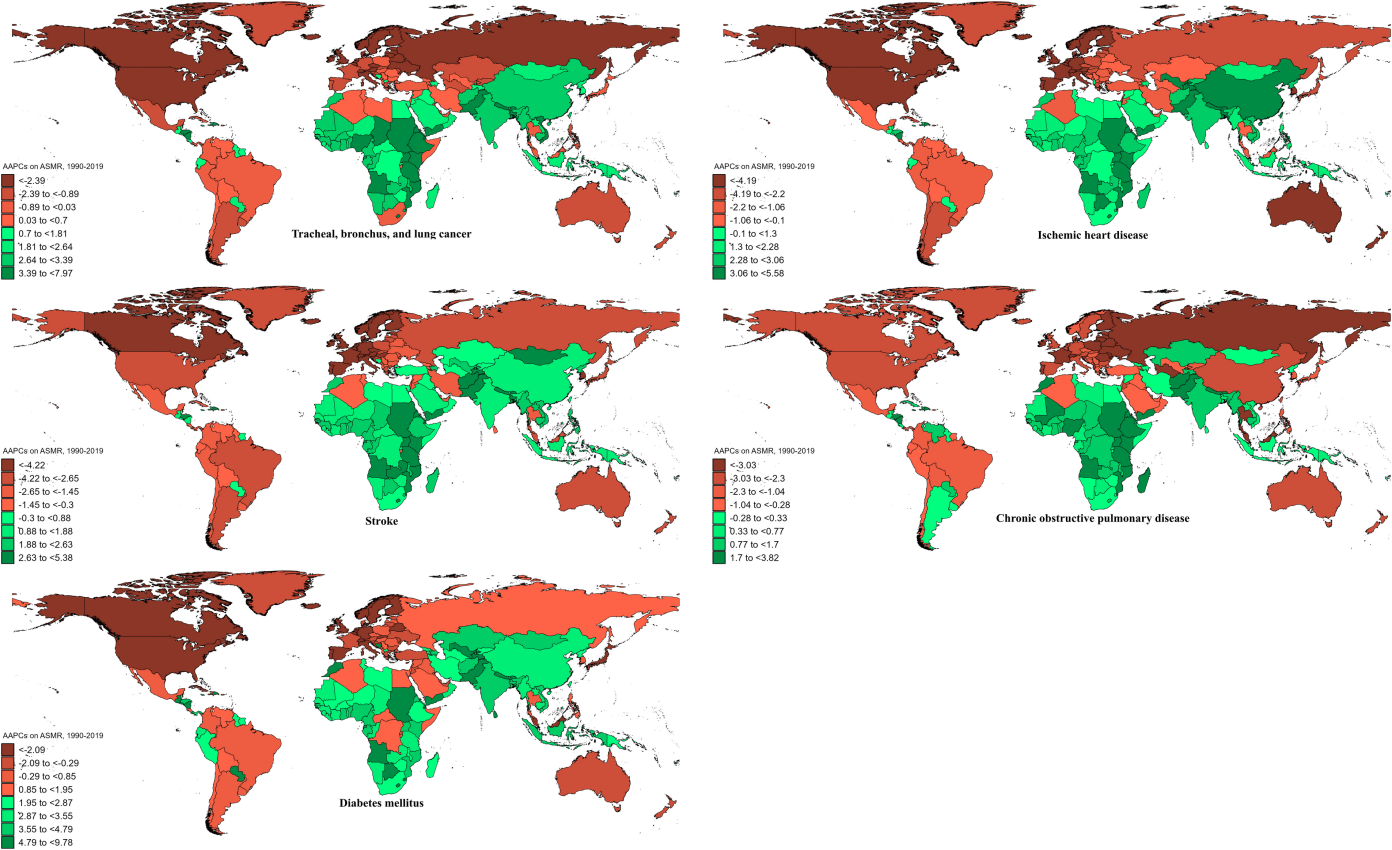
AAPCs on ASMRs of ambient particulate matter-attributed non-communicable diseases from 1990 to 2019.

**Figure 3:**
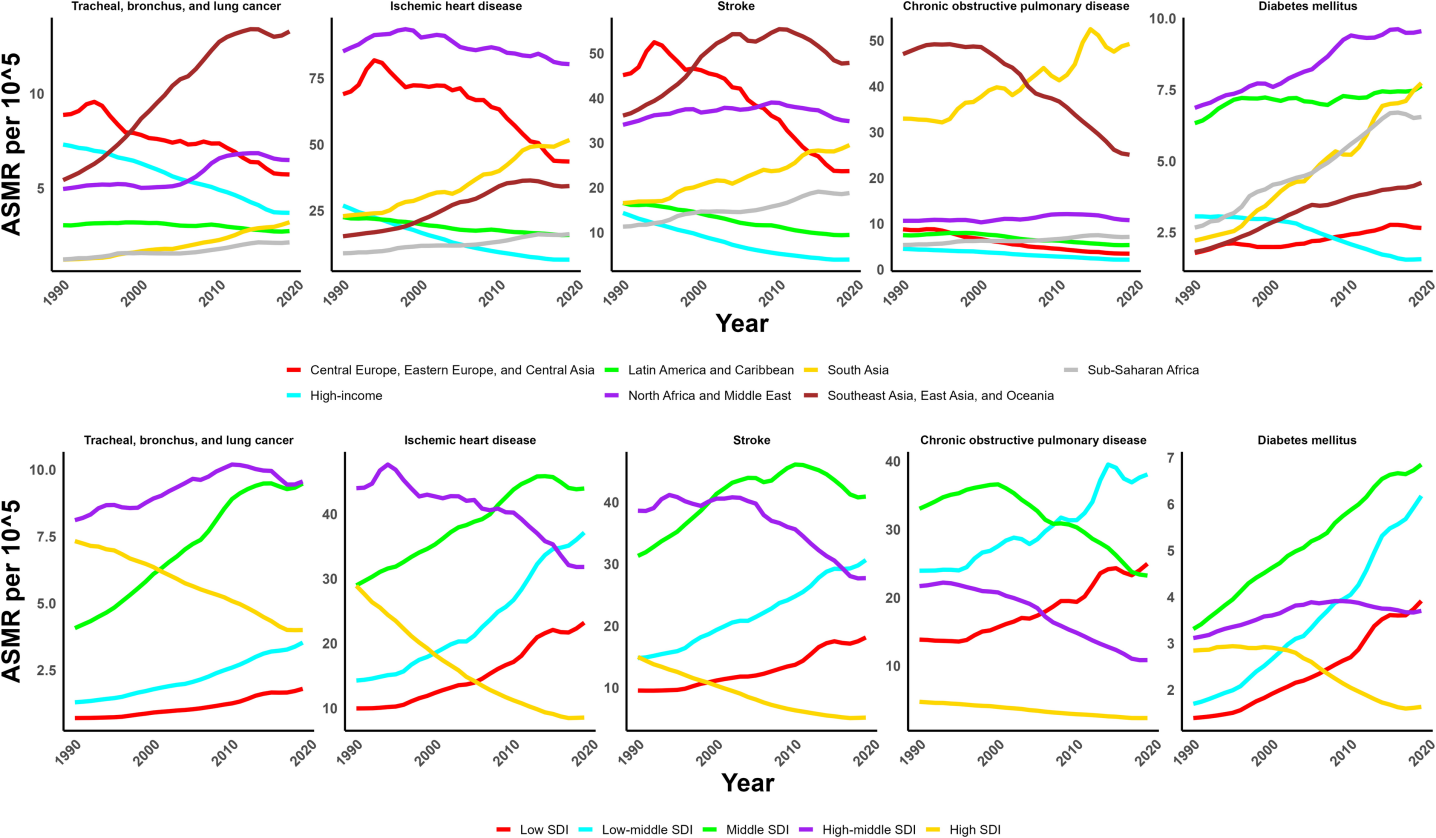
Trends of ASMR of non-communicable diseases attributed to ambient particulate matter from 1990 to 2019.

**Figure 4:**
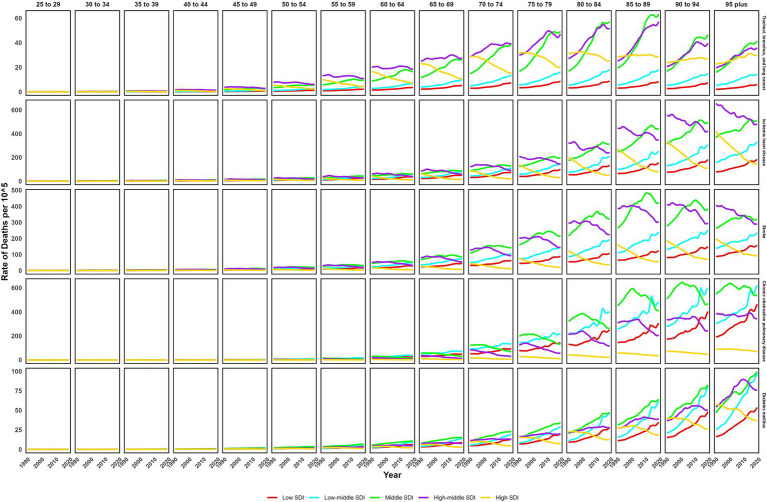
Trends of age-specific death rate of non-communicable diseases attributed to ambient particulate matter from 1990 to 2019.

In 2019, the global ASMR and ASDR for TBL cancer attributed to ambient particulate matter were 6.8 and 150 per 10^5^, with China and Serbia recording the highest values, respectively (Table 1; Supplementary Tables S1–S3; Figure 1; Supplementary Figure S1). From 1990 to 2019, the overall ASMR and ASDR significantly rose with AAPCs of 0.75 and 0.48, respectively, the highest occurred in Equatorial Guinea and the lowest in Finland (Table 1; Supplementary Tables S1–S3; Figure 2; Supplementary Figure S2). Southeast Asia, East Asia, and Oceania saw significant increases in burden, mainly before 2010, while South Asia and Sub-Saharan Africa showed steady upward trends from extremely low levels (Figure 3; Supplementary Figure S3). High-middle SDI areas have shown the highest ASMR and ASDR over the past 30 years, with similar patterns observed among individuals aged 45–74 (Figures 3, 4; Supplementary Figures S3, S4).

**Table 1:** The deaths of non-communicable diseases attributed to ambient particulate matter pollution in 2019, and their temporal trends from 1990 to 2019.

Location	TBL cancer	IHD	Stroke	COPD	Diabetes mellitus
Global					
Deaths in 2019 (million)	0.308 (0.225, 0.396)	1.332 (0.995, 1.698)	1.143 (0.871, 1.417)	0.695 (0.544, 0.870)	0.197 (0.136, 0.259)
ASMR in 2019 (per 100,000)	6.835 (4.998, 8.794)	30.024 (22.384, 38.336)	25.794 (19.626, 32.000)	16.198 (12.653, 20.275)	4.460 (3.085, 5.880)
AAPC on ASMR, 1990–2019	0.747 (0.564, 0.929)	−0.031 (−0.245, 0.184)*	0.095 (−0.196, 0.387)*	−0.473 (−0.622, −0.324)	1.569 (1.421, 1.717)
Male					
Deaths in 2019 (million)	0.216 (0.281, 0.156)	0.807 (1.029, 0.604)	0.654 (0.813, 0.495)	0.412 (0.516, 0.318)	0.099 (0.132, 0.069)
ASMR in 2019 (per 100,000)	10.458 (7.528, 13.592)	39.407 (29.389, 50.353)	32.264 (24.434, 40.160)	22.132 (17.096, 27.771)	5.000 (3.473, 6.632)
AAPC on ASMR, 1990–2019	0.367 (0.163, 0.571)	0.041 (−0.175, 0.258)*	0.346 (0.080, 0.613)	−0.607 (−0.768, −0.445)	1.870 (1.683, 2.057)
Female					
Deaths in 2019 (million)	0.091 (0.119, 0.065)	0.525 (0.682, 0.383)	0.489 (0.625, 0.363)	0.283 (0.373, 0.205)	0.097 (0.129, 0.067)
ASMR in 2019 (per 100,000)	3.771 (2.697, 4.922)	21.682 (15.839, 28.173)	20.204 (14.993, 25.794)	11.661 (8.427, 15.340)	4.018 (2.754, 5.323)
AAPC on ASMR, 1990–2019	1.638 (1.493, 1.783)	−0.173 (−0.384, 0.038)*	−0.227 (−0.494, 0.041)*	−0.404 (−0.611, −0.198)	1.301 (1.160, 1.442)
Low SDI					
Deaths in 2019 (million)	0.005 (0.003, 0.008)	0.062 (0.037, 0.093)	0.047 (0.027, 0.071)	0.053 (0.036, 0.073)	0.009 (0.005, 0.015)
ASMR in 2019 (per 100,000)	1.804 (1.033, 2.780)	23.204 (13.667, 34.549)	18.117 (10.554, 27.488)	24.970 (17.095, 34.153)	3.913 (2.035, 6.283)
AAPC on ASMR, 1990–2019	3.270 (2.859, 3.683)	2.981 (2.520, 3.443)	2.237 (1.716, 2.760)	2.071 (1.551, 2.593)	3.637 (3.433, 3.842)
Low-middle SDI					
Deaths in 2019 (million)	0.026 (0.017, 0.035)	0.269 (0.184, 0.358)	0.216 (0.147, 0.287)	0.238 (0.173, 0.307)	0.041 (0.026, 0.058)
ASMR in 2019 (per 100,000)	3.531 (2.311, 4.773)	37.132 (25.370, 49.462)	30.629 (20.859, 40.674)	38.073 (27.590, 49.019)	6.176 (3.928, 8.682)
AAPC on ASMR, 1990–2019	3.515 (3.309, 3.721)	3.335 (2.890, 3.782)	2.519 (2.072, 2.968)	1.737 (1.072, 2.406)	4.565 (4.056, 5.077)
Middle SDI					
Deaths in 2019 (million)	0.125 (0.090, 0.164)	0.558 (0.427, 0.697)	0.516 (0.395, 0.639)	0.257 (0.201, 0.326)	0.086 (0.061, 0.112)
ASMR in 2019 (per 100,000)	9.478 (6.779, 12.367)	43.941 (33.478, 55.064)	40.891 (31.184, 50.797)	23.234 (18.076, 29.471)	6.852 (4.831, 8.916)
AAPC on ASMR, 1990–2019	2.925 (2.581, 3.271)	1.437 (1.247, 1.628)	0.962 (0.722, 1.202)	−1.256 (−1.454, −1.058)	2.535 (2.432, 2.639)
High-middle SDI					
Deaths in 2019 (million)	0.109 (0.080, 0.140)	0.352 (0.253, 0.464)	0.308 (0.235, 0.387)	0.118 (0.088, 0.158)	0.041 (0.029, 0.054)
ASMR in 2019 (per 100,000)	9.557 (7.005, 12.300)	31.817 (22.861, 42.039)	27.705 (21.087, 34.838)	10.840 (8.060, 14.531)	3.701 (2.599, 4.849)
AAPC on ASMR, 1990–2019	0.574 (0.242, 0.907)	−1.078 (−1.433, −0.723)	−1.112 (−1.561, −0.660)	−2.382 (−2.614, −2.149)	0.602 (0.494, 0.710)
High SDI					
Deaths in 2019 (million)	0.043 (0.028, 0.062)	0.090 (0.055, 0.137)	0.056 (0.038, 0.079)	0.028 (0.017, 0.042)	0.018 (0.011, 0.028)
ASMR in 2019 (per 100,000)	4.001 (2.590, 5.776)	8.535 (5.307, 12.853)	5.167 (3.541, 7.275)	2.340 (1.422, 3.502)	1.632 (1.001, 2.427)
AAPC on ASMR, 1990–2019	−2.063 (−2.179, −1.946)	−4.108 (−4.257, −3.959)	−3.592 (−3.757, −3.426)	−2.411 (−2.633, −2.188)	−1.888 (−2.148, −1.627)
Central Europe, Eastern Europe, and Central Asia					
Deaths in 2019 (million)	0.020 (0.014, 0.027)	0.150 (0.096, 0.216)	0.082 (0.057, 0.114)	0.012 (0.008, 0.017)	0.009 (0.006, 0.013)
ASMR in 2019 (per 100,000)	5.716 (3.979, 7.662)	43.535 (27.691, 62.740)	23.698 (16.311, 32.839)	3.451 (2.290, 4.825)	2.652 (1.780, 3.621)
AAPC on ASMR, 1990–2019	−1.471 (−1.872, −1.068)	−1.509 (−2.041, −0.975)	−2.125 (−2.613, −1.635)	−3.133 (−3.429, −2.835)	1.419 (1.212, 1.626)
High-income					
Deaths in 2019 (million)	0.044 (0.028, 0.065)	0.081 (0.045, 0.130)	0.051 (0.032, 0.077)	0.030 (0.017, 0.045)	0.020 (0.012, 0.031)
ASMR in 2019 (per 100,000)	3.689 (2.303, 5.428)	6.474 (3.598, 10.454)	3.952 (2.496, 5.957)	2.150 (1.261, 3.273)	1.558 (0.918, 2.365)
AAPC on ASMR, 1990–2019	−2.349 (−2.554, −2.144)	−4.794 (−4.934, −4.655)	−4.335 (−4.491, −4.180)	−2.522 (−2.717, −2.326)	−2.311 (−2.470, −2.153)
Latin America and Caribbean					
Deaths in 2019 (million)	0.009 (0.006, 0.012)	0.050 (0.033, 0.071)	0.030 (0.021, 0.041)	0.016 (0.011, 0.022)	0.024 (0.016, 0.033)
ASMR in 2019 (per 100,000)	2.723 (1.884, 3.662)	15.816 (10.416, 22.452)	9.449 (6.590, 12.896)	5.338 (3.618, 7.334)	7.631 (5.071, 10.443)
AAPC on ASMR, 1990–2019	−0.376 (−0.582, −0.169)	−1.236 (−1.428, −1.044)	−1.930 (−2.214, −1.644)	−1.143 (−1.343, −0.942)	0.654 (0.535, 0.773)
North Africa and Middle East					
Deaths in 2019 (million)	0.015 (0.011, 0.019)	0.178 (0.132, 0.230)	0.075 (0.057, 0.095)	0.021 (0.015, 0.028)	0.020 (0.014, 0.026)
ASMR in 2019 (per 100,000)	6.480 (4.689, 8.480)	80.323 (59.265, 103.651)	34.847 (26.360, 44.213)	10.767 (7.704, 14.327)	9.552 (6.755, 12.553)
AAPC on ASMR, 1990–2019	0.887 (0.625, 1.150)	−0.219 (−0.603, 0.166)*	0.074 (−0.122, 0.270)*	0.019 (−0.424, 0.464)*	1.160 (0.856, 1.465)
South Asia					
Deaths in 2019 (million)	0.024 (0.016, 0.033)	0.386 (0.279, 0.502)	0.214 (0.152, 0.280)	0.308 (0.224, 0.395)	0.051 (0.034, 0.070)
ASMR in 2019 (per 100,000)	3.190 (2.170, 4.305)	51.659 (37.205, 67.311)	29.506 (20.946, 38.746)	49.235 (35.872, 63.214)	7.732 (5.080, 10.572)
AAPC on ASMR, 1990–2019	3.360 (2.854, 3.868)	2.796 (2.019, 3.578)	1.989 (0.998, 2.990)	1.597 (0.628, 2.576)	4.345 (3.527, 5.170)
Southeast Asia, East Asia, and Oceania					
Deaths in 2019 (million)	0.191 (0.136, 0.253)	0.451 (0.333, 0.577)	0.650 (0.488, 0.817)	0.295 (0.227, 0.386)	0.058 (0.040, 0.078)
ASMR in 2019 (per 100,000)	13.279 (9.488, 17.539)	34.276 (25.273, 43.904)	47.851 (35.889, 60.225)	25.060 (19.193, 32.924)	4.237 (2.901, 5.672)
AAPC on ASMR, 1990–2019	3.134 (2.861, 3.408)	2.804 (2.497, 3.113)	0.946 (0.618, 1.274)	−2.292 (−2.776, −1.806)	2.998 (2.758, 3.239)
Sub-Saharan Africa					
Deaths in 2019 (million)	0.005 (0.003, 0.007)	0.035 (0.022, 0.052)	0.042 (0.026, 0.061)	0.014 (0.009, 0.019)	0.013 (0.008, 0.020)
ASMR in 2019 (per 100,000)	2.126 (1.372, 2.982)	16.111 (10.044, 23.725)	18.795 (12.017, 27.318)	7.115 (4.904, 9.843)	6.543 (4.133, 9.433)
AAPC on ASMR, 1990–2019	1.873 (1.669, 2.076)	2.051 (1.747, 2.356)	1.748 (1.418, 2.078)	0.964 (0.652, 1.277)	3.143 (2.886, 3.400)

TBL, tracheal, bronchus, and lung; IHD, ischemic heart disease; COPD, chronic obstructive pulmonary disease; ASMR, age-standardized mortality rate; AAPC, average annual percent change. ^*^NOT statistically significant since *p* value > 0.05.

### 3.2 Trends of IHD due to ambient particulate matter

In 2019, IHD attributed to ambient particulate matter had an overall ASMR of 30 per 10^5^ and an ASDR of 700 per 10^5^, with Uzbekistan recording the highest ASMR and Egypt the highest ASDR (Table 1; Supplementary Tables S1–S3; Figure 1; Supplementary Figure S1). From 1990 to 2019, the total ASMR and ASDR displayed only slight fluctuations, with Norway showing the lowest AAPCs, and the highest observed in Equatorial Guinea (ASMR) and Bhutan (ASDR) (Table 1; Supplementary Tables S1–S3; Figure 2; Supplementary Figure S2). In North Africa and Middle East, the trend was uncertain though rates remained highest, whereas Southeast Asia, East Asia, and Oceania, as well as South Asia and sub-Sahara, saw a notable increase in burden (Figure 3; Supplementary Figure S3). High-middle SDI areas initially had the highest ASMR and ASDR but showed continuous declines, whereas middle SDI areas later ranked first with persistent growth; a pattern echoed in ages 70–94 (Figures 3, 4; Supplementary Figures S3, S4).

### 3.3 Trends of stroke due to ambient particulate matter

In 2019, stroke attributable to ambient particulate matter reached an ASMR of 26 per 10^5^ and an ASDR of 630 per 10^5^, with the highest rates recorded in North Macedonia and Mongolia, respectively (Table 1; Supplementary Tables S1–S3; Figure 1; Supplementary Figure S1). From 1990 to 2019, ASMR showed no clear change, while ASDR rose significantly (AAPC: 0.30), with Estonia recording the lowest AAPCs and Cabo Verde and Equatorial Guinea the highest for ASMR and ASDR, respectively (Table 1; Supplementary Tables S1–S3; Figure 2; Supplementary Figure S2). Although Southeast Asia, East Asia, and Oceania took the lead after 2000, the marked growth in South Asia and Sub-Saharan Africa remained noteworthy (Figure 3; Supplementary Figure S3). High-middle SDI areas initially held the highest ASMR and ASDR before declining, but after 2000, middle SDI regions surged to the lead with persistent growth, a shift also reflected in ages 65–94 (Figures 3, 4; Supplementary Figures S3, S4).

### 3.4 Trends of COPD due to ambient particulate matter

In 2019, COPD due to ambient particulate matter showed global ASMR and ASDR of 16 and 350 per 10^5^, respectively, with Nepal reporting the highest rates (Table 1; Supplementary Tables S1–S3; Figure 1; Supplementary Figure S1). From 1990 to 2019, ASMR and ASDR declined significantly, with AAPCs of −0.47 and −0.34, respectively; Nicaragua and Equatorial Guinea recorded the highest values, while Singapore and Lithuania had the lowest for ASMR and ASDR, respectively (Table 1; Supplementary Tables S1–S3; Figure 2; Supplementary Figure S2). The dominance of Southeast Asia, East Asia, and Oceania in ASMR or ASDR gradually diminished due to continuous declines, while South Asia overtook them after 2005, showing uninterrupted growth thereafter (Figure 3; Supplementary Figure S3). ASMR and ASDR, initially highest in middle SDI regions, declined steadily, while low-middle SDI regions later assumed the lead with sustained growth, paralleling rates in ages 70–84 (Figures 3, 4; Supplementary Figures S3, S4).

### 3.5 Trends of diabetes mellitus due to ambient particulate matter

In 2019, the global ASMR and ASDR for diabetes mellitus due to ambient particulate matter were 4.5 and 200 per 10^5^, respectively, with Bahrain ranking first worldwide (Table 1; Supplementary Tables S1–S3; Figure 1; Supplementary Figure S1). From 1990 to 2019, significant upward trends were discovered in ASMR (AAPC: 1.57) and ASDR (AAPC: 2.2), with Cabo Verde and Equatorial Guinea showing the highest increases, and Singapore and Sweden the lowest (Table 1; Supplementary Tables S1–S3; Figure 2; Supplementary Figure S2). Except for high-income regions, all GBD super-regions showed some growth in ASMR and ASDR, while North Africa and the Middle East remained the top-ranked regions (Figure 3; Supplementary Figure S3). ASMR and ASDR increased in all SDI regions except high-SDI, with middle-SDI regions leading; parallel trends were observed in death and DALY rates among ages 60–94 and 45–94, respectively (Figures 3, 4; Supplementary Figures S3, S4).

### 3.6 Decomposition of burden changes due to ambient particulate matter

**Figure 5:**
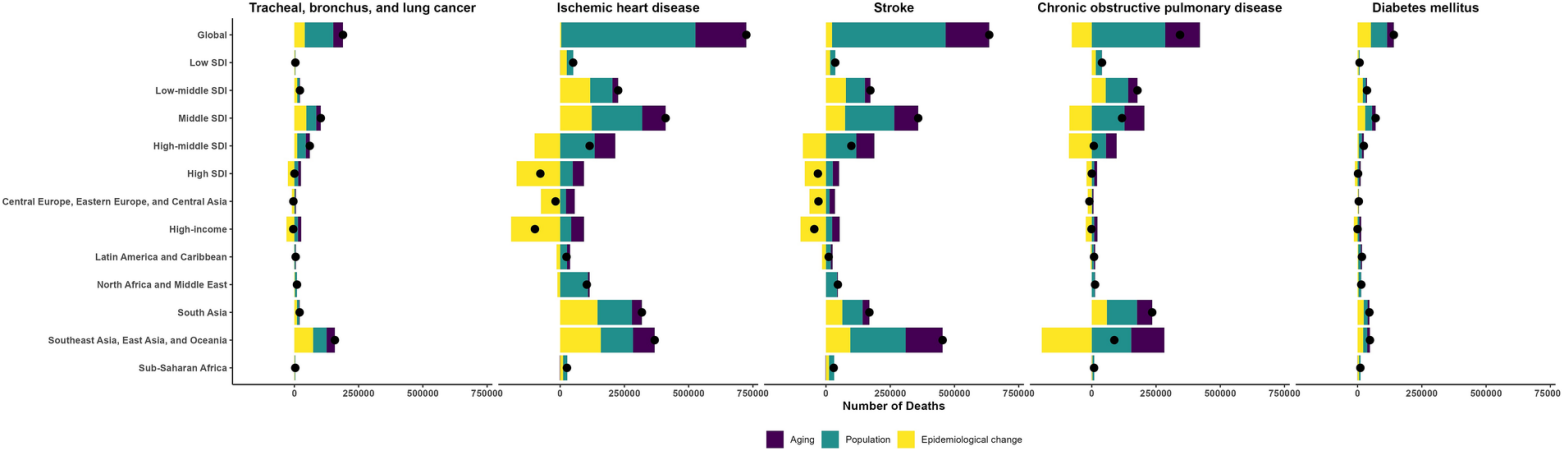
The changes in the deaths of ambient particulate matter-attributed non-communicable diseases driven by aging, population growth, and epidemiological change from 1990 to 2019.

Between 1990 and 2019, population growth and aging largely drove the increases in deaths and DALYs for TBL cancer, IHD, stroke, COPD, and diabetes mellitus (Figure 5; Supplementary Figure S5; Supplementary Tables S4, S5). More precisely, for TBL cancer, population growth and aging accounted for 59 and 20% of the rise in deaths, and 66 and 18% of the increment in DALYs; for IHD, the corresponding contributions were 72 and 27% for deaths, and 71 and 18% for DALYs; for stroke, 70 and 27% for deaths, and 70 and 18% for DALYs; for COPD, 83 and 39% for deaths, and 86 and 30% for DALYs; and for diabetes, 45 and 19% for deaths, and 42 and 11% for DALYs.

### 3.7 Correlation between SDI and burdens due to ambient particulate matter

**Figure 6:**
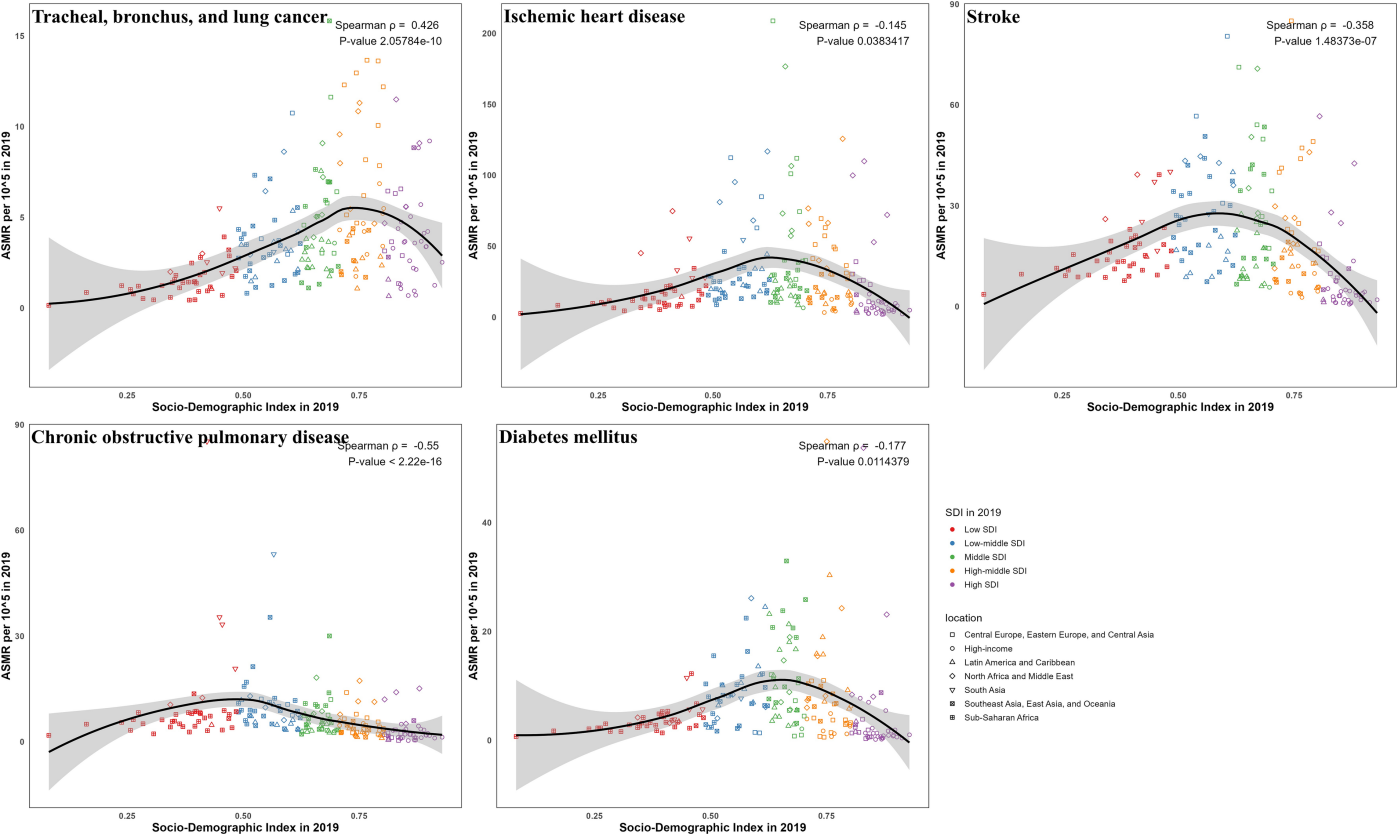
The correlation between SDI and ASMR in 2019 for ambient particulate matter-attributed non-communicable diseases.

**Figure 7:**
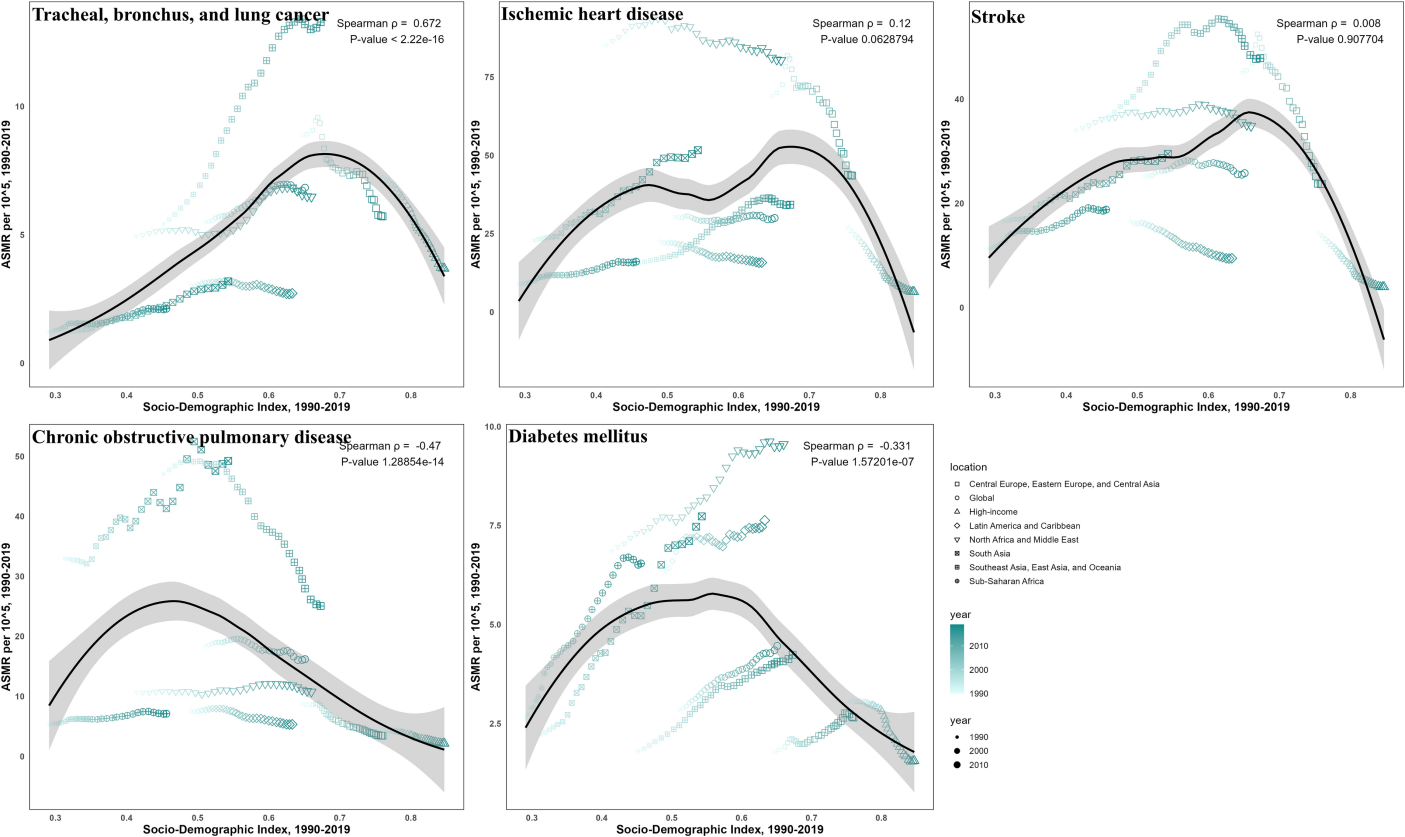
The correlation between SDI and ASMR from 1990 to 2019 for ambient particulate matter-attributed non-communicable diseases.

Correlations with SDI showed disease-specific disparities for ASMR (Figures 6, 7) and ASDR (Supplementary Figures S6, S7). For TBL cancer, both ASMR and ASDR had a significant positive correlation with SDI in 2019 (*ρ* = 0.43 and 0.40, respectively; *p* < 0.05), a trend that persisted from 1990 to 2019 (both *ρ* = 0.67; *p* < 0.05). In contrast, IHD exhibited a pronounced negative correlation in 2019 (*ρ* = −0.145 and −0.170, respectively; *p* < 0.05), though this pattern was not consistent over time. Stroke also demonstrated a negative correlation in 2019 (both *ρ* = −0.36; *p* < 0.05), which did not persist across past 30 years. For COPD, the negative correlation observed in 2019 (both *ρ* = −0.55; *p* < 0.05) was consistent with the long-term trend (*ρ* = −0.47 and −0.49, respectively; *p* < 0.05). Finally, for diabetes, only ASMR (*ρ* = −0.177; *p* < 0.05), not ASDR, was negatively correlated with SDI in 2019, continuing a trend evident since 1990 (*ρ* = −0.33; *p* < 0.05).

### 3.8 Health inequality in burdens due to ambient particulate matter by SDI

**Figure 8:**
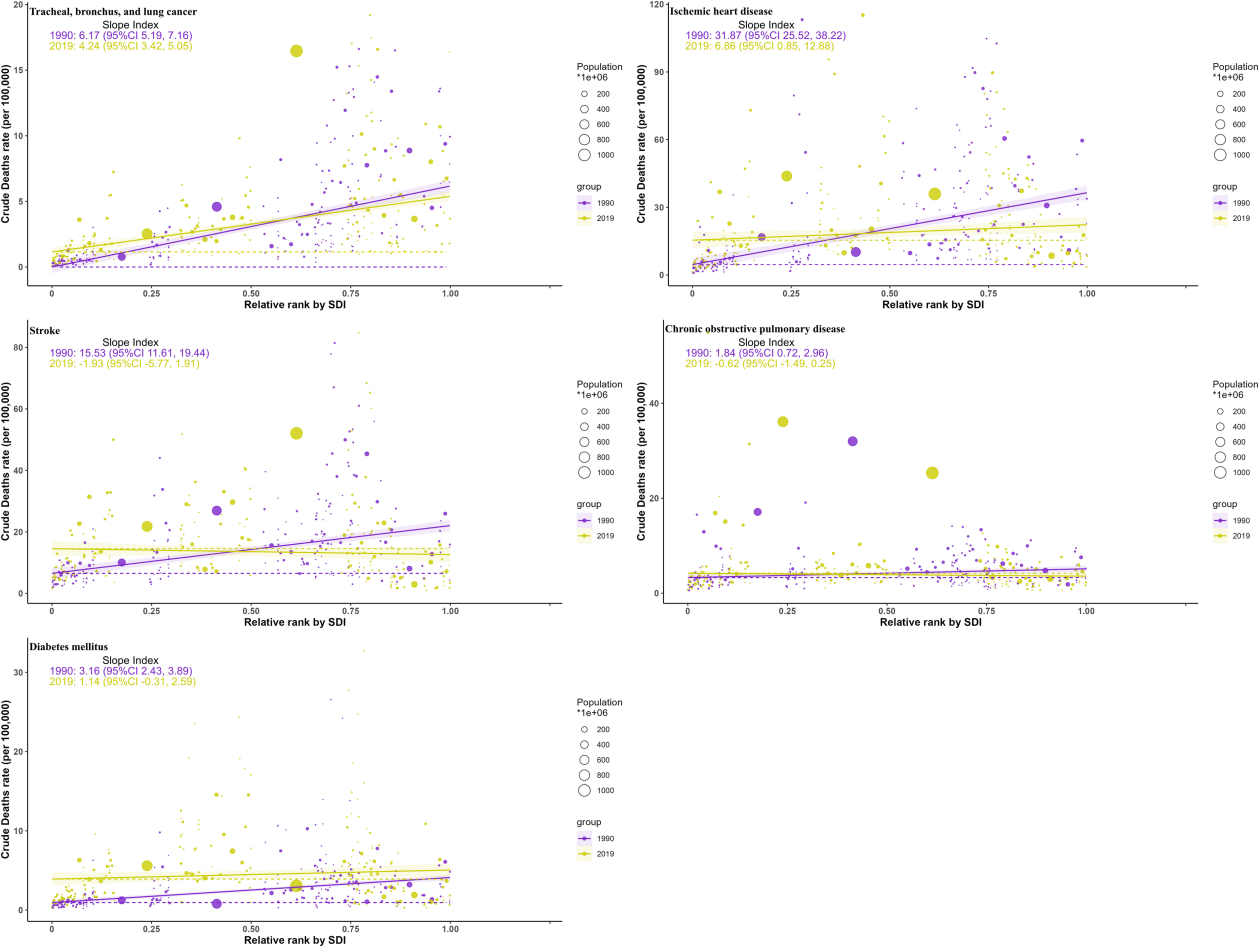
Health inequality curves for the deaths of ambient particulate matter-attributed non-communicable diseases from 1990 to 2019 across the world.

**Figure 9:**
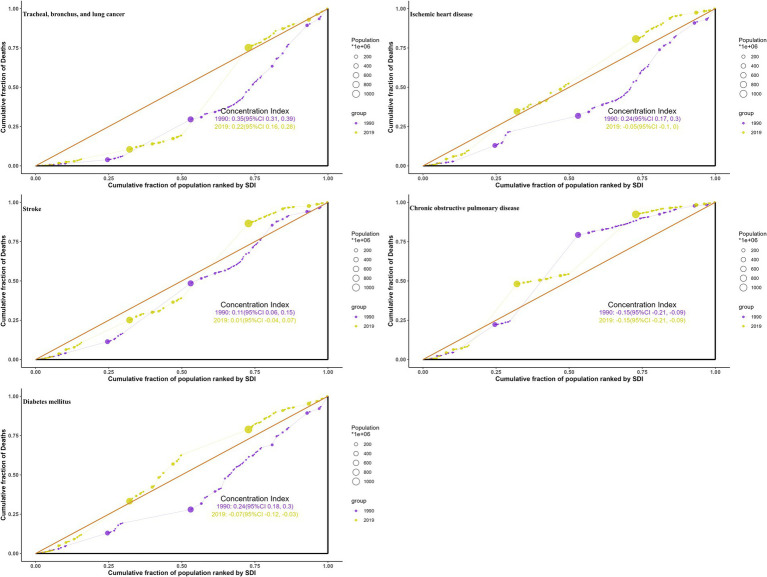
Health concentration curves for the deaths of ambient particulate matter-attributed non-communicable diseases from 1990 to 2019 across the world.

Between 1990 and 2019, health inequalities in burdens from ambient particulate matter across SDI levels, as reflected by the slope (Figure 8; Supplementary Figure S8) and concentration indices (Figure 9; Supplementary Figure S9), generally narrowed for all five diseases, with a few metrics turning negative. For TBL cancer, the slope index in death rates declined from 6.2 to 4.2 and the concentration index from 0.35 to 0.22, with comparable declines in DALYs. For IHD, values decreased from 32 to 6.9 and from 0.24 to −0.05 in death rates, respectively, with similar trends in DALYs. Stroke showed reductions from 16 to −1.93 and from 0.107 to 0.0118 in death rates, respectively, mirrored by those in DALYs. For COPD, the slope index of death rate fell from 1.84 to −0.62, while the concentration index remained stable; DALYs followed the same pattern. For diabetes, declines were observed from 3.2 to 1.14 and from 0.24 to −0.074 in death rates, respectively, with changes paralleling mortality in DALYs.

## 4 Discussion

The current research investigated the burden of non-communicable diseases linked to ambient particulate pollution from 1990 to 2019. Distinct from prior GBD studies that typically focused on a single disease, this job compared five major non-communicable diseases to provide a more integrated perspective. Substantial declines were observed in COPD, but upward trends were evident in TBL cancer and diabetes. By incorporating inequality metrics, clearly persistent cross-country disparities were also identified, with disproportionate increases in low-SDI regions such as South Asia and Sub-Saharan Africa. Together, these findings not only refine the understanding of disease-specific trends but also emphasize global health inequities that warrant policy attention.

It was observed in 2019 that although males generally suffered from a higher particle pollutants-induced chronic disease burden than females, an exception occurred among senior citizens over 75 years old with females surpassing males, particularly in diabetes mellitus (Supplementary Figures S10, S11). Some scholars have previously identified notable gender differences in short-term health effects associated with ambient atmospheric pollution, particularly in hospitalization and mortality rates for respiratory and circulatory issues (18). However, further exploration is required to understand the patterns of gender differences in the relationship between long-term exposure and health response. It should be noted that sex-stratified results are beyond the scope of this study, as the principal focus is cross-country inequality in non-communicable disease burden. The observed gender patterns, including a generally higher burden in males and a diabetes-specific exception among older females, especially postmenopausal women surpassing men, have been noted in prior literature (19). Future work with sex-specific data is warranted to better elucidate the mechanisms underlying the observed diabetes-related gender differences in older adults.

Another noteworthy discovery was that the peak age for DALYs of the same disease tended to precede the peak age for mortality, suggesting that disability and premature death among middle-aged would result in a greater loss of life expectancy, yielding more adverse socioeconomic consequences (20). Notably, although older adults exhibit higher absolute mortality rates, the earlier peak of DALYs indicates that premature deaths in midlife lead to greater losses of life expectancy, as individuals are deprived of decades of potential working years, family responsibilities, and social contributions, thereby offering a complementary view to mortality-based assessments. Therefore, the target population for future interventions addressing the health hazards of ambient airborne particles should gradually transition to younger cohorts. Actually, as this study has uncovered, population growth emerged as the primary determinant of changes in the burden of particulate matter-attributed chronic diseases.

The cross-region inequalities in particulate pollution hazards not only existed but also evolved over time, necessitating adaptable adjustments to complementary intervention strategies whenever necessary. As early as the GBD 2015 study, PM2.5 was identified as the fifth leading risk factor for mortality in 2015, with 59% of the related deaths occurring in East and South Asia (21). Extending these findings, this study reveals that over the last three decades, particle pollutant-related disease burden in higher-SDI regions has either experienced a decrease or continued to rise with a noticeably slowed momentum, while the burden has consistently increased without interruption in regions with lower SDI. The observed divergent trends across SDI levels can be attributed to multiple factors. In higher-SDI areas, slower population growth, effective air quality regulations, and better healthcare infrastructure likely contributed to decreases in non-communicable disease burden attributed to ambient particulate matter. Conversely, lower SDI-areas experienced rapid population growth, ongoing industrialization with limited pollution control, and under-resourced healthcare systems, collectively driving the observed increases burden.

Similarly, during the same period, nearly all slope and concentration indices have decreased from initially higher positive values to lower positives or even negatives, strongly suggesting a gradual shift of health consequences associated with atmospheric particulate pollution from developed to less developed regions. Interestingly, a survey focusing on the health impacts of PM2.5 also confirmed that between 1960 and 2019, despite a decrease in attributable health burdens in Europe and the United States, Asian countries with China and India as representatives continued to dominate the increase in attributable health burdens (22). The conditions in sub-Sahara and South Asia were observed to be relatively unfavorable, which might be ascribed to the rapid population growth, insufficiency of healthcare facilities, and underdevelopment of environmental surveillance. Consequently, attention should be paid to the potential for future climate change to exacerbate existing health inequalities, especially in vulnerable regions and populations in sub-Saharan Africa and South Asia (23).

During global monitoring from 1998 to 2018, it was observed that more than 60% of people in East Asia and South Asia were perpetually exposed to PM2.5 levels above 35 μg/m^3^ per year, greatly exceeding the WHO’s 2021 guideline of 5 μg/m^3^, which could also account for why the atmospheric particulates-attributed TBL cancer burden had been substantially greater in Southeast Asia, East Asia, and Oceania since the year 2000 (24). Mechanistically, inhaled particulate matter can trigger lung inflammation and DNA damage, promoting tumor development. However, since Asia has become the biggest producer and consumer of tobacco worldwide, it is difficult to attribute local TBL cancer exclusively to particulate matter (25). According to a U.S. investigation, long-term exposure to PM2.5 could modestly elevate the lung cancer mortality rate among lifelong never-smokers, and similar risks are likely to exist among Asian non-smokers (26). From 2010 on, the slowdown in haze-reduced TBL cancer growth rates in East Asia and Southeast Asia could be primarily ascribed to the drastic reduction in air pollutant emissions, thanks to the Clean Air Action enforced by leading countries, notably China (27). In 2019, the ASMR for TBL cancer in high-middle SDI regions was higher than that for other non-communicable diseases. This elevated burden may be attributed to a combination of elevated ambient particulate exposure, high smoking prevalence, and industrialization-related environmental pollution. These factors likely act synergistically to increase the lung cancer burden in these regions, despite relatively better healthcare infrastructure and declining trends in other non-communicable diseases.

It has been noted that over the past three decades, North Africa and Middle East have continually topped the charts of ambient particulate-induced IHD burdens, with early research convincingly attributing this to a substantial rise in local particulate pollution exposure during the same period (28). Literature on the health effects of air pollution in this region is relatively scarce, however, a few foundational studies, for example, the Isfahan case-crossover design in Iran, have verified the correlation between exposure to PM2.5 and PM10-2.5 and cardiovascular disease hospitalizations (29). Underlying mechanisms shows that particulate matter can trigger systemic inflammation, oxidative stress, and endothelial dysfunction, promoting ischemic heart disease. Additionally, age-standardized rates of particulate matter-attributed IHD burden were found to be negatively correlated with the SDI in 2019, with concentration indices declining from positive values in 1990 to negative values in 2019. Such alteration signified a gradual relocation of airborne particle-induced IHD burden from developed to developing countries, indicating the growth in South Asia and Sub-Saharan Africa, exemplified by Bhutan and Equatorial Guinea, respectively, was of special interest.

We’ve noticed that the burden of particulate pollution-induced stroke in Central Europe Eastern Europe, and Central Asia, was once the highest globally, hitting its peak in 1995 before gradually declining, which aligned with the observed decrease in PM2.5 levels in rural Central Europe between the mid-1990s and 2009/10 (30). Beginning in 2000, Southeast Asia, East Asia, and Oceania have experienced a growing burden of airborne particle-attributed stroke, progressively widening the disparity with other regions. This emphasizes the importance of addressing not only conventional stroke risk factors like smoking and hypertension prevalent in Asians but also the potential threats posed by particulate pollution (31). Functionally, inhaled particulate matter can trigger systemic inflammation, endothelial dysfunction, and atherosclerosis, thereby increasing the risk of stroke. The shift of both stroke mortality and DALY rates attributable to particle pollutants from positive values in 1990 to negative values in 2019 signaled a reversal of cross-region inequality, with the burden transferred from higher- to lower-income nations. Hence, it is imperative to consider particulate matter as a risk factor when formulating stroke intervention strategies in underdeveloped areas.

Despite significant reductions in airborne particulate-induced COPD across most areas in the last 30 years, the increasing trends in South Asia and Sub-Saharan Africa, notably in low/lower-middle SDI regions, remained concerning. Earlier spatiotemporal studies also noted the annual increase in PM2.5 concentrations from 1998 to 2016 in India and the Sub-Sahara, closely correlated with population growth, in stark contrast to the “decoupling” phenomenon observed in high-income Western countries, where PM2.5 levels and population growth have diverged (32). Such increasing trend is mechanistically linked to chronic inhalation of fine particulate matter, causing airway inflammation and progressive lung function decline, which elevates COPD risk and mortality. South Asia, particularly Nepal, has faced a much more severe threat, recording the highest global ASMR and ASDR in 2019, while in India, 54.5% of premature deaths caused by PM2.5 in the first decade of this century were attributable to COPD (33). Moreover, recent analyses have revealed extreme cross-region inequality in the burden of particulate pollution-related COPD, evidenced by a negative correlation between age-standardized rates and SDI, and a shift in the slope index of inequality from positive to negative values.

Over the past three decades, a global increase in the burden of diabetes mellitus induced by particulate matter was spotted, except in high-income countries. This increase was particularly pronounced in LMICs, aligning with previous findings (34). In North Africa and Middle East, not only did the burden rank the highest, but it also continued to rise, consistent with another review that noted the ongoing concerns about the rising incidence of diabetes in the Middle East and the failure to translate these concerns into effective action (35). The circumstances in Latin America and Caribbean, coming in second, were also not optimistic, as reviews have suggested that the actual numbers of diabetes cases were likely underestimated (36). Traditionally, obesity, dyslipidemia, and hypertension have been recognized as primary risk factors for diabetes mellitus. Biologically, chronic exposure to particulate matter may induce systemic inflammation and insulin resistance, contributing to the development of diabetes. However, the role of particulate matter exposure in the incidence of diabetes mellitus still demands further population-based studies to establish strong causal relationships, especially in less developed regions.

The disparities observed in particulate matter-attributed disease burden suggest the need for multi-level interventions. Evidence indicates that modest improvements in PM2.5 can lead to substantial health benefits in relatively clean regions such as North America and Europe, whereas heavily polluted regions like China and India require major reductions to achieve comparable gains, underscoring pronounced geographic inequalities in the global air pollution burden (37). Implementation of WHO Air Quality Guidelines and emission control policies could reduce population exposure, while climate change mitigation efforts may provide co-benefits for air quality. The health and economic costs associated with air pollution are largely preventable, making it critical to assess their scale for guiding policies and interventions (38). Moreover, strengthening health system preparedness, particularly in low- and middle-SDI regions, is important to manage uprising rates of respiratory, cardiovascular, and metabolic diseases. These measures collectively highlight potential strategies to mitigate health inequalities and inform future public health planning, though further context-specific analyses are warranted.

Before coming to the conclusions, some inherent limitations of this study should be acknowledged. Although GBD 2019 reports health outcomes from ambient particulate matter, it omitted some conditions (e.g., asthma, Alzheimer’s) and considered only mortality and DALYs, excluding incidence due to survey challenges. Secondly, while GBD 2019 provided authoritative global estimates of particulate matter-related disease burden, its reliance on annual average concentrations at coarse spatial resolution limited the ability to capture local variations in exposure, seasonal patterns, and short-term peak events (39). Third, inconsistencies arising from variable data quality and surveillance delays may have compromised the reliability of these findings. Moreover, some other cohort-based models like the Global Exposure Mortality Model (GEMM) suggested that estimates of PM2.5-attributed burden may be underestimated, particularly at high exposure levels, due to assumptions of equal toxicity across particle sources and the inclusion of non-outdoor exposure (40). In addition, excluding populations under 25 may underestimate overall disease burden, potentially overlooking early-onset cases of conditions such as diabetes. Finally, this study relied on the widely cited GBD 2019 estimates, but newer iterations (e.g., GBD 2021) may reflect significant methodological refinements that could inform future analyses (41).

## 5 Conclusion

This study not only highlighted the unfavorable global situation regarding interventions for chronic diseases attributed to ambient particulate matter, primarily lower respiratory tract cancers and diabetes mellitus, over the last three decades but also underscored the presence of cross-region inequalities in the atmospheric aerosol-induced health impacts, with the particularly alarming persistent and rapid growth in underdeveloped areas like South Asia and Sub-Saharan Africa. Therefore, the future management of air pollution-related chronic diseases should pay closer attention to the rational allocation of health resources across different populations and regions.

## Data Availability

Publicly available datasets were analyzed in this study. This data can be found at: Global Burden of Disease 2019 datasets (https://vizhub.healthdata.org/gbd-results/).

## Footnotes

^1^ https://vizhub.healthdata.org/gbd-results/
