# Supplementary material for: The global burden, trends and cross-region inequities of non-communicable diseases attributed to ambient particulate matter pollution
Source: Front Public Health. 2025 Nov 3;13:1682574. doi: 10.3389/fpubh.2025.1682574 (PMC12620374; doi:10.3389/fpubh.2025.1682574)
Supplement: Supplementary file 1 [file Data_Sheet_1.pdf]

Socio-demographic index (SDI) outcomes for 204 countries and territories spanning from 1990 to 2019

Location	1990	1991	1992	1993	1994	1995	1996	1997	1998	1999	2000	2001	2002	2003	2004	2005	2006	2007	2008	2009	2010	2011	2012	2013	2014	2015	2016	2017	2018	2019
Afghanistan	0.187	0.191	0.195	0.196	0.194	0.194	0.193	0.192	0.19	0.189	0.188	0.188	0.194	0.202	0.209	0.216	0.224	0.234	0.243	0.253	0.264	0.274	0.285	0.295	0.304	0.313	0.321	0.329	0.337	0.343
Albania	0.54	0.537	0.534	0.533	0.535	0.538	0.544	0.549	0.555	0.561	0.569	0.577	0.585	0.593	0.601	0.608	0.615	0.621	0.627	0.631	0.636	0.64	0.645	0.651	0.658	0.664	0.669	0.674	0.678	0.681
Algeria	0.436	0.446	0.456	0.465	0.474	0.483	0.492	0.5	0.509	0.518	0.526	0.534	0.542	0.55	0.558	0.566	0.573	0.58	0.587	0.593	0.599	0.605	0.611	0.617	0.623	0.628	0.634	0.64	0.646	0.652
American Samoa	0.606	0.609	0.613	0.616	0.619	0.623	0.627	0.63	0.634	0.637	0.641	0.645	0.649	0.652	0.656	0.66	0.663	0.667	0.671	0.674	0.678	0.682	0.686	0.69	0.694	0.698	0.702	0.706	0.709	0.712
Andorra	0.834	0.838	0.84	0.841	0.841	0.841	0.843	0.845	0.847	0.849	0.851	0.854	0.855	0.859	0.862	0.865	0.867	0.869	0.872	0.874	0.876	0.879	0.881	0.883	0.885	0.887	0.889	0.891	0.892	0.894
Angola	0.238	0.243	0.247	0.25	0.253	0.257	0.262	0.267	0.273	0.278	0.283	0.289	0.296	0.303	0.311	0.319	0.33	0.341	0.353	0.364	0.376	0.387	0.398	0.41	0.421	0.432	0.443	0.454	0.463	0.47
Antigua and Barbuda	0.579	0.586	0.592	0.598	0.604	0.61	0.616	0.623	0.631	0.638	0.645	0.652	0.659	0.665	0.672	0.679	0.686	0.694	0.7	0.705	0.709	0.713	0.716	0.719	0.723	0.727	0.731	0.735	0.739	0.743
Argentina	0.581	0.585	0.593	0.599	0.605	0.611	0.617	0.622	0.625	0.628	0.634	0.637	0.64	0.641	0.642	0.649	0.653	0.655	0.657	0.661	0.665	0.67	0.674	0.677	0.679	0.687	0.696	0.702	0.706	0.708
Armenia	0.536	0.541	0.541	0.542	0.544	0.546	0.55	0.554	0.559	0.564	0.57	0.577	0.586	0.596	0.606	0.616	0.626	0.637	0.647	0.652	0.658	0.662	0.666	0.67	0.673	0.676	0.679	0.682	0.686	0.689
Australia	0.738	0.741	0.745	0.749	0.753	0.757	0.761	0.766	0.77	0.774	0.778	0.782	0.787	0.791	0.795	0.797	0.798	0.799	0.802	0.806	0.809	0.812	0.815	0.82	0.824	0.828	0.832	0.834	0.837	0.839
Austria	0.753	0.754	0.757	0.761	0.768	0.773	0.778	0.783	0.787	0.791	0.795	0.799	0.803	0.805	0.808	0.811	0.815	0.818	0.821	0.824	0.826	0.83	0.833	0.835	0.838	0.839	0.841	0.844	0.847	0.849
Azerbaijan	0.576	0.578	0.579	0.578	0.576	0.573	0.569	0.565	0.561	0.559	0.559	0.561	0.564	0.569	0.575	0.583	0.594	0.607	0.619	0.628	0.637	0.645	0.652	0.658	0.664	0.669	0.673	0.677	0.68	0.683
Bahamas	0.692	0.684	0.676	0.679	0.686	0.692	0.697	0.702	0.71	0.721	0.731	0.735	0.737	0.739	0.742	0.749	0.755	0.759	0.762	0.767	0.774	0.779	0.78	0.782	0.784	0.786	0.789	0.791	0.794	0.796
Bahrain	0.553	0.56	0.566	0.573	0.58	0.587	0.595	0.602	0.61	0.618	0.626	0.634	0.643	0.653	0.662	0.672	0.68	0.688	0.697	0.705	0.711	0.715	0.72	0.726	0.731	0.735	0.739	0.743	0.747	0.751
Bangladesh	0.267	0.275	0.283	0.29	0.297	0.304	0.311	0.317	0.324	0.33	0.336	0.343	0.349	0.355	0.362	0.369	0.376	0.384	0.392	0.4	0.408	0.416	0.425	0.433	0.441	0.449	0.457	0.466	0.475	0.483
Barbados	0.649	0.653	0.658	0.663	0.667	0.67	0.672	0.674	0.676	0.677	0.68	0.683	0.687	0.691	0.695	0.699	0.703	0.707	0.711	0.714	0.718	0.721	0.725	0.728	0.73	0.733	0.735	0.737	0.74	0.742
Belarus	0.591	0.595	0.6	0.606	0.611	0.614	0.618	0.62	0.621	0.624	0.629	0.635	0.642	0.65	0.658	0.665	0.671	0.678	0.687	0.695	0.703	0.709	0.713	0.719	0.725	0.73	0.734	0.738	0.742	0.745
Belgium	0.746	0.75	0.756	0.762	0.767	0.771	0.775	0.779	0.782	0.784	0.787	0.792	0.796	0.799	0.802	0.805	0.808	0.81	0.813	0.816	0.82	0.824	0.829	0.834	0.837	0.841	0.843	0.846	0.849	0.851
Belize	0.428	0.437	0.447	0.457	0.466	0.475	0.482	0.488	0.494	0.499	0.505	0.51	0.515	0.521	0.528	0.534	0.541	0.547	0.553	0.559	0.564	0.569	0.574	0.579	0.583	0.588	0.592	0.596	0.6	0.603
Benin	0.209	0.213	0.218	0.222	0.227	0.231	0.236	0.241	0.245	0.25	0.254	0.259	0.263	0.267	0.271	0.275	0.279	0.283	0.288	0.292	0.297	0.301	0.306	0.312	0.318	0.324	0.331	0.338	0.346	0.352
Bermuda	0.685	0.689	0.693	0.697	0.701	0.704	0.707	0.71	0.714	0.719	0.724	0.731	0.737	0.743	0.749	0.755	0.76	0.766	0.773	0.779	0.785	0.79	0.795	0.799	0.802	0.805	0.807	0.809	0.811	0.813
Bhutan	0.228	0.232	0.237	0.243	0.251	0.258	0.266	0.275	0.282	0.29	0.298	0.306	0.314	0.322	0.33	0.338	0.347	0.356	0.365	0.375	0.384	0.394	0.403	0.411	0.419	0.426	0.434	0.442	0.449	0.455
Bolivia (Plurinational State of)	0.412	0.417	0.422	0.428	0.434	0.441	0.448	0.455	0.462	0.469	0.474	0.48	0.485	0.491	0.496	0.501	0.506	0.51	0.514	0.518	0.521	0.525	0.528	0.533	0.538	0.544	0.55	0.556	0.562	0.566
Bosnia and Herzegovina	0.533	0.534	0.532	0.529	0.527	0.528	0.54	0.558	0.576	0.591	0.604	0.616	0.626	0.636	0.644	0.651	0.658	0.665	0.671	0.677	0.682	0.686	0.691	0.695	0.698	0.702	0.706	0.71	0.714	0.718
Botswana	0.431	0.441	0.451	0.459	0.467	0.475	0.483	0.491	0.498	0.506	0.514	0.521	0.528	0.535	0.541	0.548	0.555	0.562	0.569	0.575	0.581	0.587	0.593	0.6	0.606	0.612	0.618	0.624	0.63	0.634
Brazil	0.487	0.492	0.498	0.503	0.508	0.513	0.519	0.524	0.529	0.533	0.538	0.543	0.547	0.551	0.556	0.561	0.566	0.572	0.577	0.583	0.59	0.597	0.603	0.61	0.616	0.622	0.627	0.632	0.636	0.64
Brunei Darussalam	0.676	0.682	0.688	0.694	0.7	0.706	0.712	0.717	0.723	0.729	0.735	0.741	0.747	0.753	0.758	0.764	0.769	0.774	0.779	0.784	0.789	0.793	0.797	0.801	0.806	0.809	0.813	0.817	0.82	0.823
Bulgaria	0.631	0.641	0.648	0.656	0.666	0.671	0.676	0.681	0.677	0.675	0.68	0.688	0.693	0.697	0.701	0.706	0.71	0.715	0.718	0.724	0.733	0.737	0.74	0.743	0.746	0.75	0.752	0.755	0.76	0.764
Burkina Faso	0.125	0.128	0.131	0.134	0.137	0.14	0.144	0.147	0.152	0.156	0.161	0.166	0.17	0.175	0.18	0.186	0.191	0.196	0.2	0.205	0.21	0.215	0.22	0.226	0.231	0.236	0.241	0.247	0.252	0.257
Burundi	0.198	0.201	0.204	0.207	0.209	0.21	0.21	0.211	0.212	0.213	0.213	0.214	0.216	0.218	0.22	0.223	0.226	0.23	0.234	0.238	0.243	0.248	0.254	0.26	0.266	0.27	0.274	0.278	0.282	0.284
Cabo Verde	0.292	0.298	0.305	0.311	0.318	0.326	0.333	0.342	0.35	0.36	0.37	0.379	0.388	0.397	0.407	0.415	0.424	0.434	0.443	0.452	0.461	0.469	0.477	0.484	0.491	0.498	0.505	0.512	0.519	0.525
Cambodia	0.266	0.272	0.277	0.282	0.286	0.291	0.296	0.301	0.307	0.313	0.321	0.328	0.337	0.345	0.354	0.363	0.373	0.382	0.391	0.399	0.406	0.413	0.421	0.428	0.435	0.442	0.449	0.456	0.463	0.469
Cameroon	0.313	0.32	0.325	0.33	0.334	0.338	0.342	0.346	0.349	0.353	0.357	0.36	0.364	0.368	0.373	0.378	0.384	0.39	0.397	0.404	0.412	0.42	0.428	0.436	0.445	0.455	0.464	0.474	0.483	0.49
Canada	0.79	0.792	0.795	0.797	0.8	0.804	0.809	0.812	0.815	0.819	0.824	0.828	0.832	0.835	0.838	0.84	0.842	0.843	0.845	0.848	0.851	0.853	0.856	0.859	0.861	0.864	0.867	0.869	0.871	0.873
Central African Republic	0.186	0.19	0.193	0.196	0.199	0.202	0.205	0.208	0.211	0.215	0.218	0.221	0.225	0.227	0.23	0.233	0.236	0.24	0.244	0.248	0.253	0.258	0.263	0.263	0.263	0.264	0.266	0.268	0.271	0.274
Chad	0.108	0.112	0.115	0.118	0.12	0.123	0.125	0.128	0.13	0.132	0.135	0.137	0.14	0.144	0.149	0.156	0.162	0.167	0.173	0.179	0.185	0.191	0.197	0.203	0.21	0.216	0.223	0.228	0.234	0.238
Chile	0.592	0.6	0.606	0.611	0.617	0.624	0.63	0.637	0.644	0.651	0.657	0.663	0.671	0.678	0.683	0.686	0.689	0.692	0.695	0.7	0.706	0.712	0.719	0.724	0.728	0.738	0.747	0.753	0.756	0.759
China	0.433	0.441	0.45	0.459	0.469	0.479	0.489	0.499	0.508	0.516	0.525	0.534	0.543	0.552	0.561	0.571	0.581	0.591	0.601	0.611	0.621	0.631	0.638	0.646	0.654	0.657	0.659	0.669	0.679	0.686
Colombia	0.478	0.48	0.482	0.486	0.492	0.498	0.503	0.509	0.514	0.518	0.522	0.526	0.529	0.533	0.536	0.54	0.546	0.552	0.559	0.567	0.574	0.582	0.589	0.596	0.603	0.609	0.616	0.622	0.628	0.633
Comoros	0.274	0.281	0.288	0.296	0.302	0.309	0.315	0.322	0.328	0.334	0.34	0.346	0.353	0.359	0.365	0.371	0.378	0.384	0.39	0.396	0.401	0.407	0.413	0.419	0.426	0.432	0.438	0.444	0.45	0.455
Congo	0.364	0.373	0.381	0.389	0.396	0.403	0.41	0.416	0.421	0.426	0.431	0.437	0.442	0.447	0.452	0.458	0.464	0.469	0.475	0.482	0.491	0.5	0.509	0.519	0.528	0.538	0.547	0.556	0.563	0.568
Cook Islands	0.625	0.631	0.637	0.643	0.649	0.654	0.659	0.663	0.667	0.671	0.676	0.682	0.687	0.692	0.697	0.701	0.706	0.711	0.715	0.719	0.722	0.726	0.73	0.734	0.739	0.744	0.75	0.755	0.76	0.764
Costa Rica	0.532	0.538	0.544	0.55	0.555	0.56	0.565	0.571	0.577	0.584	0.592	0.598	0.603	0.607	0.611	0.615	0.619	0.623	0.627	0.632	0.637	0.642	0.647	0.652	0.657	0.662	0.667	0.672	0.676	0.68
Coted'Ivoire	0.256	0.26	0.265	0.27	0.275	0.279	0.285	0.29	0.295	0.301	0.305	0.31	0.314	0.318	0.322	0.325	0.329	0.333	0.337	0.341	0.346	0.35	0.355	0.362	0.369	0.376	0.384	0.393	0.401	0.408
Croatia	0.68	0.688	0.692	0.692	0.692	0.691	0.691	0.697	0.703	0.707	0.713	0.719	0.725	0.73	0.734	0.739	0.745	0.748	0.753	0.758	0.763	0.767	0.77	0.774	0.777	0.781	0.784	0.788	0.791	0.794
Cuba	0.578	0.586	0.589	0.588	0.586	0.583	0.583	0.582	0.579	0.581	0.586	0.588	0.595	0.602	0.605	0.611	0.618	0.618	0.616	0.618	0.62	0.624	0.631	0.636	0.64	0.645	0.653	0.66	0.665	0.668
Cyprus	0.662	0.67	0.68	0.691	0.702	0.713	0.723	0.732	0.741	0.75	0.758	0.767	0.774	0.78	0.786	0.791	0.797	0.804	0.81	0.816	0.82	0.824	0.827	0.829	0.831	0.832	0.834	0.836	0.838	0.841
Czechia	0.688	0.696	0.705	0.718	0.736	0.748	0.755	0.76	0.765	0.771	0.776	0.782	0.786	0.79	0.794	0.798	0.801	0.804	0.807	0.81	0.813	0.816	0.818	0.819	0.82	0.82	0.82	0.822	0.825	0.828
Democratic People's Republic of Korea	0.431	0.436	0.439	0.442	0.445	0.447	0.45	0.451	0.452	0.455	0.458	0.463	0.467	0.473	0.478	0.485	0.49	0.496	0.502	0.507	0.513	0.518	0.523	0.528	0.534	0.538	0.543	0.548	0.553	0.558
Democratic Republic of the Congo	0.26	0.262	0.263	0.263	0.262	0.261	0.259	0.256	0.253	0.25	0.246	0.242	0.239	0.238	0.239	0.242	0.244	0.247	0.251	0.257	0.266	0.277	0.289	0.305	0.321	0.336	0.35	0.362	0.374	0.382
Denmark	0.806	0.809	0.813	0.816	0.82	0.824	0.828	0.833	0.836	0.84	0.844	0.848	0.852	0.855	0.858	0.86	0.862	0.864	0.865	0.867	0.87	0.873	0.875	0.878	0.88	0.882	0.884	0.886	0.888	0.89
Djibouti	0.275	0.277	0.28	0.285	0.289	0.293	0.297	0.302	0.306	0.311	0.317	0.322	0.327	0.333	0.339	0.345	0.353	0.36	0.368	0.376	0.384	0.392	0.4	0.408	0.416	0.425	0.434	0.443	0.452	0.459
Dominica	0.579	0.585	0.591	0.597	0.604	0.61	0.617	0.623	0.63	0.637	0.643	0.649	0.655	0.66	0.666	0.672	0.677	0.683	0.689	0.695	0.7	0.705	0.709	0.713	0.717	0.721	0.724	0.727	0.728	0.729
Dominican Republic	0.425	0.427	0.43	0.434	0.437	0.442	0.446	0.452	0.458	0.465	0.471	0.478	0.485	0.492	0.499	0.508	0.516	0.524	0.532	0.538	0.544	0.549	0.554	0.559	0.563	0.569	0.574	0.58	0.587	0.592
Ecuador	0.503	0.506	0.51	0.514	0.517	0.521	0.524	0.528	0.532	0.535	0.539	0.544	0.549	0.554	0.559	0.565	0.57	0.575	0.581	0.586	0.591	0.597	0.603	0.609	0.615	0.621	0.626	0.632	0.636	0.64
Egypt	0.403	0.415	0.426	0.437	0.448	0.459	0.469	0.478	0.487	0.496	0.504	0.512	0.52	0.527	0.535	0.542	0.549	0.557	0.565	0.573	0.582	0.591	0.6	0.609	0.617	0.626	0.635	0.643	0.651	0.658
El Salvador	0.39	0.393	0.397	0.402	0.408	0.415	0.423	0.431	0.441	0.45	0.46	0.469	0.478	0.486	0.493	0.499	0.505	0.511	0.517	0.521	0.526	0.531	0.536	0.542	0.547	0.552	0.558	0.563	0.568	0.573
Equatorial Guinea	0.208	0.214	0.222	0.23	0.24	0.252	0.268	0.295	0.318	0.341	0.364	0.391	0.415	0.438	0.46	0.482	0.502	0.522	0.543	0.561	0.578	0.594	0.611	0.626	0.64	0.652	0.663	0.673	0.681	0.685
Eritrea	0.198	0.203	0.21	0.218	0.228	0.238	0.248	0.258	0.267	0.275	0.283	0.289	0.296	0.302	0.307	0.312	0.317	0.322	0.326	0.33	0.334	0.34	0.346	0.353	0.36	0.367	0.375	0.382	0.39	0.396
Estonia	0.665	0.676	0.687	0.696	0.7	0.705	0.711	0.717	0.721	0.726	0.733	0.741	0.748	0.753	0.759	0.765	0.771	0.777	0.785	0.792	0.798	0.804	0.809	0.813	0.817	0.821	0.825	0.829	0.833	0.835
Eswatini	0.392	0.401	0.41	0.419	0.427	0.435	0.443	0.45	0.456	0.462	0.468	0.474	0.479	0.484	0.49	0.495	0.501	0.507	0.513	0.519	0.526	0.532	0.538	0.545	0.551	0.557	0.563	0.569	0.574	0.577
Ethiopia	0.144	0.146	0.147	0.149	0.151	0.153	0.157	0.161	0.164	0.167	0.171	0.176	0.18	0.183	0.189	0.195	0.203	0.213	0.223	0.233	0.244	0.256	0.268	0.279	0.291	0.302	0.313	0.324	0.334	0.343
Fiji	0.527	0.532	0.538	0.544	0.55	0.555	0.561	0.567	0.572	0.577	0.582	0.587	0.592	0.596	0.601	0.605	0.609	0.612	0.616	0.619	0.622	0.626	0.63	0.635	0.639	0.644	0.649	0.654	0.659	0.664
Finland	0.757	0.759	0.762	0.765	0.769	0.773	0.777	0.782	0.785	0.788	0.792	0.797	0.802	0.805	0.808	0.812	0.815	0.818	0.821	0.824	0.828	0.831	0.834	0.837	0.84	0.844	0.848	0.851	0.853	0.856
France	0.738	0.743	0.75	0.755	0.759	0.763	0.767	0.77	0.773	0.775	0.777	0.781	0.785	0.787	0.79	0.793	0.796	0.799	0.801	0.803	0.806	0.809	0.812	0.815	0.819	0.822	0.826	0.829	0.832	0.834
Gabon	0.388	0.399	0.409	0.42	0.431	0.442	0.453	0.463	0.474	0.483	0.493	0.502	0.51	0.519	0.528	0.537	0.546	0.554	0.563	0.57	0.579	0.587	0.596	0.605	0.614	0.623	0.632	0.641	0.649	0.656
Gambia	0.218	0.223	0.227	0.232	0.237	0.241	0.246	0.251	0.256	0.262	0.268	0.274	0.28	0.286	0.293	0.3	0.306	0.313	0.319	0.327	0.334	0.341	0.348	0.356	0.363	0.37	0.378	0.385	0.393	0.399
Georgia	0.654	0.658	0.657	0.654	0.65	0.644	0.638	0.633	0.628	0.625	0.624	0.626	0.63	0.636	0.641	0.646	0.651	0.656	0.66	0.663	0.665	0.668	0.672	0.676	0.68	0.684	0.688	0.693	0.697	0.702
Germany	0.819	0.83	0.834	0.838	0.841	0.843	0.844	0.844	0.844	0.844	0.847	0.853	0.856	0.858	0.861	0.863	0.866	0.869	0.873	0.875	0.878	0.881	0.883	0.886	0.888	0.89	0.892	0.894	0.896	0.898
Ghana	0.355	0.362	0.368	0.374	0.38	0.385	0.391	0.396	0.401	0.406	0.411	0.415	0.42	0.426	0.431	0.437	0.444	0.45	0.458	0.466	0.474	0.484	0.494	0.504	0.514	0.523	0.531	0.541	0.549	0.557
Greece	0.682	0.688	0.695	0.702	0.707	0.713	0.717	0.723	0.729	0.735	0.74	0.743	0.748	0.753	0.758	0.761	0.765	0.768	0.771	0.775	0.779	0.782	0.785	0.786	0.786	0.787	0.788	0.79	0.792	0.794
Greenland	0.655	0.652	0.651	0.651	0.653	0.653	0.653	0.655	0.661	0.664	0.667	0.671	0.678	0.684	0.689	0.696	0.704	0.71	0.715	0.721	0.728	0.734	0.737	0.74	0.743	0.747	0.751	0.756	0.759	0.761
Grenada	0.463	0.474	0.485	0.495	0.505	0.515	0.524	0.533	0.543	0.553	0.562	0.57	0.577	0.585	0.592	0.6	0.606	0.613	0.619	0.624	0.628	0.633	0.637	0.641	0.645	0.65	0.654	0.659	0.664	0.669
Guam	0.693	0.688	0.684	0.684	0.688	0.695	0.704	0.715	0.728	0.741	0.753	0.762	0.767	0.768	0.77	0.771	0.773	0.775	0.778	0.782	0.785	0.789	0.792	0.795	0.797	0.799	0.803	0.807	0.81	0.813
Guatemala	0.315	0.32	0.328	0.336	0.345	0.352	0.36	0.369	0.378	0.387	0.395	0.403	0.412	0.421	0.43	0.439	0.446	0.453	0.459	0.465	0.472	0.478	0.485	0.491	0.498	0.504	0.51	0.516	0.522	0.526
Guinea	0.175	0.178	0.182	0.186	0.19	0.194	0.199	0.204	0.209	0.214	0.219	0.224	0.229	0.235	0.239	0.244	0.249	0.253	0.258	0.263	0.267	0.272	0.278	0.284	0.29	0.296	0.303	0.31	0.318	0.325
Guinea-Bissau	0.2	0.205	0.209	0.214	0.219	0.224	0.23	0.236	0.239	0.244	0.249	0.253	0.257	0.261	0.266	0.271	0.275	0.28	0.286	0.291	0.297	0.304	0.31	0.316	0.322	0.328	0.335	0.342	0.349	0.355
Guyana	0.452	0.456	0.461	0.467	0.474	0.481	0.488	0.496	0.503	0.51	0.516	0.522	0.527	0.532	0.536	0.54	0.544	0.549	0.554	0.559	0.565	0.571	0.577	0.583	0.59	0.596	0.602	0.608	0.614	0.618
Haiti	0.307	0.311	0.314	0.317	0.32	0.323	0.328	0.333	0.338	0.344	0.35	0.356	0.361	0.366	0.371	0.375	0.379	0.383	0.387	0.392	0.395	0.399	0.403	0.407	0.412	0.416	0.42	0.424	0.428	0.432
Honduras	0.33	0.335	0.34	0.345	0.351	0.357	0.363	0.37	0.377	0.384	0.391	0.397	0.404	0.411	0.417	0.424	0.43	0.437	0.443	0.448	0.454	0.459	0.464	0.469	0.473	0.478	0.482	0.487	0.492	0.496
Hungary	0.659	0.663	0.671	0.678	0.685	0.693	0.7	0.707	0.713	0.718	0.724	0.73	0.735	0.741	0.746	0.751	0.756	0.76	0.763	0.768	0.772	0.773	0.774	0.774	0.775	0.778	0.781	0.784	0.788	0.791
Iceland	0.764	0.77	0.774	0.778	0.782	0.785	0.788	0.79	0.794	0.799	0.806	0.813	0.818	0.822	0.824	0.827	0.83	0.834	0.838	0.842	0.846	0.847	0.847	0.848	0.85	0.854	0.858	0.863	0.866	0.869
India	0.327	0.333	0.339	0.345	0.351	0.358	0.364	0.371	0.378	0.386	0.393	0.4	0.407	0.414	0.421	0.429	0.437	0.446	0.455	0.463	0.473	0.483	0.493	0.504	0.515	0.526	0.537	0.547	0.558	0.566
Indonesia	0.452	0.462	0.472	0.481	0.49	0.499	0.508	0.516	0.522	0.527	0.533	0.537	0.542	0.547	0.552	0.558	0.564	0.571	0.578	0.585	0.593	0.601	0.609	0.617	0.625	0.633	0.64	0.647	0.654	0.66
Iran (Islamic Republic of)	0.404	0.419	0.433	0.446	0.457	0.468	0.477	0.486	0.496	0.506	0.517	0.529	0.541	0.552	0.562	0.571	0.581	0.593	0.603	0.613	0.622	0.63	0.635	0.64	0.645	0.649	0.654	0.659	0.665	0.67
Iraq	0.392	0.397	0.402	0.407	0.412	0.417	0.422	0.429	0.439	0.451	0.462	0.475	0.485	0.493	0.504	0.514	0.525	0.535	0.547	0.558	0.57	0.583	0.597	0.61	0.622	0.632	0.644	0.654	0.663	0.671
Ireland	0.73	0.735	0.741	0.747	0.753	0.758	0.763	0.768	0.774	0.78	0.786	0.793	0.799	0.806	0.812	0.816	0.819	0.821	0.824	0.827	0.831	0.835	0.839	0.842	0.845	0.85	0.854	0.859	0.864	0.867
Israel	0.717	0.721	0.726	0.73	0.734	0.738	0.742	0.745	0.749	0.752	0.756	0.76	0.762	0.765	0.769	0.773	0.776	0.778	0.778	0.779	0.781	0.784	0.787	0.79	0.792	0.794	0.796	0.798	0.8	0.803
Italy	0.712	0.717	0.722	0.727	0.732	0.737	0.74	0.744	0.747	0.75	0.753	0.757	0.761	0.763	0.766	0.768	0.771	0.773	0.775	0.777	0.78	0.782	0.784	0.787	0.789	0.791	0.794	0.796	0.798	0.801

Jamaica	0.542	0.547	0.553	0.56	0.566	0.573	0.58	0.586	0.592	0.598	0.603	0.609	0.614	0.62	0.625	0.63	0.635	0.64	0.645	0.649	0.653	0.657	0.661	0.664	0.668	0.671	0.675	0.678	0.681	0.684
Japan	0.791	0.796	0.801	0.805	0.809	0.813	0.817	0.82	0.822	0.824	0.826	0.828	0.83	0.833	0.836	0.838	0.84	0.842	0.844	0.846	0.848	0.85	0.853	0.855	0.857	0.86	0.862	0.865	0.867	0.87
Jordan	0.52	0.529	0.537	0.546	0.554	0.562	0.57	0.577	0.585	0.592	0.6	0.607	0.614	0.621	0.63	0.639	0.648	0.656	0.664	0.673	0.681	0.688	0.695	0.702	0.707	0.713	0.718	0.723	0.727	0.731
Kazakhstan	0.602	0.606	0.611	0.615	0.619	0.622	0.625	0.628	0.63	0.632	0.635	0.639	0.644	0.649	0.655	0.661	0.667	0.674	0.679	0.683	0.688	0.692	0.696	0.7	0.704	0.708	0.712	0.716	0.72	0.723
Kenya	0.333	0.341	0.348	0.354	0.36	0.366	0.372	0.378	0.383	0.388	0.392	0.397	0.401	0.404	0.409	0.413	0.418	0.424	0.429	0.435	0.441	0.448	0.455	0.463	0.47	0.478	0.486	0.494	0.502	0.508
Kiribati	0.425	0.427	0.429	0.432	0.435	0.438	0.441	0.444	0.447	0.451	0.455	0.459	0.463	0.468	0.472	0.476	0.48	0.484	0.487	0.49	0.492	0.495	0.497	0.501	0.504	0.509	0.514	0.518	0.523	0.527
Kuwait	0.655	0.659	0.662	0.667	0.673	0.68	0.689	0.699	0.709	0.717	0.724	0.729	0.735	0.742	0.75	0.76	0.769	0.777	0.785	0.793	0.801	0.808	0.815	0.822	0.828	0.834	0.839	0.844	0.848	0.851
Kyrgyzstan	0.532	0.537	0.541	0.543	0.542	0.541	0.539	0.537	0.536	0.534	0.534	0.535	0.537	0.54	0.544	0.546	0.549	0.552	0.555	0.558	0.56	0.563	0.565	0.569	0.574	0.578	0.583	0.588	0.592	0.596
Lao People's Democratic Republic	0.268	0.274	0.279	0.285	0.29	0.296	0.302	0.309	0.315	0.322	0.329	0.336	0.344	0.351	0.359	0.367	0.376	0.385	0.394	0.403	0.413	0.422	0.431	0.441	0.45	0.458	0.467	0.475	0.483	0.49
Latvia	0.675	0.682	0.691	0.7	0.708	0.713	0.716	0.719	0.721	0.723	0.727	0.733	0.739	0.745	0.753	0.76	0.766	0.774	0.784	0.793	0.797	0.798	0.801	0.803	0.804	0.805	0.809	0.813	0.817	0.82
Lebanon	0.462	0.47	0.477	0.485	0.493	0.502	0.511	0.52	0.53	0.54	0.548	0.557	0.565	0.574	0.582	0.591	0.6	0.609	0.618	0.628	0.639	0.649	0.66	0.67	0.677	0.685	0.691	0.698	0.704	0.708
Lesotho	0.321	0.327	0.333	0.34	0.346	0.353	0.36	0.367	0.373	0.38	0.387	0.393	0.399	0.405	0.411	0.417	0.423	0.429	0.435	0.441	0.448	0.455	0.462	0.469	0.476	0.483	0.489	0.496	0.502	0.507
Liberia	0.221	0.222	0.219	0.214	0.206	0.196	0.183	0.176	0.175	0.184	0.203	0.22	0.238	0.245	0.252	0.258	0.265	0.272	0.279	0.287	0.296	0.305	0.314	0.325	0.335	0.344	0.351	0.358	0.365	0.37
Libya	0.405	0.422	0.438	0.455	0.472	0.489	0.506	0.522	0.538	0.552	0.566	0.58	0.593	0.606	0.619	0.632	0.645	0.658	0.67	0.681	0.691	0.695	0.703	0.707	0.707	0.707	0.705	0.705	0.707	0.709
Lithuania	0.67	0.672	0.682	0.691	0.694	0.696	0.7	0.705	0.709	0.714	0.723	0.73	0.736	0.743	0.752	0.76	0.765	0.771	0.782	0.792	0.797	0.801	0.808	0.813	0.817	0.822	0.829	0.835	0.839	0.843
Luxembourg	0.815	0.818	0.82	0.823	0.828	0.833	0.836	0.839	0.842	0.844	0.847	0.85	0.853	0.855	0.857	0.858	0.862	0.866	0.869	0.871	0.872	0.874	0.877	0.88	0.883	0.886	0.889	0.892	0.894	0.895
Madagascar	0.265	0.268	0.27	0.273	0.275	0.277	0.28	0.283	0.286	0.29	0.294	0.299	0.302	0.307	0.311	0.316	0.32	0.324	0.328	0.332	0.336	0.342	0.348	0.355	0.361	0.369	0.376	0.383	0.391	0.396
Malawi	0.213	0.215	0.215	0.217	0.218	0.22	0.225	0.23	0.235	0.24	0.245	0.249	0.254	0.26	0.267	0.274	0.281	0.29	0.299	0.308	0.317	0.326	0.335	0.342	0.35	0.358	0.365	0.372	0.379	0.384
Malaysia	0.542	0.548	0.554	0.562	0.572	0.581	0.59	0.6	0.611	0.622	0.63	0.638	0.646	0.652	0.659	0.665	0.671	0.677	0.681	0.687	0.693	0.698	0.704	0.71	0.716	0.722	0.726	0.728	0.732	0.737
Maldives	0.303	0.314	0.324	0.336	0.347	0.359	0.37	0.382	0.394	0.406	0.417	0.427	0.437	0.447	0.456	0.464	0.473	0.481	0.49	0.497	0.504	0.511	0.518	0.525	0.532	0.538	0.544	0.551	0.557	0.562
Mali	0.126	0.129	0.132	0.136	0.139	0.143	0.147	0.151	0.155	0.159	0.163	0.168	0.173	0.178	0.183	0.188	0.193	0.198	0.203	0.209	0.214	0.22	0.225	0.23	0.235	0.241	0.247	0.253	0.259	0.263
Malta	0.666	0.67	0.675	0.682	0.69	0.695	0.696	0.7	0.708	0.715	0.722	0.729	0.733	0.737	0.741	0.745	0.749	0.753	0.757	0.761	0.764	0.768	0.772	0.775	0.779	0.784	0.788	0.793	0.797	0.801
Marshall Islands	0.398	0.404	0.41	0.416	0.423	0.43	0.435	0.439	0.442	0.446	0.45	0.454	0.458	0.463	0.467	0.472	0.477	0.482	0.487	0.493	0.498	0.504	0.509	0.515	0.52	0.525	0.531	0.536	0.541	0.544
Mauritania	0.308	0.314	0.319	0.326	0.332	0.338	0.344	0.349	0.355	0.36	0.365	0.369	0.374	0.379	0.384	0.39	0.398	0.406	0.413	0.42	0.427	0.435	0.443	0.45	0.459	0.467	0.474	0.482	0.49	0.496
Mauritius	0.527	0.532	0.535	0.543	0.556	0.565	0.57	0.576	0.583	0.588	0.593	0.598	0.603	0.608	0.614	0.621	0.627	0.633	0.64	0.646	0.652	0.658	0.665	0.673	0.68	0.686	0.69	0.695	0.7	0.705
Mexico	0.507	0.514	0.52	0.526	0.532	0.537	0.542	0.547	0.553	0.558	0.563	0.569	0.574	0.578	0.583	0.588	0.592	0.597	0.601	0.605	0.608	0.613	0.617	0.621	0.626	0.631	0.636	0.64	0.645	0.649
Micronesia (Federated States of)	0.447	0.453	0.459	0.465	0.471	0.478	0.483	0.488	0.492	0.497	0.502	0.507	0.511	0.516	0.521	0.525	0.53	0.534	0.538	0.542	0.546	0.55	0.554	0.558	0.561	0.565	0.569	0.573	0.577	0.58
Monaco	0.834	0.837	0.84	0.843	0.846	0.849	0.852	0.855	0.857	0.86	0.862	0.865	0.867	0.87	0.872	0.875	0.877	0.879	0.881	0.883	0.886	0.888	0.89	0.892	0.893	0.895	0.897	0.899	0.901	0.902
Mongolia	0.465	0.47	0.475	0.48	0.484	0.49	0.495	0.501	0.506	0.512	0.517	0.523	0.528	0.534	0.539	0.545	0.55	0.555	0.56	0.563	0.566	0.57	0.575	0.579	0.584	0.588	0.592	0.597	0.601	0.606
Montenegro	0.701	0.701	0.699	0.695	0.69	0.687	0.686	0.687	0.69	0.692	0.696	0.701	0.706	0.712	0.717	0.723	0.729	0.736	0.743	0.749	0.754	0.759	0.764	0.768	0.773	0.777	0.78	0.784	0.788	0.791
Morocco	0.347	0.354	0.361	0.367	0.374	0.38	0.386	0.392	0.398	0.403	0.409	0.414	0.42	0.426	0.432	0.439	0.445	0.452	0.46	0.467	0.475	0.483	0.491	0.499	0.508	0.516	0.524	0.533	0.541	0.548
Mozambique	0.12	0.122	0.123	0.126	0.129	0.131	0.137	0.144	0.152	0.159	0.165	0.172	0.179	0.186	0.194	0.201	0.208	0.215	0.223	0.23	0.237	0.244	0.252	0.26	0.268	0.277	0.285	0.294	0.301	0.307
Myanmar	0.284	0.287	0.29	0.295	0.3	0.306	0.313	0.32	0.327	0.335	0.344	0.353	0.363	0.373	0.384	0.395	0.406	0.417	0.427	0.437	0.446	0.455	0.464	0.473	0.482	0.49	0.498	0.506	0.514	0.521
Namibia	0.454	0.459	0.465	0.47	0.475	0.48	0.486	0.491	0.495	0.5	0.505	0.509	0.514	0.518	0.523	0.529	0.534	0.54	0.546	0.552	0.558	0.564	0.571	0.577	0.584	0.591	0.597	0.603	0.608	0.612
Nauru	0.499	0.501	0.503	0.504	0.504	0.505	0.505	0.504	0.504	0.503	0.503	0.503	0.503	0.503	0.504	0.506	0.509	0.51	0.515	0.521	0.529	0.538	0.547	0.559	0.573	0.585	0.595	0.605	0.613	0.618
Nepal	0.198	0.203	0.208	0.215	0.221	0.228	0.236	0.244	0.251	0.259	0.267	0.276	0.284	0.291	0.299	0.307	0.315	0.322	0.33	0.339	0.347	0.356	0.365	0.373	0.382	0.391	0.399	0.408	0.416	0.422
Netherlands	0.796	0.801	0.806	0.81	0.814	0.818	0.821	0.824	0.827	0.83	0.832	0.836	0.839	0.842	0.845	0.848	0.851	0.853	0.856	0.858	0.861	0.864	0.866	0.869	0.871	0.874	0.876	0.878	0.881	0.883
New Zealand	0.757	0.762	0.765	0.769	0.772	0.774	0.778	0.782	0.785	0.787	0.79	0.794	0.796	0.798	0.802	0.803	0.8	0.8	0.803	0.807	0.809	0.812	0.816	0.821	0.825	0.828	0.832	0.835	0.838	0.84
Nicaragua	0.338	0.345	0.353	0.36	0.368	0.376	0.385	0.393	0.402	0.411	0.419	0.426	0.433	0.438	0.444	0.449	0.453	0.458	0.462	0.466	0.47	0.474	0.479	0.484	0.489	0.495	0.5	0.506	0.512	0.517
Niger	0.0728	0.0746	0.0761	0.0777	0.0793	0.0808	0.0822	0.0836	0.0853	0.0871	0.0887	0.0907	0.093	0.0956	0.0982	0.101	0.104	0.108	0.111	0.115	0.119	0.123	0.128	0.133	0.138	0.143	0.148	0.153	0.158	0.162
Nigeria	0.305	0.308	0.312	0.315	0.319	0.324	0.329	0.334	0.339	0.344	0.35	0.356	0.363	0.371	0.381	0.392	0.402	0.412	0.422	0.432	0.442	0.451	0.46	0.469	0.478	0.487	0.495	0.503	0.51	0.515
Niue	0.566	0.571	0.576	0.581	0.586	0.591	0.595	0.6	0.605	0.609	0.614	0.618	0.622	0.626	0.631	0.638	0.645	0.651	0.657	0.663	0.669	0.675	0.681	0.685	0.69	0.695	0.699	0.703	0.707	0.711
North Macedonia	0.618	0.62	0.623	0.625	0.627	0.631	0.635	0.64	0.646	0.651	0.656	0.662	0.668	0.674	0.679	0.684	0.689	0.694	0.7	0.704	0.709	0.713	0.717	0.722	0.726	0.73	0.734	0.738	0.741	0.744
Northern Mariana Islands	0.692	0.698	0.704	0.709	0.714	0.718	0.722	0.725	0.727	0.731	0.738	0.744	0.748	0.75	0.751	0.752	0.752	0.753	0.753	0.752	0.751	0.75	0.75	0.75	0.75	0.751	0.756	0.761	0.767	0.771
Norway	0.807	0.812	0.818	0.823	0.828	0.832	0.837	0.843	0.847	0.851	0.856	0.861	0.866	0.869	0.872	0.874	0.876	0.877	0.879	0.881	0.885	0.889	0.893	0.896	0.9	0.903	0.907	0.91	0.912	0.913
Oman	0.441	0.455	0.469	0.485	0.5	0.514	0.528	0.543	0.558	0.573	0.588	0.601	0.615	0.628	0.641	0.653	0.664	0.678	0.692	0.704	0.715	0.726	0.737	0.747	0.754	0.76	0.767	0.773	0.778	0.783
Pakistan	0.247	0.253	0.259	0.265	0.271	0.277	0.283	0.289	0.295	0.301	0.307	0.313	0.32	0.326	0.333	0.34	0.347	0.355	0.363	0.371	0.379	0.387	0.394	0.402	0.41	0.418	0.426	0.434	0.442	0.449
Palau	0.621	0.629	0.636	0.642	0.647	0.652	0.658	0.663	0.668	0.672	0.676	0.68	0.683	0.686	0.69	0.693	0.697	0.7	0.703	0.705	0.707	0.71	0.714	0.717	0.72	0.725	0.729	0.732	0.735	0.738
Palestine	0.314	0.32	0.329	0.337	0.347	0.356	0.365	0.375	0.386	0.397	0.407	0.415	0.422	0.43	0.439	0.449	0.458	0.467	0.476	0.486	0.497	0.509	0.521	0.533	0.543	0.553	0.564	0.573	0.582	0.588
Panama	0.544	0.549	0.555	0.559	0.562	0.565	0.568	0.573	0.579	0.586	0.592	0.597	0.602	0.605	0.607	0.61	0.613	0.616	0.62	0.623	0.627	0.63	0.635	0.642	0.65	0.658	0.666	0.674	0.68	0.686
Papua New Guinea	0.292	0.295	0.299	0.304	0.309	0.313	0.317	0.32	0.323	0.326	0.329	0.331	0.333	0.335	0.337	0.339	0.342	0.345	0.348	0.352	0.356	0.36	0.363	0.367	0.372	0.377	0.382	0.386	0.391	0.394
Paraguay	0.465	0.471	0.477	0.484	0.491	0.498	0.505	0.512	0.519	0.524	0.529	0.534	0.539	0.544	0.549	0.554	0.559	0.564	0.57	0.575	0.582	0.588	0.594	0.601	0.608	0.615	0.621	0.627	0.633	0.638
Peru	0.501	0.505	0.508	0.511	0.516	0.521	0.526	0.532	0.537	0.543	0.548	0.552	0.557	0.562	0.566	0.571	0.577	0.583	0.589	0.595	0.601	0.608	0.614	0.62	0.625	0.63	0.635	0.64	0.645	0.648
Philippines	0.497	0.501	0.505	0.509	0.513	0.518	0.522	0.526	0.529	0.532	0.534	0.537	0.54	0.542	0.545	0.547	0.55	0.554	0.558	0.562	0.567	0.572	0.577	0.583	0.589	0.596	0.603	0.61	0.617	0.623
Poland	0.632	0.637	0.644	0.653	0.661	0.67	0.677	0.685	0.693	0.701	0.709	0.717	0.724	0.73	0.735	0.74	0.743	0.747	0.752	0.757	0.763	0.77	0.775	0.78	0.784	0.788	0.791	0.795	0.798	0.802
Portugal	0.607	0.615	0.622	0.629	0.636	0.641	0.647	0.651	0.656	0.661	0.666	0.671	0.676	0.681	0.686	0.69	0.694	0.698	0.701	0.705	0.709	0.714	0.718	0.722	0.726	0.729	0.732	0.736	0.739	0.743
Puerto Rico	0.67	0.676	0.679	0.684	0.689	0.693	0.699	0.705	0.711	0.714	0.72	0.731	0.738	0.742	0.744	0.748	0.752	0.756	0.76	0.764	0.769	0.774	0.779	0.785	0.793	0.802	0.808	0.811	0.813	0.814
Qatar	0.585	0.598	0.61	0.621	0.632	0.643	0.654	0.666	0.676	0.685	0.694	0.703	0.711	0.719	0.727	0.735	0.743	0.75	0.757	0.764	0.772	0.779	0.786	0.793	0.799	0.806	0.812	0.818	0.825	0.83
Republic of Korea	0.686	0.697	0.708	0.719	0.73	0.74	0.75	0.76	0.767	0.776	0.784	0.791	0.799	0.805	0.811	0.817	0.823	0.828	0.833	0.837	0.842	0.846	0.851	0.855	0.859	0.863	0.867	0.871	0.875	0.878
Republic of Moldova	0.585	0.589	0.591	0.594	0.594	0.595	0.594	0.593	0.591	0.587	0.585	0.585	0.588	0.593	0.6	0.607	0.615	0.622	0.63	0.637	0.644	0.651	0.658	0.665	0.672	0.677	0.683	0.688	0.693	0.696
Romania	0.625	0.632	0.635	0.638	0.643	0.649	0.653	0.655	0.659	0.664	0.669	0.677	0.682	0.686	0.693	0.698	0.702	0.707	0.711	0.718	0.726	0.729	0.734	0.74	0.741	0.744	0.747	0.752	0.756	0.76
Russian Federation	0.695	0.703	0.716	0.72	0.719	0.722	0.724	0.725	0.726	0.728	0.728	0.728	0.73	0.734	0.741	0.749	0.754	0.759	0.764	0.77	0.775	0.777	0.779	0.784	0.788	0.793	0.797	0.801	0.803	0.805
Rwanda	0.257	0.259	0.262	0.265	0.261	0.261	0.262	0.265	0.269	0.272	0.277	0.283	0.291	0.298	0.307	0.315	0.325	0.333	0.342	0.351	0.359	0.368	0.376	0.384	0.391	0.399	0.407	0.415	0.422	0.429
Saint Kitts and Nevis	0.583	0.592	0.599	0.607	0.614	0.62	0.626	0.633	0.638	0.644	0.65	0.657	0.663	0.668	0.673	0.679	0.685	0.69	0.696	0.701	0.706	0.71	0.714	0.719	0.724	0.728	0.733	0.738	0.742	0.746
Saint Lucia	0.483	0.493	0.504	0.514	0.524	0.534	0.543	0.551	0.56	0.568	0.575	0.581	0.587	0.593	0.6	0.606	0.612	0.617	0.623	0.629	0.634	0.639	0.643	0.648	0.652	0.656	0.659	0.663	0.667	0.67
Saint Vincent and the Grenadines	0.462	0.471	0.479	0.487	0.495	0.502	0.509	0.515	0.522	0.528	0.534	0.539	0.545	0.551	0.557	0.562	0.568	0.574	0.58	0.585	0.589	0.593	0.598	0.602	0.606	0.61	0.615	0.619	0.623	0.627
Samoa	0.531	0.535	0.539	0.543	0.546	0.55	0.555	0.559	0.563	0.567	0.571	0.576	0.581	0.586	0.59	0.595	0.599	0.603	0.607	0.61	0.612	0.615	0.618	0.62	0.623	0.626	0.629	0.633	0.637	0.641
San Marino	0.814	0.817	0.82	0.824	0.828	0.832	0.837	0.841	0.846	0.85	0.852	0.854	0.856	0.859	0.861	0.862	0.864	0.866	0.868	0.87	0.872	0.874	0.876	0.877	0.879	0.881	0.882	0.882	0.883	0.884
Sao Tome and Principe	0.299	0.302	0.306	0.309	0.313	0.317	0.322	0.327	0.332	0.338	0.344	0.351	0.358	0.365	0.373	0.381	0.39	0.398	0.407	0.416	0.424	0.433	0.443	0.452	0.461	0.47	0.478	0.487	0.495	0.502
Saudi Arabia	0.48	0.491	0.504	0.516	0.529	0.541	0.554	0.566	0.578	0.59	0.602	0.614	0.625	0.637	0.65	0.664	0.677	0.69	0.703	0.715	0.726	0.738	0.75	0.76	0.769	0.778	0.786	0.793	0.8	0.805
Senegal	0.227	0.233	0.239	0.245	0.251	0.257	0.262	0.267	0.272	0.277	0.282	0.286	0.29	0.295	0.299	0.304	0.308	0.313	0.318	0.324	0.33	0.336	0.342	0.348	0.354	0.361	0.368	0.375	0.382	0.389
Serbia	0.626	0.635	0.639	0.639	0.64	0.64	0.644	0.647	0.651	0.657	0.661	0.665	0.67	0.676	0.685	0.694	0.702	0.709	0.716	0.723	0.729	0.735	0.739	0.744	0.748	0.753	0.756	0.76	0.763	0.767
Seychelles	0.567	0.576	0.584	0.592	0.6	0.607	0.614	0.621	0.629	0.636	0.642	0.647	0.652	0.656	0.659	0.662	0.666	0.67	0.673	0.676	0.679	0.683	0.687	0.691	0.696	0.702	0.707	0.713	0.719	0.724
Sierra Leone	0.207	0.209	0.21	0.212	0.215	0.218	0.218	0.218	0.219	0.218	0.219	0.221	0.224	0.229	0.234	0.239	0.245	0.252	0.26	0.267	0.275	0.283	0.292	0.304	0.314	0.321	0.328	0.335	0.342	0.347
Singapore	0.688	0.697	0.705	0.714	0.723	0.731	0.74	0.749	0.756	0.762	0.769	0.776	0.783	0.789	0.794	0.801	0.808	0.814	0.822	0.828	0.835	0.839	0.843	0.847	0.85	0.852	0.855	0.858	0.86	0.861
Slovakia	0.656	0.662	0.668	0.679	0.693	0.702	0.709	0.716	0.724	0.731	0.739	0.746	0.752	0.756	0.76	0.766	0.772	0.777	0.781	0.784	0.789	0.794	0.798	0.801	0.803	0.803	0.804	0.805	0.808	0.812
Slovenia	0.726	0.731	0.736	0.741	0.746	0.751	0.756	0.762	0.768	0.774	0.78	0.787	0.793	0.797	0.802	0.807	0.811	0.814	0.818	0.82	0.822	0.824	0.825	0.827	0.829	0.831	0.833	0.835	0.838	0.84
Solomon Islands	0.279	0.283	0.288	0.294	0.3	0.306	0.312	0.317	0.321	0.326	0.328	0.33	0.331	0.332	0.333	0.336	0.339	0.343	0.348	0.353	0.358	0.364	0.371	0.377	0.382	0.387	0.393	0.398	0.403	0.407
Somalia	0.0508	0.0514	0.0522	0.053	0.0539	0.0547	0.0556	0.0565	0.0574	0.0584	0.0593	0.0603	0.0613	0.0622	0.0632	0.0641	0.0651	0.0661	0.0671	0.0682	0.0692	0.0703	0.0716	0.0728	0.0742	0.0756	0.077	0.0785	0.0799	0.081
South Africa	0.552	0.556	0.56	0.564	0.567	0.572	0.576	0.58	0.584	0.588	0.593	0.597	0.602	0.607	0.611	0.616	0.622	0.627	0.632	0.637	0.642	0.647	0.651	0.656	0.66	0.664	0.668	0.672	0.676	0.678
South Sudan	0.248	0.251	0.253	0.256	0.259	0.261	0.264	0.267	0.271	0.274	0.277	0.281	0.284	0.288	0.292	0.296	0.301	0.305	0.31	0.315	0.32	0.325	0.33	0.334	0.339	0.342	0.345	0.351	0.358	0.363
Spain	0.647	0.655	0.662	0.669	0.675	0.681	0.686	0.691	0.696	0.7	0.705	0.709	0.713	0.717	0.721	0.725	0.728	0.73	0.734	0.739	0.743	0.746	0.749	0.752	0.754	0.756	0.759	0.761	0.764	0.767
Sri Lanka	0.504	0.511	0.518	0.525	0.532	0.539	0.547	0.554	0.561	0.567	0.573	0.578	0.582	0.587	0.592	0.597	0.602	0.609	0.615	0.621	0.628	0.636	0.644	0.651	0.658	0.666	0.672	0.678	0.684	0.69
Sudan	0.227	0.234	0.241	0.248	0.256	0.263	0.271	0.28	0.289	0.298	0.308	0.318	0.327	0.337	0.348	0.358	0.37	0.381	0.393	0.404	0.416	0.428	0.44	0.451	0.462	0.474	0.485	0.497	0.507	0.515
Suriname	0.498	0.503	0.507	0.511	0.513	0.515	0.518	0.523	0.528	0.533	0.538	0.545	0.552	0.56	0.568	0.574	0.579	0.584	0.588	0.593	0.598	0.602	0.607	0.611	0.616	0.62	0.625	0.629	0.633	0.636
Sweden	0.769	0.775	0.782	0.788	0.794	0.801	0.806	0.811	0.815	0.819	0.823	0.826	0.829	0.832	0.835	0.838	0.84	0.842	0.844	0.846	0.849	0.852	0.855	0.858	0.86	0.863	0.865	0.868	0.87	0.872
Switzerland	0.868	0.869	0.873	0.876	0.878	0.88	0.881	0.883	0.884	0.886	0.889	0.893	0.894	0.896	0.898	0.9	0.902	0.905	0.908	0.909	0.912	0.914	0.917	0.919	0.921	0.922	0.924	0.926	0.928	0.929
Syrian Arab Republic	0.367	0.376	0.387	0.398	0.409	0.421	0.432	0.443	0.454	0.465	0.476	0.486	0.497	0.508	0.521	0.535	0.548	0.56	0.572	0.583	0.594	0.601	0.605	0.606	0.607	0.608	0.61	0.613	0.616	0.619
Taiwan (province of China)	0.667	0.678	0.685	0.694	0.702	0.711	0.719	0.731	0.743	0.747	0.754	0.763	0.772	0.779	0.787	0.795	0.802	0.81	0.817	0.824	0.83	0.833	0.838	0.843	0.848	0.852	0.856	0.86	0.865	0.868
Tajikistan	0.468	0.473	0.474	0.474	0.472	0.468	0.462	0.457	0.451	0.445	0.441	0.44	0.443	0.448	0.456	0.463	0.47	0.477	0.483	0.489	0.495	0.5	0.505	0.511	0.516	0.521	0.526	0.531	0.535	0.539
Thailand	0.508	0.518	0.527	0.536	0.544	0.553	0.562	0.569	0.574	0.578	0.583	0.589	0.594	0.599	0.605	0.61	0.616	0.623	0.629	0.633	0.638	0.643	0.649	0.655	0.66	0.666	0.671	0.676	0.682	0.687
Timor-Leste	0.274	0.28	0.286	0.293	0.3	0.308	0.317	0.326	0.334	0.339	0.345	0.352	0.358	0.364	0.374	0.388	0.406	0.421	0.436	0.448	0.458	0.469	0.48	0.488	0.493	0.498	0.503	0.508	0.511	0.514

Togo	0.266	0.272	0.278	0.281	0.286	0.291	0.296	0.302	0.306	0.31	0.313	0.317	0.32	0.323	0.327	0.33	0.334	0.338	0.342	0.347	0.352	0.358	0.364	0.371	0.379	0.386	0.394	0.402	0.411	0.417
Tokelau	0.427	0.432	0.438	0.444	0.45	0.456	0.463	0.47	0.477	0.484	0.491	0.498	0.504	0.511	0.519	0.526	0.534	0.542	0.55	0.557	0.565	0.573	0.58	0.588	0.595	0.602	0.608	0.615	0.621	0.626
Tonga	0.51	0.517	0.522	0.527	0.533	0.538	0.543	0.547	0.551	0.555	0.559	0.563	0.568	0.572	0.576	0.58	0.583	0.587	0.59	0.594	0.598	0.602	0.606	0.61	0.614	0.618	0.622	0.627	0.632	0.636
Trinidad and Tobago	0.618	0.622	0.626	0.63	0.634	0.639	0.644	0.65	0.656	0.662	0.669	0.675	0.682	0.689	0.696	0.704	0.711	0.718	0.724	0.728	0.732	0.736	0.739	0.742	0.745	0.748	0.751	0.753	0.755	0.757
Tunisia	0.434	0.444	0.455	0.466	0.476	0.487	0.498	0.508	0.518	0.528	0.538	0.548	0.556	0.565	0.574	0.582	0.591	0.599	0.607	0.614	0.622	0.628	0.634	0.64	0.646	0.651	0.657	0.662	0.667	0.672
Turkey	0.473	0.483	0.493	0.504	0.513	0.523	0.534	0.545	0.556	0.566	0.577	0.587	0.597	0.607	0.619	0.63	0.641	0.652	0.663	0.671	0.68	0.689	0.698	0.707	0.715	0.723	0.729	0.736	0.743	0.748
Turkmenistan	0.548	0.551	0.554	0.557	0.557	0.558	0.558	0.557	0.556	0.557	0.561	0.565	0.57	0.576	0.582	0.588	0.595	0.601	0.606	0.611	0.616	0.622	0.628	0.635	0.642	0.648	0.654	0.66	0.666	0.67
Tuvalu	0.426	0.434	0.442	0.45	0.458	0.465	0.47	0.476	0.483	0.49	0.496	0.502	0.509	0.514	0.519	0.523	0.527	0.531	0.537	0.541	0.545	0.549	0.553	0.558	0.562	0.567	0.573	0.579	0.584	0.589
Uganda	0.167	0.169	0.172	0.176	0.181	0.187	0.194	0.201	0.208	0.216	0.225	0.234	0.243	0.253	0.262	0.272	0.282	0.293	0.303	0.314	0.325	0.335	0.345	0.355	0.364	0.373	0.382	0.391	0.399	0.404
Ukraine	0.653	0.657	0.661	0.665	0.666	0.667	0.667	0.667	0.666	0.665	0.664	0.665	0.668	0.672	0.679	0.685	0.692	0.699	0.706	0.71	0.713	0.718	0.721	0.725	0.727	0.729	0.73	0.732	0.734	0.736
United Arab Emirates	0.621	0.637	0.653	0.669	0.685	0.7	0.714	0.727	0.74	0.751	0.762	0.773	0.783	0.792	0.801	0.81	0.819	0.829	0.838	0.846	0.853	0.859	0.863	0.868	0.87	0.872	0.874	0.876	0.879	0.88
United Kingdom	0.745	0.749	0.755	0.761	0.766	0.769	0.771	0.774	0.778	0.784	0.789	0.793	0.797	0.799	0.802	0.804	0.806	0.808	0.811	0.813	0.816	0.82	0.825	0.83	0.834	0.837	0.839	0.842	0.845	0.847
United Republic of Tanzania	0.26	0.265	0.269	0.273	0.276	0.28	0.284	0.288	0.292	0.296	0.301	0.305	0.31	0.315	0.32	0.326	0.331	0.337	0.342	0.348	0.354	0.361	0.368	0.375	0.383	0.391	0.399	0.408	0.416	0.423
United States of America	0.768	0.771	0.775	0.778	0.782	0.785	0.788	0.789	0.791	0.794	0.797	0.802	0.806	0.809	0.811	0.812	0.811	0.814	0.819	0.826	0.832	0.835	0.839	0.842	0.845	0.849	0.853	0.856	0.858	0.859
United States Virgin Islands	0.667	0.68	0.689	0.697	0.704	0.71	0.715	0.72	0.724	0.728	0.731	0.734	0.742	0.749	0.756	0.762	0.768	0.773	0.778	0.782	0.785	0.788	0.79	0.791	0.792	0.794	0.795	0.796	0.798	0.799
Uruguay	0.581	0.584	0.588	0.591	0.594	0.597	0.6	0.606	0.612	0.618	0.622	0.626	0.628	0.631	0.633	0.636	0.639	0.642	0.645	0.649	0.653	0.658	0.663	0.668	0.673	0.678	0.684	0.688	0.693	0.697
Uzbekistan	0.49	0.492	0.494	0.496	0.498	0.501	0.505	0.51	0.515	0.52	0.525	0.531	0.536	0.541	0.546	0.551	0.556	0.561	0.567	0.572	0.578	0.584	0.59	0.597	0.603	0.609	0.616	0.622	0.627	0.631
Vanuatu	0.361	0.365	0.369	0.373	0.377	0.381	0.386	0.39	0.395	0.399	0.403	0.407	0.41	0.413	0.417	0.42	0.425	0.43	0.435	0.44	0.446	0.451	0.455	0.46	0.464	0.468	0.473	0.477	0.481	0.485
Venezuela (Bolivarian Republic of)	0.509	0.514	0.522	0.53	0.537	0.542	0.546	0.551	0.554	0.557	0.559	0.56	0.558	0.552	0.548	0.55	0.557	0.566	0.575	0.582	0.586	0.591	0.596	0.6	0.604	0.607	0.608	0.608	0.608	0.607
Viet Nam	0.39	0.397	0.404	0.412	0.42	0.429	0.438	0.447	0.455	0.463	0.471	0.478	0.486	0.493	0.501	0.509	0.517	0.525	0.533	0.541	0.549	0.558	0.566	0.573	0.581	0.589	0.596	0.604	0.611	0.617
Yemen	0.176	0.183	0.191	0.198	0.207	0.215	0.224	0.234	0.243	0.253	0.263	0.273	0.283	0.293	0.303	0.314	0.325	0.335	0.346	0.356	0.366	0.375	0.384	0.393	0.402	0.407	0.41	0.412	0.413	0.412
Zambia	0.299	0.304	0.307	0.311	0.314	0.316	0.319	0.323	0.326	0.33	0.334	0.34	0.345	0.352	0.359	0.367	0.376	0.386	0.396	0.407	0.418	0.429	0.44	0.451	0.462	0.472	0.481	0.491	0.499	0.505
Zimbabwe	0.394	0.403	0.409	0.415	0.421	0.426	0.432	0.437	0.441	0.444	0.446	0.448	0.448	0.447	0.444	0.441	0.437	0.433	0.428	0.425	0.425	0.427	0.432	0.438	0.445	0.452	0.459	0.465	0.471	0.476
