# Supplementary material for: The global burden, trends and cross-region inequities of non-communicable diseases attributed to ambient particulate matter pollution
Source: Front Public Health. 2025 Nov 3;13:1682574. doi: 10.3389/fpubh.2025.1682574 (PMC12620374; doi:10.3389/fpubh.2025.1682574)
Supplement: Supplementary file 2 [file Supplementary_file_1.docx]

| **Table S1 The DALYs of non-communicable diseases attributed to ambient particulate matter pollution in 2019, and their temporal trends from 1990 to 2019** | | | | | |
| --- | --- | --- | --- | --- | --- |
| Location | TBL cancer | IHD | Stroke | COPD | Diabetes mellitus |
| Global | | | | | |
| DALYs in 2019 (million) | 7.016 (5.139, 9.026) | 32.174 (24.232, 40.676) | 28.736 (22.057, 35.397) | 15.414 (12.275, 19.078) | 9.034 (6.069, 12.489) |
| ASDR in 2019 (per 100,000) | 152.330 (111.577, 195.966) | 703.886 (529.821, 890.632) | 629.549 (483.053, 775.871) | 345.133 (274.751, 427.404) | 197.126 (132.439, 272.324) |
| AAPC on ASDR, 1990-2019 | 0.484 (0.255, 0.713) | 0.229 (-0.071, 0.530)* | 0.304 (0.063, 0.546) | -0.342 (-0.457, -0.227) | 2.186 (2.049, 2.324) |
| Male | | | | | |
| DALYs in 2019 (million) | 4.996 (6.499, 3.589) | 21.098 (26.722, 15.835) | 16.821 (20.780, 12.799) | 9.013 (11.207, 7.073) | 4.821 (6.649, 3.253) |
| ASDR in 2019 (per 100,000) | 228.743 (164.421, 297.435) | 960.806 (720.570, 1218.487) | 774.382 (589.281, 957.186) | 441.733 (346.565, 549.318) | 221.043 (149.287, 304.353) |
| AAPC on ASDR, 1990-2019 | 0.109 (-0.158, 0.376)* | 0.272 (0.008, 0.537) | 0.465 (0.227, 0.703) | -0.561 (-0.667, -0.455) | 2.340 (2.233, 2.446) |
| Female | | | | | |
| DALYs in 2019 (million) | 2.020 (2.627, 1.449) | 11.076 (14.255, 8.177) | 11.916 (15.071, 8.920) | 6.401 (8.168, 4.828) | 4.213 (5.854, 2.813) |
| ASDR in 2019 (per 100,000) | 83.812 (60.154, 109.015) | 461.550 (340.836, 593.981) | 496.228 (371.530, 627.596) | 264.874 (199.818, 337.989) | 175.253 (116.901, 243.665) |
| AAPC on ASDR, 1990-2019 | 1.440 (1.292, 1.587) | 0.220 (-0.03, 0.471)* | 0.085 (-0.146, 0.317)* | -0.097 (-0.276, 0.082)* | 2.011 (1.88, 2.143) |
| Low SDI | | | | | |
| DALYs in 2019 (million) | 0.127 (0.073, 0.198) | 1.754 (1.051, 2.604) | 1.333 (0.776, 2.024) | 1.303 (0.909, 1.753) | 0.412 (0.219, 0.662) |
| ASDR in 2019 (per 100,000) | 42.034 (24.125, 65.079) | 551.178 (328.294, 818.353) | 431.581 (252.219, 654.490) | 506.734 (354.104, 681.752) | 138.061 (73.393, 220.871) |
| AAPC on ASDR, 1990-2019 | 3.239 (2.938, 3.540) | 2.938 (2.443, 3.436) | 2.265 (1.823, 2.709) | 1.928 (1.586, 2.271) | 4.005 (3.749, 4.261) |
| Low-middle SDI | | | | | |
| DALYs in 2019 (million) | 0.651 (0.426, 0.881) | 7.298 (4.982, 9.686) | 5.731 (3.903, 7.563) | 5.401 (4.025, 6.888) | 1.753 (1.098, 2.508) |
| ASDR in 2019 (per 100,000) | 82.615 (54.040, 111.737) | 906.780 (619.334, 1203.559) | 730.471 (497.458, 964.736) | 763.390 (569.022, 972.574) | 226.839 (142.528, 323.484) |
| AAPC on ASDR, 1990-2019 | 3.393 (3.228, 3.557) | 3.403 (2.935, 3.874) | 2.602 (2.303, 2.902) | 1.706 (1.187, 2.227) | 4.841 (4.638, 5.045) |
| Middle SDI | | | | | |
| DALYs in 2019 (million) | 2.946 (2.110, 3.852) | 13.694 (10.555, 17.011) | 13.074 (10.095, 16.076) | 5.599 (4.445, 6.971) | 3.849 (2.635, 5.218) |
| ASDR in 2019 (per 100,000) | 207.303 (148.482, 270.872) | 974.148 (749.273, 1212.005) | 940.683 (724.997, 1158.518) | 443.725 (351.857, 553.413) | 271.579 (186.156, 367.105) |
| AAPC on ASDR, 1990-2019 | 2.594 (2.334, 2.854) | 1.297 (1.080, 1.514) | 0.930 (0.735, 1.126) | -1.174 (-1.339, -1.009) | 2.901 (2.793, 3.010) |
| High-middle SDI | | | | | |
| DALYs in 2019 (million) | 2.449 (1.796, 3.158) | 7.561 (5.513, 9.884) | 7.215 (5.551, 8.966) | 2.460 (1.875, 3.174) | 2.021 (1.359, 2.798) |
| ASDR in 2019 (per 100,000) | 215.354 (157.972, 277.726) | 682.914 (498.481, 892.876) | 648.440 (498.731, 805.968) | 221.115 (168.379, 285.355) | 181.168 (121.571, 251.279) |
| AAPC on ASDR, 1990-2019 | 0.144 (-0.159, 0.448)* | -1.088 (-1.503, -0.672) | -0.914 (-1.333, -0.494) | -2.150 (-2.313, -1.986) | 1.359 (1.186, 1.532) |
| High SDI | | | | | |
| DALYs in 2019 (million) | 0.840 (0.546, 1.212) | 1.854 (1.175, 2.748) | 1.373 (0.959, 1.913) | 0.648 (0.400, 0.961) | 0.993 (0.570, 1.553) |
| ASDR in 2019 (per 100,000) | 85.781 (55.927, 123.437) | 204.771 (131.906, 299.991) | 151.832 (107.336, 209.232) | 62.255 (38.615, 92.146) | 106.045 (60.889, 166.017) |
| AAPC on ASDR, 1990-2019 | -2.425 (-2.537, -2.314) | -3.682 (-3.852, -3.511) | -2.815 (-2.902, -2.728) | -2.164 (-2.287, -2.042) | -0.306 (-0.427, -0.186) |
| Central Europe, Eastern Europe, and Central Asia | | | | | |
| DALYs in 2019 (million) | 0.491 (0.341, 0.658) | 3.144 (2.018, 4.498) | 1.878 (1.301, 2.580) | 0.297 (0.199, 0.411) | 0.503 (0.315, 0.723) |
| ASDR in 2019 (per 100,000) | 142.549 (99.112, 191.285) | 934.247 (600.308, 1337.191) | 559.316 (388.086, 768.150) | 86.921 (58.406, 120.640) | 148.280 (92.769, 213.998) |
| AAPC on ASDR, 1990-2019 | -1.786 (-2.215, -1.354) | -1.430 (-1.994, -0.863) | -1.893 (-2.407, -1.377) | -2.785 (-3.216, -2.352) | 1.374 (1.245, 1.503) |
| High-income | | | | | |
| DALYs in 2019 (million) | 0.857 (0.535, 1.263) | 1.461 (0.811, 2.355) | 1.076 (0.680, 1.616) | 0.647 (0.383, 0.978) | 1.002 (0.557, 1.604) |
| ASDR in 2019 (per 100,000) | 78.829 (49.261, 116.130) | 137.763 (76.621, 222.088) | 101.885 (64.564, 152.526) | 53.838 (31.803, 81.613) | 94.187 (51.958, 151.616) |
| AAPC on ASDR, 1990-2019 | -2.707 (-2.875, -2.538) | -4.735 (-4.862, -4.608) | -3.879 (-4.026, -3.732) | -2.464 (-2.575, -2.353) | -0.746 (-0.840, -0.653) |
| Latin America and Caribbean | | | | | |
| DALYs in 2019 (million) | 0.197 (0.137, 0.266) | 1.193 (0.794, 1.683) | 0.783 (0.554, 1.058) | 0.315 (0.216, 0.428) | 0.968 (0.626, 1.370) |
| ASDR in 2019 (per 100,000) | 60.165 (41.610, 81.012) | 361.538 (240.392, 510.245) | 237.028 (167.467, 320.495) | 99.736 (68.234, 135.259) | 294.685 (190.485, 416.396) |
| AAPC on ASDR, 1990-2019 | -0.524 (-0.745, -0.303) | -1.187 (-1.429, -0.945) | -1.918 (-2.163, -1.671) | -1.142 (-1.339, -0.944) | 0.987 (0.850, 1.124) |
| North Africa and Middle East | | | | | |
| DALYs in 2019 (million) | 0.382 (0.277, 0.501) | 4.615 (3.420, 5.975) | 2.157 (1.658, 2.716) | 0.631 (0.468, 0.823) | 0.996 (0.689, 1.358) |
| ASDR in 2019 (per 100,000) | 150.286 (108.881, 196.876) | 1796.031 (1330.851, 2318.433) | 843.625 (648.101, 1060.162) | 269.093 (199.394, 350.695) | 398.931 (277.270, 539.108) |
| AAPC on ASDR, 1990-2019 | 0.685 (0.415, 0.955) | -0.379 (-0.476, -0.282) | 0.081 (-0.075, 0.237)* | 0.319 (0.060, 0.578) | 2.066 (1.923, 2.209) |
| South Asia | | | | | |
| DALYs in 2019 (million) | 0.621 (0.423, 0.838) | 10.674 (7.716, 13.862) | 5.764 (4.111, 7.489) | 7.255 (5.478, 9.171) | 2.303 (1.488, 3.224) |
| ASDR in 2019 (per 100,000) | 75.971 (51.675, 102.483) | 1279.506 (924.304, 1662.580) | 709.458 (505.658, 923.235) | 1005.368 (758.275, 1270.528) | 289.869 (188.096, 404.219) |
| AAPC on ASDR, 1990-2019 | 3.353 (2.926, 3.783) | 2.817 (2.301, 3.336) | 2.138 (1.511, 2.769) | 1.584 (0.929, 2.244) | 4.716 (4.351, 5.081) |
| Southeast Asia, East Asia, and Oceania | | | | | |
| DALYs in 2019 (million) | 4.341 (3.092, 5.770) | 10.183 (7.531, 13.002) | 15.905 (12.045, 19.785) | 5.845 (4.573, 7.439) | 2.812 (1.844, 3.872) |
| ASDR in 2019 (per 100,000) | 283.301 (202.113, 375.976) | 697.296 (515.814, 890.383) | 1072.897 (812.023, 1335.537) | 435.603 (340.441, 555.761) | 184.995 (121.538, 254.504) |
| AAPC on ASDR, 1990-2019 | 2.689 (2.441, 2.938) | 2.416 (2.108, 2.725) | 0.967 (0.671, 1.264) | -2.164 (-2.519, -1.808) | 3.220 (3.081, 3.360) |
| Sub-Saharan Africa | | | | | |
| DALYs in 2019 (million) | 0.126 (0.081, 0.179) | 0.904 (0.561, 1.351) | 1.174 (0.740, 1.720) | 0.424 (0.298, 0.582) | 0.449 (0.282, 0.660) |
| ASDR in 2019 (per 100,000) | 47.571 (30.538, 67.170) | 339.186 (211.352, 503.686) | 430.507 (273.920, 627.154) | 170.138 (119.969, 232.775) | 177.150 (112.134, 258.082) |
| AAPC on ASDR, 1990-2019 | 1.682 (1.457, 1.907) | 1.964 (1.651, 2.277) | 1.694 (1.412, 1.977) | 1.090 (0.740, 1.440) | 3.232 (3.009, 3.456) |
| TBL: tracheal, bronchus, and lung; IHD: ischemic heart disease; COPD: chronic obstructive pulmonary disease; ASDR: age-standardized DALY rate; AAPC: average annual percent change.  ***NOT** statistically significant since *P* value > 0.05. | | | | | |

| **Table S2 The ASMR of non-communicable diseases attributed to ambient particulate matter pollution in 2019 as well as temporal trends from 1990-2019, by national level** | | | | | |
| --- | --- | --- | --- | --- | --- |
| Location | TBL cancer | IHD | Stroke | COPD | Diabetes mellitus |
| Afghanistan |  |  |  |  |  |
| ASMR in 2019 (per 100,000) | 1.996 (0.664, 4.627) | 45.161 (18.392, 86.948) | 26.021 (10.290, 52.381) | 10.533 (4.468, 19.537) | 4.200 (1.381, 8.904) |
| AAPC on ASMR, 1990-2019 | 2.882 (2.659, 3.105) | 2.394 (2.074, 2.716) | 2.629 (2.289, 2.970) | 2.345 (2.097, 2.594) | 4.735 (4.311, 5.162) |
| Albania |  |  |  |  |  |
| ASMR in 2019 (per 100,000) | 5.790 (3.481, 8.977) | 26.678 (16.332, 40.578) | 26.789 (17.406, 39.171) | 3.225 (1.943, 4.926) | 0.782 (0.433, 1.271) |
| AAPC on ASMR, 1990-2019 | 0.173 (-0.290, 0.638)* | 0.299 (-0.131, 0.731)* | -0.438 (-0.966, 0.093)* | -3.761 (-4.134, -3.386) | 1.189 (0.776, 1.605) |
| Algeria |  |  |  |  |  |
| ASMR in 2019 (per 100,000) | 3.905 (2.253, 6.186) | 73.093 (44.142, 106.880) | 35.224 (21.309, 52.916) | 7.960 (4.443, 12.876) | 7.014 (4.154, 10.876) |
| AAPC on ASMR, 1990-2019 | 0.036 (-0.089, 0.160)* | -1.066 (-1.185, -0.946) | -1.198 (-1.357, -1.040) | -0.731 (-0.991, -0.471) | 1.156 (1.076, 1.236) |
| American Samoa |  |  |  |  |  |
| ASMR in 2019 (per 100,000) | 1.834 (0.658, 3.924) | 10.105 (3.503, 21.973) | 7.198 (2.591, 15.022) | 2.753 (0.921, 5.772) | 7.677 (2.656, 17.493) |
| AAPC on ASMR, 1990-2019 | -0.446 (-0.686, -0.206) | -0.195 (-0.397, 0.007)* | -1.317 (-1.566, -1.067) | -2.291 (-2.547, -2.035) | 1.320 (0.883, 1.760) |
| Andorra |  |  |  |  |  |
| ASMR in 2019 (per 100,000) | 3.378 (1.595, 5.903) | 4.023 (1.705, 7.602) | 1.946 (0.868, 3.651) | 1.906 (0.840, 3.418) | 0.780 (0.339, 1.418) |
| AAPC on ASMR, 1990-2019 | -2.782 (-2.904, -2.661) | -3.827 (-3.993, -3.661) | -3.887 (-4.075, -3.699) | -3.385 (-3.541, -3.229) | -2.565 (-2.712, -2.418) |
| Angola |  |  |  |  |  |
| ASMR in 2019 (per 100,000) | 2.877 (1.260, 5.358) | 18.750 (8.388, 34.029) | 23.464 (10.904, 41.882) | 7.761 (3.967, 13.695) | 6.721 (3.001, 11.967) |
| AAPC on ASMR, 1990-2019 | 4.769 (4.365, 5.175) | 4.541 (4.178, 4.906) | 3.959 (3.551, 4.369) | 2.285 (2.006, 2.563) | 5.594 (5.186, 6.004) |
| Antigua and Barbuda |  |  |  |  |  |
| ASMR in 2019 (per 100,000) | 2.124 (0.757, 3.999) | 17.164 (5.826, 32.018) | 15.702 (5.396, 29.791) | 1.772 (0.580, 3.446) | 15.765 (6.340, 26.081) |
| AAPC on ASMR, 1990-2019 | -0.314 (-1.268, 0.650)* | -1.472 (-1.930, -1.011) | -1.453 (-2.039, -0.863) | 0.424 (-0.609, 1.467)* | 0.361 (-0.143, 0.866)* |
| Argentina |  |  |  |  |  |
| ASMR in 2019 (per 100,000) | 4.308 (2.486, 6.666) | 12.423 (6.590, 19.945) | 8.131 (4.511, 13.084) | 4.645 (2.522, 7.338) | 3.931 (2.207, 6.047) |
| AAPC on ASMR, 1990-2019 | -0.936 (-1.135, -0.737) | -2.655 (-2.896, -2.412) | -3.043 (-3.379, -2.706) | -0.213 (-0.477, 0.052)* | -0.093 (-0.424, 0.240)* |
| Armenia |  |  |  |  |  |
| ASMR in 2019 (per 100,000) | 11.630 (7.276, 16.741) | 74.382 (46.753, 105.339) | 24.932 (15.334, 36.749) | 12.002 (6.796, 18.171) | 10.552 (6.895, 14.899) |
| AAPC on ASMR, 1990-2019 | 0.285 (-0.168, 0.740)* | -0.230 (-0.486, 0.027)* | -1.352 (-1.877, -0.824) | -0.691 (-1.156, -0.224) | 2.895 (2.355, 3.437) |
| Australia |  |  |  |  |  |
| ASMR in 2019 (per 100,000) | 1.307 (0.318, 2.588) | 2.594 (0.598, 5.528) | 1.170 (0.277, 2.537) | 0.975 (0.243, 2.006) | 0.728 (0.169, 1.509) |
| AAPC on ASMR, 1990-2019 | -2.115 (-2.417, -1.813) | -5.053 (-5.501, -4.603) | -4.140 (-4.581, -3.697) | -2.485 (-2.777, -2.192) | -1.440 (-1.776, -1.103) |
| Austria |  |  |  |  |  |
| ASMR in 2019 (per 100,000) | 3.677 (2.380, 5.339) | 10.086 (5.769, 15.864) | 3.150 (1.909, 4.900) | 2.114 (1.314, 3.128) | 2.231 (1.318, 3.344) |
| AAPC on ASMR, 1990-2019 | -2.460 (-2.829, -2.089) | -4.589 (-4.945, -4.232) | -6.346 (-6.620, -6.072) | -2.304 (-2.670, -1.937) | -1.804 (-2.236, -1.370) |
| Azerbaijan |  |  |  |  |  |
| ASMR in 2019 (per 100,000) | 6.974 (3.502, 11.492) | 112.002 (59.427, 172.893) | 49.842 (26.338, 79.479) | 7.585 (3.550, 14.113) | 7.285 (3.958, 11.571) |
| AAPC on ASMR, 1990-2019 | 0.820 (0.565, 1.076) | 2.051 (1.729, 2.375) | 2.122 (1.738, 2.508) | 0.305 (-0.087, 0.698)* | 4.726 (4.467, 4.986) |
| Bahamas |  |  |  |  |  |
| ASMR in 2019 (per 100,000) | 2.829 (0.765, 5.826) | 17.528 (4.803, 35.221) | 12.838 (3.421, 26.726) | 1.792 (0.462, 3.782) | 9.224 (2.801, 16.854) |
| AAPC on ASMR, 1990-2019 | -0.710 (-1.107, -0.311) | -1.482 (-1.740, -1.223) | -1.334 (-1.532, -1.135) | -0.433 (-0.784, -0.080) | -0.686 (-0.965, -0.406) |
| Bahrain |  |  |  |  |  |
| ASMR in 2019 (per 100,000) | 11.313 (7.070, 16.897) | 66.424 (47.440, 89.412) | 26.356 (18.207, 37.608) | 17.309 (11.480, 24.711) | 54.895 (36.395, 77.472) |
| AAPC on ASMR, 1990-2019 | -2.390 (-2.756, -2.023) | -3.598 (-4.247, -2.944) | -2.352 (-3.169, -1.528) | -2.518 (-3.676, -1.346) | 1.886 (1.242, 2.535) |
| Bangladesh |  |  |  |  |  |
| ASMR in 2019 (per 100,000) | 2.193 (1.028, 4.222) | 27.901 (16.038, 42.752) | 40.136 (23.197, 60.952) | 20.756 (12.542, 37.663) | 5.696 (3.031, 9.244) |
| AAPC on ASMR, 1990-2019 | 3.452 (3.001, 3.906) | 4.106 (3.643, 4.572) | 3.483 (3.215, 3.751) | 0.688 (0.327, 1.050) | 4.690 (3.898, 5.488) |
| Barbados |  |  |  |  |  |
| ASMR in 2019 (per 100,000) | 2.568 (1.044, 4.574) | 16.395 (6.446, 28.749) | 17.227 (6.660, 31.736) | 2.138 (0.810, 3.965) | 18.936 (8.939, 29.818) |
| AAPC on ASMR, 1990-2019 | -0.068 (-0.957, 0.828)* | -2.079 (-2.602, -1.553) | -1.692 (-2.188, -1.194) | 0.412 (-0.085, 0.912)* | -0.384 (-1.008, 0.243)* |
| Belarus |  |  |  |  |  |
| ASMR in 2019 (per 100,000) | 4.688 (2.808, 7.268) | 63.134 (36.293, 97.995) | 21.950 (13.259, 33.807) | 2.745 (1.604, 4.818) | 0.583 (0.338, 0.910) |
| AAPC on ASMR, 1990-2019 | -2.614 (-3.303, -1.921) | -0.943 (-1.658, -0.222) | -2.000 (-2.508, -1.489) | -5.456 (-6.002, -4.906) | -2.858 (-3.645, -2.065) |
| Belgium |  |  |  |  |  |
| ASMR in 2019 (per 100,000) | 5.600 (3.639, 8.034) | 7.430 (4.311, 11.570) | 4.461 (2.735, 6.908) | 3.605 (2.243, 5.329) | 1.300 (0.769, 1.936) |
| AAPC on ASMR, 1990-2019 | -2.921 (-3.393, -2.446) | -5.051 (-5.340, -4.760) | -4.781 (-5.075, -4.486) | -2.962 (-3.269, -2.655) | -3.161 (-3.763, -2.555) |
| Belize |  |  |  |  |  |
| ASMR in 2019 (per 100,000) | 3.164 (1.058, 6.023) | 18.226 (6.433, 33.660) | 13.837 (5.017, 26.192) | 5.105 (1.817, 9.882) | 13.577 (4.625, 23.142) |
| AAPC on ASMR, 1990-2019 | 2.438 (1.783, 3.098) | 0.393 (-0.001, 0.788)* | 0.798 (0.067, 1.533) | 1.611 (0.868, 2.360) | 3.474 (2.497, 4.460) |
| Benin |  |  |  |  |  |
| ASMR in 2019 (per 100,000) | 1.563 (0.586, 3.080) | 12.780 (5.216, 24.520) | 16.176 (6.819, 30.363) | 5.864 (2.715, 10.615) | 3.178 (1.181, 6.340) |
| AAPC on ASMR, 1990-2019 | 2.939 (2.591, 3.289) | 2.221 (1.786, 2.658) | 2.013 (1.526, 2.503) | 0.984 (0.584, 1.385) | 3.717 (3.347, 4.089) |
| Bermuda |  |  |  |  |  |
| ASMR in 2019 (per 100,000) | 1.566 (0.280, 3.404) | 4.435 (0.770, 9.791) | 1.941 (0.346, 4.321) | 0.503 (0.088, 1.073) | 1.572 (0.244, 3.456) |
| AAPC on ASMR, 1990-2019 | -3.480 (-3.925, -3.032) | -5.778 (-6.146, -5.409) | -4.835 (-5.328, -4.339) | -3.250 (-3.637, -2.862) | -3.243 (-3.820, -2.662) |
| Bhutan |  |  |  |  |  |
| ASMR in 2019 (per 100,000) | 1.931 (0.933, 3.411) | 27.718 (14.919, 43.973) | 16.596 (8.967, 26.928) | 33.290 (20.612, 50.823) | 5.697 (2.824, 9.734) |
| AAPC on ASMR, 1990-2019 | 5.977 (5.758, 6.197) | 5.448 (5.263, 5.634) | 3.737 (3.532, 3.942) | 3.512 (3.362, 3.661) | 7.530 (7.308, 7.753) |
| Bolivia (Plurinational State of) |  |  |  |  |  |
| ASMR in 2019 (per 100,000) | 3.642 (1.714, 6.420) | 22.522 (11.440, 37.512) | 18.105 (9.304, 30.630) | 10.473 (5.809, 17.053) | 10.557 (5.528, 17.434) |
| AAPC on ASMR, 1990-2019 | 0.578 (0.319, 0.838) | -0.577 (-0.811, -0.343) | -1.241 (-1.449, -1.033) | -1.119 (-1.277, -0.960) | 1.794 (1.574, 2.015) |
| Bosnia and Herzegovina |  |  |  |  |  |
| ASMR in 2019 (per 100,000) | 12.305 (7.858, 17.446) | 41.320 (27.603, 58.204) | 39.969 (27.465, 55.251) | 6.511 (4.269, 9.333) | 10.911 (6.440, 16.175) |
| AAPC on ASMR, 1990-2019 | 1.100 (0.631, 1.572) | -0.341 (-0.989, 0.311)* | 0.511 (0.084, 0.941) | -1.536 (-2.408, -0.656) | 5.329 (4.561, 6.103) |
| Botswana |  |  |  |  |  |
| ASMR in 2019 (per 100,000) | 5.609 (2.854, 9.507) | 29.808 (16.104, 48.145) | 34.472 (19.383, 54.953) | 10.864 (5.755, 18.169) | 20.734 (10.947, 34.100) |
| AAPC on ASMR, 1990-2019 | 3.292 (3.007, 3.577) | 3.183 (2.933, 3.434) | 2.119 (1.886, 2.353) | 0.498 (0.258, 0.738) | 5.194 (4.715, 5.675) |
| Brazil |  |  |  |  |  |
| ASMR in 2019 (per 100,000) | 2.194 (1.401, 3.159) | 10.235 (6.060, 15.719) | 8.251 (5.241, 11.997) | 4.170 (2.603, 6.226) | 4.370 (2.600, 6.690) |
| AAPC on ASMR, 1990-2019 | -0.342 (-0.551, -0.132) | -2.193 (-2.355, -2.031) | -2.831 (-3.027, -2.635) | -2.109 (-2.460, -1.756) | 0.449 (0.264, 0.635) |
| Brunei Darussalam |  |  |  |  |  |
| ASMR in 2019 (per 100,000) | 3.298 (0.931, 6.414) | 8.894 (2.325, 18.212) | 5.304 (1.428, 10.741) | 3.208 (0.843, 6.361) | 7.728 (2.013, 15.254) |
| AAPC on ASMR, 1990-2019 | -0.869 (-1.810, 0.080)* | -2.219 (-3.157, -1.272) | -3.652 (-4.511, -2.785) | -3.020 (-3.716, -2.319) | -1.372 (-1.975, -0.765) |
| Bulgaria |  |  |  |  |  |
| ASMR in 2019 (per 100,000) | 8.178 (5.293, 11.821) | 49.461 (31.691, 72.294) | 44.016 (29.856, 61.918) | 4.598 (3.008, 6.709) | 3.729 (2.272, 5.417) |
| AAPC on ASMR, 1990-2019 | 0.111 (-0.265, 0.489)* | -2.050 (-2.313, -1.786) | -1.766 (-2.121, -1.409) | -2.458 (-2.659, -2.256) | -0.351 (-0.658, -0.044) |
| Burkina Faso |  |  |  |  |  |
| ASMR in 2019 (per 100,000) | 1.025 (0.300, 2.406) | 10.459 (3.193, 22.995) | 9.154 (2.870, 20.199) | 3.259 (1.204, 6.734) | 2.286 (0.624, 5.327) |
| AAPC on ASMR, 1990-2019 | 2.953 (2.596, 3.310) | 2.885 (2.485, 3.287) | 2.471 (2.152, 2.790) | 1.507 (0.995, 2.021) | 2.443 (2.115, 2.772) |
| Burundi |  |  |  |  |  |
| ASMR in 2019 (per 100,000) | 0.520 (0.143, 1.290) | 6.582 (1.904, 15.734) | 8.773 (2.568, 20.862) | 5.107 (1.723, 11.049) | 1.648 (0.440, 3.925) |
| AAPC on ASMR, 1990-2019 | 0.658 (0.408, 0.908) | 0.525 (0.349, 0.701) | -0.394 (-0.607, -0.181) | -0.174 (-0.353, 0.005)* | 0.451 (0.201, 0.702) |
| Cabo Verde |  |  |  |  |  |
| ASMR in 2019 (per 100,000) | 7.325 (4.116, 11.394) | 46.351 (29.113, 66.432) | 33.667 (20.101, 50.480) | 8.723 (5.190, 13.719) | 10.180 (6.054, 15.301) |
| AAPC on ASMR, 1990-2019 | 6.431 (5.335, 7.539) | 4.857 (4.137, 5.583) | 5.379 (4.527, 6.237) | 0.747 (0.043, 1.456) | 9.776 (8.656, 10.908) |
| Cambodia |  |  |  |  |  |
| ASMR in 2019 (per 100,000) | 3.204 (1.337, 6.049) | 12.095 (5.483, 21.813) | 18.421 (8.448, 33.374) | 6.599 (3.445, 11.263) | 2.695 (1.104, 5.069) |
| AAPC on ASMR, 1990-2019 | 2.953 (2.821, 3.084) | 2.607 (2.426, 2.788) | 2.168 (2.055, 2.280) | 1.423 (1.231, 1.614) | 3.196 (3.050, 3.341) |
| Cameroon |  |  |  |  |  |
| ASMR in 2019 (per 100,000) | 4.335 (2.100, 7.395) | 28.953 (15.481, 46.937) | 34.238 (17.953, 54.532) | 10.925 (5.936, 17.267) | 10.365 (5.090, 17.569) |
| AAPC on ASMR, 1990-2019 | 3.131 (2.932, 3.329) | 2.805 (2.584, 3.027) | 2.273 (1.958, 2.590) | 0.330 (0.085, 0.575) | 3.677 (3.514, 3.840) |
| Canada |  |  |  |  |  |
| ASMR in 2019 (per 100,000) | 2.127 (0.903, 3.749) | 3.492 (1.356, 6.753) | 1.232 (0.494, 2.431) | 1.118 (0.444, 2.037) | 0.837 (0.329, 1.582) |
| AAPC on ASMR, 1990-2019 | -3.044 (-3.302, -2.786) | -5.207 (-5.461, -4.951) | -4.548 (-4.787, -4.308) | -2.818 (-3.075, -2.560) | -2.691 (-2.997, -2.384) |
| Central African Republic |  |  |  |  |  |
| ASMR in 2019 (per 100,000) | 1.206 (0.259, 3.206) | 11.598 (3.237, 27.228) | 15.453 (4.473, 36.298) | 8.284 (2.714, 19.051) | 3.039 (0.782, 7.189) |
| AAPC on ASMR, 1990-2019 | 0.946 (0.791, 1.100) | 1.295 (1.122, 1.468) | 1.151 (0.840, 1.462) | 0.855 (0.617, 1.094) | 1.472 (1.328, 1.616) |
| Chad |  |  |  |  |  |
| ASMR in 2019 (per 100,000) | 1.233 (0.354, 2.957) | 9.724 (3.038, 22.601) | 11.400 (3.666, 25.771) | 5.507 (2.018, 11.851) | 2.112 (0.567, 5.018) |
| AAPC on ASMR, 1990-2019 | 3.694 (3.506, 3.883) | 2.477 (2.122, 2.834) | 2.343 (2.104, 2.581) | 1.697 (1.428, 1.966) | 3.490 (3.272, 3.708) |
| Chile |  |  |  |  |  |
| ASMR in 2019 (per 100,000) | 4.660 (3.221, 6.375) | 12.584 (8.299, 17.467) | 12.027 (8.079, 16.760) | 5.194 (3.263, 7.420) | 4.921 (3.271, 6.771) |
| AAPC on ASMR, 1990-2019 | 0.390 (0.089, 0.692) | -2.234 (-2.646, -1.821) | -1.873 (-2.206, -1.539) | -0.298 (-0.799, 0.207)* | 1.332 (0.973, 1.692) |
| China |  |  |  |  |  |
| ASMR in 2019 (per 100,000) | 15.835 (11.262, 21.089) | 38.563 (28.408, 49.625) | 53.454 (39.913, 67.641) | 30.032 (22.761, 40.025) | 3.141 (2.177, 4.210) |
| AAPC on ASMR, 1990-2019 | 3.349 (3.002, 3.697) | 3.162 (2.862, 3.463) | 0.921 (0.414, 1.432) | -2.563 (-3.046, -2.077) | 3.464 (3.222, 3.706) |
| Colombia |  |  |  |  |  |
| ASMR in 2019 (per 100,000) | 3.071 (1.869, 4.708) | 17.127 (10.296, 26.254) | 8.201 (4.882, 12.762) | 7.777 (4.544, 11.876) | 3.539 (2.115, 5.353) |
| AAPC on ASMR, 1990-2019 | -0.509 (-1.139, 0.126)* | -1.846 (-2.476, -1.213) | -2.584 (-3.130, -2.035) | -1.054 (-1.584, -0.521) | 0.027 (-0.538, 0.595)* |
| Comoros |  |  |  |  |  |
| ASMR in 2019 (per 100,000) | 0.698 (0.277, 1.372) | 7.721 (3.280, 14.698) | 9.355 (4.047, 17.302) | 3.328 (1.660, 5.742) | 2.236 (0.915, 4.424) |
| AAPC on ASMR, 1990-2019 | 2.878 (2.487, 3.270) | 2.757 (2.335, 3.180) | 1.891 (1.502, 2.282) | 0.973 (0.645, 1.304) | 3.127 (2.748, 3.508) |
| Congo |  |  |  |  |  |
| ASMR in 2019 (per 100,000) | 4.850 (2.200, 8.931) | 35.115 (17.198, 60.497) | 38.689 (19.576, 66.068) | 12.353 (6.043, 22.223) | 11.685 (5.615, 19.652) |
| AAPC on ASMR, 1990-2019 | 2.818 (2.501, 3.137) | 2.667 (2.210, 3.127) | 2.116 (1.660, 2.574) | 0.830 (0.475, 1.186) | 3.386 (3.034, 3.740) |
| Cook Islands |  |  |  |  |  |
| ASMR in 2019 (per 100,000) | 1.698 (0.290, 4.348) | 6.055 (1.057, 15.414) | 3.779 (0.676, 9.515) | 1.300 (0.240, 3.134) | 8.543 (1.572, 21.161) |
| AAPC on ASMR, 1990-2019 | -0.948 (-1.158, -0.736) | -1.153 (-1.242, -1.064) | -2.189 (-2.588, -1.789) | -3.094 (-3.425, -2.762) | 0.453 (0.138, 0.768) |
| Costa Rica |  |  |  |  |  |
| ASMR in 2019 (per 100,000) | 2.132 (1.293, 3.209) | 14.397 (8.345, 22.393) | 6.008 (3.491, 9.369) | 4.967 (2.968, 7.533) | 2.905 (1.711, 4.409) |
| AAPC on ASMR, 1990-2019 | 0.732 (0.366, 1.099) | -0.568 (-1.046, -0.088) | -0.766 (-1.268, -0.262) | 0.307 (-0.427, 1.046)* | 0.910 (-0.422, 2.260)* |
| Coted'Ivoire |  |  |  |  |  |
| ASMR in 2019 (per 100,000) | 2.793 (1.171, 5.303) | 20.577 (9.063, 37.079) | 21.054 (9.433, 38.190) | 8.001 (3.900, 14.012) | 5.355 (2.213, 9.916) |
| AAPC on ASMR, 1990-2019 | 2.735 (2.441, 3.030) | 1.758 (1.329, 2.189) | 1.774 (1.333, 2.217) | 0.498 (0.095, 0.902) | 3.126 (2.669, 3.586) |
| Croatia |  |  |  |  |  |
| ASMR in 2019 (per 100,000) | 7.853 (5.051, 11.392) | 27.037 (16.567, 40.537) | 16.892 (10.924, 24.608) | 4.194 (2.620, 6.053) | 3.166 (1.947, 4.616) |
| AAPC on ASMR, 1990-2019 | -1.478 (-2.114, -0.837) | -3.076 (-3.261, -2.891) | -3.416 (-4.029, -2.798) | -0.518 (-0.749, -0.286) | -0.373 (-1.241, 0.504)* |
| Cuba |  |  |  |  |  |
| ASMR in 2019 (per 100,000) | 7.553 (3.548, 13.291) | 21.685 (9.696, 37.874) | 11.886 (5.368, 21.354) | 4.537 (1.983, 8.336) | 2.649 (1.306, 4.304) |
| AAPC on ASMR, 1990-2019 | -0.072 (-0.517, 0.374)* | -2.222 (-2.852, -1.589) | -1.269 (-1.695, -0.840) | 0.746 (0.004, 1.494) | -2.280 (-2.756, -1.802) |
| Cyprus |  |  |  |  |  |
| ASMR in 2019 (per 100,000) | 4.742 (2.995, 6.953) | 16.261 (9.566, 25.340) | 7.387 (4.676, 11.153) | 4.988 (2.745, 7.665) | 6.995 (4.079, 10.515) |
| AAPC on ASMR, 1990-2019 | 0.217 (-0.144, 0.578)* | -3.300 (-3.861, -2.735) | -3.908 (-4.589, -3.222) | -2.304 (-2.610, -1.997) | -3.049 (-3.640, -2.455) |
| Czechia |  |  |  |  |  |
| ASMR in 2019 (per 100,000) | 6.317 (4.161, 9.022) | 26.065 (15.796, 39.406) | 9.793 (6.300, 14.532) | 3.356 (2.115, 4.895) | 3.886 (2.384, 5.633) |
| AAPC on ASMR, 1990-2019 | -2.770 (-3.211, -2.327) | -4.185 (-4.647, -3.721) | -5.695 (-6.153, -5.235) | -1.987 (-2.522, -1.449) | -0.079 (-0.694, 0.540)* |
| Democratic People's Republic of Korea |  |  |  |  |  |
| ASMR in 2019 (per 100,000) | 7.124 (3.684, 11.691) | 26.959 (14.915, 42.632) | 50.638 (29.054, 78.204) | 35.322 (21.652, 53.652) | 2.189 (1.121, 3.724) |
| AAPC on ASMR, 1990-2019 | 2.306 (2.258, 2.354) | 2.838 (2.695, 2.981) | 2.047 (1.959, 2.135) | 0.185 (0.059, 0.310) | 2.141 (2.055, 2.227) |
| Democratic Republic of the Congo |  |  |  |  |  |
| ASMR in 2019 (per 100,000) | 1.430 (0.408, 3.739) | 10.699 (3.959, 21.777) | 13.428 (5.095, 27.211) | 8.278 (3.369, 17.582) | 2.781 (0.966, 5.714) |
| AAPC on ASMR, 1990-2019 | 0.984 (0.700, 1.268) | 1.253 (0.896, 1.612) | 1.081 (0.696, 1.469) | 0.817 (0.542, 1.092) | 1.102 (0.812, 1.392) |
| Denmark |  |  |  |  |  |
| ASMR in 2019 (per 100,000) | 4.127 (2.281, 6.459) | 5.093 (2.562, 8.685) | 3.367 (1.751, 5.730) | 3.342 (1.754, 5.326) | 1.698 (0.885, 2.770) |
| AAPC on ASMR, 1990-2019 | -3.156 (-3.348, -2.964) | -6.832 (-7.274, -6.388) | -5.063 (-5.432, -4.692) | -2.377 (-2.834, -1.919) | -1.233 (-1.688, -0.775) |
| Djibouti |  |  |  |  |  |
| ASMR in 2019 (per 100,000) | 3.924 (1.584, 7.994) | 34.428 (14.897, 59.974) | 39.239 (17.978, 65.584) | 8.848 (3.864, 16.675) | 12.248 (6.109, 20.223) |
| AAPC on ASMR, 1990-2019 | 4.740 (4.586, 4.895) | 4.711 (4.390, 5.032) | 3.422 (3.274, 3.571) | 1.630 (1.500, 1.760) | 5.444 (5.273, 5.615) |
| Dominica |  |  |  |  |  |
| ASMR in 2019 (per 100,000) | 3.997 (1.530, 7.579) | 17.882 (6.678, 33.273) | 16.645 (6.382, 30.985) | 3.776 (1.324, 7.391) | 15.888 (6.524, 27.394) |
| AAPC on ASMR, 1990-2019 | 1.081 (0.848, 1.315) | -0.573 (-0.947, -0.199) | 0.124 (-0.246, 0.495)* | 0.716 (0.500, 0.934) | 1.833 (1.541, 2.126) |
| Dominican Republic |  |  |  |  |  |
| ASMR in 2019 (per 100,000) | 3.234 (1.192, 6.369) | 34.170 (13.195, 65.352) | 20.747 (8.246, 40.575) | 3.370 (1.295, 6.755) | 6.946 (2.700, 12.996) |
| AAPC on ASMR, 1990-2019 | 3.805 (3.317, 4.294) | 3.967 (3.287, 4.651) | 3.340 (2.680, 4.005) | 2.232 (1.573, 2.894) | 5.386 (4.681, 6.097) |
| Ecuador |  |  |  |  |  |
| ASMR in 2019 (per 100,000) | 2.653 (1.530, 4.176) | 17.264 (9.681, 27.046) | 11.062 (6.423, 17.108) | 5.459 (2.992, 8.514) | 9.742 (5.682, 14.685) |
| AAPC on ASMR, 1990-2019 | 1.383 (1.006, 1.761) | 0.016 (-0.269, 0.302)* | -1.068 (-1.443, -0.692) | -0.462 (-0.894, -0.028) | 3.321 (2.814, 3.832) |
| Egypt |  |  |  |  |  |
| ASMR in 2019 (per 100,000) | 5.035 (2.912, 7.920) | 176.644 (120.269, 242.321) | 50.473 (31.161, 78.478) | 18.201 (10.255, 28.825) | 14.681 (8.931, 22.152) |
| AAPC on ASMR, 1990-2019 | 1.632 (1.172, 2.094) | 0.145 (-0.428, 0.720)* | -0.127 (-0.753, 0.503)* | 0.258 (-0.440, 0.961)* | 1.934 (1.333, 2.538) |
| El Salvador |  |  |  |  |  |
| ASMR in 2019 (per 100,000) | 2.580 (1.396, 4.339) | 22.036 (11.954, 36.114) | 8.787 (4.881, 14.589) | 4.768 (2.552, 7.920) | 9.390 (5.099, 15.056) |
| AAPC on ASMR, 1990-2019 | 3.022 (2.207, 3.844) | 1.868 (0.935, 2.809) | -0.201 (-1.437, 1.052)* | 0.565 (-0.027, 1.160)* | 6.369 (5.091, 7.662) |
| Equatorial Guinea |  |  |  |  |  |
| ASMR in 2019 (per 100,000) | 6.942 (3.183, 12.747) | 33.218 (16.564, 55.778) | 39.359 (20.053, 65.434) | 13.901 (6.055, 26.856) | 18.891 (10.136, 30.422) |
| AAPC on ASMR, 1990-2019 | 7.975 (7.619, 8.331) | 5.580 (5.090, 6.073) | 5.059 (4.592, 5.529) | 3.407 (2.951, 3.866) | 8.766 (8.484, 9.048) |
| Eritrea |  |  |  |  |  |
| ASMR in 2019 (per 100,000) | 1.458 (0.554, 2.876) | 16.254 (6.242, 31.711) | 22.956 (9.269, 44.892) | 8.546 (3.776, 15.907) | 5.264 (1.965, 10.484) |
| AAPC on ASMR, 1990-2019 | 4.051 (3.907, 4.196) | 3.743 (3.600, 3.887) | 2.682 (2.556, 2.809) | 1.908 (1.814, 2.001) | 4.082 (3.954, 4.210) |
| Estonia |  |  |  |  |  |
| ASMR in 2019 (per 100,000) | 1.348 (0.448, 2.622) | 5.893 (1.869, 12.072) | 1.877 (0.609, 3.842) | 0.389 (0.124, 0.769) | 0.309 (0.100, 0.640) |
| AAPC on ASMR, 1990-2019 | -4.171 (-5.391, -2.936) | -6.008 (-6.809, -5.200) | -7.919 (-8.722, -7.110) | -4.198 (-5.022, -3.367) | -1.124 (-2.601, 0.376)* |
| Eswatini |  |  |  |  |  |
| ASMR in 2019 (per 100,000) | 3.722 (1.648, 6.768) | 21.744 (11.000, 37.019) | 28.322 (14.635, 46.648) | 10.057 (5.534, 16.758) | 22.447 (10.859, 38.486) |
| AAPC on ASMR, 1990-2019 | 2.672 (2.577, 2.766) | 2.805 (2.543, 3.068) | 2.144 (1.927, 2.362) | 0.341 (0.155, 0.527) | 4.823 (4.579, 5.068) |
| Ethiopia |  |  |  |  |  |
| ASMR in 2019 (per 100,000) | 0.592 (0.258, 1.155) | 6.782 (3.053, 12.694) | 8.648 (3.985, 15.799) | 4.200 (2.081, 7.219) | 2.343 (1.022, 4.419) |
| AAPC on ASMR, 1990-2019 | 3.572 (3.345, 3.800) | 2.799 (2.560, 3.038) | 1.967 (1.795, 2.140) | 1.750 (1.577, 1.924) | 2.552 (2.219, 2.886) |
| Fiji |  |  |  |  |  |
| ASMR in 2019 (per 100,000) | 1.317 (0.362, 3.150) | 31.020 (8.732, 71.210) | 14.302 (4.253, 33.030) | 3.526 (0.968, 8.705) | 32.941 (8.280, 75.589) |
| AAPC on ASMR, 1990-2019 | 2.371 (2.085, 2.658) | 2.052 (1.633, 2.473) | 1.481 (1.099, 1.864) | 0.133 (-0.285, 0.553)* | 5.408 (4.944, 5.873) |
| Finland |  |  |  |  |  |
| ASMR in 2019 (per 100,000) | 0.689 (0.130, 1.540) | 2.778 (0.489, 6.707) | 1.079 (0.194, 2.619) | 0.334 (0.061, 0.760) | 0.170 (0.031, 0.405) |
| AAPC on ASMR, 1990-2019 | -4.640 (-4.920, -4.358) | -6.634 (-6.892, -6.375) | -6.464 (-6.746, -6.180) | -4.129 (-4.546, -3.711) | -5.392 (-5.865, -4.917) |
| France |  |  |  |  |  |
| ASMR in 2019 (per 100,000) | 4.378 (2.743, 6.444) | 4.492 (2.495, 7.209) | 2.923 (1.683, 4.684) | 1.240 (0.734, 1.951) | 1.351 (0.774, 2.098) |
| AAPC on ASMR, 1990-2019 | -1.888 (-2.166, -1.608) | -4.586 (-4.966, -4.204) | -4.975 (-5.340, -4.608) | -4.203 (-4.687, -3.716) | -1.613 (-1.964, -1.261) |
| Gabon |  |  |  |  |  |
| ASMR in 2019 (per 100,000) | 7.645 (3.635, 13.662) | 40.191 (21.309, 64.273) | 40.925 (21.859, 65.920) | 10.564 (4.940, 18.642) | 23.805 (13.546, 36.489) |
| AAPC on ASMR, 1990-2019 | 3.133 (2.738, 3.529) | 2.823 (2.661, 2.987) | 2.003 (1.829, 2.178) | 0.411 (0.131, 0.692) | 4.181 (3.917, 4.446) |
| Gambia |  |  |  |  |  |
| ASMR in 2019 (per 100,000) | 1.372 (0.579, 2.579) | 21.591 (9.545, 39.877) | 20.166 (9.203, 37.225) | 8.674 (4.254, 15.379) | 4.307 (1.728, 8.373) |
| AAPC on ASMR, 1990-2019 | 3.627 (3.078, 4.179) | 3.296 (2.561, 4.035) | 3.132 (2.566, 3.703) | 2.009 (1.534, 2.486) | 4.409 (3.927, 4.893) |
| Georgia |  |  |  |  |  |
| ASMR in 2019 (per 100,000) | 6.414 (3.898, 9.709) | 40.138 (24.408, 60.225) | 34.405 (21.718, 51.112) | 3.327 (1.962, 5.441) | 4.456 (2.576, 6.796) |
| AAPC on ASMR, 1990-2019 | 0.458 (-0.341, 1.263)* | -1.855 (-2.761, -0.941) | -0.871 (-1.553, -0.185) | 0.407 (-0.299, 1.117)* | 3.411 (2.690, 4.138) |
| Germany |  |  |  |  |  |
| ASMR in 2019 (per 100,000) | 4.223 (2.675, 6.170) | 9.748 (5.544, 15.478) | 3.897 (2.327, 6.151) | 2.406 (1.431, 3.604) | 1.908 (1.122, 2.907) |
| AAPC on ASMR, 1990-2019 | -1.983 (-2.207, -1.759) | -5.289 (-5.572, -5.005) | -5.509 (-5.845, -5.172) | -2.986 (-3.348, -2.623) | -3.065 (-3.502, -2.627) |
| Ghana |  |  |  |  |  |
| ASMR in 2019 (per 100,000) | 2.951 (1.630, 4.744) | 34.234 (19.469, 52.850) | 44.136 (25.306, 68.389) | 11.203 (5.648, 17.938) | 10.237 (5.543, 16.625) |
| AAPC on ASMR, 1990-2019 | 3.098 (2.908, 3.289) | 2.946 (2.802, 3.090) | 2.628 (2.491, 2.765) | 1.465 (1.327, 1.603) | 4.583 (4.384, 4.782) |
| Greece |  |  |  |  |  |
| ASMR in 2019 (per 100,000) | 6.860 (4.506, 9.717) | 15.139 (9.076, 22.930) | 9.834 (6.242, 14.582) | 3.172 (1.883, 4.709) | 1.186 (0.725, 1.731) |
| AAPC on ASMR, 1990-2019 | -1.451 (-1.756, -1.145) | -2.507 (-2.801, -2.212) | -3.917 (-4.196, -3.638) | -0.851 (-1.439, -0.259) | -2.051 (-2.516, -1.584) |
| Greenland |  |  |  |  |  |
| ASMR in 2019 (per 100,000) | 4.221 (0.372, 12.454) | 4.921 (0.421, 14.531) | 4.070 (0.371, 12.343) | 2.389 (0.224, 6.873) | 0.683 (0.051, 2.015) |
| AAPC on ASMR, 1990-2019 | -0.992 (-1.447, -0.535) | -3.187 (-3.813, -2.558) | -3.240 (-3.996, -2.477) | -2.455 (-2.912, -1.996) | -1.989 (-2.918, -1.051) |
| Grenada |  |  |  |  |  |
| ASMR in 2019 (per 100,000) | 3.763 (1.408, 6.908) | 25.873 (9.408, 46.019) | 25.098 (9.140, 46.952) | 3.712 (1.231, 7.118) | 21.311 (9.230, 33.977) |
| AAPC on ASMR, 1990-2019 | 1.260 (1.053, 1.466) | -0.192 (-0.494, 0.112)* | -0.280 (-0.761, 0.203)* | 0.436 (0.072, 0.802) | 2.793 (2.413, 3.174) |
| Guam |  |  |  |  |  |
| ASMR in 2019 (per 100,000) | 2.808 (1.075, 5.338) | 15.801 (5.667, 30.920) | 4.991 (1.807, 9.701) | 1.633 (0.573, 3.183) | 2.858 (1.065, 5.496) |
| AAPC on ASMR, 1990-2019 | -1.909 (-3.206, -0.595) | -0.986 (-2.109, 0.149)* | -2.694 (-3.551, -1.829) | -3.549 (-4.739, -2.343) | -1.996 (-2.853, -1.131) |
| Guatemala |  |  |  |  |  |
| ASMR in 2019 (per 100,000) | 1.689 (0.876, 2.748) | 16.824 (9.126, 26.397) | 9.889 (5.230, 15.966) | 5.500 (3.365, 8.311) | 10.073 (5.210, 16.707) |
| AAPC on ASMR, 1990-2019 | 1.779 (0.682, 2.888) | 0.557 (-0.463, 1.587)* | 1.150 (0.496, 1.807) | 0.261 (-0.371, 0.896)* | 7.376 (6.732, 8.024) |
| Guinea |  |  |  |  |  |
| ASMR in 2019 (per 100,000) | 1.237 (0.432, 2.603) | 11.754 (4.348, 24.406) | 13.556 (5.121, 27.745) | 6.231 (2.623, 12.091) | 2.840 (0.947, 6.211) |
| AAPC on ASMR, 1990-2019 | 2.803 (2.596, 3.011) | 2.661 (2.488, 2.834) | 2.323 (2.080, 2.567) | 1.417 (1.298, 1.536) | 3.350 (3.187, 3.514) |
| Guinea-Bissau |  |  |  |  |  |
| ASMR in 2019 (per 100,000) | 1.804 (0.608, 3.984) | 18.323 (7.152, 36.542) | 19.482 (7.857, 38.609) | 7.757 (3.365, 14.772) | 4.195 (1.442, 8.846) |
| AAPC on ASMR, 1990-2019 | 2.140 (2.057, 2.223) | 2.171 (2.074, 2.268) | 1.954 (1.858, 2.050) | 0.693 (0.584, 0.802) | 2.905 (2.835, 2.976) |
| Guyana |  |  |  |  |  |
| ASMR in 2019 (per 100,000) | 2.121 (0.787, 4.058) | 44.292 (16.239, 83.334) | 39.957 (14.919, 77.160) | 3.593 (1.296, 7.146) | 24.488 (9.706, 41.859) |
| AAPC on ASMR, 1990-2019 | 0.882 (0.344, 1.422) | -0.335 (-0.762, 0.094)* | -1.434 (-1.914, -0.953) | 0.146 (-0.634, 0.932)* | 2.009 (1.586, 2.434) |
| Haiti |  |  |  |  |  |
| ASMR in 2019 (per 100,000) | 1.051 (0.312, 2.537) | 14.363 (5.054, 30.701) | 14.804 (4.993, 32.214) | 4.761 (1.811, 10.101) | 3.457 (1.137, 8.002) |
| AAPC on ASMR, 1990-2019 | 1.902 (1.676, 2.129) | 1.590 (1.360, 1.820) | 1.483 (1.258, 1.708) | 1.512 (1.255, 1.770) | 1.791 (1.533, 2.049) |
| Honduras |  |  |  |  |  |
| ASMR in 2019 (per 100,000) | 3.482 (1.488, 6.678) | 19.773 (9.748, 33.802) | 16.760 (8.197, 28.763) | 11.816 (5.954, 19.146) | 2.329 (1.077, 4.192) |
| AAPC on ASMR, 1990-2019 | 4.183 (3.712, 4.656) | 2.993 (2.289, 3.701) | 3.264 (2.665, 3.866) | 2.568 (1.884, 3.257) | 4.361 (3.505, 5.225) |
| Hungary |  |  |  |  |  |
| ASMR in 2019 (per 100,000) | 10.071 (6.698, 14.280) | 31.129 (19.554, 45.897) | 12.817 (8.427, 18.469) | 5.621 (3.649, 8.128) | 2.900 (1.805, 4.195) |
| AAPC on ASMR, 1990-2019 | -0.837 (-1.208, -0.464) | -2.696 (-3.127, -2.264) | -4.294 (-4.678, -3.907) | -2.001 (-2.368, -1.633) | -0.075 (-0.511, 0.363)* |
| Iceland |  |  |  |  |  |
| ASMR in 2019 (per 100,000) | 0.942 (0.181, 2.146) | 1.987 (0.363, 4.802) | 0.648 (0.123, 1.564) | 0.482 (0.095, 1.083) | 0.209 (0.036, 0.500) |
| AAPC on ASMR, 1990-2019 | -2.736 (-3.028, -2.443) | -5.473 (-5.760, -5.185) | -5.354 (-5.933, -4.772) | -2.856 (-3.146, -2.565) | -2.392 (-2.707, -2.076) |
| India |  |  |  |  |  |
| ASMR in 2019 (per 100,000) | 3.102 (2.115, 4.190) | 54.423 (39.299, 71.003) | 27.553 (19.685, 36.173) | 53.236 (37.298, 69.240) | 7.696 (5.108, 10.456) |
| AAPC on ASMR, 1990-2019 | 3.248 (2.740, 3.758) | 2.444 (1.567, 3.329) | 1.630 (1.022, 2.242) | 1.076 (-0.085, 2.251)* | 3.956 (2.815, 5.110) |
| Indonesia |  |  |  |  |  |
| ASMR in 2019 (per 100,000) | 5.063 (3.034, 7.609) | 27.335 (17.666, 39.441) | 42.179 (28.404, 58.244) | 10.039 (6.631, 14.248) | 9.825 (6.032, 14.671) |
| AAPC on ASMR, 1990-2019 | 2.529 (2.406, 2.653) | 2.136 (1.931, 2.340) | 1.764 (1.534, 1.995) | -0.220 (-0.489, 0.049)* | 3.780 (3.543, 4.017) |
| Iran (Islamic Republic of) |  |  |  |  |  |
| ASMR in 2019 (per 100,000) | 5.145 (3.808, 6.528) | 57.269 (42.032, 73.212) | 26.251 (20.086, 32.725) | 8.655 (6.229, 11.145) | 8.863 (6.169, 11.436) |
| AAPC on ASMR, 1990-2019 | 0.691 (0.485, 0.897) | -1.620 (-1.784, -1.455) | -1.671 (-1.867, -1.475) | -0.244 (-0.325, -0.162) | 2.317 (2.038, 2.597) |
| Iraq |  |  |  |  |  |
| ASMR in 2019 (per 100,000) | 9.089 (5.808, 13.157) | 106.554 (72.607, 144.404) | 70.818 (47.886, 97.514) | 5.099 (3.149, 7.828) | 18.957 (12.358, 26.901) |
| AAPC on ASMR, 1990-2019 | 1.299 (1.127, 1.471) | 0.028 (-0.162, 0.219)* | 0.253 (-0.031, 0.538)* | -0.289 (-0.462, -0.115) | 0.851 (0.541, 1.162) |
| Ireland |  |  |  |  |  |
| ASMR in 2019 (per 100,000) | 2.201 (1.022, 3.728) | 4.811 (2.059, 8.862) | 1.875 (0.833, 3.493) | 1.867 (0.836, 3.177) | 0.679 (0.295, 1.220) |
| AAPC on ASMR, 1990-2019 | -3.492 (-3.807, -3.176) | -6.489 (-6.834, -6.143) | -6.069 (-6.434, -5.702) | -4.642 (-5.042, -4.241) | -3.759 (-4.215, -3.301) |
| Israel |  |  |  |  |  |
| ASMR in 2019 (per 100,000) | 5.491 (3.814, 7.411) | 9.907 (6.326, 14.094) | 5.831 (3.873, 8.296) | 3.327 (2.176, 4.745) | 6.278 (4.054, 8.672) |
| AAPC on ASMR, 1990-2019 | -0.695 (-1.083, -0.305) | -4.993 (-5.532, -4.452) | -3.683 (-4.254, -3.109) | -2.223 (-2.733, -1.710) | 0.439 (-0.327, 1.211)* |
| Italy |  |  |  |  |  |
| ASMR in 2019 (per 100,000) | 5.215 (3.638, 7.084) | 8.904 (5.510, 13.220) | 5.723 (3.875, 8.074) | 2.733 (1.786, 3.857) | 2.919 (1.846, 4.092) |
| AAPC on ASMR, 1990-2019 | -2.651 (-2.951, -2.350) | -4.248 (-4.556, -3.939) | -4.698 (-5.158, -4.234) | -2.962 (-3.343, -2.580) | -2.386 (-2.782, -1.989) |
| Jamaica |  |  |  |  |  |
| ASMR in 2019 (per 100,000) | 3.644 (2.011, 5.849) | 11.142 (5.973, 18.282) | 17.330 (9.592, 27.840) | 3.118 (1.691, 5.055) | 16.716 (9.174, 26.044) |
| AAPC on ASMR, 1990-2019 | 2.789 (1.464, 4.132) | 1.258 (0.263, 2.264) | 0.778 (-0.112, 1.676)* | 1.959 (0.739, 3.193) | 4.166 (2.986, 5.359) |
| Japan |  |  |  |  |  |
| ASMR in 2019 (per 100,000) | 3.585 (2.160, 5.347) | 4.484 (2.461, 7.123) | 4.587 (2.709, 7.061) | 1.150 (0.615, 1.915) | 0.436 (0.248, 0.655) |
| AAPC on ASMR, 1990-2019 | -0.426 (-0.536, -0.316) | -2.865 (-3.034, -2.696) | -3.277 (-3.519, -3.033) | -1.668 (-1.936, -1.400) | -3.601 (-3.774, -3.429) |
| Jordan |  |  |  |  |  |
| ASMR in 2019 (per 100,000) | 5.461 (3.505, 8.017) | 39.999 (26.508, 56.337) | 26.325 (17.789, 36.662) | 4.500 (2.810, 6.718) | 15.474 (10.132, 21.943) |
| AAPC on ASMR, 1990-2019 | 0.762 (0.339, 1.187) | -2.053 (-2.575, -1.528) | -2.257 (-2.715, -1.796) | -2.320 (-2.902, -1.734) | -1.511 (-2.014, -1.005) |
| Kazakhstan |  |  |  |  |  |
| ASMR in 2019 (per 100,000) | 5.136 (3.026, 7.690) | 51.185 (29.894, 77.436) | 41.223 (24.482, 61.981) | 13.973 (7.755, 22.053) | 4.098 (2.410, 6.171) |
| AAPC on ASMR, 1990-2019 | -1.653 (-1.959, -1.347) | -0.099 (-0.646, 0.452)* | 0.398 (0.066, 0.731) | 0.960 (0.333, 1.590) | 4.296 (3.663, 4.932) |
| Kenya |  |  |  |  |  |
| ASMR in 2019 (per 100,000) | 0.817 (0.429, 1.316) | 8.817 (4.613, 14.794) | 13.624 (7.257, 22.610) | 5.025 (2.932, 8.002) | 3.276 (1.636, 5.455) |
| AAPC on ASMR, 1990-2019 | 3.395 (3.236, 3.553) | 3.577 (3.345, 3.809) | 2.654 (2.449, 2.859) | 1.702 (1.526, 1.878) | 3.561 (3.269, 3.853) |
| Kiribati |  |  |  |  |  |
| ASMR in 2019 (per 100,000) | 1.141 (0.308, 2.881) | 13.932 (3.828, 35.568) | 15.999 (4.556, 39.734) | 4.567 (1.424, 10.798) | 8.296 (2.070, 21.732) |
| AAPC on ASMR, 1990-2019 | 1.213 (0.939, 1.488) | 1.033 (0.839, 1.227) | 0.702 (0.382, 1.022) | 0.400 (0.205, 0.595) | 2.818 (2.548, 3.088) |
| Kuwait |  |  |  |  |  |
| ASMR in 2019 (per 100,000) | 5.581 (3.729, 7.836) | 52.894 (38.633, 69.625) | 24.809 (17.184, 33.911) | 5.105 (3.431, 7.215) | 8.016 (5.269, 11.322) |
| AAPC on ASMR, 1990-2019 | -0.357 (-1.532, 0.833)* | -1.984 (-3.532, -0.411) | -0.281 (-1.600, 1.055)* | -0.343 (-1.208, 0.530)* | -1.537 (-2.672, -0.388) |
| Kyrgyzstan |  |  |  |  |  |
| ASMR in 2019 (per 100,000) | 2.962 (1.660, 4.607) | 62.915 (35.654, 97.870) | 30.753 (17.588, 47.097) | 10.614 (6.010, 17.222) | 1.466 (0.797, 2.310) |
| AAPC on ASMR, 1990-2019 | -1.295 (-1.685, -0.903) | 2.395 (1.752, 3.042) | 0.047 (-0.484, 0.580)* | -1.727 (-2.516, -0.931) | 2.339 (1.848, 2.831) |
| Lao People's Democratic Republic |  |  |  |  |  |
| ASMR in 2019 (per 100,000) | 2.758 (1.205, 5.029) | 15.575 (7.325, 27.034) | 20.551 (9.639, 35.349) | 8.417 (4.571, 13.908) | 2.893 (1.227, 5.439) |
| AAPC on ASMR, 1990-2019 | 2.348 (2.241, 2.456) | 2.403 (2.300, 2.506) | 2.278 (2.127, 2.429) | 0.481 (0.373, 0.590) | 2.870 (2.715, 3.026) |
| Latvia |  |  |  |  |  |
| ASMR in 2019 (per 100,000) | 3.653 (2.144, 5.757) | 25.733 (14.285, 41.550) | 14.377 (8.492, 22.750) | 1.171 (0.666, 1.891) | 1.476 (0.808, 2.363) |
| AAPC on ASMR, 1990-2019 | -2.849 (-4.243, -1.435) | -3.388 (-4.531, -2.231) | -3.848 (-4.980, -2.703) | -3.859 (-4.898, -2.809) | 0.578 (-1.413, 2.608)* |
| Lebanon |  |  |  |  |  |
| ASMR in 2019 (per 100,000) | 9.577 (5.934, 14.668) | 76.650 (47.080, 109.446) | 11.322 (6.129, 17.505) | 5.892 (3.364, 9.355) | 6.000 (3.425, 9.336) |
| AAPC on ASMR, 1990-2019 | 1.542 (1.393, 1.691) | -0.629 (-0.867, -0.391) | -0.978 (-1.264, -0.691) | -0.354 (-0.627, -0.080) | 0.006 (-0.179, 0.191)* |
| Lesotho |  |  |  |  |  |
| ASMR in 2019 (per 100,000) | 3.497 (1.546, 6.399) | 19.980 (9.562, 35.136) | 33.004 (16.189, 56.600) | 16.910 (9.086, 28.678) | 15.531 (7.250, 27.622) |
| AAPC on ASMR, 1990-2019 | 3.739 (3.656, 3.822) | 3.841 (3.595, 4.088) | 3.224 (2.981, 3.467) | 1.331 (1.144, 1.519) | 5.516 (5.213, 5.820) |
| Liberia |  |  |  |  |  |
| ASMR in 2019 (per 100,000) | 1.404 (0.547, 2.869) | 13.477 (5.575, 26.358) | 13.284 (5.588, 25.552) | 4.294 (1.976, 7.913) | 3.256 (1.223, 6.720) |
| AAPC on ASMR, 1990-2019 | 1.991 (0.900, 3.095) | 1.707 (0.815, 2.606) | 1.504 (0.899, 2.112) | 1.421 (1.093, 1.750) | 2.875 (2.404, 3.350) |
| Libya |  |  |  |  |  |
| ASMR in 2019 (per 100,000) | 7.986 (4.576, 12.474) | 65.809 (40.103, 99.228) | 29.806 (17.562, 46.546) | 7.741 (4.199, 12.583) | 7.354 (4.335, 11.369) |
| AAPC on ASMR, 1990-2019 | 0.521 (0.063, 0.981) | 0.569 (0.043, 1.098) | 0.370 (-0.220, 0.964)* | 0.397 (-0.099, 0.895)* | 2.000 (1.410, 2.594) |
| Lithuania |  |  |  |  |  |
| ASMR in 2019 (per 100,000) | 2.891 (1.626, 4.626) | 23.003 (12.029, 38.718) | 8.506 (4.666, 14.047) | 1.245 (0.661, 2.059) | 0.621 (0.324, 1.029) |
| AAPC on ASMR, 1990-2019 | -3.316 (-4.411, -2.208) | -3.485 (-4.388, -2.573) | -3.029 (-3.980, -2.068) | -5.357 (-6.138, -4.571) | -1.246 (-2.033, -0.454) |
| Luxembourg |  |  |  |  |  |
| ASMR in 2019 (per 100,000) | 3.394 (1.948, 5.304) | 5.255 (2.722, 8.898) | 2.940 (1.588, 4.933) | 1.828 (0.992, 2.916) | 0.946 (0.495, 1.523) |
| AAPC on ASMR, 1990-2019 | -3.237 (-3.491, -2.982) | -5.796 (-5.970, -5.621) | -6.607 (-6.770, -6.443) | -3.561 (-3.849, -3.273) | -3.529 (-4.007, -3.049) |
| Madagascar |  |  |  |  |  |
| ASMR in 2019 (per 100,000) | 0.456 (0.174, 0.933) | 7.554 (3.002, 15.408) | 12.060 (4.976, 24.007) | 4.412 (2.091, 7.915) | 1.369 (0.530, 2.835) |
| AAPC on ASMR, 1990-2019 | 2.211 (1.639, 2.787) | 2.526 (1.952, 3.105) | 2.399 (1.809, 2.992) | 1.709 (1.014, 2.410) | 2.518 (1.934, 3.105) |
| Malawi |  |  |  |  |  |
| ASMR in 2019 (per 100,000) | 0.437 (0.164, 0.904) | 5.327 (2.001, 11.128) | 7.668 (2.997, 15.488) | 2.764 (1.268, 5.087) | 1.777 (0.636, 3.713) |
| AAPC on ASMR, 1990-2019 | 2.781 (2.442, 3.121) | 2.264 (1.876, 2.654) | 1.912 (1.666, 2.159) | 1.311 (1.088, 1.534) | 2.369 (2.107, 2.633) |
| Malaysia |  |  |  |  |  |
| ASMR in 2019 (per 100,000) | 4.385 (2.499, 6.880) | 30.281 (16.952, 48.105) | 19.186 (11.166, 29.784) | 5.842 (3.287, 9.340) | 3.518 (1.985, 5.605) |
| AAPC on ASMR, 1990-2019 | -0.759 (-0.929, -0.589) | -1.701 (-2.063, -1.338) | -3.401 (-3.709, -3.091) | -3.360 (-4.543, -2.163) | -2.960 (-3.606, -2.308) |
| Maldives |  |  |  |  |  |
| ASMR in 2019 (per 100,000) | 1.245 (0.669, 2.050) | 13.089 (7.053, 21.799) | 7.376 (4.094, 11.917) | 5.043 (2.885, 8.071) | 2.653 (1.306, 4.568) |
| AAPC on ASMR, 1990-2019 | 0.235 (-0.266, 0.738)* | -1.116 (-1.679, -0.550) | -1.632 (-2.432, -0.824) | -3.453 (-4.117, -2.784) | 1.238 (0.382, 2.101) |
| Mali |  |  |  |  |  |
| ASMR in 2019 (per 100,000) | 0.777 (0.218, 1.788) | 9.359 (2.835, 20.814) | 10.817 (3.448, 23.391) | 6.264 (2.252, 13.024) | 2.290 (0.643, 5.376) |
| AAPC on ASMR, 1990-2019 | 2.784 (2.623, 2.945) | 1.840 (1.665, 2.016) | 1.602 (1.482, 1.722) | 1.712 (1.593, 1.831) | 2.867 (2.712, 3.023) |
| Malta |  |  |  |  |  |
| ASMR in 2019 (per 100,000) | 3.409 (2.111, 5.030) | 12.721 (7.281, 19.903) | 4.517 (2.675, 7.123) | 1.789 (1.055, 2.733) | 2.594 (1.476, 3.919) |
| AAPC on ASMR, 1990-2019 | -1.777 (-2.207, -1.346) | -3.868 (-4.177, -3.558) | -4.450 (-4.702, -4.197) | -2.957 (-3.370, -2.542) | -2.839 (-3.584, -2.089) |
| Marshall Islands |  |  |  |  |  |
| ASMR in 2019 (per 100,000) | 2.784 (0.756, 6.816) | 23.898 (7.502, 54.171) | 18.306 (6.029, 40.292) | 7.298 (2.559, 15.922) | 8.432 (2.049, 21.391) |
| AAPC on ASMR, 1990-2019 | 2.492 (2.006, 2.980) | 2.514 (2.239, 2.790) | 1.775 (1.535, 2.016) | -0.124 (-0.328, 0.081)* | 3.844 (3.512, 4.177) |
| Mauritania |  |  |  |  |  |
| ASMR in 2019 (per 100,000) | 3.828 (1.788, 6.842) | 28.835 (15.128, 46.141) | 27.141 (13.857, 44.119) | 8.960 (4.705, 14.957) | 7.993 (3.957, 13.742) |
| AAPC on ASMR, 1990-2019 | 2.946 (2.585, 3.307) | 1.501 (1.069, 1.934) | 1.134 (0.757, 1.513) | 0.414 (0.125, 0.704) | 2.985 (2.820, 3.151) |
| Mauritius |  |  |  |  |  |
| ASMR in 2019 (per 100,000) | 2.299 (1.040, 3.928) | 18.701 (8.102, 32.553) | 12.640 (5.644, 21.692) | 3.392 (1.358, 6.089) | 25.842 (12.290, 40.927) |
| AAPC on ASMR, 1990-2019 | -0.179 (-0.657, 0.302)* | -2.729 (-3.321, -2.133) | -2.695 (-2.988, -2.401) | -1.526 (-2.172, -0.876) | 4.043 (3.417, 4.673) |
| Mexico |  |  |  |  |  |
| ASMR in 2019 (per 100,000) | 2.245 (1.530, 3.117) | 20.188 (12.866, 29.132) | 8.181 (5.550, 11.340) | 7.548 (4.857, 10.558) | 16.609 (11.065, 23.005) |
| AAPC on ASMR, 1990-2019 | -1.818 (-2.267, -1.366) | -0.699 (-1.023, -0.375) | -2.090 (-2.388, -1.791) | -1.662 (-2.002, -1.320) | 0.206 (-0.152, 0.565)* |
| Micronesia (Federated States of) |  |  |  |  |  |
| ASMR in 2019 (per 100,000) | 3.444 (0.835, 9.020) | 30.416 (7.647, 74.353) | 22.690 (6.069, 54.763) | 8.831 (2.559, 20.930) | 16.327 (3.555, 42.312) |
| AAPC on ASMR, 1990-2019 | 2.390 (2.149, 2.632) | 2.340 (1.993, 2.689) | 1.465 (1.237, 1.693) | -0.277 (-0.691, 0.138)* | 5.014 (4.842, 5.187) |
| Monaco |  |  |  |  |  |
| ASMR in 2019 (per 100,000) | 9.213 (4.770, 15.097) | 8.117 (3.787, 14.311) | 4.343 (2.101, 7.536) | 1.784 (0.882, 3.070) | 0.606 (0.293, 1.022) |
| AAPC on ASMR, 1990-2019 | 2.176 (1.775, 2.579) | -1.557 (-2.012, -1.100) | -2.013 (-2.403, -1.622) | 0.484 (0.097, 0.872) | 0.860 (0.390, 1.332) |
| Mongolia |  |  |  |  |  |
| ASMR in 2019 (per 100,000) | 10.753 (6.440, 16.665) | 84.903 (53.247, 124.161) | 80.469 (49.941, 119.700) | 7.552 (4.532, 11.853) | 1.366 (0.752, 2.241) |
| AAPC on ASMR, 1990-2019 | 1.818 (1.148, 2.493) | 1.938 (1.497, 2.380) | 3.400 (3.101, 3.699) | 0.122 (-0.323, 0.569)* | 3.782 (3.514, 4.050) |
| Montenegro |  |  |  |  |  |
| ASMR in 2019 (per 100,000) | 13.625 (8.761, 19.534) | 36.394 (23.438, 52.529) | 49.113 (34.011, 67.391) | 2.179 (1.320, 3.208) | 3.757 (2.221, 5.669) |
| AAPC on ASMR, 1990-2019 | -0.188 (-0.443, 0.068)* | -0.584 (-0.977, -0.190) | -0.785 (-1.157, -0.411) | -1.042 (-1.396, -0.687) | 0.585 (0.294, 0.876) |
| Morocco |  |  |  |  |  |
| ASMR in 2019 (per 100,000) | 6.444 (3.709, 9.954) | 95.231 (62.020, 133.875) | 44.706 (29.051, 64.708) | 9.736 (5.817, 14.923) | 8.383 (5.091, 12.755) |
| AAPC on ASMR, 1990-2019 | 3.188 (3.030, 3.347) | 2.502 (2.073, 2.932) | 2.528 (2.362, 2.695) | 2.132 (1.868, 2.397) | 5.089 (4.832, 5.346) |
| Mozambique |  |  |  |  |  |
| ASMR in 2019 (per 100,000) | 0.452 (0.153, 0.999) | 4.474 (1.584, 9.698) | 9.357 (3.485, 19.984) | 2.241 (0.982, 4.297) | 1.611 (0.519, 3.552) |
| AAPC on ASMR, 1990-2019 | 4.522 (4.299, 4.746) | 3.929 (3.733, 4.126) | 3.492 (3.257, 3.728) | 2.556 (2.415, 2.696) | 3.939 (3.688, 4.192) |
| Myanmar |  |  |  |  |  |
| ASMR in 2019 (per 100,000) | 4.522 (2.287, 7.781) | 17.113 (9.687, 26.402) | 42.067 (24.626, 63.390) | 21.327 (13.452, 30.326) | 7.152 (3.794, 11.525) |
| AAPC on ASMR, 1990-2019 | 2.638 (2.506, 2.769) | 1.781 (1.703, 1.860) | 1.987 (1.898, 2.077) | 0.873 (0.751, 0.994) | 3.200 (3.048, 3.353) |
| Namibia |  |  |  |  |  |
| ASMR in 2019 (per 100,000) | 1.869 (0.974, 3.072) | 23.180 (12.298, 37.787) | 30.073 (16.199, 47.791) | 11.717 (6.206, 19.812) | 12.229 (6.000, 20.392) |
| AAPC on ASMR, 1990-2019 | 2.889 (2.665, 3.113) | 2.145 (1.866, 2.426) | 1.473 (1.216, 1.730) | 0.201 (-0.003, 0.405)* | 3.355 (3.050, 3.662) |
| Nauru |  |  |  |  |  |
| ASMR in 2019 (per 100,000) | 1.956 (0.419, 5.271) | 18.821 (4.449, 48.558) | 12.212 (3.120, 30.334) | 3.435 (0.815, 8.375) | 9.728 (1.720, 27.615) |
| AAPC on ASMR, 1990-2019 | -0.443 (-1.015, 0.132)* | -0.014 (-0.436, 0.409)* | -0.889 (-1.220, -0.557) | -2.338 (-2.550, -2.125) | 1.972 (1.578, 2.368) |
| Nepal |  |  |  |  |  |
| ASMR in 2019 (per 100,000) | 2.528 (1.224, 4.366) | 33.074 (17.712, 52.344) | 25.209 (13.756, 39.905) | 85.252 (52.286, 124.104) | 3.856 (1.844, 6.688) |
| AAPC on ASMR, 1990-2019 | 4.629 (4.322, 4.937) | 4.620 (4.294, 4.947) | 3.260 (3.039, 3.481) | 3.381 (3.192, 3.571) | 6.261 (6.039, 6.484) |
| Netherlands |  |  |  |  |  |
| ASMR in 2019 (per 100,000) | 5.705 (3.628, 8.313) | 5.988 (3.399, 9.469) | 4.141 (2.481, 6.511) | 3.594 (2.156, 5.343) | 1.731 (1.011, 2.631) |
| AAPC on ASMR, 1990-2019 | -2.545 (-2.809, -2.281) | -5.804 (-6.068, -5.538) | -4.273 (-4.503, -4.043) | -2.539 (-2.876, -2.200) | -3.569 (-3.928, -3.209) |
| New Zealand |  |  |  |  |  |
| ASMR in 2019 (per 100,000) | 1.070 (0.186, 2.316) | 2.692 (0.435, 6.311) | 1.118 (0.186, 2.604) | 0.910 (0.151, 2.015) | 0.500 (0.079, 1.136) |
| AAPC on ASMR, 1990-2019 | -2.387 (-2.763, -2.010) | -4.728 (-5.115, -4.341) | -4.039 (-4.353, -3.725) | -2.309 (-2.706, -1.911) | -1.957 (-2.242, -1.672) |
| Nicaragua |  |  |  |  |  |
| ASMR in 2019 (per 100,000) | 1.468 (0.719, 2.496) | 19.020 (9.692, 31.763) | 8.701 (4.506, 14.550) | 7.242 (4.159, 11.369) | 6.405 (3.061, 11.131) |
| AAPC on ASMR, 1990-2019 | 3.538 (2.953, 4.125) | 3.991 (3.138, 4.850) | 1.730 (1.386, 2.075) | 3.822 (2.630, 5.029) | 5.397 (4.151, 6.658) |
| Niger |  |  |  |  |  |
| ASMR in 2019 (per 100,000) | 0.859 (0.184, 2.391) | 8.430 (1.853, 22.459) | 9.621 (2.273, 25.083) | 4.992 (1.326, 12.674) | 1.736 (0.342, 4.935) |
| AAPC on ASMR, 1990-2019 | 1.924 (1.592, 2.257) | 1.117 (0.895, 1.339) | 1.028 (0.788, 1.268) | 0.882 (0.649, 1.115) | 2.223 (1.813, 2.634) |
| Nigeria |  |  |  |  |  |
| ASMR in 2019 (per 100,000) | 2.438 (1.339, 3.736) | 25.120 (13.952, 38.761) | 25.967 (14.953, 39.654) | 8.991 (5.497, 13.461) | 7.563 (4.284, 11.704) |
| AAPC on ASMR, 1990-2019 | 3.894 (3.683, 4.105) | 2.560 (2.220, 2.901) | 2.011 (1.562, 2.462) | 2.086 (1.939, 2.233) | 4.014 (3.819, 4.210) |
| Niue |  |  |  |  |  |
| ASMR in 2019 (per 100,000) | 2.010 (0.386, 5.015) | 13.756 (2.580, 34.273) | 7.973 (1.592, 19.751) | 2.571 (0.510, 6.252) | 10.413 (1.846, 26.454) |
| AAPC on ASMR, 1990-2019 | 0.596 (0.343, 0.851) | 0.167 (-0.084, 0.419)* | -1.051 (-1.286, -0.816) | -1.913 (-2.103, -1.722) | 2.846 (2.625, 3.067) |
| North Macedonia |  |  |  |  |  |
| ASMR in 2019 (per 100,000) | 12.965 (8.314, 18.885) | 57.357 (38.836, 80.575) | 85.045 (60.665, 114.285) | 7.829 (5.205, 11.458) | 10.991 (6.943, 16.117) |
| AAPC on ASMR, 1990-2019 | 0.895 (0.725, 1.065) | -0.655 (-0.890, -0.418) | -0.550 (-0.852, -0.247) | -1.999 (-2.202, -1.796) | 2.665 (2.338, 2.993) |
| Northern Mariana Islands |  |  |  |  |  |
| ASMR in 2019 (per 100,000) | 4.291 (2.037, 7.494) | 13.997 (6.273, 25.637) | 9.741 (4.486, 17.302) | 3.383 (1.545, 6.038) | 6.790 (3.020, 12.260) |
| AAPC on ASMR, 1990-2019 | -1.915 (-2.581, -1.244) | -0.390 (-1.137, 0.362)* | -2.665 (-3.585, -1.736) | -2.885 (-3.624, -2.139) | 0.036 (-1.849, 1.957)* |
| Norway |  |  |  |  |  |
| ASMR in 2019 (per 100,000) | 1.253 (0.429, 2.379) | 2.285 (0.730, 4.784) | 1.174 (0.398, 2.416) | 1.034 (0.341, 2.000) | 0.420 (0.135, 0.836) |
| AAPC on ASMR, 1990-2019 | -3.377 (-3.748, -3.005) | -7.568 (-7.851, -7.285) | -6.806 (-7.087, -6.525) | -1.814 (-2.401, -1.225) | -3.543 (-4.091, -2.993) |
| Oman |  |  |  |  |  |
| ASMR in 2019 (per 100,000) | 4.660 (2.891, 7.109) | 125.684 (86.357, 169.145) | 45.937 (29.811, 65.982) | 11.322 (6.555, 17.169) | 24.265 (15.752, 34.941) |
| AAPC on ASMR, 1990-2019 | 1.954 (1.665, 2.244) | 0.349 (-0.154, 0.855)* | 0.424 (0.010, 0.841) | -0.314 (-0.779, 0.154)* | 3.181 (2.406, 3.962) |
| Pakistan |  |  |  |  |  |
| ASMR in 2019 (per 100,000) | 5.499 (3.124, 8.518) | 55.466 (34.808, 81.215) | 37.136 (23.112, 54.690) | 35.359 (23.506, 50.120) | 11.483 (6.378, 17.693) |
| AAPC on ASMR, 1990-2019 | 4.207 (4.010, 4.404) | 4.776 (4.634, 4.918) | 3.495 (3.316, 3.674) | 2.048 (1.873, 2.223) | 6.656 (6.502, 6.810) |
| Palau |  |  |  |  |  |
| ASMR in 2019 (per 100,000) | 2.622 (0.011, 7.084) | 13.906 (0.052, 38.563) | 7.467 (0.029, 20.504) | 3.588 (0.016, 9.709) | 9.972 (0.031, 27.522) |
| AAPC on ASMR, 1990-2019 | 0.063 (-1.660, 1.817)* | -0.277 (-1.962, 1.437)* | -0.768 (-2.480, 0.974)* | -1.163 (-2.990, 0.698)* | 1.475 (-0.153, 3.130)* |
| Palestine |  |  |  |  |  |
| ASMR in 2019 (per 100,000) | 8.620 (5.652, 12.279) | 68.146 (43.929, 94.493) | 42.743 (28.329, 58.950) | 6.137 (3.661, 9.449) | 26.099 (17.174, 36.008) |
| AAPC on ASMR, 1990-2019 | 2.306 (2.016, 2.596) | 0.858 (0.555, 1.161) | 0.888 (0.465, 1.313) | -0.559 (-0.931, -0.185) | 3.301 (2.957, 3.645) |
| Panama |  |  |  |  |  |
| ASMR in 2019 (per 100,000) | 1.747 (0.940, 2.817) | 8.439 (4.365, 14.165) | 6.687 (3.489, 11.068) | 2.597 (1.316, 4.273) | 5.530 (2.921, 8.955) |
| AAPC on ASMR, 1990-2019 | -0.736 (-1.501, 0.036)* | -1.885 (-2.552, -1.213) | -1.849 (-2.416, -1.279) | -0.846 (-1.649, -0.035) | 2.963 (2.344, 3.586) |
| Papua New Guinea |  |  |  |  |  |
| ASMR in 2019 (per 100,000) | 1.441 (0.295, 4.078) | 9.889 (2.153, 27.005) | 8.999 (2.013, 24.843) | 13.614 (3.451, 35.911) | 4.633 (0.983, 12.996) |
| AAPC on ASMR, 1990-2019 | 2.038 (1.947, 2.128) | 2.250 (2.031, 2.470) | 1.424 (1.268, 1.581) | 0.734 (0.586, 0.882) | 2.552 (2.436, 2.668) |
| Paraguay |  |  |  |  |  |
| ASMR in 2019 (per 100,000) | 2.235 (1.189, 3.647) | 11.267 (6.202, 18.665) | 9.250 (5.166, 15.319) | 2.790 (1.566, 4.565) | 6.020 (2.923, 10.383) |
| AAPC on ASMR, 1990-2019 | 2.635 (2.101, 3.173) | 0.868 (0.045, 1.697) | -0.255 (-0.814, 0.308)* | 1.095 (0.276, 1.922) | 4.990 (4.445, 5.537) |
| Peru |  |  |  |  |  |
| ASMR in 2019 (per 100,000) | 3.389 (1.967, 5.234) | 12.877 (7.531, 19.998) | 8.840 (5.160, 14.121) | 3.124 (1.746, 5.112) | 4.321 (2.524, 6.784) |
| AAPC on ASMR, 1990-2019 | -0.112 (-0.849, 0.631)* | -1.594 (-2.333, -0.850) | -1.789 (-2.523, -1.050) | -0.545 (-1.193, 0.108)* | 2.415 (1.911, 2.921) |
| Philippines |  |  |  |  |  |
| ASMR in 2019 (per 100,000) | 3.555 (2.313, 5.154) | 26.432 (17.267, 38.014) | 21.251 (13.863, 30.160) | 6.777 (4.685, 9.827) | 6.347 (3.899, 9.581) |
| AAPC on ASMR, 1990-2019 | -1.176 (-1.450, -0.902) | 2.199 (1.801, 2.599) | 1.934 (1.519, 2.350) | -1.792 (-2.179, -1.403) | 0.886 (0.426, 1.349) |
| Poland |  |  |  |  |  |
| ASMR in 2019 (per 100,000) | 12.199 (8.563, 16.722) | 30.373 (20.016, 42.971) | 16.509 (11.781, 22.222) | 3.782 (2.609, 5.225) | 3.240 (2.148, 4.389) |
| AAPC on ASMR, 1990-2019 | -0.704 (-1.163, -0.243) | -3.816 (-4.175, -3.455) | -2.988 (-3.338, -2.637) | -3.088 (-3.315, -2.860) | -0.267 (-0.711, 0.179)* |
| Portugal |  |  |  |  |  |
| ASMR in 2019 (per 100,000) | 1.744 (0.898, 2.875) | 3.330 (1.577, 5.978) | 3.959 (1.969, 6.971) | 1.535 (0.753, 2.590) | 1.735 (0.826, 2.994) |
| AAPC on ASMR, 1990-2019 | -1.619 (-1.867, -1.371) | -5.065 (-5.415, -4.714) | -6.211 (-6.443, -5.979) | -3.031 (-3.359, -2.702) | -2.332 (-2.638, -2.024) |
| Puerto Rico |  |  |  |  |  |
| ASMR in 2019 (per 100,000) | 0.654 (0.129, 1.342) | 3.223 (0.629, 7.071) | 1.215 (0.239, 2.672) | 0.892 (0.180, 1.851) | 3.438 (0.650, 7.210) |
| AAPC on ASMR, 1990-2019 | -2.183 (-2.622, -1.742) | -4.028 (-4.609, -3.444) | -3.084 (-3.573, -2.592) | -1.613 (-2.055, -1.168) | -0.918 (-1.372, -0.462) |
| Qatar |  |  |  |  |  |
| ASMR in 2019 (per 100,000) | 11.503 (6.913, 17.846) | 109.896 (77.608, 148.659) | 27.991 (18.337, 41.410) | 14.056 (8.920, 22.132) | 53.715 (34.668, 78.986) |
| AAPC on ASMR, 1990-2019 | 0.328 (-0.521, 1.185)* | -2.119 (-3.736, -0.474) | -1.700 (-3.579, 0.217)* | -1.024 (-2.649, 0.628)* | 0.395 (-1.013, 1.822)* |
| Republic of Korea |  |  |  |  |  |
| ASMR in 2019 (per 100,000) | 8.832 (6.036, 12.061) | 9.257 (5.966, 13.144) | 13.542 (9.218, 19.105) | 4.452 (2.823, 6.442) | 5.449 (3.502, 7.541) |
| AAPC on ASMR, 1990-2019 | 0.699 (0.389, 1.011) | -4.842 (-5.219, -4.464) | -5.304 (-5.491, -5.116) | -1.734 (-2.024, -1.443) | -0.161 (-0.613, 0.293)* |
| Republic of Moldova |  |  |  |  |  |
| ASMR in 2019 (per 100,000) | 3.023 (1.552, 4.851) | 39.549 (20.425, 65.113) | 17.334 (9.179, 27.779) | 2.597 (1.301, 4.399) | 0.973 (0.514, 1.540) |
| AAPC on ASMR, 1990-2019 | -2.144 (-3.537, -0.731) | -1.717 (-3.103, -0.311) | -2.394 (-3.914, -0.850) | -4.565 (-6.174, -2.928) | -0.364 (-1.704, 0.993)* |
| Romania |  |  |  |  |  |
| ASMR in 2019 (per 100,000) | 6.200 (4.078, 8.917) | 30.180 (18.669, 44.799) | 24.736 (16.292, 35.388) | 3.557 (2.265, 5.269) | 1.421 (0.889, 2.084) |
| AAPC on ASMR, 1990-2019 | 0.343 (-0.368, 1.058)* | -2.138 (-2.631, -1.643) | -2.059 (-2.457, -1.659) | -4.403 (-5.019, -3.783) | 0.195 (-0.273, 0.665)* |
| Russian Federation |  |  |  |  |  |
| ASMR in 2019 (per 100,000) | 3.149 (1.630, 4.958) | 30.409 (14.881, 50.779) | 18.641 (9.647, 30.254) | 2.011 (0.984, 3.312) | 1.346 (0.669, 2.140) |
| AAPC on ASMR, 1990-2019 | -2.799 (-4.267, -1.307) | -2.335 (-3.629, -1.024) | -3.014 (-4.189, -1.825) | -4.008 (-5.027, -2.979) | 1.054 (0.099, 2.019) |
| Rwanda |  |  |  |  |  |
| ASMR in 2019 (per 100,000) | 1.164 (0.406, 2.489) | 9.040 (3.426, 18.340) | 15.198 (5.870, 30.367) | 7.282 (3.234, 13.975) | 3.326 (1.201, 6.831) |
| AAPC on ASMR, 1990-2019 | 2.209 (1.918, 2.500) | 1.296 (1.006, 1.586) | 0.331 (0.052, 0.612) | 0.064 (-0.190, 0.318)* | 1.542 (1.311, 1.772) |
| Saint Kitts and Nevis |  |  |  |  |  |
| ASMR in 2019 (per 100,000) | 1.074 (0.429, 1.960) | 9.209 (3.367, 17.455) | 11.837 (4.580, 22.215) | 1.333 (0.555, 2.401) | 7.107 (2.712, 12.941) |
| AAPC on ASMR, 1990-2019 | -0.489 (-1.480, 0.513)* | -2.779 (-3.376, -2.179) | -2.644 (-3.322, -1.962) | -0.594 (-1.202, 0.018)* | 0.280 (-0.258, 0.822)* |
| Saint Lucia |  |  |  |  |  |
| ASMR in 2019 (per 100,000) | 3.207 (1.278, 5.781) | 15.188 (5.930, 26.981) | 21.807 (8.396, 40.140) | 5.591 (2.061, 10.452) | 18.042 (8.052, 28.856) |
| AAPC on ASMR, 1990-2019 | 0.792 (0.499, 1.085) | -1.404 (-1.763, -1.044) | -0.961 (-1.399, -0.521) | 0.083 (-0.305, 0.473)* | 1.200 (0.840, 1.562) |
| Saint Vincent and the Grenadines |  |  |  |  |  |
| ASMR in 2019 (per 100,000) | 2.669 (0.962, 4.923) | 29.161 (10.402, 52.947) | 22.619 (8.026, 42.643) | 2.882 (0.968, 5.676) | 23.192 (9.570, 37.359) |
| AAPC on ASMR, 1990-2019 | 1.590 (1.035, 2.147) | 0.167 (-0.181, 0.517)* | 0.334 (-0.118, 0.787)* | 1.114 (0.598, 1.632) | 2.028 (1.551, 2.507) |
| Samoa |  |  |  |  |  |
| ASMR in 2019 (per 100,000) | 1.109 (0.306, 2.740) | 19.494 (5.216, 45.783) | 14.416 (3.865, 33.774) | 6.563 (1.869, 15.823) | 6.936 (1.705, 17.229) |
| AAPC on ASMR, 1990-2019 | 0.650 (0.526, 0.774) | 0.680 (0.629, 0.731) | -0.114 (-0.170, -0.058) | -1.116 (-1.175, -1.057) | 1.696 (1.616, 1.777) |
| San Marino |  |  |  |  |  |
| ASMR in 2019 (per 100,000) | 3.680 (1.328, 7.441) | 4.387 (1.468, 9.118) | 3.276 (1.140, 6.583) | 1.152 (0.383, 2.331) | 0.918 (0.309, 1.800) |
| AAPC on ASMR, 1990-2019 | -1.211 (-1.394, -1.027) | -2.795 (-2.957, -2.633) | -2.748 (-2.874, -2.623) | -1.685 (-1.859, -1.510) | -1.454 (-1.600, -1.307) |
| Sao Tome and Principe |  |  |  |  |  |
| ASMR in 2019 (per 100,000) | 4.086 (1.858, 7.342) | 25.069 (11.777, 43.562) | 24.331 (11.299, 42.953) | 15.713 (7.739, 26.828) | 2.400 (1.062, 4.330) |
| AAPC on ASMR, 1990-2019 | 4.577 (4.259, 4.896) | 4.508 (4.051, 4.968) | 3.713 (3.232, 4.195) | 2.581 (2.159, 3.005) | 4.965 (4.572, 5.359) |
| Saudi Arabia |  |  |  |  |  |
| ASMR in 2019 (per 100,000) | 4.676 (2.995, 6.818) | 99.896 (70.763, 132.698) | 56.637 (37.900, 77.502) | 12.022 (7.635, 17.719) | 8.391 (5.354, 12.351) |
| AAPC on ASMR, 1990-2019 | 2.246 (1.940, 2.553) | 1.785 (1.461, 2.111) | 0.967 (0.731, 1.204) | -0.345 (-0.551, -0.139) | 1.394 (1.192, 1.596) |
| Senegal |  |  |  |  |  |
| ASMR in 2019 (per 100,000) | 2.480 (1.043, 4.832) | 17.914 (7.737, 33.342) | 17.911 (7.888, 32.807) | 7.996 (3.964, 14.140) | 4.854 (1.990, 9.525) |
| AAPC on ASMR, 1990-2019 | 2.565 (1.957, 3.177) | 1.638 (1.256, 2.022) | 1.652 (1.254, 2.052) | 0.546 (0.122, 0.971) | 3.163 (2.803, 3.524) |
| Serbia |  |  |  |  |  |
| ASMR in 2019 (per 100,000) | 13.667 (8.979, 19.579) | 48.062 (32.001, 68.423) | 47.192 (33.183, 64.212) | 6.798 (4.518, 9.718) | 6.668 (4.253, 9.688) |
| AAPC on ASMR, 1990-2019 | 0.629 (0.308, 0.950) | -0.889 (-1.347, -0.428) | -1.482 (-1.802, -1.161) | -1.156 (-1.535, -0.776) | 1.482 (1.162, 1.802) |
| Seychelles |  |  |  |  |  |
| ASMR in 2019 (per 100,000) | 3.675 (1.785, 6.295) | 21.040 (9.567, 36.041) | 14.486 (6.732, 24.594) | 4.706 (2.040, 8.175) | 6.198 (3.011, 10.010) |
| AAPC on ASMR, 1990-2019 | 0.166 (-0.178, 0.511)* | -0.750 (-1.016, -0.484) | -1.374 (-1.762, -0.986) | -0.738 (-1.180, -0.294) | 2.553 (2.244, 2.863) |
| Sierra Leone |  |  |  |  |  |
| ASMR in 2019 (per 100,000) | 1.401 (0.520, 2.972) | 13.475 (5.148, 27.986) | 13.887 (5.233, 28.688) | 5.739 (2.490, 11.021) | 2.633 (0.899, 5.633) |
| AAPC on ASMR, 1990-2019 | 2.259 (1.774, 2.746) | 1.689 (1.458, 1.921) | 1.771 (1.608, 1.935) | 0.751 (0.232, 1.273) | 3.048 (2.783, 3.315) |
| Singapore |  |  |  |  |  |
| ASMR in 2019 (per 100,000) | 5.042 (3.006, 7.516) | 11.409 (6.243, 17.462) | 4.908 (2.750, 7.624) | 2.076 (1.109, 3.359) | 0.703 (0.397, 1.046) |
| AAPC on ASMR, 1990-2019 | -2.508 (-2.869, -2.146) | -3.985 (-4.263, -3.705) | -5.293 (-5.787, -4.796) | -6.040 (-6.504, -5.573) | -6.716 (-7.810, -5.608) |
| Slovakia |  |  |  |  |  |
| ASMR in 2019 (per 100,000) | 6.452 (4.065, 9.540) | 39.120 (23.673, 59.161) | 14.735 (9.423, 21.737) | 2.499 (1.541, 3.826) | 2.414 (1.422, 3.601) |
| AAPC on ASMR, 1990-2019 | -2.319 (-2.778, -1.858) | -3.278 (-3.685, -2.869) | -3.483 (-3.834, -3.130) | -2.397 (-2.831, -1.962) | -2.107 (-2.341, -1.872) |
| Slovenia |  |  |  |  |  |
| ASMR in 2019 (per 100,000) | 6.570 (4.139, 9.804) | 10.911 (6.365, 16.933) | 7.721 (4.705, 11.708) | 2.350 (1.431, 3.525) | 1.899 (1.131, 2.903) |
| AAPC on ASMR, 1990-2019 | -1.808 (-2.037, -1.579) | -4.185 (-4.630, -3.739) | -5.109 (-5.500, -4.716) | -4.152 (-4.554, -3.748) | -1.723 (-2.529, -0.910) |
| Solomon Islands |  |  |  |  |  |
| ASMR in 2019 (per 100,000) | 1.828 (0.393, 5.457) | 22.188 (5.738, 58.235) | 17.545 (4.490, 46.427) | 8.237 (2.357, 20.148) | 4.743 (1.167, 12.900) |
| AAPC on ASMR, 1990-2019 | 3.263 (3.023, 3.504) | 3.096 (2.782, 3.412) | 3.016 (2.758, 3.274) | 2.057 (1.845, 2.270) | 4.882 (4.601, 5.164) |
| Somalia |  |  |  |  |  |
| ASMR in 2019 (per 100,000) | 0.147 (0.022, 0.486) | 2.642 (0.448, 8.142) | 3.606 (0.656, 10.813) | 1.828 (0.365, 5.349) | 0.696 (0.105, 2.111) |
| AAPC on ASMR, 1990-2019 | 0.400 (-0.111, 0.914)* | 1.205 (0.627, 1.787) | 0.370 (-0.115, 0.856)* | 0.060 (-0.464, 0.588)* | 1.002 (0.496, 1.512) |
| South Africa |  |  |  |  |  |
| ASMR in 2019 (per 100,000) | 5.941 (4.062, 8.394) | 22.193 (14.925, 30.294) | 24.419 (17.335, 32.378) | 10.237 (6.987, 14.057) | 20.637 (13.965, 27.600) |
| AAPC on ASMR, 1990-2019 | 0.031 (-0.391, 0.455)* | 0.486 (-0.340, 1.319)* | 0.409 (-0.431, 1.255)* | -0.249 (-0.867, 0.372)* | 2.772 (2.108, 3.441) |
| South Sudan |  |  |  |  |  |
| ASMR in 2019 (per 100,000) | 1.114 (0.374, 2.334) | 8.001 (2.913, 16.191) | 11.171 (4.069, 22.181) | 5.081 (2.198, 9.752) | 2.661 (0.957, 5.480) |
| AAPC on ASMR, 1990-2019 | 1.619 (1.429, 1.810) | 1.698 (1.536, 1.861) | 0.852 (0.669, 1.035) | 0.541 (0.373, 0.710) | 1.880 (1.648, 2.113) |
| Spain |  |  |  |  |  |
| ASMR in 2019 (per 100,000) | 2.961 (1.745, 4.548) | 4.288 (2.262, 7.186) | 2.692 (1.497, 4.457) | 2.439 (1.348, 3.909) | 1.231 (0.659, 1.973) |
| AAPC on ASMR, 1990-2019 | -2.009 (-2.407, -1.610) | -4.487 (-4.874, -4.098) | -5.386 (-5.854, -4.916) | -2.968 (-3.421, -2.514) | -3.548 (-3.965, -3.128) |
| Sri Lanka |  |  |  |  |  |
| ASMR in 2019 (per 100,000) | 2.059 (1.101, 3.420) | 20.307 (11.411, 32.172) | 14.111 (8.076, 22.163) | 5.368 (2.770, 9.099) | 11.334 (5.937, 18.648) |
| AAPC on ASMR, 1990-2019 | 2.186 (1.662, 2.713) | -0.071 (-0.855, 0.719)* | -0.438 (-1.114, 0.242)* | -0.132 (-0.646, 0.384)* | 5.289 (4.711, 5.871) |
| Sudan |  |  |  |  |  |
| ASMR in 2019 (per 100,000) | 2.945 (1.317, 5.708) | 81.070 (44.187, 128.931) | 43.366 (22.168, 73.375) | 12.950 (6.369, 22.132) | 4.049 (1.961, 7.066) |
| AAPC on ASMR, 1990-2019 | 5.202 (5.106, 5.298) | 3.990 (3.900, 4.080) | 3.933 (3.811, 4.055) | 3.008 (2.870, 3.146) | 6.677 (6.506, 6.847) |
| Suriname |  |  |  |  |  |
| ASMR in 2019 (per 100,000) | 3.817 (1.564, 6.988) | 25.629 (10.677, 45.758) | 27.486 (11.694, 49.392) | 3.894 (1.534, 7.343) | 11.924 (5.456, 19.803) |
| AAPC on ASMR, 1990-2019 | 0.805 (-0.059, 1.677)* | -1.205 (-2.187, -0.213) | -0.171 (-0.820, 0.482)* | -0.801 (-1.930, 0.342)* | 2.040 (1.173, 2.914) |
| Sweden |  |  |  |  |  |
| ASMR in 2019 (per 100,000) | 0.708 (0.168, 1.543) | 2.132 (0.491, 5.029) | 0.916 (0.221, 2.133) | 0.464 (0.110, 1.017) | 0.426 (0.098, 0.971) |
| AAPC on ASMR, 1990-2019 | -3.905 (-4.048, -3.762) | -7.141 (-7.340, -6.941) | -6.219 (-6.465, -5.973) | -2.857 (-3.037, -2.676) | -3.686 (-4.078, -3.292) |
| Switzerland |  |  |  |  |  |
| ASMR in 2019 (per 100,000) | 2.524 (1.472, 3.854) | 4.970 (2.586, 8.327) | 1.987 (1.082, 3.329) | 1.287 (0.721, 2.024) | 1.019 (0.547, 1.648) |
| AAPC on ASMR, 1990-2019 | -4.127 (-4.410, -3.843) | -5.668 (-5.992, -5.342) | -6.065 (-6.301, -5.829) | -3.830 (-4.078, -3.581) | -4.324 (-4.876, -3.768) |
| Syrian Arab Republic |  |  |  |  |  |
| ASMR in 2019 (per 100,000) | 4.212 (2.590, 6.498) | 116.801 (74.182, 170.645) | 36.088 (23.080, 52.543) | 7.708 (4.423, 12.946) | 6.123 (3.823, 9.286) |
| AAPC on ASMR, 1990-2019 | 0.606 (0.152, 1.062) | -0.080 (-0.526, 0.369)* | -1.059 (-1.456, -0.660) | -0.116 (-0.699, 0.471)* | 0.055 (-0.343, 0.454)* |
| Taiwan (Province of China) |  |  |  |  |  |
| ASMR in 2019 (per 100,000) | 8.843 (5.837, 12.723) | 10.580 (6.666, 15.598) | 11.238 (7.243, 16.508) | 5.994 (3.882, 8.747) | 8.763 (5.617, 12.643) |
| AAPC on ASMR, 1990-2019 | 1.232 (0.598, 1.870) | -1.719 (-1.952, -1.485) | -3.595 (-3.867, -3.322) | -0.717 (-1.057, -0.376) | 0.610 (0.235, 0.987) |
| Tajikistan |  |  |  |  |  |
| ASMR in 2019 (per 100,000) | 3.823 (1.841, 6.470) | 112.351 (55.508, 181.452) | 56.680 (28.225, 92.952) | 12.145 (5.528, 23.931) | 8.151 (3.996, 13.243) |
| AAPC on ASMR, 1990-2019 | 0.771 (0.214, 1.330) | 4.393 (4.080, 4.708) | 3.387 (3.010, 3.766) | 1.218 (0.884, 1.554) | 6.492 (5.803, 7.185) |
| Thailand |  |  |  |  |  |
| ASMR in 2019 (per 100,000) | 6.951 (4.379, 10.385) | 14.400 (8.801, 22.200) | 17.441 (11.031, 26.217) | 6.379 (3.957, 9.724) | 5.546 (3.357, 8.472) |
| AAPC on ASMR, 1990-2019 | -0.101 (-0.604, 0.405)* | -0.914 (-1.339, -0.488) | -1.603 (-2.036, -1.169) | -3.480 (-3.856, -3.103) | 0.826 (0.169, 1.488) |
| Timor-Leste |  |  |  |  |  |
| ASMR in 2019 (per 100,000) | 2.056 (0.768, 4.154) | 12.965 (4.976, 24.893) | 17.146 (6.572, 33.115) | 7.432 (3.391, 14.296) | 1.698 (0.618, 3.549) |
| AAPC on ASMR, 1990-2019 | 4.417 (4.173, 4.662) | 5.128 (4.930, 5.326) | 4.593 (4.411, 4.774) | 2.496 (2.210, 2.783) | 4.506 (4.135, 4.878) |
| Togo |  |  |  |  |  |
| ASMR in 2019 (per 100,000) | 1.974 (0.820, 3.710) | 18.687 (8.289, 33.291) | 18.652 (8.462, 33.194) | 6.750 (3.314, 11.763) | 3.681 (1.547, 6.999) |
| AAPC on ASMR, 1990-2019 | 2.521 (2.197, 2.846) | 2.039 (1.656, 2.423) | 1.872 (1.490, 2.256) | 0.637 (0.289, 0.986) | 3.110 (2.742, 3.479) |
| Tokelau |  |  |  |  |  |
| ASMR in 2019 (per 100,000) | 1.393 (0.006, 4.321) | 10.479 (0.045, 31.843) | 6.640 (0.035, 20.230) | 2.167 (0.011, 6.511) | 7.608 (0.021, 22.103) |
| AAPC on ASMR, 1990-2019 | -0.300 (-0.764, 0.166)* | -0.759 (-1.117, -0.399) | -1.934 (-2.352, -1.514) | -2.839 (-3.318, -2.358) | 0.508 (-0.041, 1.059)* |
| Tonga |  |  |  |  |  |
| ASMR in 2019 (per 100,000) | 3.344 (0.990, 7.828) | 14.257 (4.298, 32.753) | 8.998 (2.757, 20.443) | 4.861 (1.485, 11.523) | 11.220 (2.945, 26.458) |
| AAPC on ASMR, 1990-2019 | 1.523 (1.151, 1.897) | 1.542 (1.078, 2.009) | 1.109 (0.587, 1.634) | -0.403 (-0.925, 0.123)* | 3.306 (2.886, 3.728) |
| Trinidad and Tobago |  |  |  |  |  |
| ASMR in 2019 (per 100,000) | 2.743 (0.938, 5.394) | 30.623 (10.276, 57.659) | 18.416 (5.862, 36.363) | 2.713 (0.771, 5.689) | 30.331 (12.341, 49.965) |
| AAPC on ASMR, 1990-2019 | -0.324 (-0.739, 0.094)* | -1.998 (-2.418, -1.577) | -2.189 (-2.780, -1.594) | -1.074 (-1.542, -0.604) | -0.458 (-0.724, -0.191) |
| Tunisia |  |  |  |  |  |
| ASMR in 2019 (per 100,000) | 7.222 (4.092, 11.800) | 60.931 (35.857, 92.533) | 27.786 (16.455, 42.441) | 6.124 (3.317, 10.131) | 5.649 (3.320, 8.870) |
| AAPC on ASMR, 1990-2019 | 0.576 (0.370, 0.782) | -0.096 (-0.270, 0.078)* | -0.322 (-0.499, -0.143) | -0.145 (-0.414, 0.124)* | 1.991 (1.875, 2.107) |
| Turkey |  |  |  |  |  |
| ASMR in 2019 (per 100,000) | 10.852 (7.064, 15.732) | 34.414 (21.735, 50.003) | 18.699 (12.272, 26.891) | 11.466 (6.593, 16.965) | 8.164 (5.097, 11.858) |
| AAPC on ASMR, 1990-2019 | -0.073 (-0.492, 0.348)* | -1.953 (-2.399, -1.506) | -0.164 (-0.755, 0.430)* | -0.810 (-1.366, -0.250) | -1.096 (-1.332, -0.859) |
| Turkmenistan |  |  |  |  |  |
| ASMR in 2019 (per 100,000) | 3.027 (1.647, 4.870) | 101.066 (54.072, 157.141) | 54.095 (29.766, 86.183) | 3.533 (1.676, 7.672) | 5.503 (3.165, 8.538) |
| AAPC on ASMR, 1990-2019 | -1.367 (-2.251, -0.475) | -0.110 (-0.840, 0.625)* | 0.729 (-0.069, 1.533)* | -3.738 (-4.285, -3.188) | 2.104 (1.490, 2.721) |
| Tuvalu |  |  |  |  |  |
| ASMR in 2019 (per 100,000) | 1.515 (0.475, 3.644) | 14.329 (4.723, 34.211) | 10.239 (3.663, 22.851) | 3.670 (1.297, 7.946) | 6.739 (1.573, 18.197) |
| AAPC on ASMR, 1990-2019 | 2.075 (1.987, 2.163) | 2.073 (1.999, 2.147) | 0.847 (0.748, 0.947) | -1.324 (-1.496, -1.152) | 4.285 (4.044, 4.526) |
| Uganda |  |  |  |  |  |
| ASMR in 2019 (per 100,000) | 0.913 (0.397, 1.680) | 8.574 (3.514, 16.549) | 12.689 (5.580, 23.512) | 5.764 (2.763, 10.246) | 3.312 (1.322, 6.529) |
| AAPC on ASMR, 1990-2019 | 4.374 (4.176, 4.572) | 3.815 (3.692, 3.939) | 3.183 (3.016, 3.349) | 1.786 (1.582, 1.991) | 3.977 (3.721, 4.233) |
| Ukraine |  |  |  |  |  |
| ASMR in 2019 (per 100,000) | 4.148 (2.250, 6.580) | 69.474 (36.991, 111.693) | 23.012 (12.594, 36.853) | 2.559 (1.318, 4.617) | 0.726 (0.388, 1.151) |
| AAPC on ASMR, 1990-2019 | -3.158 (-3.946, -2.364) | -0.453 (-1.132, 0.230)* | -2.399 (-3.741, -1.039) | -5.409 (-5.966, -4.848) | -1.282 (-2.221, -0.335) |
| United Arab Emirates |  |  |  |  |  |
| ASMR in 2019 (per 100,000) | 9.095 (5.159, 14.869) | 72.077 (44.432, 107.232) | 42.593 (26.218, 65.516) | 15.122 (8.446, 25.168) | 23.110 (14.029, 35.054) |
| AAPC on ASMR, 1990-2019 | -0.134 (-0.452, 0.185)* | -1.739 (-2.446, -1.027) | -2.301 (-3.031, -1.565) | -0.996 (-1.617, -0.371) | -1.107 (-1.740, -0.470) |
| United Kingdom |  |  |  |  |  |
| ASMR in 2019 (per 100,000) | 3.628 (2.164, 5.509) | 6.581 (3.490, 10.950) | 3.154 (1.799, 5.135) | 3.053 (1.730, 4.758) | 0.663 (0.364, 1.046) |
| AAPC on ASMR, 1990-2019 | -3.443 (-3.681, -3.204) | -6.044 (-6.187, -5.900) | -5.295 (-5.526, -5.062) | -2.895 (-3.066, -2.725) | -3.972 (-4.093, -3.850) |
| United Republic of Tanzania |  |  |  |  |  |
| ASMR in 2019 (per 100,000) | 0.947 (0.381, 1.925) | 7.755 (3.358, 14.901) | 10.804 (4.908, 19.927) | 3.140 (1.663, 5.309) | 2.457 (1.036, 4.732) |
| AAPC on ASMR, 1990-2019 | 3.480 (3.291, 3.670) | 3.410 (3.094, 3.726) | 2.709 (2.221, 3.199) | 1.852 (1.676, 2.028) | 3.429 (3.233, 3.625) |
| United States of America |  |  |  |  |  |
| ASMR in 2019 (per 100,000) | 2.607 (1.253, 4.378) | 6.119 (2.709, 11.237) | 2.078 (0.983, 3.755) | 2.081 (0.962, 3.540) | 1.335 (0.608, 2.376) |
| AAPC on ASMR, 1990-2019 | -3.845 (-4.073, -3.616) | -5.051 (-5.260, -4.841) | -4.132 (-4.358, -3.905) | -2.328 (-2.654, -2.000) | -2.545 (-2.759, -2.330) |
| United States Virgin Islands |  |  |  |  |  |
| ASMR in 2019 (per 100,000) | 2.346 (1.015, 4.184) | 15.823 (6.551, 29.111) | 5.496 (2.322, 10.072) | 1.029 (0.434, 1.852) | 5.822 (2.401, 10.416) |
| AAPC on ASMR, 1990-2019 | 0.625 (0.431, 0.819) | -0.798 (-1.102, -0.494) | -1.021 (-1.350, -0.690) | -0.669 (-1.076, -0.261) | 0.257 (0.051, 0.463) |
| Uruguay |  |  |  |  |  |
| ASMR in 2019 (per 100,000) | 3.582 (1.612, 6.049) | 6.611 (2.833, 11.876) | 5.699 (2.565, 10.187) | 3.019 (1.336, 5.278) | 2.110 (0.917, 3.666) |
| AAPC on ASMR, 1990-2019 | -1.218 (-1.566, -0.869) | -3.061 (-3.408, -2.713) | -2.591 (-2.824, -2.358) | 0.044 (-0.428, 0.519)* | -0.231 (-0.529, 0.069)* |
| Uzbekistan |  |  |  |  |  |
| ASMR in 2019 (per 100,000) | 4.572 (2.693, 6.975) | 208.753 (121.678, 303.465) | 71.265 (41.521, 106.802) | 7.374 (3.806, 14.225) | 12.065 (7.449, 17.604) |
| AAPC on ASMR, 1990-2019 | 0.136 (-0.217, 0.490)* | 4.008 (3.661, 4.356) | 2.356 (1.803, 2.913) | -0.812 (-1.292, -0.331) | 6.787 (6.283, 7.293) |
| Vanuatu |  |  |  |  |  |
| ASMR in 2019 (per 100,000) | 2.067 (0.509, 5.213) | 22.196 (6.105, 53.401) | 16.528 (4.343, 39.534) | 8.558 (2.432, 20.579) | 4.160 (1.035, 10.376) |
| AAPC on ASMR, 1990-2019 | 2.391 (2.084, 2.698) | 2.406 (2.135, 2.677) | 1.749 (1.514, 1.985) | 0.195 (0.001, 0.389) | 4.015 (3.729, 4.302) |
| Venezuela (Bolivarian Republic of) |  |  |  |  |  |
| ASMR in 2019 (per 100,000) | 5.354 (2.992, 8.477) | 33.911 (18.600, 53.745) | 16.311 (8.916, 26.066) | 6.172 (3.247, 9.922) | 12.021 (6.984, 18.187) |
| AAPC on ASMR, 1990-2019 | 0.464 (-0.304, 1.238)* | -0.690 (-1.352, -0.023) | -0.769 (-1.381, -0.154) | 1.157 (0.298, 2.023) | 0.868 (-0.153, 1.900)* |
| Viet Nam |  |  |  |  |  |
| ASMR in 2019 (per 100,000) | 5.539 (3.198, 8.577) | 17.000 (10.075, 25.951) | 37.354 (23.362, 54.094) | 8.689 (3.067, 13.607) | 6.401 (3.430, 10.345) |
| AAPC on ASMR, 1990-2019 | 3.396 (3.314, 3.477) | 2.657 (2.578, 2.736) | 2.582 (2.442, 2.722) | 0.461 (0.397, 0.526) | 4.440 (4.351, 4.530) |
| Yemen |  |  |  |  |  |
| ASMR in 2019 (per 100,000) | 3.000 (1.242, 5.835) | 74.751 (35.164, 124.545) | 39.260 (17.813, 67.909) | 12.474 (6.386, 21.325) | 3.207 (1.435, 5.861) |
| AAPC on ASMR, 1990-2019 | 4.921 (4.742, 5.100) | 4.021 (3.717, 4.326) | 3.889 (3.660, 4.118) | 2.826 (2.576, 3.077) | 5.987 (5.829, 6.144) |
| Zambia |  |  |  |  |  |
| ASMR in 2019 (per 100,000) | 1.745 (0.778, 3.227) | 12.122 (5.659, 21.457) | 26.428 (12.893, 44.824) | 6.014 (3.256, 10.081) | 4.897 (2.190, 8.911) |
| AAPC on ASMR, 1990-2019 | 3.380 (3.089, 3.673) | 2.725 (2.492, 2.959) | 3.886 (3.675, 4.098) | 1.531 (1.405, 1.658) | 3.540 (3.383, 3.698) |
| Zimbabwe |  |  |  |  |  |
| ASMR in 2019 (per 100,000) | 1.741 (0.776, 3.087) | 16.252 (7.379, 28.835) | 11.811 (5.293, 21.669) | 3.977 (1.931, 6.934) | 4.729 (1.947, 8.836) |
| AAPC on ASMR, 1990-2019 | 0.947 (0.763, 1.131) | 1.623 (1.292, 1.956) | 1.159 (0.870, 1.449) | 0.055 (-0.236, 0.345)* | 2.626 (2.324, 2.929) |
| TBL: tracheal, bronchus, and lung; IHD: ischemic heart disease; COPD: chronic obstructive pulmonary disease; ASMR: age-standardized mortality rate; AAPC: average annual percent change.  ***NOT** statistically significant since *P* value > 0.05. | | | | | |

| **Table S3 The ASDR of non-communicable diseases attributed to ambient particulate matter pollution in 2019 as well as temporal trends from 1990-2019, by national level** | | | | | |
| --- | --- | --- | --- | --- | --- |
| Location | TBL cancer | IHD | Stroke | COPD | Diabetes mellitus |
| Afghanistan |  |  |  |  |  |
| ASDR in 2019 (per 100,000) | 47.790 (15.421, 112.499) | 1111.544 (449.930, 2162.347) | 633.159 (253.824, 1269.953) | 242.914 (114.848, 440.102) | 167.377 (60.741, 324.643) |
| AAPC on ASDR, 1990-2019 | 2.890 (2.647, 3.134) | 2.330 (1.940, 2.722) | 2.661 (2.302, 3.022) | 2.470 (2.226, 2.714) | 5.209 (4.906, 5.512) |
| Albania |  |  |  |  |  |
| ASDR in 2019 (per 100,000) | 132.354 (79.001, 207.790) | 541.373 (330.994, 827.196) | 504.672 (330.954, 731.392) | 65.652 (42.393, 96.012) | 79.489 (44.217, 124.694) |
| AAPC on ASDR, 1990-2019 | -0.032 (-0.549, 0.489)* | 0.334 (-0.047, 0.716)* | -0.589 (-1.116, -0.058) | -3.307 (-3.698, -2.913) | 2.786 (2.569, 3.003) |
| Algeria |  |  |  |  |  |
| ASDR in 2019 (per 100,000) | 86.263 (49.358, 136.745) | 1423.724 (857.942, 2096.119) | 739.920 (458.963, 1090.235) | 173.994 (104.279, 267.232) | 365.130 (227.751, 537.191) |
| AAPC on ASDR, 1990-2019 | -0.022 (-0.163, 0.119)* | -1.322 (-1.385, -1.258) | -1.192 (-1.337, -1.046) | -0.255 (-0.446, -0.064) | 2.281 (2.221, 2.340) |
| American Samoa |  |  |  |  |  |
| ASDR in 2019 (per 100,000) | 41.383 (14.733, 88.881) | 265.923 (91.598, 581.061) | 205.181 (74.756, 422.739) | 55.120 (18.658, 114.144) | 289.189 (100.193, 651.485) |
| AAPC on ASDR, 1990-2019 | -0.457 (-0.694, -0.219) | -0.224 (-0.446, 0.000) | -1.163 (-1.418, -0.908) | -2.182 (-2.431, -1.933) | 1.793 (1.442, 2.145) |
| Andorra |  |  |  |  |  |
| ASDR in 2019 (per 100,000) | 77.323 (36.180, 135.626) | 82.068 (34.855, 155.450) | 46.906 (21.861, 84.660) | 43.891 (20.630, 75.338) | 54.059 (23.848, 95.073) |
| AAPC on ASDR, 1990-2019 | -2.814 (-2.939, -2.688) | -3.835 (-3.972, -3.697) | -3.609 (-3.781, -3.436) | -2.989 (-3.127, -2.851) | -0.608 (-0.721, -0.495) |
| Angola |  |  |  |  |  |
| ASDR in 2019 (per 100,000) | 69.355 (30.219, 129.900) | 420.042 (188.339, 763.983) | 561.430 (263.162, 1001.517) | 180.827 (98.553, 304.605) | 211.650 (98.540, 368.016) |
| AAPC on ASDR, 1990-2019 | 4.578 (4.143, 5.015) | 4.351 (3.938, 4.766) | 3.845 (3.423, 4.269) | 2.445 (2.143, 2.747) | 5.718 (5.373, 6.064) |
| Antigua and Barbuda |  |  |  |  |  |
| ASDR in 2019 (per 100,000) | 46.273 (16.459, 87.408) | 349.027 (119.968, 641.861) | 348.092 (122.964, 648.625) | 36.448 (12.390, 71.342) | 512.372 (202.392, 849.017) |
| AAPC on ASDR, 1990-2019 | -0.561 (-1.445, 0.331)* | -1.839 (-2.225, -1.452) | -1.670 (-2.402, -0.933) | 0.499 (-0.442, 1.449)* | 0.693 (0.372, 1.014) |
| Argentina |  |  |  |  |  |
| ASDR in 2019 (per 100,000) | 101.310 (58.258, 156.922) | 268.411 (143.859, 429.246) | 208.467 (117.268, 331.747) | 95.985 (53.076, 150.489) | 157.461 (88.351, 242.042) |
| AAPC on ASDR, 1990-2019 | -1.273 (-1.482, -1.064) | -2.662 (-2.884, -2.440) | -3.035 (-3.453, -2.615) | -0.392 (-0.598, -0.186) | 0.865 (0.632, 1.098) |
| Armenia |  |  |  |  |  |
| ASDR in 2019 (per 100,000) | 282.559 (175.640, 408.845) | 1486.405 (940.052, 2092.088) | 562.436 (355.774, 814.970) | 221.506 (132.945, 332.105) | 391.382 (256.093, 541.688) |
| AAPC on ASDR, 1990-2019 | -0.264 (-0.769, 0.244)* | -0.263 (-0.551, 0.025)* | -1.267 (-1.769, -0.762) | -1.055 (-1.447, -0.662) | 2.892 (2.604, 3.181) |
| Australia |  |  |  |  |  |
| ASDR in 2019 (per 100,000) | 27.360 (6.622, 54.574) | 52.794 (12.074, 112.323) | 26.851 (6.412, 57.603) | 24.148 (5.879, 49.965) | 34.698 (7.565, 73.091) |
| AAPC on ASDR, 1990-2019 | -2.459 (-2.740, -2.176) | -5.108 (-5.429, -4.787) | -3.876 (-4.147, -3.604) | -2.426 (-2.623, -2.230) | -0.229 (-0.405, -0.053) |
| Austria |  |  |  |  |  |
| ASDR in 2019 (per 100,000) | 85.466 (55.405, 123.918) | 185.569 (107.000, 290.068) | 76.589 (47.360, 117.540) | 53.993 (33.412, 79.703) | 98.606 (55.153, 154.820) |
| AAPC on ASDR, 1990-2019 | -2.636 (-3.019, -2.251) | -4.920 (-5.270, -4.568) | -5.694 (-5.964, -5.423) | -2.091 (-2.365, -1.817) | -0.621 (-0.927, -0.314) |
| Azerbaijan |  |  |  |  |  |
| ASDR in 2019 (per 100,000) | 180.765 (90.445, 299.301) | 2094.931 (1114.935, 3241.857) | 1010.665 (544.274, 1602.600) | 157.388 (78.779, 273.258) | 306.362 (173.956, 464.076) |
| AAPC on ASDR, 1990-2019 | 0.423 (0.117, 0.731) | 1.314 (1.052, 1.578) | 1.395 (1.120, 1.671) | -0.219 (-0.507, 0.070)* | 4.389 (4.156, 4.622) |
| Bahamas |  |  |  |  |  |
| ASDR in 2019 (per 100,000) | 68.803 (18.427, 142.784) | 427.946 (119.052, 852.712) | 327.682 (89.167, 671.629) | 40.266 (10.562, 84.726) | 372.742 (110.504, 673.572) |
| AAPC on ASDR, 1990-2019 | -0.845 (-1.220, -0.469) | -1.438 (-1.668, -1.207) | -1.238 (-1.438, -1.038) | -0.379 (-0.636, -0.122) | 0.101 (-0.126, 0.329)* |
| Bahrain |  |  |  |  |  |
| ASDR in 2019 (per 100,000) | 200.410 (125.570, 301.074) | 1291.362 (923.627, 1737.483) | 550.656 (393.651, 753.450) | 329.218 (230.606, 452.159) | 1393.179 (944.105, 1929.465) |
| AAPC on ASDR, 1990-2019 | -2.719 (-3.110, -2.325) | -3.977 (-4.427, -3.524) | -2.375 (-2.730, -2.019) | -2.497 (-3.239, -1.749) | 1.712 (1.224, 2.201) |
| Bangladesh |  |  |  |  |  |
| ASDR in 2019 (per 100,000) | 50.699 (23.481, 99.077) | 675.571 (389.335, 1036.854) | 885.730 (511.118, 1342.816) | 469.653 (307.901, 747.228) | 164.306 (91.067, 259.621) |
| AAPC on ASDR, 1990-2019 | 3.251 (2.660, 3.845) | 3.943 (3.314, 4.576) | 3.063 (2.518, 3.610) | 0.761 (0.312, 1.212) | 4.738 (4.276, 5.201) |
| Barbados |  |  |  |  |  |
| ASDR in 2019 (per 100,000) | 57.483 (23.353, 103.129) | 352.183 (140.157, 612.508) | 382.183 (151.421, 692.663) | 44.492 (17.014, 82.570) | 575.218 (265.064, 895.588) |
| AAPC on ASDR, 1990-2019 | -0.193 (-0.748, 0.365)* | -2.038 (-2.512, -1.561) | -1.648 (-2.059, -1.235) | 0.448 (0.069, 0.829) | 0.031 (-0.197, 0.261)* |
| Belarus |  |  |  |  |  |
| ASDR in 2019 (per 100,000) | 120.702 (71.668, 188.382) | 1294.633 (740.681, 2017.397) | 532.203 (325.400, 811.032) | 66.124 (40.382, 106.724) | 73.311 (40.668, 116.262) |
| AAPC on ASDR, 1990-2019 | -2.897 (-3.631, -2.158) | -0.992 (-1.736, -0.243) | -2.025 (-2.542, -1.506) | -4.752 (-5.429, -4.069) | -0.819 (-1.158, -0.478) |
| Belgium |  |  |  |  |  |
| ASDR in 2019 (per 100,000) | 126.362 (82.098, 181.372) | 152.640 (89.151, 236.457) | 98.474 (61.342, 150.581) | 89.333 (56.462, 130.554) | 95.907 (52.538, 152.642) |
| AAPC on ASDR, 1990-2019 | -3.204 (-3.616, -2.790) | -5.104 (-5.360, -4.847) | -4.516 (-4.674, -4.359) | -2.573 (-2.775, -2.371) | -0.880 (-1.090, -0.668) |
| Belize |  |  |  |  |  |
| ASDR in 2019 (per 100,000) | 76.425 (25.471, 145.862) | 424.251 (153.796, 773.126) | 340.175 (126.365, 630.239) | 110.382 (39.370, 212.991) | 442.527 (155.139, 746.873) |
| AAPC on ASDR, 1990-2019 | 2.581 (1.940, 3.226) | 0.529 (0.221, 0.838) | 1.134 (0.609, 1.662) | 1.694 (1.110, 2.282) | 3.788 (3.132, 4.449) |
| Benin |  |  |  |  |  |
| ASDR in 2019 (per 100,000) | 34.532 (12.885, 68.435) | 273.222 (110.417, 528.681) | 368.545 (154.071, 692.612) | 150.505 (73.877, 261.023) | 98.447 (37.854, 190.800) |
| AAPC on ASDR, 1990-2019 | 2.847 (2.517, 3.178) | 2.287 (1.883, 2.693) | 1.979 (1.485, 2.475) | 1.352 (1.012, 1.692) | 3.981 (3.560, 4.404) |
| Bermuda |  |  |  |  |  |
| ASDR in 2019 (per 100,000) | 33.871 (6.004, 73.768) | 92.509 (16.090, 204.310) | 45.946 (8.376, 101.263) | 10.990 (1.906, 23.600) | 72.729 (11.402, 157.631) |
| AAPC on ASDR, 1990-2019 | -3.653 (-4.145, -3.157) | -5.877 (-6.240, -5.513) | -4.670 (-5.159, -4.180) | -3.058 (-3.501, -2.613) | -1.840 (-2.287, -1.391) |
| Bhutan |  |  |  |  |  |
| ASDR in 2019 (per 100,000) | 42.874 (20.636, 76.064) | 625.624 (331.377, 1008.612) | 367.366 (199.460, 593.569) | 628.587 (408.981, 934.133) | 180.823 (93.738, 294.542) |
| AAPC on ASDR, 1990-2019 | 5.599 (5.317, 5.881) | 5.125 (4.925, 5.325) | 3.599 (3.395, 3.803) | 3.165 (3.036, 3.295) | 7.317 (7.096, 7.539) |
| Bolivia (Plurinational State of) |  |  |  |  |  |
| ASDR in 2019 (per 100,000) | 77.454 (36.356, 136.806) | 454.953 (227.643, 767.086) | 426.553 (221.260, 712.934) | 167.500 (95.671, 268.297) | 312.164 (172.300, 485.252) |
| AAPC on ASDR, 1990-2019 | 0.279 (0.013, 0.546) | -0.774 (-1.021, -0.526) | -1.406 (-1.619, -1.191) | -1.400 (-1.566, -1.235) | 1.794 (1.609, 1.980) |
| Bosnia and Herzegovina |  |  |  |  |  |
| ASDR in 2019 (per 100,000) | 297.986 (189.051, 425.181) | 795.298 (530.443, 1126.327) | 799.036 (557.839, 1095.575) | 145.660 (100.779, 200.779) | 383.082 (240.216, 549.062) |
| AAPC on ASDR, 1990-2019 | 0.826 (0.308, 1.347) | -0.561 (-0.804, -0.317) | 0.179 (-0.215, 0.575)* | -1.216 (-1.395, -1.037) | 4.660 (4.264, 5.057) |
| Botswana |  |  |  |  |  |
| ASDR in 2019 (per 100,000) | 135.690 (67.624, 233.533) | 686.135 (364.412, 1121.989) | 783.330 (445.116, 1252.602) | 269.958 (155.397, 431.562) | 542.978 (299.148, 859.145) |
| AAPC on ASDR, 1990-2019 | 3.165 (2.963, 3.368) | 3.152 (2.897, 3.408) | 2.000 (1.756, 2.245) | 0.810 (0.543, 1.078) | 5.212 (4.686, 5.741) |
| Brazil |  |  |  |  |  |
| ASDR in 2019 (per 100,000) | 49.556 (31.803, 71.165) | 255.240 (152.220, 390.106) | 208.405 (133.816, 301.167) | 82.383 (51.999, 121.294) | 163.622 (94.096, 255.050) |
| AAPC on ASDR, 1990-2019 | -0.601 (-0.744, -0.457) | -2.107 (-2.291, -1.923) | -2.892 (-3.061, -2.723) | -2.015 (-2.298, -1.731) | 0.632 (0.435, 0.828) |
| Brunei Darussalam |  |  |  |  |  |
| ASDR in 2019 (per 100,000) | 63.626 (17.895, 123.682) | 192.403 (49.793, 395.517) | 128.639 (35.609, 256.411) | 54.735 (14.448, 106.771) | 248.301 (64.422, 492.538) |
| AAPC on ASDR, 1990-2019 | -1.119 (-2.054, -0.176) | -2.359 (-3.261, -1.449) | -3.635 (-4.434, -2.828) | -2.956 (-3.450, -2.460) | -0.767 (-1.223, -0.310) |
| Bulgaria |  |  |  |  |  |
| ASDR in 2019 (per 100,000) | 223.985 (143.326, 326.509) | 1036.942 (661.182, 1525.342) | 939.449 (637.521, 1322.469) | 117.264 (79.145, 166.106) | 197.210 (118.895, 287.148) |
| AAPC on ASDR, 1990-2019 | -0.027 (-0.440, 0.387)* | -1.756 (-2.288, -1.221) | -1.834 (-2.349, -1.317) | -2.069 (-2.200, -1.938) | 0.464 (0.278, 0.651) |
| Burkina Faso |  |  |  |  |  |
| ASDR in 2019 (per 100,000) | 23.135 (6.706, 54.734) | 222.079 (68.277, 491.108) | 216.255 (68.200, 483.339) | 84.464 (31.949, 170.352) | 68.937 (19.176, 159.134) |
| AAPC on ASDR, 1990-2019 | 2.912 (2.587, 3.238) | 2.915 (2.551, 3.280) | 2.439 (2.169, 2.709) | 1.898 (1.452, 2.345) | 2.858 (2.589, 3.127) |
| Burundi |  |  |  |  |  |
| ASDR in 2019 (per 100,000) | 12.091 (3.320, 30.095) | 149.662 (43.107, 360.175) | 205.090 (60.605, 491.179) | 115.978 (40.797, 247.570) | 47.744 (12.915, 111.886) |
| AAPC on ASDR, 1990-2019 | 0.511 (0.237, 0.786) | 0.436 (0.247, 0.624) | -0.626 (-0.893, -0.358) | -0.145 (-0.315, 0.026)* | 0.637 (0.375, 0.899) |
| Cabo Verde |  |  |  |  |  |
| ASDR in 2019 (per 100,000) | 154.364 (87.378, 240.678) | 913.044 (568.694, 1326.823) | 783.007 (479.633, 1157.752) | 226.706 (143.413, 334.657) | 338.300 (210.070, 492.545) |
| AAPC on ASDR, 1990-2019 | 6.021 (5.299, 6.747) | 4.742 (4.124, 5.365) | 4.677 (4.072, 5.286) | 0.969 (0.411, 1.531) | 8.287 (7.736, 8.840) |
| Cambodia |  |  |  |  |  |
| ASDR in 2019 (per 100,000) | 73.323 (30.592, 138.250) | 272.403 (121.558, 497.316) | 423.820 (194.244, 765.306) | 141.104 (76.948, 233.248) | 98.897 (43.058, 179.906) |
| AAPC on ASDR, 1990-2019 | 2.770 (2.630, 2.911) | 2.350 (2.167, 2.533) | 1.991 (1.884, 2.098) | 1.448 (1.319, 1.577) | 3.982 (3.845, 4.120) |
| Cameroon |  |  |  |  |  |
| ASDR in 2019 (per 100,000) | 97.644 (46.737, 168.581) | 620.754 (326.175, 1020.386) | 824.350 (433.747, 1317.187) | 300.575 (180.896, 451.186) | 282.242 (144.233, 464.102) |
| AAPC on ASDR, 1990-2019 | 3.037 (2.818, 3.257) | 3.005 (2.790, 3.220) | 2.331 (2.016, 2.646) | 0.794 (0.666, 0.923) | 3.866 (3.716, 4.017) |
| Canada |  |  |  |  |  |
| ASDR in 2019 (per 100,000) | 44.674 (19.010, 78.626) | 73.519 (28.436, 142.090) | 33.639 (13.685, 65.612) | 24.914 (10.091, 44.850) | 36.537 (13.895, 70.782) |
| AAPC on ASDR, 1990-2019 | -3.466 (-3.737, -3.195) | -5.206 (-5.528, -4.883) | -4.169 (-4.359, -3.979) | -2.747 (-2.919, -2.574) | -1.039 (-1.397, -0.679) |
| Central African Republic |  |  |  |  |  |
| ASDR in 2019 (per 100,000) | 31.810 (6.664, 85.793) | 282.636 (79.437, 666.157) | 383.159 (111.159, 899.223) | 190.829 (65.891, 423.548) | 101.292 (26.553, 234.228) |
| AAPC on ASDR, 1990-2019 | 0.935 (0.776, 1.094) | 1.284 (1.081, 1.487) | 1.105 (0.800, 1.411) | 0.892 (0.612, 1.174) | 1.779 (1.638, 1.920) |
| Chad |  |  |  |  |  |
| ASDR in 2019 (per 100,000) | 27.428 (7.873, 66.215) | 206.459 (65.062, 483.994) | 269.372 (87.184, 615.789) | 130.138 (49.030, 272.085) | 65.633 (17.983, 150.462) |
| AAPC on ASDR, 1990-2019 | 3.582 (3.384, 3.780) | 2.634 (2.339, 2.929) | 2.390 (2.136, 2.644) | 1.818 (1.545, 2.091) | 3.647 (3.385, 3.910) |
| Chile |  |  |  |  |  |
| ASDR in 2019 (per 100,000) | 97.397 (67.361, 133.537) | 273.745 (182.704, 375.737) | 279.770 (192.786, 383.994) | 96.190 (62.163, 134.354) | 225.168 (141.685, 324.068) |
| AAPC on ASDR, 1990-2019 | -0.016 (-0.382, 0.351)* | -1.799 (-2.203, -1.394) | -1.927 (-2.276, -1.576) | -0.466 (-0.908, -0.022) | 2.200 (1.893, 2.507) |
| China |  |  |  |  |  |
| ASDR in 2019 (per 100,000) | 337.811 (239.238, 453.142) | 757.210 (560.703, 971.543) | 1171.451 (883.107, 1470.685) | 504.105 (390.784, 650.497) | 162.248 (106.288, 224.831) |
| AAPC on ASDR, 1990-2019 | 2.921 (2.619, 3.224) | 2.728 (2.459, 2.997) | 0.941 (0.655, 1.228) | -2.430 (-2.836, -2.023) | 3.542 (3.413, 3.671) |
| Colombia |  |  |  |  |  |
| ASDR in 2019 (per 100,000) | 65.881 (39.823, 102.079) | 367.057 (223.636, 562.703) | 208.510 (127.727, 319.058) | 139.190 (85.360, 208.646) | 242.072 (148.852, 359.421) |
| AAPC on ASDR, 1990-2019 | -0.659 (-1.209, -0.105) | -1.969 (-2.623, -1.310) | -2.407 (-2.961, -1.850) | -1.225 (-1.765, -0.683) | 1.419 (0.950, 1.890) |
| Comoros |  |  |  |  |  |
| ASDR in 2019 (per 100,000) | 16.013 (6.273, 32.023) | 170.930 (72.257, 327.739) | 220.219 (96.250, 407.405) | 77.954 (41.139, 130.743) | 61.109 (25.491, 116.863) |
| AAPC on ASDR, 1990-2019 | 2.857 (2.381, 3.335) | 2.800 (2.171, 3.432) | 1.787 (1.329, 2.247) | 1.242 (0.874, 1.612) | 3.323 (2.909, 3.740) |
| Congo |  |  |  |  |  |
| ASDR in 2019 (per 100,000) | 115.823 (51.473, 217.059) | 775.160 (379.299, 1347.689) | 907.861 (465.463, 1539.012) | 290.168 (150.705, 497.712) | 352.517 (177.696, 571.554) |
| AAPC on ASDR, 1990-2019 | 2.594 (2.209, 2.981) | 2.393 (1.784, 3.005) | 1.932 (1.380, 2.486) | 0.998 (0.611, 1.387) | 3.552 (3.220, 3.885) |
| Cook Islands |  |  |  |  |  |
| ASDR in 2019 (per 100,000) | 37.809 (6.394, 97.384) | 161.225 (27.604, 411.420) | 117.540 (21.516, 290.775) | 37.260 (7.238, 88.214) | 257.050 (48.113, 630.829) |
| AAPC on ASDR, 1990-2019 | -1.010 (-1.203, -0.817) | -1.186 (-1.281, -1.090) | -1.911 (-2.323, -1.497) | -2.428 (-2.677, -2.178) | 0.889 (0.632, 1.148) |
| Costa Rica |  |  |  |  |  |
| ASDR in 2019 (per 100,000) | 44.761 (26.961, 68.019) | 321.244 (188.014, 498.710) | 147.131 (89.131, 223.831) | 83.453 (51.304, 124.471) | 230.556 (133.157, 352.338) |
| AAPC on ASDR, 1990-2019 | 0.534 (0.154, 0.915) | -0.417 (-0.930, 0.098)* | -0.539 (-1.011, -0.064) | 0.036 (-0.634, 0.710)* | 2.509 (2.026, 2.995) |
| Coted'Ivoire |  |  |  |  |  |
| ASDR in 2019 (per 100,000) | 62.380 (25.840, 119.370) | 442.968 (193.528, 806.605) | 505.387 (225.657, 909.032) | 198.943 (102.865, 333.851) | 157.988 (67.918, 283.137) |
| AAPC on ASDR, 1990-2019 | 2.597 (2.264, 2.931) | 1.804 (1.334, 2.276) | 1.760 (1.326, 2.196) | 0.790 (0.425, 1.156) | 3.394 (2.981, 3.809) |
| Croatia |  |  |  |  |  |
| ASDR in 2019 (per 100,000) | 190.593 (121.799, 278.853) | 504.035 (310.530, 753.807) | 354.282 (234.356, 508.199) | 97.045 (63.387, 136.566) | 195.549 (116.436, 291.296) |
| AAPC on ASDR, 1990-2019 | -1.769 (-2.407, -1.127) | -3.363 (-3.557, -3.168) | -3.586 (-4.138, -3.030) | -0.931 (-1.113, -0.749) | 0.504 (0.123, 0.886) |
| Cuba |  |  |  |  |  |
| ASDR in 2019 (per 100,000) | 168.720 (78.654, 298.158) | 460.090 (208.559, 796.582) | 274.051 (125.747, 486.228) | 104.774 (46.440, 191.370) | 231.091 (108.973, 389.382) |
| AAPC on ASDR, 1990-2019 | -0.161 (-0.617, 0.298)* | -2.213 (-2.705, -1.719) | -1.553 (-2.229, -0.873) | 0.825 (0.243, 1.412) | -0.228 (-0.373, -0.084) |
| Cyprus |  |  |  |  |  |
| ASDR in 2019 (per 100,000) | 100.405 (63.697, 146.803) | 332.106 (196.989, 511.606) | 136.201 (87.289, 201.866) | 88.679 (53.577, 131.254) | 206.154 (120.812, 312.567) |
| AAPC on ASDR, 1990-2019 | 0.099 (-0.199, 0.398)* | -3.177 (-3.741, -2.610) | -3.694 (-4.258, -3.127) | -1.920 (-2.396, -1.442) | -1.762 (-2.174, -1.347) |
| Czechia |  |  |  |  |  |
| ASDR in 2019 (per 100,000) | 143.637 (94.243, 205.989) | 480.949 (293.712, 725.019) | 221.733 (145.825, 322.554) | 88.992 (57.511, 125.269) | 274.033 (160.655, 412.981) |
| AAPC on ASDR, 1990-2019 | -3.263 (-3.718, -2.806) | -4.636 (-5.070, -4.200) | -5.430 (-5.815, -5.043) | -1.767 (-1.934, -1.599) | 0.750 (0.575, 0.925) |
| Democratic People's Republic of Korea |  |  |  |  |  |
| ASDR in 2019 (per 100,000) | 178.194 (90.644, 295.211) | 632.734 (345.925, 1015.281) | 1262.789 (722.850, 1953.638) | 680.155 (428.797, 1003.212) | 108.940 (57.261, 179.424) |
| AAPC on ASDR, 1990-2019 | 2.183 (2.129, 2.238) | 2.788 (2.625, 2.951) | 2.152 (2.052, 2.252) | 0.325 (0.218, 0.432) | 2.986 (2.868, 3.104) |
| Democratic Republic of the Congo |  |  |  |  |  |
| ASDR in 2019 (per 100,000) | 35.166 (10.014, 94.275) | 239.030 (88.265, 490.054) | 311.210 (117.898, 632.727) | 182.551 (80.466, 360.234) | 94.221 (34.138, 189.193) |
| AAPC on ASDR, 1990-2019 | 1.058 (0.730, 1.387) | 1.268 (0.844, 1.694) | 1.092 (0.662, 1.524) | 1.029 (0.755, 1.303) | 1.745 (1.447, 2.044) |
| Denmark |  |  |  |  |  |
| ASDR in 2019 (per 100,000) | 87.645 (48.539, 137.379) | 97.019 (49.202, 164.935) | 74.146 (39.309, 124.596) | 76.359 (40.808, 121.323) | 65.008 (33.006, 106.788) |
| AAPC on ASDR, 1990-2019 | -3.627 (-3.804, -3.450) | -7.020 (-7.439, -6.599) | -5.038 (-5.508, -4.566) | -2.679 (-3.119, -2.238) | -0.701 (-1.157, -0.242) |
| Djibouti |  |  |  |  |  |
| ASDR in 2019 (per 100,000) | 89.585 (35.332, 185.576) | 760.352 (326.075, 1333.189) | 931.029 (429.305, 1544.192) | 221.085 (106.139, 391.138) | 335.404 (174.553, 532.460) |
| AAPC on ASDR, 1990-2019 | 4.575 (4.382, 4.768) | 4.698 (4.325, 5.072) | 3.303 (3.122, 3.484) | 1.926 (1.773, 2.079) | 5.479 (5.316, 5.642) |
| Dominica |  |  |  |  |  |
| ASDR in 2019 (per 100,000) | 91.183 (34.610, 173.385) | 375.230 (142.059, 693.917) | 367.087 (145.057, 675.093) | 74.807 (26.792, 144.914) | 564.163 (230.164, 938.829) |
| AAPC on ASDR, 1990-2019 | 1.038 (0.753, 1.324) | -0.575 (-0.901, -0.249) | 0.018 (-0.281, 0.319)* | 0.750 (0.537, 0.963) | 2.530 (2.251, 2.810) |
| Dominican Republic |  |  |  |  |  |
| ASDR in 2019 (per 100,000) | 74.570 (27.111, 148.759) | 823.276 (320.603, 1572.284) | 523.941 (211.945, 1012.272) | 68.775 (27.164, 134.963) | 252.876 (99.176, 451.893) |
| AAPC on ASDR, 1990-2019 | 3.771 (3.281, 4.264) | 4.081 (3.344, 4.823) | 3.365 (2.709, 4.025) | 2.480 (1.930, 3.033) | 5.771 (5.305, 6.239) |
| Ecuador |  |  |  |  |  |
| ASDR in 2019 (per 100,000) | 53.892 (30.791, 85.749) | 378.675 (213.831, 594.359) | 282.898 (167.441, 431.998) | 79.275 (44.283, 123.200) | 326.748 (196.985, 474.464) |
| AAPC on ASDR, 1990-2019 | 0.958 (0.547, 1.371) | -0.071 (-0.373, 0.233)* | -1.258 (-1.610, -0.905) | -0.679 (-1.215, -0.141) | 3.184 (2.798, 3.571) |
| Egypt |  |  |  |  |  |
| ASDR in 2019 (per 100,000) | 126.589 (73.523, 198.637) | 3941.105 (2645.637, 5488.678) | 1326.687 (862.900, 1970.567) | 489.010 (312.085, 716.332) | 530.891 (350.481, 747.181) |
| AAPC on ASDR, 1990-2019 | 1.523 (1.094, 1.955) | 0.027 (-0.720, 0.780)* | -0.016 (-0.577, 0.548)* | 0.684 (0.193, 1.177) | 2.395 (1.954, 2.839) |
| El Salvador |  |  |  |  |  |
| ASDR in 2019 (per 100,000) | 58.142 (31.327, 98.368) | 488.216 (266.812, 805.217) | 221.735 (126.401, 363.474) | 86.449 (48.404, 140.763) | 387.135 (221.135, 596.476) |
| AAPC on ASDR, 1990-2019 | 2.745 (1.867, 3.630) | 1.409 (0.446, 2.381) | -0.307 (-1.544, 0.946)* | 0.461 (-0.052, 0.976)* | 6.149 (5.471, 6.831) |
| Equatorial Guinea |  |  |  |  |  |
| ASDR in 2019 (per 100,000) | 158.553 (70.926, 297.687) | 672.171 (331.569, 1151.656) | 885.132 (458.934, 1462.598) | 316.318 (153.760, 557.394) | 533.051 (297.743, 815.440) |
| AAPC on ASDR, 1990-2019 | 7.497 (7.128, 7.867) | 5.003 (4.550, 5.459) | 4.738 (4.257, 5.221) | 3.456 (3.059, 3.854) | 8.458 (8.188, 8.729) |
| Eritrea |  |  |  |  |  |
| ASDR in 2019 (per 100,000) | 35.987 (13.504, 71.237) | 382.312 (144.770, 752.833) | 558.795 (224.687, 1095.766) | 213.859 (100.416, 383.849) | 150.877 (58.415, 292.359) |
| AAPC on ASDR, 1990-2019 | 3.839 (3.656, 4.021) | 3.547 (3.390, 3.704) | 2.389 (2.240, 2.539) | 1.881 (1.788, 1.974) | 3.967 (3.826, 4.109) |
| Estonia |  |  |  |  |  |
| ASDR in 2019 (per 100,000) | 31.237 (10.279, 61.159) | 111.724 (34.296, 230.927) | 48.971 (16.031, 97.469) | 9.599 (3.115, 18.615) | 28.986 (9.333, 58.471) |
| AAPC on ASDR, 1990-2019 | -4.637 (-5.988, -3.266) | -6.233 (-7.270, -5.184) | -7.311 (-8.207, -6.407) | -4.284 (-5.045, -3.516) | -0.883 (-1.270, -0.495) |
| Eswatini |  |  |  |  |  |
| ASDR in 2019 (per 100,000) | 93.273 (40.385, 171.711) | 513.775 (254.319, 886.926) | 649.666 (335.245, 1069.997) | 255.659 (151.268, 406.411) | 560.847 (279.216, 940.290) |
| AAPC on ASDR, 1990-2019 | 2.687 (2.559, 2.815) | 2.861 (2.543, 3.179) | 2.086 (1.848, 2.324) | 0.622 (0.461, 0.783) | 4.713 (4.450, 4.976) |
| Ethiopia |  |  |  |  |  |
| ASDR in 2019 (per 100,000) | 12.782 (5.591, 24.857) | 147.285 (67.008, 274.142) | 191.098 (89.021, 348.170) | 90.704 (46.051, 153.388) | 61.513 (27.556, 115.373) |
| AAPC on ASDR, 1990-2019 | 3.252 (3.022, 3.482) | 2.318 (2.078, 2.558) | 1.557 (1.355, 1.760) | 1.426 (1.243, 1.610) | 2.392 (2.155, 2.629) |
| Fiji |  |  |  |  |  |
| ASDR in 2019 (per 100,000) | 29.920 (8.268, 71.669) | 807.004 (229.649, 1830.962) | 384.002 (116.408, 868.702) | 68.902 (19.306, 168.582) | 880.528 (224.284, 2004.615) |
| AAPC on ASDR, 1990-2019 | 2.332 (2.056, 2.609) | 1.883 (1.326, 2.444) | 1.512 (1.188, 1.837) | 0.137 (-0.223, 0.498)* | 5.282 (4.885, 5.682) |
| Finland |  |  |  |  |  |
| ASDR in 2019 (per 100,000) | 14.462 (2.707, 32.350) | 51.701 (8.976, 124.829) | 25.057 (4.570, 60.593) | 8.965 (1.630, 20.024) | 24.611 (4.200, 60.541) |
| AAPC on ASDR, 1990-2019 | -5.006 (-5.332, -4.679) | -7.078 (-7.349, -6.806) | -6.390 (-6.644, -6.135) | -4.204 (-4.565, -3.841) | -2.276 (-2.531, -2.020) |
| France |  |  |  |  |  |
| ASDR in 2019 (per 100,000) | 108.315 (67.815, 159.356) | 95.478 (53.510, 152.758) | 70.793 (42.029, 111.341) | 28.106 (16.649, 43.162) | 52.469 (29.331, 83.096) |
| AAPC on ASDR, 1990-2019 | -1.976 (-2.315, -1.635) | -4.597 (-4.900, -4.293) | -4.448 (-4.745, -4.151) | -3.487 (-3.879, -3.093) | -0.617 (-0.869, -0.364) |
| Gabon |  |  |  |  |  |
| ASDR in 2019 (per 100,000) | 183.203 (85.527, 332.519) | 867.526 (464.128, 1401.245) | 958.633 (522.917, 1530.044) | 250.477 (126.962, 422.599) | 686.202 (406.700, 1005.647) |
| AAPC on ASDR, 1990-2019 | 2.935 (2.493, 3.379) | 2.594 (2.184, 3.006) | 1.832 (1.659, 2.005) | 0.596 (0.288, 0.904) | 4.273 (3.967, 4.581) |
| Gambia |  |  |  |  |  |
| ASDR in 2019 (per 100,000) | 30.890 (12.960, 58.361) | 458.554 (201.041, 854.289) | 473.491 (214.818, 872.832) | 214.785 (110.300, 366.440) | 123.565 (50.966, 231.316) |
| AAPC on ASDR, 1990-2019 | 3.533 (2.840, 4.230) | 3.371 (2.453, 4.297) | 3.118 (2.439, 3.802) | 2.122 (1.653, 2.593) | 4.412 (3.901, 4.927) |
| Georgia |  |  |  |  |  |
| ASDR in 2019 (per 100,000) | 173.093 (104.473, 264.072) | 890.395 (544.612, 1330.615) | 786.779 (499.257, 1166.751) | 89.686 (55.522, 137.799) | 219.578 (124.546, 338.153) |
| AAPC on ASDR, 1990-2019 | 0.114 (-0.136, 0.364)* | -1.766 (-2.593, -0.932) | -0.876 (-1.415, -0.334) | 1.013 (0.632, 1.395) | 3.487 (3.102, 3.873) |
| Germany |  |  |  |  |  |
| ASDR in 2019 (per 100,000) | 97.226 (61.621, 142.285) | 187.095 (107.397, 294.959) | 90.418 (54.905, 140.596) | 67.546 (40.761, 99.791) | 117.919 (65.409, 188.764) |
| AAPC on ASDR, 1990-2019 | -2.286 (-2.478, -2.093) | -5.455 (-5.733, -5.176) | -5.149 (-5.404, -4.894) | -2.536 (-2.787, -2.284) | -1.253 (-1.424, -1.081) |
| Ghana |  |  |  |  |  |
| ASDR in 2019 (per 100,000) | 68.159 (37.512, 109.971) | 721.610 (407.741, 1121.503) | 1041.024 (600.521, 1603.217) | 282.827 (161.499, 432.836) | 306.681 (168.341, 478.854) |
| AAPC on ASDR, 1990-2019 | 2.984 (2.801, 3.167) | 2.878 (2.748, 3.008) | 2.658 (2.534, 2.782) | 1.834 (1.720, 1.948) | 4.731 (4.597, 4.866) |
| Greece |  |  |  |  |  |
| ASDR in 2019 (per 100,000) | 155.930 (102.450, 220.963) | 344.724 (207.728, 519.049) | 191.056 (122.130, 281.077) | 77.268 (48.306, 111.703) | 107.532 (59.536, 170.607) |
| AAPC on ASDR, 1990-2019 | -1.519 (-1.829, -1.209) | -2.287 (-2.679, -1.893) | -3.673 (-3.870, -3.477) | -0.876 (-1.241, -0.510) | 0.283 (0.143, 0.423) |
| Greenland |  |  |  |  |  |
| ASDR in 2019 (per 100,000) | 95.396 (8.384, 282.302) | 110.418 (9.315, 326.051) | 102.009 (9.341, 303.302) | 51.635 (4.865, 146.162) | 35.100 (2.721, 103.002) |
| AAPC on ASDR, 1990-2019 | -1.309 (-1.730, -0.887) | -3.336 (-3.746, -2.924) | -3.184 (-3.651, -2.714) | -2.359 (-2.739, -1.977) | 0.021 (-0.604, 0.649)* |
| Grenada |  |  |  |  |  |
| ASDR in 2019 (per 100,000) | 88.170 (32.670, 162.633) | 572.060 (210.666, 1007.694) | 566.922 (210.021, 1046.804) | 82.236 (27.167, 158.231) | 686.056 (289.326, 1088.162) |
| AAPC on ASDR, 1990-2019 | 1.163 (0.961, 1.365) | -0.386 (-0.657, -0.115) | -0.474 (-0.786, -0.162) | 0.591 (0.219, 0.964) | 2.962 (2.673, 3.252) |
| Guam |  |  |  |  |  |
| ASDR in 2019 (per 100,000) | 66.244 (25.166, 126.572) | 417.390 (150.694, 813.977) | 174.926 (65.634, 332.052) | 36.143 (12.837, 69.406) | 142.475 (53.722, 260.968) |
| AAPC on ASDR, 1990-2019 | -1.675 (-2.968, -0.365) | -0.536 (-1.580, 0.520)* | -1.795 (-2.796, -0.784) | -3.275 (-4.398, -2.139) | -0.568 (-1.582, 0.458)* |
| Guatemala |  |  |  |  |  |
| ASDR in 2019 (per 100,000) | 36.733 (18.869, 60.358) | 343.865 (185.886, 541.947) | 234.141 (124.338, 377.765) | 87.526 (54.112, 131.419) | 364.149 (191.884, 593.014) |
| AAPC on ASDR, 1990-2019 | 1.611 (0.514, 2.720) | 0.312 (-0.485, 1.116)* | 1.099 (0.468, 1.734) | 0.149 (-0.566, 0.869)* | 6.550 (5.966, 7.137) |
| Guinea |  |  |  |  |  |
| ASDR in 2019 (per 100,000) | 29.459 (10.182, 62.517) | 253.399 (93.565, 527.956) | 321.331 (121.410, 663.032) | 155.467 (69.048, 291.319) | 82.628 (28.565, 176.462) |
| AAPC on ASDR, 1990-2019 | 2.826 (2.633, 3.019) | 2.872 (2.697, 3.047) | 2.400 (2.164, 2.637) | 1.738 (1.613, 1.862) | 3.587 (3.454, 3.720) |
| Guinea-Bissau |  |  |  |  |  |
| ASDR in 2019 (per 100,000) | 42.464 (14.061, 94.712) | 418.568 (163.876, 836.751) | 488.182 (196.330, 973.917) | 198.588 (90.170, 367.891) | 121.605 (43.160, 252.664) |
| AAPC on ASDR, 1990-2019 | 2.055 (1.978, 2.131) | 2.199 (2.080, 2.318) | 1.915 (1.810, 2.020) | 0.912 (0.791, 1.033) | 3.016 (2.941, 3.091) |
| Guyana |  |  |  |  |  |
| ASDR in 2019 (per 100,000) | 52.030 (19.134, 100.391) | 1062.417 (395.038, 1983.153) | 966.139 (369.052, 1832.816) | 78.309 (28.451, 154.484) | 812.775 (331.397, 1342.919) |
| AAPC on ASDR, 1990-2019 | 0.946 (0.557, 1.336) | -0.421 (-0.924, 0.086)* | -1.576 (-2.076, -1.072) | 0.434 (-0.201, 1.073)* | 2.239 (1.888, 2.591) |
| Haiti |  |  |  |  |  |
| ASDR in 2019 (per 100,000) | 24.732 (7.310, 59.661) | 326.782 (113.031, 704.876) | 346.929 (115.644, 757.408) | 92.392 (36.253, 195.732) | 123.308 (42.361, 276.496) |
| AAPC on ASDR, 1990-2019 | 1.686 (1.450, 1.923) | 1.452 (1.209, 1.696) | 1.372 (1.130, 1.616) | 1.454 (1.191, 1.717) | 2.355 (2.084, 2.627) |
| Honduras |  |  |  |  |  |
| ASDR in 2019 (per 100,000) | 79.716 (33.946, 154.519) | 398.816 (193.608, 686.033) | 371.812 (179.255, 643.400) | 211.696 (112.959, 341.244) | 145.665 (70.402, 252.868) |
| AAPC on ASDR, 1990-2019 | 3.933 (3.425, 4.443) | 2.642 (2.126, 3.161) | 2.877 (2.293, 3.464) | 2.356 (1.762, 2.952) | 4.102 (3.724, 4.480) |
| Hungary |  |  |  |  |  |
| ASDR in 2019 (per 100,000) | 255.312 (169.224, 363.784) | 624.640 (396.347, 922.131) | 316.791 (212.187, 450.096) | 149.049 (99.071, 208.918) | 185.112 (108.751, 273.725) |
| AAPC on ASDR, 1990-2019 | -1.075 (-1.474, -0.674) | -3.056 (-3.555, -2.555) | -4.144 (-4.544, -3.743) | -1.510 (-1.802, -1.218) | 0.674 (0.322, 1.028) |
| Iceland |  |  |  |  |  |
| ASDR in 2019 (per 100,000) | 20.915 (4.010, 47.585) | 40.301 (7.262, 97.693) | 15.557 (2.946, 37.180) | 13.026 (2.571, 28.797) | 19.985 (3.348, 49.101) |
| AAPC on ASDR, 1990-2019 | -2.923 (-3.255, -2.590) | -5.543 (-5.822, -5.264) | -5.069 (-5.575, -4.562) | -2.843 (-3.073, -2.613) | -0.310 (-0.579, -0.040) |
| India |  |  |  |  |  |
| ASDR in 2019 (per 100,000) | 73.208 (49.833, 98.899) | 1342.580 (971.779, 1748.486) | 671.926 (482.905, 874.984) | 1082.564 (801.218, 1375.075) | 299.999 (195.995, 417.233) |
| AAPC on ASDR, 1990-2019 | 3.245 (2.630, 3.865) | 2.541 (1.978, 3.107) | 1.812 (1.076, 2.555) | 1.440 (0.674, 2.211) | 4.520 (4.075, 4.966) |
| Indonesia |  |  |  |  |  |
| ASDR in 2019 (per 100,000) | 114.946 (69.169, 172.025) | 657.463 (427.074, 946.583) | 1012.302 (687.284, 1390.655) | 215.455 (145.346, 299.980) | 305.306 (187.533, 448.230) |
| AAPC on ASDR, 1990-2019 | 2.294 (2.164, 2.424) | 1.891 (1.703, 2.079) | 1.499 (1.237, 1.762) | -0.164 (-0.369, 0.042)* | 3.656 (3.399, 3.914) |
| Iran (Islamic Republic of) |  |  |  |  |  |
| ASDR in 2019 (per 100,000) | 113.233 (84.054, 143.698) | 1188.762 (884.239, 1505.871) | 587.935 (454.809, 727.853) | 213.341 (157.604, 272.533) | 384.138 (269.010, 522.025) |
| AAPC on ASDR, 1990-2019 | 0.551 (0.393, 0.709) | -1.862 (-2.008, -1.715) | -1.622 (-1.834, -1.409) | 0.015 (-0.047, 0.076)* | 2.616 (2.451, 2.781) |
| Iraq |  |  |  |  |  |
| ASDR in 2019 (per 100,000) | 205.761 (130.111, 301.617) | 2319.724 (1566.894, 3202.945) | 1656.178 (1128.179, 2290.587) | 132.143 (87.524, 191.500) | 669.151 (450.613, 915.204) |
| AAPC on ASDR, 1990-2019 | 1.090 (0.921, 1.260) | -0.224 (-0.385, -0.063) | 0.078 (-0.103, 0.260)* | -0.622 (-0.731, -0.513) | 1.238 (1.090, 1.386) |
| Ireland |  |  |  |  |  |
| ASDR in 2019 (per 100,000) | 45.321 (21.039, 77.017) | 94.049 (40.158, 173.604) | 40.404 (18.007, 74.077) | 39.370 (17.716, 67.062) | 42.961 (17.579, 80.395) |
| AAPC on ASDR, 1990-2019 | -3.779 (-4.113, -3.445) | -6.754 (-7.117, -6.390) | -5.886 (-6.192, -5.579) | -4.523 (-4.803, -4.243) | -0.935 (-1.271, -0.599) |
| Israel |  |  |  |  |  |
| ASDR in 2019 (per 100,000) | 120.784 (84.123, 162.708) | 190.794 (123.536, 269.252) | 131.501 (89.448, 184.140) | 81.128 (54.049, 113.533) | 204.367 (131.284, 290.128) |
| AAPC on ASDR, 1990-2019 | -0.865 (-1.205, -0.525) | -5.158 (-5.591, -4.722) | -3.376 (-4.093, -2.654) | -1.670 (-1.980, -1.358) | 0.725 (0.242, 1.211) |
| Italy |  |  |  |  |  |
| ASDR in 2019 (per 100,000) | 111.750 (78.451, 151.594) | 173.988 (109.286, 254.835) | 115.931 (79.603, 162.267) | 59.334 (39.166, 83.899) | 143.172 (85.732, 214.661) |
| AAPC on ASDR, 1990-2019 | -3.106 (-3.400, -2.812) | -4.346 (-4.628, -4.063) | -4.560 (-4.902, -4.217) | -2.938 (-3.212, -2.663) | -0.822 (-1.049, -0.594) |
| Jamaica |  |  |  |  |  |
| ASDR in 2019 (per 100,000) | 88.549 (48.763, 143.183) | 256.778 (138.794, 419.916) | 395.447 (219.409, 636.096) | 70.247 (38.769, 110.885) | 498.437 (279.171, 765.365) |
| AAPC on ASDR, 1990-2019 | 2.720 (1.287, 4.172) | 1.571 (-0.244, 3.419)* | 0.816 (-0.048, 1.686)* | 1.855 (0.790, 2.931) | 4.328 (3.405, 5.259) |
| Japan |  |  |  |  |  |
| ASDR in 2019 (per 100,000) | 68.957 (42.052, 102.273) | 100.905 (56.445, 158.414) | 148.468 (89.851, 226.063) | 30.932 (17.675, 48.369) | 69.903 (37.614, 112.931) |
| AAPC on ASDR, 1990-2019 | -0.700 (-0.818, -0.581) | -2.393 (-2.596, -2.189) | -2.118 (-2.305, -1.931) | -1.340 (-1.441, -1.240) | 0.069 (-0.094, 0.233)* |
| Jordan |  |  |  |  |  |
| ASDR in 2019 (per 100,000) | 121.599 (78.263, 177.827) | 883.152 (589.239, 1239.765) | 592.956 (416.920, 804.714) | 122.232 (82.389, 172.283) | 495.167 (331.754, 689.716) |
| AAPC on ASDR, 1990-2019 | 0.516 (0.133, 0.901) | -2.081 (-2.381, -1.779) | -2.227 (-2.633, -1.820) | -1.707 (-2.092, -1.321) | -0.586 (-0.942, -0.229) |
| Kazakhstan |  |  |  |  |  |
| ASDR in 2019 (per 100,000) | 128.454 (75.435, 192.895) | 998.119 (580.290, 1520.635) | 921.070 (551.658, 1377.742) | 283.349 (158.512, 444.593) | 226.718 (129.192, 344.895) |
| AAPC on ASDR, 1990-2019 | -2.039 (-2.343, -1.735) | -0.545 (-1.215, 0.129)* | 0.198 (-0.121, 0.517)* | 0.413 (-0.301, 1.132)* | 3.573 (3.407, 3.739) |
| Kenya |  |  |  |  |  |
| ASDR in 2019 (per 100,000) | 18.639 (9.857, 30.173) | 198.870 (105.616, 331.076) | 320.200 (175.232, 524.394) | 119.039 (70.810, 184.452) | 95.308 (48.220, 157.858) |
| AAPC on ASDR, 1990-2019 | 3.447 (3.256, 3.637) | 3.733 (3.489, 3.978) | 2.608 (2.395, 2.821) | 1.823 (1.670, 1.976) | 3.823 (3.544, 4.102) |
| Kiribati |  |  |  |  |  |
| ASDR in 2019 (per 100,000) | 29.031 (7.724, 73.826) | 415.109 (113.533, 1053.500) | 477.695 (137.062, 1180.677) | 94.362 (29.847, 224.845) | 252.064 (64.260, 654.714) |
| AAPC on ASDR, 1990-2019 | 1.092 (0.822, 1.363) | 0.904 (0.704, 1.103) | 0.652 (0.367, 0.937) | 0.169 (-0.022, 0.361)* | 2.844 (2.567, 3.121) |
| Kuwait |  |  |  |  |  |
| ASDR in 2019 (per 100,000) | 111.154 (74.441, 156.198) | 1284.852 (950.361, 1679.540) | 581.522 (424.461, 771.106) | 125.891 (90.499, 170.320) | 490.081 (315.550, 714.285) |
| AAPC on ASDR, 1990-2019 | -0.824 (-2.047, 0.414)* | -1.938 (-3.409, -0.445) | -0.447 (-1.386, 0.501)* | -0.298 (-0.810, 0.217)* | 0.167 (-0.365, 0.701)* |
| Kyrgyzstan |  |  |  |  |  |
| ASDR in 2019 (per 100,000) | 73.758 (41.229, 115.096) | 1198.893 (683.346, 1849.877) | 776.666 (447.762, 1182.862) | 217.341 (125.524, 349.795) | 99.262 (53.440, 158.052) |
| AAPC on ASDR, 1990-2019 | -1.799 (-2.227, -1.368) | 1.708 (0.903, 2.519) | 0.043 (-0.444, 0.533)* | -1.829 (-2.357, -1.299) | 3.052 (2.841, 3.263) |
| Lao People's Democratic Republic |  |  |  |  |  |
| ASDR in 2019 (per 100,000) | 63.931 (27.611, 116.982) | 354.075 (163.489, 622.991) | 500.325 (234.369, 863.720) | 183.030 (102.918, 292.676) | 105.770 (47.303, 190.117) |
| AAPC on ASDR, 1990-2019 | 2.080 (1.964, 2.196) | 2.203 (2.089, 2.317) | 2.149 (1.986, 2.313) | 0.471 (0.376, 0.566) | 3.311 (3.217, 3.404) |
| Latvia |  |  |  |  |  |
| ASDR in 2019 (per 100,000) | 89.179 (51.840, 141.834) | 520.498 (288.846, 840.533) | 312.531 (186.510, 490.004) | 28.939 (16.844, 45.396) | 94.182 (52.083, 148.849) |
| AAPC on ASDR, 1990-2019 | -3.145 (-5.220, -1.025) | -3.591 (-5.032, -2.128) | -3.763 (-5.029, -2.479) | -3.834 (-4.796, -2.863) | 0.515 (-0.286, 1.322)* |
| Lebanon |  |  |  |  |  |
| ASDR in 2019 (per 100,000) | 218.665 (135.440, 333.138) | 1691.927 (1046.409, 2417.031) | 299.590 (184.955, 435.448) | 179.125 (113.470, 260.461) | 372.100 (230.493, 548.072) |
| AAPC on ASDR, 1990-2019 | 1.356 (1.164, 1.548) | -0.786 (-1.010, -0.562) | -0.580 (-0.830, -0.330) | 0.417 (0.159, 0.675) | 1.605 (1.431, 1.778) |
| Lesotho |  |  |  |  |  |
| ASDR in 2019 (per 100,000) | 89.852 (39.100, 166.376) | 484.644 (229.947, 859.932) | 765.618 (372.439, 1312.550) | 396.692 (224.678, 646.391) | 397.570 (190.750, 695.622) |
| AAPC on ASDR, 1990-2019 | 3.811 (3.683, 3.938) | 4.077 (3.804, 4.350) | 3.333 (3.097, 3.569) | 1.448 (1.281, 1.616) | 5.412 (5.187, 5.637) |
| Liberia |  |  |  |  |  |
| ASDR in 2019 (per 100,000) | 30.573 (11.801, 62.804) | 284.466 (117.082, 562.763) | 309.245 (130.389, 597.045) | 111.375 (55.079, 196.809) | 106.581 (42.939, 215.411) |
| AAPC on ASDR, 1990-2019 | 1.866 (0.789, 2.955) | 1.780 (0.957, 2.609) | 1.507 (0.719, 2.301) | 1.774 (1.277, 2.273) | 3.202 (2.709, 3.698) |
| Libya |  |  |  |  |  |
| ASDR in 2019 (per 100,000) | 188.089 (107.286, 294.246) | 1543.671 (949.335, 2336.317) | 768.083 (473.086, 1155.778) | 226.696 (137.475, 340.199) | 455.259 (285.345, 662.219) |
| AAPC on ASDR, 1990-2019 | 0.449 (-0.033, 0.933)* | 0.511 (-0.019, 1.045)* | 0.519 (0.035, 1.006) | 0.909 (0.484, 1.337) | 2.957 (2.709, 3.206) |
| Lithuania |  |  |  |  |  |
| ASDR in 2019 (per 100,000) | 70.562 (39.480, 113.783) | 451.452 (235.301, 763.232) | 204.830 (113.066, 334.725) | 30.083 (16.404, 48.681) | 56.735 (29.079, 94.654) |
| AAPC on ASDR, 1990-2019 | -3.428 (-4.544, -2.299) | -3.638 (-4.707, -2.557) | -3.183 (-4.130, -2.226) | -5.212 (-5.952, -4.468) | -0.536 (-0.901, -0.168) |
| Luxembourg |  |  |  |  |  |
| ASDR in 2019 (per 100,000) | 76.274 (43.851, 119.027) | 104.248 (54.280, 175.603) | 64.073 (34.900, 106.584) | 46.990 (26.133, 74.178) | 90.449 (45.398, 153.484) |
| AAPC on ASDR, 1990-2019 | -3.525 (-3.763, -3.287) | -6.034 (-6.253, -5.815) | -6.282 (-6.450, -6.114) | -3.185 (-3.400, -2.969) | 0.554 (0.298, 0.810) |
| Madagascar |  |  |  |  |  |
| ASDR in 2019 (per 100,000) | 10.712 (4.058, 22.102) | 172.358 (67.596, 356.702) | 304.702 (125.683, 609.434) | 102.433 (51.423, 177.737) | 41.227 (16.396, 83.071) |
| AAPC on ASDR, 1990-2019 | 2.161 (1.586, 2.740) | 2.687 (2.035, 3.343) | 2.151 (1.602, 2.702) | 1.869 (1.254, 2.487) | 2.787 (2.242, 3.335) |
| Malawi |  |  |  |  |  |
| ASDR in 2019 (per 100,000) | 9.981 (3.741, 20.860) | 125.800 (47.133, 265.355) | 178.166 (69.280, 362.065) | 68.226 (32.328, 123.450) | 54.186 (20.196, 110.631) |
| AAPC on ASDR, 1990-2019 | 2.804 (2.472, 3.136) | 2.311 (1.929, 2.694) | 1.948 (1.702, 2.195) | 1.587 (1.345, 1.831) | 2.760 (2.496, 3.025) |
| Malaysia |  |  |  |  |  |
| ASDR in 2019 (per 100,000) | 96.183 (54.570, 151.472) | 720.100 (405.286, 1140.811) | 500.703 (301.538, 760.942) | 132.901 (78.507, 202.472) | 215.583 (126.705, 322.479) |
| AAPC on ASDR, 1990-2019 | -0.853 (-1.021, -0.684) | -1.552 (-1.775, -1.329) | -3.350 (-3.695, -3.005) | -3.089 (-3.387, -2.790) | -1.065 (-1.514, -0.613) |
| Maldives |  |  |  |  |  |
| ASDR in 2019 (per 100,000) | 24.263 (13.014, 39.986) | 282.145 (152.394, 467.943) | 182.563 (104.043, 289.346) | 110.791 (66.923, 170.006) | 109.132 (53.546, 182.438) |
| AAPC on ASDR, 1990-2019 | -0.014 (-0.546, 0.520)* | -1.374 (-1.923, -0.821) | -1.596 (-2.314, -0.872) | -3.211 (-3.794, -2.625) | 2.352 (1.596, 3.113) |
| Mali |  |  |  |  |  |
| ASDR in 2019 (per 100,000) | 17.590 (4.895, 40.723) | 181.202 (54.876, 402.512) | 237.368 (75.511, 521.033) | 169.299 (63.017, 341.984) | 67.737 (19.266, 155.622) |
| AAPC on ASDR, 1990-2019 | 2.674 (2.530, 2.817) | 1.840 (1.729, 1.950) | 1.470 (1.367, 1.573) | 1.805 (1.631, 1.980) | 3.065 (2.922, 3.208) |
| Malta |  |  |  |  |  |
| ASDR in 2019 (per 100,000) | 76.217 (47.068, 112.591) | 252.354 (146.197, 391.384) | 98.194 (59.192, 151.793) | 46.065 (28.026, 68.749) | 137.425 (76.025, 216.892) |
| AAPC on ASDR, 1990-2019 | -1.860 (-2.159, -1.560) | -3.971 (-4.237, -3.704) | -4.230 (-4.440, -4.020) | -2.461 (-2.747, -2.174) | -0.918 (-1.108, -0.728) |
| Marshall Islands |  |  |  |  |  |
| ASDR in 2019 (per 100,000) | 64.623 (17.072, 160.020) | 660.806 (207.493, 1493.495) | 522.268 (173.486, 1134.080) | 149.508 (52.899, 326.311) | 314.208 (80.065, 774.825) |
| AAPC on ASDR, 1990-2019 | 2.390 (2.139, 2.642) | 2.571 (2.306, 2.836) | 1.954 (1.733, 2.175) | -0.252 (-0.438, -0.065) | 4.225 (4.019, 4.432) |
| Mauritania |  |  |  |  |  |
| ASDR in 2019 (per 100,000) | 82.225 (37.424, 151.098) | 578.010 (299.191, 941.347) | 637.291 (329.507, 1023.033) | 236.872 (135.131, 374.457) | 206.851 (106.296, 341.154) |
| AAPC on ASDR, 1990-2019 | 2.620 (2.308, 2.932) | 1.245 (0.977, 1.515) | 0.942 (0.516, 1.370) | 0.641 (0.401, 0.882) | 3.036 (2.850, 3.223) |
| Mauritius |  |  |  |  |  |
| ASDR in 2019 (per 100,000) | 52.093 (23.550, 89.476) | 446.405 (195.191, 769.149) | 349.645 (160.062, 594.121) | 85.803 (35.308, 148.568) | 777.841 (373.864, 1211.915) |
| AAPC on ASDR, 1990-2019 | -0.315 (-0.797, 0.168)* | -2.917 (-3.430, -2.401) | -2.490 (-2.775, -2.203) | -1.234 (-1.686, -0.781) | 3.504 (3.096, 3.914) |
| Mexico |  |  |  |  |  |
| ASDR in 2019 (per 100,000) | 47.805 (32.625, 66.379) | 439.530 (281.632, 629.551) | 205.989 (141.247, 282.528) | 130.712 (85.671, 181.595) | 584.425 (382.271, 815.189) |
| AAPC on ASDR, 1990-2019 | -2.008 (-2.350, -1.665) | -0.561 (-0.929, -0.192) | -1.843 (-2.177, -1.507) | -1.559 (-1.967, -1.149) | 0.410 (0.148, 0.672) |
| Micronesia (Federated States of) |  |  |  |  |  |
| ASDR in 2019 (per 100,000) | 82.565 (18.998, 220.182) | 840.939 (198.570, 2064.102) | 635.224 (167.573, 1520.639) | 182.828 (52.378, 432.605) | 474.476 (106.261, 1210.851) |
| AAPC on ASDR, 1990-2019 | 2.292 (2.076, 2.509) | 2.239 (1.901, 2.577) | 1.457 (1.254, 1.659) | -0.304 (-0.534, -0.073) | 5.044 (4.930, 5.157) |
| Monaco |  |  |  |  |  |
| ASDR in 2019 (per 100,000) | 212.354 (109.530, 350.425) | 163.195 (75.316, 288.996) | 96.486 (48.326, 163.638) | 48.815 (25.235, 81.123) | 67.986 (32.278, 116.098) |
| AAPC on ASDR, 1990-2019 | 1.959 (1.577, 2.342) | -1.627 (-2.022, -1.231) | -1.613 (-1.983, -1.241) | 0.483 (0.094, 0.874) | 2.672 (2.276, 3.071) |
| Mongolia |  |  |  |  |  |
| ASDR in 2019 (per 100,000) | 234.344 (138.926, 367.575) | 1812.310 (1117.496, 2690.689) | 2033.072 (1271.974, 3018.474) | 154.447 (95.399, 238.275) | 84.564 (49.813, 127.503) |
| AAPC on ASDR, 1990-2019 | 1.609 (1.029, 2.192) | 1.814 (1.369, 2.261) | 3.169 (2.905, 3.434) | 0.166 (-0.104, 0.437)* | 4.506 (4.355, 4.658) |
| Montenegro |  |  |  |  |  |
| ASDR in 2019 (per 100,000) | 342.221 (220.070, 492.469) | 751.559 (483.511, 1087.206) | 932.878 (649.584, 1276.311) | 49.351 (32.037, 70.324) | 232.331 (133.617, 344.603) |
| AAPC on ASDR, 1990-2019 | -0.297 (-0.587, -0.007) | -0.814 (-1.136, -0.492) | -1.081 (-1.226, -0.935) | -1.049 (-1.275, -0.823) | 1.158 (0.874, 1.442) |
| Morocco |  |  |  |  |  |
| ASDR in 2019 (per 100,000) | 158.716 (91.793, 247.092) | 2053.539 (1322.528, 2946.067) | 1008.636 (667.658, 1445.730) | 227.088 (144.430, 330.938) | 376.702 (242.443, 534.918) |
| AAPC on ASDR, 1990-2019 | 3.018 (2.912, 3.125) | 2.226 (1.874, 2.580) | 2.336 (2.211, 2.462) | 2.289 (1.952, 2.628) | 5.322 (5.134, 5.510) |
| Mozambique |  |  |  |  |  |
| ASDR in 2019 (per 100,000) | 10.258 (3.482, 22.775) | 103.229 (36.678, 225.031) | 230.109 (85.568, 496.802) | 56.328 (26.099, 104.556) | 47.026 (15.562, 101.565) |
| AAPC on ASDR, 1990-2019 | 4.636 (4.467, 4.806) | 4.116 (3.933, 4.300) | 3.690 (3.463, 3.918) | 2.909 (2.799, 3.020) | 4.253 (4.036, 4.471) |
| Myanmar |  |  |  |  |  |
| ASDR in 2019 (per 100,000) | 105.187 (52.946, 181.870) | 391.115 (219.326, 609.244) | 974.973 (565.714, 1479.694) | 438.564 (290.091, 614.558) | 225.131 (120.974, 358.165) |
| AAPC on ASDR, 1990-2019 | 2.400 (2.266, 2.533) | 1.454 (1.356, 1.553) | 1.705 (1.589, 1.821) | 0.815 (0.697, 0.933) | 3.370 (3.245, 3.494) |
| Namibia |  |  |  |  |  |
| ASDR in 2019 (per 100,000) | 42.735 (21.883, 71.209) | 507.644 (266.654, 838.934) | 651.320 (351.735, 1035.517) | 269.950 (150.197, 437.456) | 323.851 (163.289, 522.971) |
| AAPC on ASDR, 1990-2019 | 2.799 (2.567, 3.032) | 2.075 (1.752, 2.398) | 1.299 (1.017, 1.581) | 0.317 (0.082, 0.553) | 3.370 (3.073, 3.667) |
| Nauru |  |  |  |  |  |
| ASDR in 2019 (per 100,000) | 45.831 (9.610, 125.703) | 532.755 (125.787, 1377.563) | 364.045 (93.964, 902.532) | 77.203 (18.923, 186.161) | 277.813 (49.769, 772.529) |
| AAPC on ASDR, 1990-2019 | -0.555 (-1.142, 0.035)* | -0.078 (-0.533, 0.380)* | -0.816 (-1.128, -0.502) | -2.264 (-2.450, -2.077) | 2.072 (1.673, 2.472) |
| Nepal |  |  |  |  |  |
| ASDR in 2019 (per 100,000) | 56.940 (27.349, 99.166) | 761.311 (405.156, 1211.448) | 552.755 (301.094, 874.130) | 1541.115 (968.305, 2217.256) | 161.774 (83.526, 264.795) |
| AAPC on ASDR, 1990-2019 | 4.389 (4.093, 4.687) | 4.300 (3.995, 4.606) | 3.085 (2.860, 3.311) | 3.102 (2.920, 3.284) | 6.177 (5.725, 6.632) |
| Netherlands |  |  |  |  |  |
| ASDR in 2019 (per 100,000) | 123.496 (78.788, 179.601) | 121.341 (69.654, 190.656) | 90.463 (55.031, 140.193) | 86.845 (53.430, 128.019) | 81.029 (45.364, 128.286) |
| AAPC on ASDR, 1990-2019 | -2.790 (-3.048, -2.531) | -6.041 (-6.328, -5.754) | -4.147 (-4.357, -3.937) | -2.297 (-2.575, -2.019) | -2.067 (-2.256, -1.878) |
| New Zealand |  |  |  |  |  |
| ASDR in 2019 (per 100,000) | 23.268 (4.053, 50.320) | 54.107 (8.601, 126.390) | 25.342 (4.196, 58.767) | 21.695 (3.604, 47.932) | 24.658 (3.841, 56.980) |
| AAPC on ASDR, 1990-2019 | -2.653 (-2.973, -2.333) | -5.029 (-5.381, -4.676) | -3.903 (-4.250, -3.556) | -2.340 (-2.620, -2.059) | -0.805 (-1.094, -0.515) |
| Nicaragua |  |  |  |  |  |
| ASDR in 2019 (per 100,000) | 29.981 (14.620, 51.335) | 355.432 (180.482, 595.519) | 182.674 (94.955, 303.300) | 112.817 (66.570, 177.117) | 227.322 (108.716, 387.181) |
| AAPC on ASDR, 1990-2019 | 3.224 (2.611, 3.842) | 3.526 (2.804, 4.253) | 1.455 (1.156, 1.756) | 3.380 (2.672, 4.093) | 5.315 (4.549, 6.087) |
| Niger |  |  |  |  |  |
| ASDR in 2019 (per 100,000) | 18.567 (3.947, 51.836) | 171.113 (38.197, 459.945) | 215.437 (51.158, 567.107) | 116.829 (32.342, 290.637) | 47.694 (9.492, 134.108) |
| AAPC on ASDR, 1990-2019 | 1.643 (1.330, 1.957) | 1.082 (0.859, 1.306) | 0.906 (0.667, 1.145) | 0.914 (0.693, 1.135) | 2.551 (2.147, 2.957) |
| Nigeria |  |  |  |  |  |
| ASDR in 2019 (per 100,000) | 50.092 (27.171, 78.075) | 496.642 (275.658, 775.398) | 570.423 (333.238, 872.308) | 207.269 (131.136, 305.273) | 189.138 (107.074, 292.397) |
| AAPC on ASDR, 1990-2019 | 3.660 (3.449, 3.872) | 2.496 (2.148, 2.845) | 2.043 (1.620, 2.467) | 1.915 (1.797, 2.032) | 3.961 (3.804, 4.118) |
| Niue |  |  |  |  |  |
| ASDR in 2019 (per 100,000) | 45.528 (8.583, 113.945) | 354.318 (64.569, 887.095) | 223.587 (45.110, 546.974) | 62.460 (12.939, 147.267) | 335.620 (58.876, 823.890) |
| AAPC on ASDR, 1990-2019 | 0.423 (0.161, 0.686) | -0.003 (-0.264, 0.259)* | -1.017 (-1.261, -0.771) | -1.732 (-1.914, -1.549) | 3.056 (2.846, 3.266) |
| North Macedonia |  |  |  |  |  |
| ASDR in 2019 (per 100,000) | 332.501 (211.553, 488.051) | 1108.497 (743.878, 1576.476) | 1546.229 (1103.146, 2087.473) | 158.992 (109.574, 224.790) | 412.188 (264.462, 582.028) |
| AAPC on ASDR, 1990-2019 | 0.701 (0.530, 0.872) | -1.063 (-1.364, -0.762) | -0.916 (-1.157, -0.675) | -1.880 (-2.095, -1.665) | 2.428 (2.298, 2.558) |
| Northern Mariana Islands |  |  |  |  |  |
| ASDR in 2019 (per 100,000) | 94.125 (44.512, 165.858) | 359.580 (159.060, 657.544) | 272.152 (126.699, 479.850) | 65.572 (30.154, 116.478) | 239.288 (108.178, 420.796) |
| AAPC on ASDR, 1990-2019 | -2.057 (-2.729, -1.380) | -0.513 (-1.256, 0.236)* | -2.567 (-3.508, -1.617) | -3.014 (-3.747, -2.276) | 0.252 (-1.237, 1.764)* |
| Norway |  |  |  |  |  |
| ASDR in 2019 (per 100,000) | 26.590 (9.142, 50.369) | 45.317 (14.524, 94.317) | 29.307 (10.022, 59.736) | 24.580 (8.236, 46.798) | 32.809 (10.183, 67.103) |
| AAPC on ASDR, 1990-2019 | -3.776 (-4.205, -3.345) | -7.850 (-8.139, -7.559) | -6.249 (-6.526, -5.971) | -2.336 (-2.858, -1.812) | -2.380 (-2.572, -2.187) |
| Oman |  |  |  |  |  |
| ASDR in 2019 (per 100,000) | 94.130 (57.688, 147.676) | 2377.359 (1628.221, 3210.040) | 932.812 (621.363, 1318.177) | 217.632 (134.309, 319.495) | 671.373 (451.968, 932.844) |
| AAPC on ASDR, 1990-2019 | 1.534 (1.189, 1.880) | -0.244 (-0.636, 0.150)* | -0.002 (-0.335, 0.332)* | -0.293 (-0.717, 0.133)* | 3.130 (2.792, 3.469) |
| Pakistan |  |  |  |  |  |
| ASDR in 2019 (per 100,000) | 136.318 (77.328, 211.795) | 1423.808 (893.465, 2089.265) | 903.030 (564.594, 1321.064) | 738.389 (507.744, 1020.416) | 359.276 (208.045, 538.471) |
| AAPC on ASDR, 1990-2019 | 4.253 (4.035, 4.472) | 4.920 (4.758, 5.083) | 3.650 (3.470, 3.831) | 2.147 (1.952, 2.342) | 6.295 (6.171, 6.418) |
| Palau |  |  |  |  |  |
| ASDR in 2019 (per 100,000) | 60.925 (0.245, 164.790) | 386.476 (1.403, 1068.725) | 227.983 (0.899, 622.446) | 74.525 (0.339, 199.028) | 312.592 (0.949, 843.270) |
| AAPC on ASDR, 1990-2019 | 0.014 (-1.712, 1.770)* | -0.227 (-1.947, 1.523)* | -0.621 (-2.350, 1.138)* | -1.127 (-2.951, 0.731)* | 1.675 (0.031, 3.346) |
| Palestine |  |  |  |  |  |
| ASDR in 2019 (per 100,000) | 196.566 (128.937, 280.852) | 1457.893 (948.191, 2014.119) | 864.408 (582.744, 1180.758) | 150.455 (95.717, 219.287) | 721.311 (489.247, 980.318) |
| AAPC on ASDR, 1990-2019 | 2.283 (2.003, 2.563) | 0.645 (0.342, 0.950) | 0.788 (0.399, 1.178) | -0.248 (-0.577, 0.083)* | 3.610 (3.358, 3.862) |
| Panama |  |  |  |  |  |
| ASDR in 2019 (per 100,000) | 37.887 (20.311, 61.580) | 187.087 (96.874, 312.507) | 156.985 (83.871, 256.420) | 46.442 (24.045, 74.150) | 246.013 (129.332, 394.472) |
| AAPC on ASDR, 1990-2019 | -0.944 (-1.443, -0.443) | -1.721 (-2.530, -0.905) | -1.714 (-2.314, -1.111) | -1.066 (-1.721, -0.406) | 2.927 (2.495, 3.360) |
| Papua New Guinea |  |  |  |  |  |
| ASDR in 2019 (per 100,000) | 33.544 (6.808, 94.670) | 260.956 (56.223, 707.849) | 240.613 (54.526, 658.199) | 266.722 (67.817, 701.684) | 142.988 (31.026, 399.923) |
| AAPC on ASDR, 1990-2019 | 1.980 (1.878, 2.082) | 2.220 (1.964, 2.476) | 1.485 (1.301, 1.668) | 0.675 (0.520, 0.831) | 2.682 (2.571, 2.793) |
| Paraguay |  |  |  |  |  |
| ASDR in 2019 (per 100,000) | 50.160 (26.538, 82.490) | 265.245 (145.956, 439.037) | 224.853 (127.031, 367.997) | 54.097 (30.908, 87.231) | 184.975 (93.936, 310.601) |
| AAPC on ASDR, 1990-2019 | 2.474 (1.912, 3.038) | 1.018 (0.449, 1.589) | -0.208 (-0.652, 0.238)* | 1.043 (0.245, 1.847) | 4.585 (4.294, 4.878) |
| Peru |  |  |  |  |  |
| ASDR in 2019 (per 100,000) | 73.335 (42.284, 114.196) | 277.969 (163.507, 432.542) | 239.602 (146.972, 370.986) | 62.632 (38.234, 96.927) | 183.378 (113.603, 264.417) |
| AAPC on ASDR, 1990-2019 | -0.405 (-1.185, 0.381)* | -1.386 (-2.161, -0.606) | -1.542 (-2.197, -0.883) | -0.515 (-1.119, 0.094)* | 2.936 (2.574, 3.300) |
| Philippines |  |  |  |  |  |
| ASDR in 2019 (per 100,000) | 85.525 (55.566, 124.552) | 673.991 (438.268, 967.741) | 590.909 (388.162, 826.451) | 177.747 (127.077, 244.546) | 204.529 (127.355, 305.493) |
| AAPC on ASDR, 1990-2019 | -1.305 (-1.571, -1.039) | 3.013 (2.636, 3.392) | 2.424 (2.071, 2.780) | -1.043 (-1.237, -0.849) | 1.312 (1.116, 1.510) |
| Poland |  |  |  |  |  |
| ASDR in 2019 (per 100,000) | 289.013 (202.592, 397.631) | 594.120 (395.658, 841.505) | 393.417 (284.273, 523.381) | 104.326 (73.695, 140.876) | 219.956 (139.225, 312.670) |
| AAPC on ASDR, 1990-2019 | -1.123 (-1.633, -0.610) | -4.169 (-4.596, -3.739) | -2.943 (-3.304, -2.580) | -2.687 (-2.859, -2.515) | 0.638 (0.378, 0.900) |
| Portugal |  |  |  |  |  |
| ASDR in 2019 (per 100,000) | 42.409 (21.772, 69.866) | 71.268 (33.814, 127.341) | 80.259 (40.353, 140.377) | 31.553 (15.597, 53.324) | 81.393 (36.470, 144.572) |
| AAPC on ASDR, 1990-2019 | -1.659 (-1.940, -1.377) | -4.966 (-5.352, -4.578) | -6.021 (-6.276, -5.764) | -3.195 (-3.475, -2.913) | -1.050 (-1.211, -0.888) |
| Puerto Rico |  |  |  |  |  |
| ASDR in 2019 (per 100,000) | 14.285 (2.821, 29.514) | 76.642 (14.700, 168.916) | 32.713 (6.538, 70.333) | 18.329 (3.779, 37.527) | 142.800 (27.890, 294.951) |
| AAPC on ASDR, 1990-2019 | -2.305 (-2.739, -1.869) | -3.783 (-4.346, -3.217) | -2.676 (-3.099, -2.251) | -1.333 (-1.680, -0.985) | -0.142 (-0.404, 0.120)* |
| Qatar |  |  |  |  |  |
| ASDR in 2019 (per 100,000) | 206.880 (124.010, 322.527) | 1802.316 (1263.587, 2464.418) | 563.347 (390.034, 792.175) | 278.187 (192.964, 398.994) | 1306.305 (861.836, 1873.409) |
| AAPC on ASDR, 1990-2019 | -0.364 (-1.009, 0.285)* | -2.711 (-3.520, -1.896) | -2.152 (-3.017, -1.280) | -1.370 (-2.350, -0.380) | 0.566 (0.026, 1.108) |
| Republic of Korea |  |  |  |  |  |
| ASDR in 2019 (per 100,000) | 167.035 (115.056, 227.092) | 172.008 (112.246, 243.998) | 327.952 (231.441, 449.244) | 103.122 (69.687, 143.522) | 219.989 (141.297, 312.300) |
| AAPC on ASDR, 1990-2019 | -0.243 (-0.497, 0.012)* | -5.196 (-5.471, -4.921) | -5.029 (-5.387, -4.670) | -1.145 (-1.490, -0.800) | 0.079 (-0.174, 0.333)* |
| Republic of Moldova |  |  |  |  |  |
| ASDR in 2019 (per 100,000) | 81.531 (41.740, 131.024) | 787.204 (408.089, 1287.004) | 422.430 (224.009, 674.295) | 61.413 (31.203, 102.907) | 100.969 (49.861, 166.627) |
| AAPC on ASDR, 1990-2019 | -2.388 (-3.840, -0.913) | -1.496 (-3.116, 0.152)* | -2.254 (-3.919, -0.559) | -4.480 (-5.633, -3.313) | 1.041 (0.362, 1.726) |
| Romania |  |  |  |  |  |
| ASDR in 2019 (per 100,000) | 164.033 (107.343, 237.131) | 617.187 (383.443, 913.769) | 533.667 (354.275, 761.410) | 90.566 (59.005, 130.579) | 115.217 (68.034, 173.721) |
| AAPC on ASDR, 1990-2019 | -0.083 (-0.798, 0.638)* | -2.030 (-2.631, -1.424) | -2.104 (-2.514, -1.694) | -3.459 (-3.948, -2.968) | 1.172 (0.942, 1.402) |
| Russian Federation |  |  |  |  |  |
| ASDR in 2019 (per 100,000) | 79.696 (41.049, 125.580) | 663.504 (324.894, 1107.913) | 425.562 (221.173, 686.686) | 49.238 (24.216, 80.369) | 70.200 (34.367, 115.223) |
| AAPC on ASDR, 1990-2019 | -3.063 (-4.592, -1.509) | -2.516 (-3.911, -1.100) | -2.773 (-4.074, -1.454) | -3.819 (-4.840, -2.787) | 0.215 (-0.149, 0.579)* |
| Rwanda |  |  |  |  |  |
| ASDR in 2019 (per 100,000) | 26.344 (9.152, 56.806) | 189.702 (71.915, 388.533) | 340.773 (132.733, 676.347) | 168.380 (77.399, 315.874) | 90.448 (34.297, 180.883) |
| AAPC on ASDR, 1990-2019 | 2.002 (1.666, 2.339) | 1.021 (0.657, 1.386) | -0.092 (-0.457, 0.274)* | 0.171 (-0.123, 0.466)* | 1.574 (1.283, 1.865) |
| Saint Kitts and Nevis |  |  |  |  |  |
| ASDR in 2019 (per 100,000) | 24.492 (9.470, 45.161) | 199.661 (71.207, 382.791) | 260.048 (97.373, 492.585) | 29.824 (12.418, 53.537) | 247.135 (96.318, 445.270) |
| AAPC on ASDR, 1990-2019 | -0.411 (-0.964, 0.146)* | -2.717 (-3.283, -2.147) | -2.684 (-3.228, -2.136) | -0.423 (-1.020, 0.178)* | 0.774 (0.366, 1.185) |
| Saint Lucia |  |  |  |  |  |
| ASDR in 2019 (per 100,000) | 74.697 (29.659, 135.315) | 334.181 (132.537, 587.337) | 490.088 (193.267, 890.502) | 116.432 (43.404, 218.347) | 667.548 (289.826, 1069.833) |
| AAPC on ASDR, 1990-2019 | 0.747 (0.413, 1.081) | -1.211 (-1.566, -0.854) | -0.912 (-1.219, -0.604) | 0.335 (0.064, 0.606) | 1.947 (1.441, 2.457) |
| Saint Vincent and the Grenadines |  |  |  |  |  |
| ASDR in 2019 (per 100,000) | 62.579 (22.579, 115.704) | 606.629 (219.228, 1089.299) | 519.014 (189.663, 964.168) | 59.573 (19.921, 117.263) | 748.129 (301.046, 1210.722) |
| AAPC on ASDR, 1990-2019 | 1.567 (1.013, 2.124) | 0.021 (-0.333, 0.377)* | 0.400 (0.035, 0.767) | 1.335 (0.869, 1.804) | 2.566 (2.148, 2.985) |
| Samoa |  |  |  |  |  |
| ASDR in 2019 (per 100,000) | 26.699 (7.284, 66.177) | 498.066 (132.100, 1169.056) | 393.814 (107.096, 922.187) | 128.165 (36.764, 309.576) | 224.517 (56.249, 548.916) |
| AAPC on ASDR, 1990-2019 | 0.596 (0.472, 0.720) | 0.678 (0.616, 0.741) | 0.035 (-0.021, 0.090)* | -1.214 (-1.286, -1.142) | 2.007 (1.898, 2.116) |
| San Marino |  |  |  |  |  |
| ASDR in 2019 (per 100,000) | 82.568 (29.487, 168.191) | 88.367 (29.769, 185.145) | 69.466 (24.955, 135.788) | 34.801 (12.292, 65.132) | 65.409 (23.765, 120.168) |
| AAPC on ASDR, 1990-2019 | -1.289 (-1.475, -1.102) | -2.704 (-2.981, -2.427) | -2.533 (-2.657, -2.410) | -1.511 (-1.693, -1.328) | 0.440 (0.269, 0.611) |
| Sao Tome and Principe |  |  |  |  |  |
| ASDR in 2019 (per 100,000) | 90.088 (40.646, 163.053) | 507.528 (236.110, 889.103) | 594.920 (278.676, 1044.975) | 371.106 (191.627, 615.223) | 109.235 (48.857, 192.394) |
| AAPC on ASDR, 1990-2019 | 4.528 (4.018, 5.042) | 4.535 (3.976, 5.098) | 3.786 (3.205, 4.370) | 2.803 (2.393, 3.215) | 5.266 (5.072, 5.461) |
| Saudi Arabia |  |  |  |  |  |
| ASDR in 2019 (per 100,000) | 106.150 (67.263, 155.901) | 2375.933 (1670.802, 3197.769) | 1366.024 (945.927, 1853.003) | 305.137 (211.171, 423.180) | 454.957 (300.332, 638.819) |
| AAPC on ASDR, 1990-2019 | 2.105 (1.914, 2.296) | 1.848 (1.574, 2.123) | 1.223 (1.079, 1.367) | 0.485 (0.297, 0.673) | 2.789 (2.571, 3.008) |
| Senegal |  |  |  |  |  |
| ASDR in 2019 (per 100,000) | 54.453 (22.823, 107.007) | 371.066 (159.617, 695.496) | 419.468 (184.892, 770.852) | 195.882 (101.943, 334.506) | 157.538 (67.044, 299.871) |
| AAPC on ASDR, 1990-2019 | 2.394 (1.687, 3.105) | 1.633 (1.084, 2.186) | 1.588 (1.057, 2.121) | 0.827 (0.416, 1.240) | 3.233 (2.885, 3.582) |
| Serbia |  |  |  |  |  |
| ASDR in 2019 (per 100,000) | 351.572 (229.398, 506.469) | 869.095 (574.039, 1245.048) | 885.147 (622.489, 1205.174) | 161.131 (110.398, 221.178) | 300.649 (189.133, 429.915) |
| AAPC on ASDR, 1990-2019 | 0.449 (0.105, 0.795) | -1.266 (-1.724, -0.805) | -1.784 (-2.017, -1.551) | -0.994 (-1.251, -0.737) | 1.762 (1.484, 2.040) |
| Seychelles |  |  |  |  |  |
| ASDR in 2019 (per 100,000) | 82.706 (40.105, 143.002) | 498.307 (228.276, 849.746) | 409.268 (195.096, 682.327) | 115.564 (51.185, 196.368) | 340.257 (170.114, 543.090) |
| AAPC on ASDR, 1990-2019 | -0.013 (-0.328, 0.304)* | -0.951 (-1.308, -0.593) | -1.330 (-1.573, -1.087) | -0.507 (-0.939, -0.073) | 3.191 (3.012, 3.369) |
| Sierra Leone |  |  |  |  |  |
| ASDR in 2019 (per 100,000) | 31.143 (11.464, 66.629) | 288.772 (109.746, 606.377) | 335.074 (126.115, 695.484) | 144.079 (65.322, 269.696) | 70.371 (25.063, 147.159) |
| AAPC on ASDR, 1990-2019 | 2.192 (1.722, 2.664) | 1.825 (1.561, 2.089) | 1.838 (1.647, 2.029) | 1.119 (0.607, 1.634) | 3.331 (3.108, 3.556) |
| Singapore |  |  |  |  |  |
| ASDR in 2019 (per 100,000) | 97.890 (58.832, 145.788) | 244.267 (135.881, 370.523) | 149.632 (86.223, 225.725) | 56.108 (31.390, 86.629) | 138.147 (72.223, 225.344) |
| AAPC on ASDR, 1990-2019 | -2.914 (-3.324, -2.502) | -4.129 (-4.454, -3.803) | -4.588 (-4.998, -4.176) | -4.984 (-5.271, -4.697) | -1.779 (-2.314, -1.241) |
| Slovakia |  |  |  |  |  |
| ASDR in 2019 (per 100,000) | 154.921 (97.118, 230.763) | 726.872 (437.947, 1101.972) | 350.805 (230.507, 508.177) | 67.553 (43.726, 97.580) | 164.767 (98.174, 245.918) |
| AAPC on ASDR, 1990-2019 | -2.695 (-3.047, -2.341) | -3.732 (-4.246, -3.215) | -3.513 (-3.835, -3.190) | -1.969 (-2.331, -1.606) | -0.515 (-0.697, -0.333) |
| Slovenia |  |  |  |  |  |
| ASDR in 2019 (per 100,000) | 154.676 (97.036, 232.368) | 225.663 (133.560, 345.921) | 174.165 (111.534, 255.959) | 56.819 (36.690, 81.766) | 140.641 (82.764, 212.051) |
| AAPC on ASDR, 1990-2019 | -2.080 (-2.512, -1.645) | -4.335 (-4.660, -4.008) | -5.058 (-5.365, -4.751) | -3.782 (-4.057, -3.506) | -0.251 (-0.503, 0.002)* |
| Solomon Islands |  |  |  |  |  |
| ASDR in 2019 (per 100,000) | 45.558 (9.493, 138.290) | 628.693 (162.021, 1650.925) | 462.330 (118.249, 1226.111) | 170.009 (48.933, 417.101) | 155.467 (39.055, 417.850) |
| AAPC on ASDR, 1990-2019 | 3.204 (2.964, 3.445) | 3.075 (2.751, 3.400) | 2.915 (2.664, 3.167) | 1.869 (1.648, 2.091) | 4.896 (4.634, 5.159) |
| Somalia |  |  |  |  |  |
| ASDR in 2019 (per 100,000) | 3.601 (0.546, 11.979) | 61.491 (10.768, 190.466) | 85.693 (15.872, 261.584) | 42.591 (8.796, 122.815) | 20.483 (3.154, 62.009) |
| AAPC on ASDR, 1990-2019 | 0.418 (-0.104, 0.942)* | 1.243 (0.630, 1.860) | 0.201 (-0.285, 0.689)* | 0.212 (-0.285, 0.712)* | 1.216 (0.707, 1.728) |
| South Africa |  |  |  |  |  |
| ASDR in 2019 (per 100,000) | 140.116 (94.806, 200.005) | 497.061 (335.680, 678.700) | 574.739 (410.975, 760.186) | 263.560 (183.760, 355.883) | 567.665 (380.677, 758.344) |
| AAPC on ASDR, 1990-2019 | -0.293 (-0.775, 0.191)* | 0.022 (-0.888, 0.940)* | -0.112 (-0.940, 0.722)* | -0.270 (-0.795, 0.259)* | 2.610 (2.046, 3.177) |
| South Sudan |  |  |  |  |  |
| ASDR in 2019 (per 100,000) | 24.674 (8.219, 52.053) | 172.775 (63.131, 351.872) | 258.838 (95.777, 516.676) | 116.977 (54.300, 215.799) | 75.366 (28.059, 150.697) |
| AAPC on ASDR, 1990-2019 | 1.412 (1.182, 1.642) | 1.669 (1.500, 1.839) | 0.718 (0.522, 0.915) | 0.606 (0.417, 0.794) | 2.111 (1.934, 2.289) |
| Spain |  |  |  |  |  |
| ASDR in 2019 (per 100,000) | 70.053 (41.315, 107.294) | 92.378 (49.012, 154.130) | 60.180 (33.824, 98.755) | 47.457 (26.689, 74.482) | 84.576 (42.177, 145.795) |
| AAPC on ASDR, 1990-2019 | -2.207 (-2.595, -1.819) | -4.375 (-4.793, -3.956) | -5.054 (-5.359, -4.747) | -2.917 (-3.245, -2.588) | -1.314 (-1.478, -1.150) |
| Sri Lanka |  |  |  |  |  |
| ASDR in 2019 (per 100,000) | 48.455 (25.641, 81.131) | 446.179 (249.972, 712.089) | 328.862 (194.532, 506.817) | 134.125 (79.116, 210.851) | 373.908 (203.722, 590.425) |
| AAPC on ASDR, 1990-2019 | 2.051 (1.525, 2.580) | -0.297 (-1.009, 0.421)* | -0.076 (-0.684, 0.535)* | 0.159 (-0.485, 0.808)* | 5.151 (4.703, 5.601) |
| Sudan |  |  |  |  |  |
| ASDR in 2019 (per 100,000) | 67.675 (29.982, 131.439) | 1833.516 (986.703, 2951.942) | 1010.375 (530.493, 1680.057) | 296.732 (160.393, 481.772) | 221.255 (120.738, 350.895) |
| AAPC on ASDR, 1990-2019 | 5.097 (4.994, 5.200) | 3.810 (3.716, 3.905) | 3.984 (3.855, 4.113) | 3.180 (3.052, 3.309) | 7.435 (7.252, 7.619) |
| Suriname |  |  |  |  |  |
| ASDR in 2019 (per 100,000) | 90.731 (37.320, 166.073) | 630.378 (264.109, 1117.996) | 678.068 (294.525, 1199.954) | 84.224 (33.944, 157.067) | 526.051 (242.828, 857.487) |
| AAPC on ASDR, 1990-2019 | 0.778 (-0.053, 1.615)* | -1.077 (-2.207, 0.067)* | -0.123 (-0.888, 0.649)* | -0.583 (-1.608, 0.453)* | 2.564 (2.088, 3.044) |
| Sweden |  |  |  |  |  |
| ASDR in 2019 (per 100,000) | 14.668 (3.465, 31.924) | 40.777 (9.271, 95.590) | 21.787 (5.243, 50.359) | 12.743 (3.075, 27.753) | 21.609 (4.752, 50.099) |
| AAPC on ASDR, 1990-2019 | -4.354 (-4.536, -4.172) | -7.244 (-7.411, -7.077) | -5.941 (-6.149, -5.733) | -3.405 (-3.534, -3.277) | -2.762 (-3.009, -2.514) |
| Switzerland |  |  |  |  |  |
| ASDR in 2019 (per 100,000) | 56.044 (32.762, 85.416) | 90.918 (47.773, 151.387) | 44.468 (24.905, 72.909) | 33.899 (19.257, 53.279) | 64.048 (32.275, 109.055) |
| AAPC on ASDR, 1990-2019 | -4.452 (-4.729, -4.175) | -6.009 (-6.363, -5.654) | -5.681 (-5.912, -5.449) | -3.711 (-3.872, -3.550) | -2.130 (-2.323, -1.936) |
| Syrian Arab Republic |  |  |  |  |  |
| ASDR in 2019 (per 100,000) | 98.281 (59.944, 153.286) | 2514.945 (1590.840, 3701.810) | 851.819 (556.524, 1225.423) | 193.294 (122.064, 292.453) | 326.920 (205.715, 473.562) |
| AAPC on ASDR, 1990-2019 | 0.501 (-0.034, 1.039)* | -0.446 (-0.917, 0.027)* | -1.297 (-1.671, -0.921) | -0.084 (-0.497, 0.331)* | 1.045 (0.833, 1.259) |
| Taiwan (Province of China) |  |  |  |  |  |
| ASDR in 2019 (per 100,000) | 185.736 (121.940, 269.934) | 247.538 (157.606, 361.894) | 335.650 (227.379, 476.334) | 102.871 (69.045, 146.039) | 304.410 (197.842, 431.041) |
| AAPC on ASDR, 1990-2019 | 0.841 (0.206, 1.480) | -1.345 (-1.649, -1.039) | -2.960 (-3.217, -2.703) | -1.239 (-1.557, -0.920) | 1.032 (0.701, 1.364) |
| Tajikistan |  |  |  |  |  |
| ASDR in 2019 (per 100,000) | 90.482 (43.737, 153.642) | 2152.068 (1068.810, 3486.278) | 1200.792 (602.606, 1954.727) | 256.346 (122.281, 480.189) | 316.616 (158.812, 497.058) |
| AAPC on ASDR, 1990-2019 | 0.154 (-0.405, 0.716)* | 3.762 (3.484, 4.040) | 2.923 (2.621, 3.226) | 0.709 (0.530, 0.888) | 5.999 (5.611, 6.387) |
| Thailand |  |  |  |  |  |
| ASDR in 2019 (per 100,000) | 154.422 (96.507, 232.130) | 349.375 (214.773, 539.538) | 520.347 (345.878, 757.629) | 163.859 (110.818, 231.915) | 248.366 (157.517, 353.100) |
| AAPC on ASDR, 1990-2019 | -0.502 (-1.069, 0.069)* | -0.590 (-0.992, -0.187) | -1.039 (-1.423, -0.652) | -3.006 (-3.307, -2.704) | 1.948 (1.528, 2.368) |
| Timor-Leste |  |  |  |  |  |
| ASDR in 2019 (per 100,000) | 46.831 (16.939, 96.006) | 281.537 (100.930, 552.011) | 409.659 (155.943, 790.491) | 151.411 (71.424, 286.026) | 66.622 (26.065, 134.809) |
| AAPC on ASDR, 1990-2019 | 4.183 (3.725, 4.643) | 5.087 (4.827, 5.348) | 4.604 (4.388, 4.821) | 2.505 (2.221, 2.790) | 5.221 (4.960, 5.482) |
| Togo |  |  |  |  |  |
| ASDR in 2019 (per 100,000) | 45.352 (18.614, 85.814) | 409.272 (180.384, 737.609) | 456.895 (206.154, 816.445) | 178.940 (94.037, 297.174) | 101.757 (43.752, 188.028) |
| AAPC on ASDR, 1990-2019 | 2.500 (2.179, 2.822) | 2.218 (1.865, 2.572) | 1.920 (1.573, 2.268) | 1.019 (0.673, 1.365) | 3.289 (2.987, 3.592) |
| Tokelau |  |  |  |  |  |
| ASDR in 2019 (per 100,000) | 31.376 (0.131, 97.679) | 268.987 (1.149, 823.066) | 188.663 (1.015, 571.159) | 51.883 (0.275, 155.392) | 236.691 (0.667, 678.094) |
| AAPC on ASDR, 1990-2019 | -0.325 (-0.805, 0.158)* | -0.799 (-1.156, -0.442) | -1.829 (-2.258, -1.398) | -2.579 (-3.127, -2.028) | 0.682 (0.119, 1.248) |
| Tonga |  |  |  |  |  |
| ASDR in 2019 (per 100,000) | 74.765 (22.096, 175.555) | 365.727 (111.020, 834.180) | 239.911 (75.243, 534.977) | 94.589 (29.353, 223.384) | 333.725 (88.037, 776.686) |
| AAPC on ASDR, 1990-2019 | 1.541 (1.183, 1.901) | 1.550 (1.127, 1.974) | 1.155 (0.697, 1.616) | -0.382 (-0.798, 0.035)* | 3.471 (3.087, 3.856) |
| Trinidad and Tobago |  |  |  |  |  |
| ASDR in 2019 (per 100,000) | 67.044 (22.913, 132.837) | 717.621 (242.632, 1339.239) | 428.090 (139.565, 834.094) | 60.246 (17.083, 125.032) | 957.998 (397.622, 1540.087) |
| AAPC on ASDR, 1990-2019 | -0.333 (-0.802, 0.139)* | -2.025 (-2.462, -1.587) | -2.045 (-2.646, -1.440) | -0.783 (-1.176, -0.388) | -0.292 (-0.560, -0.024) |
| Tunisia |  |  |  |  |  |
| ASDR in 2019 (per 100,000) | 168.748 (95.129, 276.953) | 1259.436 (737.584, 1929.727) | 609.363 (375.202, 914.075) | 161.185 (96.524, 244.863) | 358.094 (225.220, 521.186) |
| AAPC on ASDR, 1990-2019 | 0.642 (0.456, 0.829) | -0.216 (-0.382, -0.049) | -0.208 (-0.372, -0.043) | 0.382 (0.207, 0.558) | 2.710 (2.631, 2.789) |
| Turkey |  |  |  |  |  |
| ASDR in 2019 (per 100,000) | 261.193 (169.652, 379.396) | 724.053 (461.707, 1046.514) | 427.734 (290.443, 600.142) | 269.821 (174.062, 379.361) | 311.107 (202.065, 441.686) |
| AAPC on ASDR, 1990-2019 | -0.292 (-0.775, 0.194)* | -2.473 (-2.865, -2.080) | -0.312 (-0.647, 0.025)* | -0.655 (-1.047, -0.261) | -0.007 (-0.190, 0.176)* |
| Turkmenistan |  |  |  |  |  |
| ASDR in 2019 (per 100,000) | 80.714 (43.721, 130.897) | 2168.659 (1162.532, 3364.299) | 1438.855 (805.630, 2269.360) | 89.045 (44.817, 169.605) | 280.362 (164.293, 418.830) |
| AAPC on ASDR, 1990-2019 | -1.621 (-2.562, -0.670) | -0.129 (-1.028, 0.779)* | 0.786 (0.039, 1.539) | -3.352 (-3.986, -2.714) | 2.345 (1.724, 2.970) |
| Tuvalu |  |  |  |  |  |
| ASDR in 2019 (per 100,000) | 35.273 (10.884, 85.279) | 393.451 (127.896, 939.463) | 294.293 (106.297, 648.772) | 82.924 (30.260, 177.140) | 206.191 (50.502, 544.454) |
| AAPC on ASDR, 1990-2019 | 1.894 (1.806, 1.981) | 2.070 (1.994, 2.145) | 0.974 (0.899, 1.048) | -1.346 (-1.518, -1.173) | 4.413 (4.200, 4.626) |
| Uganda |  |  |  |  |  |
| ASDR in 2019 (per 100,000) | 21.216 (9.199, 39.295) | 186.740 (76.420, 364.077) | 294.614 (130.359, 545.666) | 134.274 (68.655, 229.578) | 98.320 (41.472, 189.087) |
| AAPC on ASDR, 1990-2019 | 4.327 (4.114, 4.540) | 3.973 (3.847, 4.100) | 3.159 (2.975, 3.344) | 2.072 (1.821, 2.323) | 4.299 (4.157, 4.441) |
| Ukraine |  |  |  |  |  |
| ASDR in 2019 (per 100,000) | 114.509 (61.679, 182.998) | 1464.382 (783.601, 2351.460) | 578.204 (317.782, 918.542) | 67.197 (35.654, 115.069) | 78.693 (40.562, 126.897) |
| AAPC on ASDR, 1990-2019 | -3.176 (-4.048, -2.297) | -0.054 (-1.553, 1.468)* | -1.814 (-3.554, -0.043) | -4.965 (-5.589, -4.336) | -0.150 (-0.445, 0.147)* |
| United Arab Emirates |  |  |  |  |  |
| ASDR in 2019 (per 100,000) | 181.426 (103.316, 294.185) | 1619.991 (988.301, 2437.645) | 1051.822 (674.918, 1578.228) | 420.408 (257.847, 647.695) | 779.120 (513.450, 1115.810) |
| AAPC on ASDR, 1990-2019 | -0.200 (-0.380, -0.020) | -1.597 (-2.120, -1.071) | -1.958 (-2.516, -1.396) | -0.444 (-0.875, -0.010) | -0.036 (-0.317, 0.246)* |
| United Kingdom |  |  |  |  |  |
| ASDR in 2019 (per 100,000) | 73.567 (43.850, 111.625) | 141.265 (75.465, 233.698) | 69.528 (39.912, 112.055) | 68.647 (39.143, 105.981) | 92.810 (46.338, 156.162) |
| AAPC on ASDR, 1990-2019 | -3.788 (-4.020, -3.556) | -6.056 (-6.281, -5.830) | -5.047 (-5.268, -4.825) | -2.953 (-3.104, -2.801) | -0.182 (-0.306, -0.057) |
| United Republic of Tanzania |  |  |  |  |  |
| ASDR in 2019 (per 100,000) | 21.599 (8.571, 44.568) | 163.958 (70.240, 318.528) | 246.703 (113.356, 456.377) | 76.202 (42.628, 125.133) | 67.144 (29.083, 126.561) |
| AAPC on ASDR, 1990-2019 | 3.410 (3.217, 3.604) | 3.439 (3.117, 3.761) | 2.477 (2.017, 2.938) | 2.042 (1.861, 2.222) | 3.772 (3.519, 4.025) |
| United States of America |  |  |  |  |  |
| ASDR in 2019 (per 100,000) | 55.431 (26.696, 92.962) | 137.843 (60.764, 252.506) | 61.636 (29.318, 109.999) | 56.847 (26.548, 95.949) | 82.848 (36.153, 149.454) |
| AAPC on ASDR, 1990-2019 | -4.287 (-4.511, -4.062) | -4.948 (-5.145, -4.751) | -3.758 (-3.982, -3.533) | -2.782 (-3.037, -2.526) | -1.576 (-1.735, -1.416) |
| United States Virgin Islands |  |  |  |  |  |
| ASDR in 2019 (per 100,000) | 53.855 (23.119, 96.405) | 344.781 (141.808, 640.669) | 131.056 (55.492, 238.208) | 22.232 (9.425, 39.283) | 221.842 (93.730, 384.218) |
| AAPC on ASDR, 1990-2019 | 0.548 (0.342, 0.754) | -0.846 (-1.158, -0.533) | -1.122 (-1.419, -0.823) | -0.384 (-0.757, -0.009) | 0.912 (0.756, 1.068) |
| Uruguay |  |  |  |  |  |
| ASDR in 2019 (per 100,000) | 86.858 (39.029, 147.046) | 144.753 (62.789, 261.266) | 136.509 (62.228, 241.341) | 65.583 (28.907, 113.183) | 76.128 (33.033, 132.480) |
| AAPC on ASDR, 1990-2019 | -1.435 (-1.747, -1.121) | -3.003 (-3.323, -2.683) | -2.651 (-2.898, -2.403) | -0.025 (-0.412, 0.363)* | 1.198 (0.938, 1.459) |
| Uzbekistan |  |  |  |  |  |
| ASDR in 2019 (per 100,000) | 112.558 (65.823, 172.473) | 3841.578 (2246.273, 5578.397) | 1577.904 (928.721, 2347.422) | 154.968 (83.511, 271.178) | 443.256 (276.309, 640.289) |
| AAPC on ASDR, 1990-2019 | -0.323 (-0.655, 0.011)* | 3.559 (3.205, 3.914) | 1.836 (1.385, 2.288) | -1.242 (-1.630, -0.852) | 5.922 (5.575, 6.270) |
| Vanuatu |  |  |  |  |  |
| ASDR in 2019 (per 100,000) | 47.709 (11.563, 120.941) | 591.665 (161.601, 1423.720) | 459.154 (120.283, 1085.859) | 173.227 (49.526, 414.873) | 142.105 (37.583, 346.877) |
| AAPC on ASDR, 1990-2019 | 2.345 (2.011, 2.680) | 2.445 (2.148, 2.742) | 1.861 (1.605, 2.118) | 0.192 (-0.042, 0.427)* | 4.079 (3.792, 4.366) |
| Venezuela (Bolivarian Republic of) |  |  |  |  |  |
| ASDR in 2019 (per 100,000) | 124.841 (69.073, 199.266) | 787.439 (432.913, 1245.103) | 400.070 (221.737, 635.893) | 122.490 (66.651, 194.150) | 458.971 (274.203, 670.551) |
| AAPC on ASDR, 1990-2019 | 0.240 (-0.496, 0.982)* | -0.727 (-1.355, -0.096) | -0.858 (-1.486, -0.227) | 1.137 (0.381, 1.898) | 1.147 (0.478, 1.820) |
| Viet Nam |  |  |  |  |  |
| ASDR in 2019 (per 100,000) | 137.395 (78.630, 214.455) | 362.916 (213.065, 559.360) | 874.143 (551.346, 1263.411) | 186.878 (93.575, 277.584) | 197.843 (110.523, 304.853) |
| AAPC on ASDR, 1990-2019 | 3.308 (3.214, 3.403) | 2.535 (2.426, 2.645) | 2.565 (2.440, 2.690) | 0.804 (0.745, 0.864) | 4.835 (4.762, 4.907) |
| Yemen |  |  |  |  |  |
| ASDR in 2019 (per 100,000) | 70.042 (28.720, 136.873) | 1733.203 (803.730, 2911.597) | 912.783 (417.095, 1566.649) | 288.661 (155.802, 475.829) | 153.596 (74.685, 256.843) |
| AAPC on ASDR, 1990-2019 | 4.718 (4.533, 4.904) | 3.863 (3.480, 4.248) | 3.924 (3.624, 4.226) | 2.969 (2.686, 3.253) | 6.587 (6.334, 6.841) |
| Zambia |  |  |  |  |  |
| ASDR in 2019 (per 100,000) | 41.039 (18.129, 76.558) | 283.212 (131.436, 507.373) | 606.584 (292.701, 1034.848) | 145.019 (82.043, 235.548) | 139.010 (63.610, 246.007) |
| AAPC on ASDR, 1990-2019 | 3.439 (3.178, 3.701) | 2.697 (2.519, 2.875) | 3.872 (3.685, 4.059) | 1.798 (1.712, 1.885) | 3.771 (3.613, 3.929) |
| Zimbabwe |  |  |  |  |  |
| ASDR in 2019 (per 100,000) | 43.191 (19.072, 77.016) | 361.540 (163.403, 646.400) | 274.355 (122.518, 501.762) | 107.142 (58.414, 177.167) | 146.947 (63.646, 267.568) |
| AAPC on ASDR, 1990-2019 | 1.034 (0.808, 1.260) | 1.829 (1.486, 2.173) | 1.324 (1.025, 1.625) | 0.228 (0.033, 0.424) | 2.729 (2.461, 2.999) |
| TBL: tracheal, bronchus, and lung; IHD: ischemic heart disease; COPD: chronic obstructive pulmonary disease; ASDR: age-standardized DALY rate; AAPC: average annual percent change.  ***NOT** statistically significant since *P* value > 0.05. | | | | | |

| **Table S4 Changes in deaths of non-communicable diseases driven by aging, population growth, and epidemiological change from 1990 to 2019** | | | | |
| --- | --- | --- | --- | --- |
| **Location** | **Overall difference** | **Aging** | **Population Growth** | **Epidemiological change** |
| Tracheal, bronchus, and lung cancer attributed to ambient particulate matter pollution among both genders | | | | |
| Global | 189244.45 | 37943.02 (20.05%) | 110891.81 (58.6%) | 40409.63 (21.35%) |
| Low SDI | 3990 | -78.4 (-1.97%) | 1928.37 (48.33%) | 2140.03 (53.63%) |
| Low-middle SDI | 21709.64 | 2165.37 (9.97%) | 8263.71 (38.06%) | 11280.56 (51.96%) |
| Middle SDI | 102960 | 17905.85 (17.39%) | 38644.19 (37.53%) | 46409.97 (45.08%) |
| High-middle SDI | 60060.13 | 15655.52 (26.07%) | 33431.35 (55.66%) | 10973.26 (18.27%) |
| High SDI | 477.96 | 10881.94 (2276.77%) | 14752.76 (3086.64%) | -25156.74 (-5263.4%) |
| Central Europe, Eastern Europe, and Central Asia | -3705.09 | 2827.83 (-76.32%) | 3074.68 (-82.99%) | -9607.6 (259.31%) |
| High-income | -4266.72 | 12472.12 (-292.31%) | 13983.8 (-327.74%) | -30722.65 (720.05%) |
| Latin America and Caribbean | 5122.23 | 1752.58 (34.22%) | 4089.73 (79.84%) | -720.08 (-14.06%) |
| North Africa and Middle East | 10231.43 | 342.94 (3.35%) | 7754.47 (75.79%) | 2134.03 (20.86%) |
| South Asia | 20541.91 | 2444.66 (11.9%) | 8006.71 (38.98%) | 10090.54 (49.12%) |
| Southeast Asia, East Asia, and Oceania | 157740.31 | 32466.13 (20.58%) | 52255.79 (33.13%) | 73018.39 (46.29%) |
| Sub-Saharan Africa | 3580.39 | -259.28 (-7.24%) | 2357.96 (65.86%) | 1481.71 (41.38%) |
| Tracheal, bronchus, and lung cancer attributed to ambient particulate matter pollution among males | | | | |
| Global | 125245.64 | 32897.99 (26.27%) | 80391 (64.19%) | 11956.65 (9.55%) |
| Low SDI | 3127.91 | -122.02 (-3.9%) | 1572.06 (50.26%) | 1677.87 (53.64%) |
| Low-middle SDI | 15730.25 | 1422.6 (9.04%) | 6065.8 (38.56%) | 8241.85 (52.39%) |
| Middle SDI | 71779.57 | 13636.46 (19%) | 26990.26 (37.6%) | 31152.84 (43.4%) |
| High-middle SDI | 37554.47 | 14553.38 (38.75%) | 25751.01 (68.57%) | -2749.92 (-7.32%) |
| High SDI | -2974.81 | 9908.18 (-333.07%) | 11135.14 (-374.31%) | -24018.13 (807.38%) |
| Central Europe, Eastern Europe, and Central Asia | -4669.35 | 3345.93 (-71.66%) | 2664.96 (-57.07%) | -10680.24 (228.73%) |
| High-income | -7012.01 | 11614.18 (-165.63%) | 10555.2 (-150.53%) | -29181.39 (416.16%) |
| Latin America and Caribbean | 2710.69 | 1073.84 (39.62%) | 2650.84 (97.79%) | -1014 (-37.41%) |
| North Africa and Middle East | 7650.34 | 176.21 (2.3%) | 6484.74 (84.76%) | 989.4 (12.93%) |
| South Asia | 14957.49 | 1676.08 (11.21%) | 5973.45 (39.94%) | 7307.96 (48.86%) |
| Southeast Asia, East Asia, and Oceania | 109080.53 | 24442.86 (22.41%) | 36233.43 (33.22%) | 48404.24 (44.37%) |
| Sub-Saharan Africa | 2527.96 | -246.88 (-9.77%) | 1731.61 (68.5%) | 1043.22 (41.27%) |
| Tracheal, bronchus, and lung cancer attributed to ambient particulate matter pollution among females | | | | |
| Global | 63998.81 | 9237.03 (14.43%) | 30041.87 (46.94%) | 24719.91 (38.63%) |
| Low SDI | 862.09 | 1.21 (0.14%) | 344.67 (39.98%) | 516.21 (59.88%) |
| Low-middle SDI | 5979.4 | 607.51 (10.16%) | 2043.86 (34.18%) | 3328.03 (55.66%) |
| Middle SDI | 31180.43 | 4817.63 (15.45%) | 11031.01 (35.38%) | 15331.8 (49.17%) |
| High-middle SDI | 22505.65 | 3563.18 (15.83%) | 8118.95 (36.08%) | 10823.53 (48.09%) |
| High SDI | 3452.77 | 2891.42 (83.74%) | 4208.68 (121.89%) | -3647.33 (-105.63%) |
| Central Europe, Eastern Europe, and Central Asia | 964.25 | 518.18 (53.74%) | 574.44 (59.57%) | -128.37 (-13.31%) |
| High-income | 2745.29 | 3147.3 (114.64%) | 3875.34 (141.16%) | -4277.35 (-155.81%) |
| Latin America and Caribbean | 2411.55 | 632.76 (26.24%) | 1398.75 (58%) | 380.04 (15.76%) |
| North Africa and Middle East | 2581.09 | 99.04 (3.84%) | 1385.71 (53.69%) | 1096.35 (42.48%) |
| South Asia | 5584.42 | 615 (11.01%) | 1840.92 (32.97%) | 3128.5 (56.02%) |
| Southeast Asia, East Asia, and Oceania | 48659.78 | 9083.95 (18.67%) | 15361.4 (31.57%) | 24214.43 (49.76%) |
| Sub-Saharan Africa | 1052.43 | -47.96 (-4.56%) | 591.44 (56.2%) | 508.95 (48.36%) |
| Ischemic heart disease attributed to ambient particulate matter pollution among both genders | | | | |
| Global | 724466.86 | 198146.14 (27.35%) | 522285.15 (72.09%) | 4035.57 (0.56%) |
| Low SDI | 50500.42 | -385.89 (-0.76%) | 25215.43 (49.93%) | 25670.88 (50.83%) |
| Low-middle SDI | 225755.34 | 22576.1 (10%) | 86453.72 (38.3%) | 116725.52 (51.7%) |
| Middle SDI | 410278.59 | 91124.93 (22.21%) | 196263.29 (47.84%) | 122890.37 (29.95%) |
| High-middle SDI | 114759.39 | 80067.46 (69.77%) | 134302.16 (117.03%) | -99610.22 (-86.8%) |
| High SDI | -77068.72 | 43699.98 (-56.7%) | 48793.17 (-63.31%) | -169561.87 (220.01%) |
| Central Europe, Eastern Europe, and Central Asia | -17951.15 | 34160.69 (-190.3%) | 22569.87 (-125.73%) | -74681.71 (416.03%) |
| High-income | -98126.83 | 49815.29 (-50.77%) | 43008.05 (-43.83%) | -190950.16 (194.6%) |
| Latin America and Caribbean | 24633.8 | 11890.58 (48.27%) | 26745.98 (108.57%) | -14002.75 (-56.84%) |
| North Africa and Middle East | 103655.33 | 6912.05 (6.67%) | 107721.39 (103.92%) | -10978.12 (-10.59%) |
| South Asia | 318036.46 | 38509.22 (12.11%) | 134397.35 (42.26%) | 145129.89 (45.63%) |
| Southeast Asia, East Asia, and Oceania | 367874.21 | 84296.04 (22.91%) | 125525.6 (34.12%) | 158052.56 (42.96%) |
| Sub-Saharan Africa | 26345.04 | -1454.36 (-5.52%) | 16343.89 (62.04%) | 11455.5 (43.48%) |
| Ischemic heart disease attributed to ambient particulate matter pollution among males | | | | |
| Global | 450395.45 | 124325.68 (27.6%) | 308937.2 (68.59%) | 17132.57 (3.8%) |
| Low SDI | 32843.2 | -814.64 (-2.48%) | 16779.46 (51.09%) | 16878.38 (51.39%) |
| Low-middle SDI | 142821.12 | 11881.44 (8.32%) | 54494.49 (38.16%) | 76445.19 (53.53%) |
| Middle SDI | 246104.97 | 55110.27 (22.39%) | 116663.21 (47.4%) | 74331.48 (30.2%) |
| High-middle SDI | 67838.56 | 46775.57 (68.95%) | 77501.22 (114.24%) | -56438.23 (-83.19%) |
| High SDI | -39364.33 | 27784.41 (-70.58%) | 30316.22 (-77.01%) | -97464.96 (247.6%) |
| Central Europe, Eastern Europe, and Central Asia | -8200.14 | 19767.18 (-241.06%) | 12807.25 (-156.18%) | -40774.56 (497.24%) |
| High-income | -53725.15 | 30848.51 (-57.42%) | 25784.59 (-47.99%) | -110358.26 (205.41%) |
| Latin America and Caribbean | 14402.96 | 6183.29 (42.93%) | 15938.48 (110.66%) | -7718.82 (-53.59%) |
| North Africa and Middle East | 61649.57 | 3587.75 (5.82%) | 67548.35 (109.57%) | -9486.53 (-15.39%) |
| South Asia | 201552.89 | 20541.51 (10.19%) | 86001.84 (42.67%) | 95009.55 (47.14%) |
| Southeast Asia, East Asia, and Oceania | 219661.38 | 50036.93 (22.78%) | 74786.35 (34.05%) | 94838.09 (43.17%) |
| Sub-Saharan Africa | 15053.94 | -1061.51 (-7.05%) | 9589.58 (63.7%) | 6525.87 (43.35%) |
| Ischemic heart disease attributed to ambient particulate matter pollution among females | | | | |
| Global | 274071.42 | 79966.2 (29.18%) | 212397.79 (77.5%) | -18292.57 (-6.67%) |
| Low SDI | 17657.23 | 213.58 (1.21%) | 8342.4 (47.25%) | 9101.25 (51.54%) |
| Low-middle SDI | 82934.22 | 9981.09 (12.03%) | 31008.67 (37.39%) | 41944.47 (50.58%) |
| Middle SDI | 164173.63 | 36619.66 (22.31%) | 77984.7 (47.5%) | 49569.28 (30.19%) |
| High-middle SDI | 46920.84 | 36817.23 (78.47%) | 57352.57 (122.23%) | -47248.96 (-100.7%) |
| High SDI | -37704.39 | 19087.9 (-50.63%) | 19372.56 (-51.38%) | -76164.84 (202.01%) |
| Central Europe, Eastern Europe, and Central Asia | -9751.02 | 17340.45 (-177.83%) | 10019.87 (-102.76%) | -37111.34 (380.59%) |
| High-income | -44401.67 | 22309.21 (-50.24%) | 17825.53 (-40.15%) | -84536.42 (190.39%) |
| Latin America and Caribbean | 10230.85 | 5538.64 (54.14%) | 10638.27 (103.98%) | -5946.06 (-58.12%) |
| North Africa and Middle East | 42005.76 | 3105.58 (7.39%) | 40703.14 (96.9%) | -1802.96 (-4.29%) |
| South Asia | 116483.58 | 16698.49 (14.34%) | 46439.72 (39.87%) | 53345.37 (45.8%) |
| Southeast Asia, East Asia, and Oceania | 148212.83 | 35341.84 (23.85%) | 49931.84 (33.69%) | 62939.15 (42.47%) |
| Sub-Saharan Africa | 11291.1 | -520.12 (-4.61%) | 6648.1 (58.88%) | 5163.11 (45.73%) |
| Stroke attributed to ambient particulate matter pollution among both genders | | | | |
| Global | 635318.6 | 169793.06 (26.73%) | 441880.31 (69.55%) | 23645.23 (3.72%) |
| Low SDI | 36094.71 | -315.13 (-0.87%) | 20309.56 (56.27%) | 16100.28 (44.61%) |
| Low-middle SDI | 172871.26 | 21571.2 (12.48%) | 73742.26 (42.66%) | 77557.8 (44.86%) |
| Middle SDI | 358838.48 | 93179.74 (25.97%) | 191456.36 (53.35%) | 74202.38 (20.68%) |
| High-middle SDI | 98719.77 | 70069.34 (70.98%) | 118283.48 (119.82%) | -89633.04 (-90.8%) |
| High SDI | -31404.38 | 24427.73 (-77.78%) | 26578.17 (-84.63%) | -82410.28 (262.42%) |
| Central Europe, Eastern Europe, and Central Asia | -29142.65 | 21086.18 (-72.36%) | 13881.8 (-47.63%) | -64110.62 (219.99%) |
| High-income | -45392.4 | 29400.12 (-64.77%) | 24037.72 (-52.96%) | -98830.24 (217.72%) |
| Latin America and Caribbean | 10577.77 | 7535.45 (71.24%) | 18291.1 (172.92%) | -15248.78 (-144.16%) |
| North Africa and Middle East | 46318.2 | 3069.39 (6.63%) | 43130.86 (93.12%) | 117.95 (0.25%) |
| South Asia | 169013.32 | 26723.11 (15.81%) | 78596.9 (46.5%) | 63693.31 (37.69%) |
| Southeast Asia, East Asia, and Oceania | 453911.1 | 143746.06 (31.67%) | 215647.55 (47.51%) | 94517.49 (20.82%) |
| Sub-Saharan Africa | 30033.25 | -1764.33 (-5.87%) | 19989.08 (66.56%) | 11808.5 (39.32%) |
| Stroke attributed to ambient particulate matter pollution among males | | | | |
| Global | 387652.17 | 104276.89 (26.9%) | 240439.45 (62.02%) | 42935.84 (11.08%) |
| Low SDI | 20207.71 | -628.91 (-3.11%) | 11950.67 (59.14%) | 8885.95 (43.97%) |
| Low-middle SDI | 97808.24 | 10588.06 (10.83%) | 41810.39 (42.75%) | 45409.79 (46.43%) |
| Middle SDI | 211016.83 | 55226.82 (26.17%) | 107049.7 (50.73%) | 48740.31 (23.1%) |
| High-middle SDI | 69195.87 | 40925.39 (59.14%) | 63042.67 (91.11%) | -34772.19 (-50.25%) |
| High SDI | -10688.77 | 13578.4 (-127.03%) | 13871.54 (-129.78%) | -38138.71 (356.81%) |
| Central Europe, Eastern Europe, and Central Asia | -7723.22 | 10736.57 (-139.02%) | 6535.09 (-84.62%) | -24994.88 (323.63%) |
| High-income | -19039.94 | 15463.02 (-81.21%) | 11695.72 (-61.43%) | -46198.68 (242.64%) |
| Latin America and Caribbean | 5062.61 | 3597.88 (71.07%) | 9585.04 (189.33%) | -8120.31 (-160.4%) |
| North Africa and Middle East | 23677.64 | 1415 (5.98%) | 23172.69 (97.87%) | -910.05 (-3.84%) |
| South Asia | 92984.87 | 13269.6 (14.27%) | 44315.95 (47.66%) | 35399.33 (38.07%) |
| Southeast Asia, East Asia, and Oceania | 276847.91 | 87722.1 (31.69%) | 125649.56 (45.39%) | 63476.24 (22.93%) |
| Sub-Saharan Africa | 15842.29 | -1213.14 (-7.66%) | 10664.52 (67.32%) | 6390.91 (40.34%) |
| Stroke attributed to ambient particulate matter pollution among females | | | | |
| Global | 247666.43 | 70593.72 (28.5%) | 200831.36 (81.09%) | -23758.65 (-9.59%) |
| Low SDI | 15887 | 197.81 (1.25%) | 8307.76 (52.29%) | 7381.43 (46.46%) |
| Low-middle SDI | 75063.02 | 10502.37 (13.99%) | 31432 (41.87%) | 33128.65 (44.13%) |
| Middle SDI | 147821.65 | 38876.42 (26.3%) | 83330.84 (56.37%) | 25614.38 (17.33%) |
| High-middle SDI | 29523.9 | 32275.51 (109.32%) | 55377.44 (187.57%) | -58129.05 (-196.89%) |
| High SDI | -20715.61 | 11824.95 (-57.08%) | 12736.03 (-61.48%) | -45276.59 (218.56%) |
| Central Europe, Eastern Europe, and Central Asia | -21419.43 | 11392.02 (-53.19%) | 7315.33 (-34.15%) | -40126.78 (187.34%) |
| High-income | -26352.46 | 14798.96 (-56.16%) | 12349.13 (-46.86%) | -53500.56 (203.02%) |
| Latin America and Caribbean | 5515.16 | 3870.41 (70.18%) | 8650.61 (156.85%) | -7005.86 (-127.03%) |
| North Africa and Middle East | 22640.56 | 1613 (7.12%) | 19985.62 (88.27%) | 1041.95 (4.6%) |
| South Asia | 76028.45 | 12951.75 (17.04%) | 33629.89 (44.23%) | 29446.8 (38.73%) |
| Southeast Asia, East Asia, and Oceania | 177063.2 | 58429.79 (33%) | 88801.29 (50.15%) | 29832.12 (16.85%) |
| Sub-Saharan Africa | 14190.96 | -660.66 (-4.66%) | 9255.22 (65.22%) | 5596.4 (39.44%) |
| Chronic obstructive pulmonary disease attributed to ambient particulate matter pollution among both genders | | | | |
| Global | 343337.09 | 135132.35 (39.36%) | 285908.4 (83.27%) | -77703.67 (-22.63%) |
| Low SDI | 39731.48 | 802.02 (2.02%) | 23391.39 (58.87%) | 15538.07 (39.11%) |
| Low-middle SDI | 177804.27 | 36161.72 (20.34%) | 87441.26 (49.18%) | 54201.29 (30.48%) |
| Middle SDI | 117670.24 | 77186.33 (65.6%) | 127532.28 (108.38%) | -87048.37 (-73.98%) |
| High-middle SDI | 7991.26 | 41792.05 (522.97%) | 55038.25 (688.73%) | -88839.04 (-1111.7%) |
| High SDI | 80.03 | 10679.24 (13344.26%) | 9868.83 (12331.61%) | -20468.04 (-25575.87%) |
| Central Europe, Eastern Europe, and Central Asia | -9059.9 | 4183.72 (-46.18%) | 2428.35 (-26.8%) | -15671.97 (172.98%) |
| High-income | -1088.09 | 12904.74 (-1186%) | 9271.77 (-852.11%) | -23264.6 (2138.11%) |
| Latin America and Caribbean | 8880.34 | 4994.11 (56.24%) | 8181.26 (92.13%) | -4295.03 (-48.37%) |
| North Africa and Middle East | 12880.61 | 1147.34 (8.91%) | 11870.64 (92.16%) | -137.37 (-1.07%) |
| South Asia | 235058.44 | 59292.21 (25.22%) | 116959.71 (49.76%) | 58806.52 (25.02%) |
| Southeast Asia, East Asia, and Oceania | 87737.24 | 129076.89 (147.12%) | 153325.17 (174.75%) | -194664.82 (-221.87%) |
| Sub-Saharan Africa | 8928.47 | -708.82 (-7.94%) | 7169.27 (80.3%) | 2468.02 (27.64%) |
| Chronic obstructive pulmonary disease attributed to ambient particulate matter pollution among males | | | | |
| Global | 200719.03 | 96272.24 (47.96%) | 169359.09 (84.38%) | -64912.3 (-32.34%) |
| Low SDI | 22778.33 | -118.18 (-0.52%) | 14598.88 (64.09%) | 8297.63 (36.43%) |
| Low-middle SDI | 102238.48 | 19350.56 (18.93%) | 52779.81 (51.62%) | 30108.1 (29.45%) |
| Middle SDI | 71199.36 | 47293.01 (66.42%) | 71744.33 (100.77%) | -47837.99 (-67.19%) |
| High-middle SDI | 5449.82 | 28229.25 (517.98%) | 32749.9 (600.94%) | -55529.33 (-1018.92%) |
| High SDI | -983.59 | 8298.14 (-843.66%) | 6468.26 (-657.62%) | -15749.99 (1601.27%) |
| Central Europe, Eastern Europe, and Central Asia | -5963.33 | 3340.69 (-56.02%) | 1667.55 (-27.96%) | -10971.56 (183.98%) |
| High-income | -2525.62 | 9861.1 (-390.44%) | 5968.9 (-236.33%) | -18355.63 (726.78%) |
| Latin America and Caribbean | 4325.74 | 2532.92 (58.55%) | 4582.21 (105.93%) | -2789.38 (-64.48%) |
| North Africa and Middle East | 8349.65 | 734.17 (8.79%) | 7792.43 (93.33%) | -176.95 (-2.12%) |
| South Asia | 132731.18 | 31743.59 (23.92%) | 70235.52 (52.92%) | 30752.07 (23.17%) |
| Southeast Asia, East Asia, and Oceania | 58197.37 | 76204.62 (130.94%) | 84741.91 (145.61%) | -102749.16 (-176.55%) |
| Sub-Saharan Africa | 5604.03 | -527.71 (-9.42%) | 4568.48 (81.52%) | 1563.26 (27.9%) |
| Chronic obstructive pulmonary disease attributed to ambient particulate matter pollution among females | | | | |
| Global | 142618.07 | 49171.45 (34.48%) | 116437.22 (81.64%) | -22990.6 (-16.12%) |
| Low SDI | 16953.15 | 701.92 (4.14%) | 8707.03 (51.36%) | 7544.2 (44.5%) |
| Low-middle SDI | 75565.79 | 15734.28 (20.82%) | 33702.78 (44.6%) | 26128.73 (34.58%) |
| Middle SDI | 46470.88 | 31364.01 (67.49%) | 55035.43 (118.43%) | -39928.55 (-85.92%) |
| High-middle SDI | 2541.44 | 17453.04 (686.74%) | 22795.09 (896.93%) | -37706.69 (-1483.67%) |
| High SDI | 1063.62 | 3952.37 (371.6%) | 3689.56 (346.89%) | -6578.31 (-618.48%) |
| Central Europe, Eastern Europe, and Central Asia | -3096.57 | 1640.33 (-52.97%) | 842.12 (-27.2%) | -5579.02 (180.17%) |
| High-income | 1437.53 | 4803.28 (334.13%) | 3535.37 (245.93%) | -6901.13 (-480.07%) |
| Latin America and Caribbean | 4554.6 | 2363.71 (51.9%) | 3550 (77.94%) | -1359.12 (-29.84%) |
| North Africa and Middle East | 4530.96 | 424.7 (9.37%) | 4152.34 (91.64%) | -46.08 (-1.02%) |
| South Asia | 102327.25 | 25857.26 (25.27%) | 45158.42 (44.13%) | 31311.58 (30.6%) |
| Southeast Asia, East Asia, and Oceania | 29539.86 | 55587.43 (188.18%) | 68116.93 (230.59%) | -94164.49 (-318.77%) |
| Sub-Saharan Africa | 3324.43 | -211.1 (-6.35%) | 2529.16 (76.08%) | 1006.37 (30.27%) |
| Diabetes mellitus attributed to ambient particulate matter pollution among both genders | | | | |
| Global | 140967.64 | 26267.82 (18.63%) | 62978.85 (44.68%) | 51720.97 (36.69%) |
| Low SDI | 7933.28 | -7.93 (-0.1%) | 3623.81 (45.68%) | 4317.4 (54.42%) |
| Low-middle SDI | 36597.87 | 4207.84 (11.5%) | 12071.8 (32.98%) | 20318.24 (55.52%) |
| Middle SDI | 70214.46 | 13710.55 (19.53%) | 26989.82 (38.44%) | 29514.09 (42.03%) |
| High-middle SDI | 24272.38 | 7661.44 (31.56%) | 12219.08 (50.34%) | 4391.86 (18.09%) |
| High SDI | 1861.15 | 6035.55 (324.29%) | 6085.62 (326.98%) | -10260.03 (-551.27%) |
| Central Europe, Eastern Europe, and Central Asia | 4774.25 | 1252.83 (26.24%) | 927.92 (19.44%) | 2593.51 (54.32%) |
| High-income | -136.51 | 7950.79 (-5824.36%) | 6245.75 (-4575.33%) | -14333.04 (10499.7%) |
| Latin America and Caribbean | 16885.61 | 4570.29 (27.07%) | 9798.98 (58.03%) | 2516.34 (14.9%) |
| North Africa and Middle East | 14395.95 | 704.86 (4.9%) | 9947.91 (69.1%) | 3743.17 (26%) |
| South Asia | 46095.75 | 6699.28 (14.53%) | 15515.86 (33.66%) | 23880.62 (51.81%) |
| Southeast Asia, East Asia, and Oceania | 48044.57 | 10608.4 (22.08%) | 15868.91 (33.03%) | 21567.26 (44.89%) |
| Sub-Saharan Africa | 10908.02 | -571.94 (-5.24%) | 5681.93 (52.09%) | 5798.03 (53.15%) |
| Diabetes mellitus attributed to ambient particulate matter pollution among males | | | | |
| Global | 74041.84 | 14384.17 (19.43%) | 30258.78 (40.87%) | 29398.88 (39.71%) |
| Low SDI | 4444.57 | -91.81 (-2.07%) | 2155.61 (48.5%) | 2380.77 (53.57%) |
| Low-middle SDI | 19726.22 | 2022.53 (10.25%) | 6445.28 (32.67%) | 11258.42 (57.07%) |
| Middle SDI | 34962.59 | 6900.64 (19.74%) | 12827.91 (36.69%) | 15234.04 (43.57%) |
| High-middle SDI | 12294.74 | 3906.52 (31.77%) | 5554.68 (45.18%) | 2833.54 (23.05%) |
| High SDI | 2567.11 | 3113.58 (121.29%) | 2949.61 (114.9%) | -3496.09 (-136.19%) |
| Central Europe, Eastern Europe, and Central Asia | 2234.27 | 651.99 (29.18%) | 406.59 (18.2%) | 1175.69 (52.62%) |
| High-income | 1672.55 | 3895.19 (232.89%) | 2842.27 (169.94%) | -5064.9 (-302.83%) |
| Latin America and Caribbean | 8571.51 | 1904.24 (22.22%) | 4548.83 (53.07%) | 2118.44 (24.71%) |
| North Africa and Middle East | 7059.69 | 280.98 (3.98%) | 4760.69 (67.43%) | 2018.02 (28.59%) |
| South Asia | 24074.43 | 3202.5 (13.3%) | 8168.61 (33.93%) | 12703.32 (52.77%) |
| Southeast Asia, East Asia, and Oceania | 24648.66 | 5471.17 (22.2%) | 7796.92 (31.63%) | 11380.57 (46.17%) |
| Sub-Saharan Africa | 5780.73 | -376.84 (-6.52%) | 3002.62 (51.94%) | 3154.95 (54.58%) |
| Diabetes mellitus attributed to ambient particulate matter pollution among females | | | | |
| Global | 66925.8 | 12099.43 (18.08%) | 32696.74 (48.86%) | 22129.63 (33.07%) |
| Low SDI | 3488.71 | 59.23 (1.7%) | 1459.64 (41.84%) | 1969.83 (56.46%) |
| Low-middle SDI | 16871.65 | 2116.87 (12.55%) | 5583.83 (33.1%) | 9170.95 (54.36%) |
| Middle SDI | 35251.88 | 6764.31 (19.19%) | 14181.38 (40.23%) | 14306.19 (40.58%) |
| High-middle SDI | 11977.64 | 3859.59 (32.22%) | 6630.5 (55.36%) | 1487.55 (12.42%) |
| High SDI | -705.96 | 3041.35 (-430.81%) | 3094.48 (-438.34%) | -6841.79 (969.15%) |
| Central Europe, Eastern Europe, and Central Asia | 2539.98 | 633.43 (24.94%) | 514.14 (20.24%) | 1392.42 (54.82%) |
| High-income | -1809.06 | 4132.51 (-228.43%) | 3365.28 (-186.02%) | -9306.85 (514.46%) |
| Latin America and Caribbean | 8314.09 | 2668.99 (32.1%) | 5261.25 (63.28%) | 383.86 (4.62%) |
| North Africa and Middle East | 7336.25 | 415.05 (5.66%) | 5163.65 (70.39%) | 1757.56 (23.96%) |
| South Asia | 22021.32 | 3415.76 (15.51%) | 7285.01 (33.08%) | 11320.56 (51.41%) |
| Southeast Asia, East Asia, and Oceania | 23395.92 | 5148.01 (22%) | 8071.2 (34.5%) | 10176.71 (43.5%) |
| Sub-Saharan Africa | 5127.29 | -223.59 (-4.36%) | 2664.52 (51.97%) | 2686.36 (52.39%) |

| **Table S5 Changes in DALYs of non-communicable diseases driven by aging, population growth, and epidemiological change from 1990 to 2019** | | | | |
| --- | --- | --- | --- | --- |
| **Location** | **Overall difference** | **Aging** | **Population Growth** | **Epidemiological change** |
| Tracheal, bronchus, and lung cancer attributed to ambient particulate matter pollution among both genders | | | | |
| Global | 4004722.87 | 723969.15 (18.08%) | 2656998.44 (66.35%) | 623755.28 (15.58%) |
| Low SDI | 104274.98 | -2828.19 (-2.71%) | 50808.5 (48.73%) | 56294.67 (53.99%) |
| Low-middle SDI | 538761.36 | 39352.79 (7.3%) | 213916.13 (39.71%) | 285492.43 (52.99%) |
| Middle SDI | 2330419.69 | 365081.71 (15.67%) | 954236.16 (40.95%) | 1011101.81 (43.39%) |
| High-middle SDI | 1171705.23 | 304048.96 (25.95%) | 811396.69 (69.25%) | 56259.59 (4.8%) |
| High SDI | -141495.91 | 185319.9 (-130.97%) | 320121.49 (-226.24%) | -646937.3 (457.21%) |
| Central Europe, Eastern Europe, and Central Asia | -164211.26 | 54641.62 (-33.28%) | 80623.33 (-49.1%) | -299476.21 (182.37%) |
| High-income | -262807.02 | 207057.04 (-78.79%) | 301778.44 (-114.83%) | -771642.51 (293.62%) |
| Latin America and Caribbean | 108186.46 | 33747.7 (31.19%) | 97604.16 (90.22%) | -23165.4 (-21.41%) |
| North Africa and Middle East | 256852.09 | 7891.01 (3.07%) | 205297.65 (79.93%) | 43663.43 (17%) |
| South Asia | 522238.1 | 43559.84 (8.34%) | 211552.86 (40.51%) | 267125.39 (51.15%) |
| Southeast Asia, East Asia, and Oceania | 3454404.23 | 643597.79 (18.63%) | 1254124.82 (36.31%) | 1556681.63 (45.06%) |
| Sub-Saharan Africa | 90060.28 | -5545.05 (-6.16%) | 61467.97 (68.25%) | 34137.36 (37.91%) |
| Tracheal, bronchus, and lung cancer attributed to ambient particulate matter pollution among males | | | | |
| Global | 2646891.64 | 620050.19 (23.43%) | 1955077.45 (73.86%) | 71763.99 (2.71%) |
| Low SDI | 81246.72 | -3601.55 (-4.43%) | 41127.69 (50.62%) | 43720.58 (53.81%) |
| Low-middle SDI | 391141.5 | 25804.01 (6.6%) | 157182.95 (40.19%) | 208154.54 (53.22%) |
| Middle SDI | 1638840.27 | 277730.3 (16.95%) | 673199.59 (41.08%) | 687910.38 (41.98%) |
| High-middle SDI | 702329.39 | 284775.68 (40.55%) | 636596.11 (90.64%) | -219042.4 (-31.19%) |
| High SDI | -167309.9 | 167976.24 (-100.4%) | 244794.77 (-146.31%) | -580080.92 (346.71%) |
| Central Europe, Eastern Europe, and Central Asia | -181111.76 | 67292.23 (-37.16%) | 71413.94 (-39.43%) | -319817.93 (176.59%) |
| High-income | -272837.8 | 194641.53 (-71.34%) | 230676.06 (-84.55%) | -698155.39 (255.89%) |
| Latin America and Caribbean | 55337.81 | 21228.45 (38.36%) | 63355.74 (114.49%) | -29246.38 (-52.85%) |
| North Africa and Middle East | 191462.67 | 3632.62 (1.9%) | 171751.89 (89.71%) | 16078.16 (8.4%) |
| South Asia | 378666.55 | 29677.89 (7.84%) | 157265.43 (41.53%) | 191723.24 (50.63%) |
| Southeast Asia, East Asia, and Oceania | 2411535.29 | 487661.16 (20.22%) | 879245.92 (36.46%) | 1044628.21 (43.32%) |
| Sub-Saharan Africa | 63838.87 | -5718.65 (-8.96%) | 45587.49 (71.41%) | 23970.02 (37.55%) |
| Tracheal, bronchus, and lung cancer attributed to ambient particulate matter pollution among females | | | | |
| Global | 1357831.24 | 165371.65 (12.18%) | 689525.25 (50.78%) | 502934.34 (37.04%) |
| Low SDI | 23028.26 | -179.2 (-0.78%) | 9365.81 (40.67%) | 13841.66 (60.11%) |
| Low-middle SDI | 147619.86 | 10556.74 (7.15%) | 52675.26 (35.68%) | 84387.87 (57.17%) |
| Middle SDI | 691579.42 | 93979.52 (13.59%) | 265189.79 (38.35%) | 332410.11 (48.07%) |
| High-middle SDI | 469375.84 | 61768.29 (13.16%) | 185988.07 (39.62%) | 221619.48 (47.22%) |
| High SDI | 25813.99 | 45471.55 (176.15%) | 87990.36 (340.86%) | -107647.91 (-417.01%) |
| Central Europe, Eastern Europe, and Central Asia | 16900.5 | 8321.56 (49.24%) | 13660.13 (80.83%) | -5081.19 (-30.07%) |
| High-income | 10030.77 | 47328.85 (471.84%) | 80350.88 (801.04%) | -117648.96 (-1172.88%) |
| Latin America and Caribbean | 52848.65 | 11695.1 (22.13%) | 33302.94 (63.02%) | 7850.61 (14.85%) |
| North Africa and Middle East | 65389.41 | 2224.76 (3.4%) | 36632.38 (56.02%) | 26532.28 (40.58%) |
| South Asia | 143571.54 | 10617.61 (7.4%) | 49230.79 (34.29%) | 83723.14 (58.31%) |
| Southeast Asia, East Asia, and Oceania | 1042868.95 | 170847.59 (16.38%) | 358643.68 (34.39%) | 513377.67 (49.23%) |
| Sub-Saharan Africa | 26221.41 | -891.54 (-3.4%) | 14931.21 (56.94%) | 12181.74 (46.46%) |
| Ischemic heart disease attributed to ambient particulate matter pollution among both genders | | | | |
| Global | 17584467.71 | 3144152.24 (17.88%) | 12556983.09 (71.41%) | 1883332.39 (10.71%) |
| Low SDI | 1411597.84 | -24418.94 (-1.73%) | 712889.11 (50.5%) | 723127.68 (51.23%) |
| Low-middle SDI | 6057043.41 | 372737.34 (6.15%) | 2398859.42 (39.6%) | 3285446.65 (54.24%) |
| Middle SDI | 9643192.9 | 1648545.49 (17.1%) | 5042244.46 (52.29%) | 2952402.95 (30.62%) |
| High-middle SDI | 2049494.11 | 1177872.25 (57.47%) | 2996169.25 (146.19%) | -2124547.39 (-103.66%) |
| High SDI | -1583265.39 | 578830.36 (-36.56%) | 984843.99 (-62.2%) | -3146939.75 (198.76%) |
| Central Europe, Eastern Europe, and Central Asia | -580580.37 | 458061.34 (-78.9%) | 484302.14 (-83.42%) | -1522943.85 (262.31%) |
| High-income | -2161429.96 | 633967.01 (-29.33%) | 830127.11 (-38.41%) | -3625524.08 (167.74%) |
| Latin America and Caribbean | 535473.34 | 201742.71 (37.68%) | 656690.48 (122.64%) | -322959.84 (-60.31%) |
| North Africa and Middle East | 2584477.68 | 131301.77 (5.08%) | 2858129.87 (110.59%) | -404953.97 (-15.67%) |
| South Asia | 8634206.46 | 621415.27 (7.2%) | 3843347.83 (44.51%) | 4169443.35 (48.29%) |
| Southeast Asia, East Asia, and Oceania | 7902555.34 | 1407901.24 (17.82%) | 3038039.52 (38.44%) | 3456614.58 (43.74%) |
| Sub-Saharan Africa | 669765.21 | -31273.67 (-4.67%) | 421180.47 (62.88%) | 279858.42 (41.78%) |
| Ischemic heart disease attributed to ambient particulate matter pollution among males | | | | |
| Global | 11583998.71 | 2108740.56 (18.2%) | 8135426.27 (70.23%) | 1339831.88 (11.57%) |
| Low SDI | 949835.13 | -27313.52 (-2.88%) | 486674.19 (51.24%) | 490474.46 (51.64%) |
| Low-middle SDI | 4019042.9 | 200238.68 (4.98%) | 1574237.16 (39.17%) | 2244567.06 (55.85%) |
| Middle SDI | 6218107.39 | 1036140.74 (16.66%) | 3188054.86 (51.27%) | 1993911.8 (32.07%) |
| High-middle SDI | 1318151.13 | 782495.03 (59.36%) | 1961349.6 (148.8%) | -1425693.51 (-108.16%) |
| High SDI | -925413.88 | 414253.46 (-44.76%) | 693997.72 (-74.99%) | -2033665.05 (219.76%) |
| Central Europe, Eastern Europe, and Central Asia | -340896.37 | 318192.93 (-93.34%) | 320780.62 (-94.1%) | -979869.92 (287.44%) |
| High-income | -1347853.2 | 449762.67 (-33.37%) | 566123.11 (-42%) | -2363738.98 (175.37%) |
| Latin America and Caribbean | 344119.21 | 114914.23 (33.39%) | 419606.82 (121.94%) | -190401.83 (-55.33%) |
| North Africa and Middle East | 1630816.01 | 70089.32 (4.3%) | 1886509.34 (115.68%) | -325782.65 (-19.98%) |
| South Asia | 5718438.04 | 330436.1 (5.78%) | 2545343.02 (44.51%) | 2842658.91 (49.71%) |
| Southeast Asia, East Asia, and Oceania | 5165347.34 | 886791.47 (17.17%) | 1953611.51 (37.82%) | 2324944.36 (45.01%) |
| Sub-Saharan Africa | 414027.67 | -24808.2 (-5.99%) | 265719.77 (64.18%) | 173116.11 (41.81%) |
| Ischemic heart disease attributed to ambient particulate matter pollution among females | | | | |
| Global | 6000469 | 1114915.48 (18.58%) | 4384813.95 (73.07%) | 500739.57 (8.35%) |
| Low SDI | 461762.72 | -1784.65 (-0.39%) | 223155.59 (48.33%) | 240391.78 (52.06%) |
| Low-middle SDI | 2038000.51 | 158065.88 (7.76%) | 793316.67 (38.93%) | 1086617.96 (53.32%) |
| Middle SDI | 3425085.51 | 614503.08 (17.94%) | 1798474.29 (52.51%) | 1012108.13 (29.55%) |
| High-middle SDI | 731342.98 | 453008.98 (61.94%) | 1057302.88 (144.57%) | -778968.87 (-106.51%) |
| High SDI | -657851.51 | 210233.96 (-31.96%) | 319483.5 (-48.56%) | -1187568.97 (180.52%) |
| Central Europe, Eastern Europe, and Central Asia | -239684 | 194833.57 (-81.29%) | 174818.89 (-72.94%) | -609336.45 (254.22%) |
| High-income | -813576.77 | 235103.03 (-28.9%) | 281303.33 (-34.58%) | -1329983.13 (163.47%) |
| Latin America and Caribbean | 191354.13 | 83760.95 (43.77%) | 231728.77 (121.1%) | -124135.58 (-64.87%) |
| North Africa and Middle East | 953661.67 | 55207.53 (5.79%) | 990153.73 (103.83%) | -91699.59 (-9.62%) |
| South Asia | 2915768.42 | 265457.14 (9.1%) | 1234072.71 (42.32%) | 1416238.57 (48.57%) |
| Southeast Asia, East Asia, and Oceania | 2737208 | 536094.92 (19.59%) | 1057055.4 (38.62%) | 1144057.68 (41.8%) |
| Sub-Saharan Africa | 255737.54 | -10160.26 (-3.97%) | 151653.75 (59.3%) | 114244.05 (44.67%) |
| Stroke attributed to ambient particulate matter pollution among both genders | | | | |
| Global | 15879537.9 | 2917973.44 (18.38%) | 11124136.43 (70.05%) | 1837428.02 (11.57%) |
| Low SDI | 1018444.62 | -21962.46 (-2.16%) | 576376.77 (56.59%) | 464030.3 (45.56%) |
| Low-middle SDI | 4521569.3 | 364088.56 (8.05%) | 2000051.23 (44.23%) | 2157429.51 (47.71%) |
| Middle SDI | 8770566.28 | 1788537.46 (20.39%) | 5021762.39 (57.26%) | 1960266.44 (22.35%) |
| High-middle SDI | 2165325.06 | 1122178.57 (51.82%) | 2795503.07 (129.1%) | -1752356.58 (-80.93%) |
| High SDI | -601942.45 | 338946.04 (-56.31%) | 606231 (-100.71%) | -1547119.49 (257.02%) |
| Central Europe, Eastern Europe, and Central Asia | -703858.67 | 305187.69 (-43.36%) | 317391.95 (-45.09%) | -1326438.32 (188.45%) |
| High-income | -1009531.73 | 382632.59 (-37.9%) | 505514.82 (-50.07%) | -1897679.14 (187.98%) |
| Latin America and Caribbean | 225077.1 | 132060.81 (58.67%) | 500108.4 (222.19%) | -407092.11 (-180.87%) |
| North Africa and Middle East | 1320078.29 | 49961.24 (3.78%) | 1252102.58 (94.85%) | 18014.46 (1.36%) |
| South Asia | 4496995.75 | 446105.28 (9.92%) | 2165833.33 (48.16%) | 1885057.13 (41.92%) |
| Southeast Asia, East Asia, and Oceania | 10708562.89 | 2711183.7 (25.32%) | 5458066.66 (50.97%) | 2539312.54 (23.71%) |
| Sub-Saharan Africa | 842214.27 | -39414.47 (-4.68%) | 565291.07 (67.12%) | 316337.67 (37.56%) |
| Stroke attributed to ambient particulate matter pollution among males | | | | |
| Global | 9682790.86 | 1835513.23 (18.96%) | 6293977.99 (65%) | 1553299.63 (16.04%) |
| Low SDI | 576018.08 | -22069.15 (-3.83%) | 340085.75 (59.04%) | 258001.48 (44.79%) |
| Low-middle SDI | 2599725.1 | 178889.69 (6.88%) | 1149082.45 (44.2%) | 1271752.96 (48.92%) |
| Middle SDI | 5218990.1 | 1064527.52 (20.4%) | 2856138.5 (54.73%) | 1298324.07 (24.88%) |
| High-middle SDI | 1511986.09 | 707567.24 (46.8%) | 1582381.07 (104.66%) | -777962.22 (-51.45%) |
| High SDI | -227152.38 | 205538.81 (-90.48%) | 340260.67 (-149.79%) | -772951.86 (340.28%) |
| Central Europe, Eastern Europe, and Central Asia | -219161.74 | 183503.45 (-83.73%) | 167167.87 (-76.28%) | -569833.06 (260.01%) |
| High-income | -471577.28 | 223460.05 (-47.39%) | 265963.55 (-56.4%) | -961000.88 (203.78%) |
| Latin America and Caribbean | 106627.94 | 66801.59 (62.65%) | 263732.58 (247.34%) | -223906.24 (-209.99%) |
| North Africa and Middle East | 680330.97 | 18387.37 (2.7%) | 677680.14 (99.61%) | -15736.54 (-2.31%) |
| South Asia | 2510394.5 | 220702.28 (8.79%) | 1233019.93 (49.12%) | 1056672.28 (42.09%) |
| Southeast Asia, East Asia, and Oceania | 6615765.12 | 1670656.12 (25.25%) | 3245239.69 (49.05%) | 1699869.31 (25.69%) |
| Sub-Saharan Africa | 460411.35 | -28963.32 (-6.29%) | 311956.08 (67.76%) | 177418.6 (38.53%) |
| Stroke attributed to ambient particulate matter pollution among females | | | | |
| Global | 6196747.04 | 1149689.8 (18.55%) | 4812623.61 (77.66%) | 234433.63 (3.78%) |
| Low SDI | 442426.53 | -2409.74 (-0.54%) | 234795.96 (53.07%) | 210040.31 (47.47%) |
| Low-middle SDI | 1921844.2 | 175885.61 (9.15%) | 836053.46 (43.5%) | 909905.13 (47.35%) |
| Middle SDI | 3551576.18 | 733318.32 (20.65%) | 2133680.56 (60.08%) | 684577.3 (19.28%) |
| High-middle SDI | 653338.97 | 463150.2 (70.89%) | 1221040.05 (186.89%) | -1030851.28 (-157.78%) |
| High SDI | -374790.07 | 146213.95 (-39.01%) | 269685.22 (-71.96%) | -790689.24 (210.97%) |
| Central Europe, Eastern Europe, and Central Asia | -484696.93 | 141112.53 (-29.11%) | 151745.11 (-31.31%) | -777554.57 (160.42%) |
| High-income | -537954.45 | 171144.72 (-31.81%) | 241276.64 (-44.85%) | -950375.81 (176.66%) |
| Latin America and Caribbean | 118449.16 | 64019 (54.05%) | 234827.75 (198.25%) | -180397.59 (-152.3%) |
| North Africa and Middle East | 639747.32 | 30821.15 (4.82%) | 575326.12 (89.93%) | 33600.05 (5.25%) |
| South Asia | 1986601.25 | 215970.79 (10.87%) | 914007.5 (46.01%) | 856622.96 (43.12%) |
| Southeast Asia, East Asia, and Oceania | 4092797.77 | 1071522.81 (26.18%) | 2178440 (53.23%) | 842834.96 (20.59%) |
| Sub-Saharan Africa | 381802.92 | -13161.19 (-3.45%) | 250715.08 (65.67%) | 144249.03 (37.78%) |
| Chronic obstructive pulmonary disease attributed to ambient particulate matter pollution among both genders | | | | |
| Global | 7431272.82 | 2216333.65 (29.82%) | 6402749.66 (86.16%) | -1187810.49 (-15.98%) |
| Low SDI | 954208.68 | -13350.56 (-1.4%) | 587927.51 (61.61%) | 379631.73 (39.78%) |
| Low-middle SDI | 3920483.18 | 587567.26 (14.99%) | 2048917.59 (52.26%) | 1283998.33 (32.75%) |
| Middle SDI | 2446744.24 | 1313672.55 (53.69%) | 2812427.58 (114.95%) | -1679355.89 (-68.64%) |
| High-middle SDI | 144149.05 | 618085.97 (428.78%) | 1141717.19 (792.04%) | -1615654.1 (-1120.82%) |
| High SDI | -35865.71 | 180692.75 (-503.8%) | 233983.5 (-652.39%) | -450541.96 (1256.19%) |
| Central Europe, Eastern Europe, and Central Asia | -212574.07 | 65527.88 (-30.83%) | 58161.08 (-27.36%) | -336263.03 (158.19%) |
| High-income | -94450.51 | 211563.53 (-223.99%) | 212239.59 (-224.71%) | -518253.63 (548.7%) |
| Latin America and Caribbean | 154139.11 | 72221.44 (46.85%) | 167526.1 (108.69%) | -85608.44 (-55.54%) |
| North Africa and Middle East | 408686.2 | 17720.57 (4.34%) | 349639.64 (85.55%) | 41325.98 (10.11%) |
| South Asia | 5399140.84 | 993543.14 (18.4%) | 2855718.19 (52.89%) | 1549879.51 (28.71%) |
| Southeast Asia, East Asia, and Oceania | 1491209.04 | 2045005.3 (137.14%) | 3102077.52 (208.02%) | -3655873.79 (-245.16%) |
| Sub-Saharan Africa | 285122.21 | -19044.06 (-6.68%) | 217010.54 (76.11%) | 87155.73 (30.57%) |
| Chronic obstructive pulmonary disease attributed to ambient particulate matter pollution among males | | | | |
| Global | 4147677.72 | 1588545.24 (38.3%) | 3795100.97 (91.5%) | -1235968.48 (-29.8%) |
| Low SDI | 537900.42 | -21293.7 (-3.96%) | 359917.5 (66.91%) | 199276.62 (37.05%) |
| Low-middle SDI | 2240137.34 | 312465.9 (13.95%) | 1233300.74 (55.05%) | 694370.7 (31%) |
| Middle SDI | 1388081.42 | 814236.91 (58.66%) | 1590257.13 (114.57%) | -1016412.62 (-73.22%) |
| High-middle SDI | 13991.46 | 441160.81 (3153.07%) | 683739.14 (4886.83%) | -1110908.49 (-7939.9%) |
| High SDI | -33369.18 | 139573.16 (-418.27%) | 149114.84 (-446.86%) | -322057.18 (965.13%) |
| Central Europe, Eastern Europe, and Central Asia | -144768.68 | 58226.56 (-40.22%) | 40407.79 (-27.91%) | -243403.03 (168.13%) |
| High-income | -83507.22 | 162768.58 (-194.92%) | 132993.42 (-159.26%) | -379269.22 (454.18%) |
| Latin America and Caribbean | 74614.35 | 37803.85 (50.67%) | 93378.5 (125.15%) | -56568 (-75.81%) |
| North Africa and Middle East | 254748.64 | 8969.59 (3.52%) | 223205.41 (87.62%) | 22573.64 (8.86%) |
| South Asia | 3036871.81 | 531019.94 (17.49%) | 1704387.44 (56.12%) | 801464.43 (26.39%) |
| Southeast Asia, East Asia, and Oceania | 845547.48 | 1234158.08 (145.96%) | 1728245.05 (204.39%) | -2116855.64 (-250.35%) |
| Sub-Saharan Africa | 164171.34 | -14710.6 (-8.96%) | 128784.19 (78.44%) | 50097.75 (30.52%) |
| Chronic obstructive pulmonary disease attributed to ambient particulate matter pollution among females | | | | |
| Global | 3283595.1 | 772109.47 (23.51%) | 2601330.85 (79.22%) | -89845.22 (-2.74%) |
| Low SDI | 416308.26 | 3363.34 (0.81%) | 226012.42 (54.29%) | 186932.5 (44.9%) |
| Low-middle SDI | 1680345.83 | 255743.24 (15.22%) | 793305.41 (47.21%) | 631297.17 (37.57%) |
| Middle SDI | 1058662.82 | 519082.62 (49.03%) | 1203661.43 (113.7%) | -664081.23 (-62.73%) |
| High-middle SDI | 130157.59 | 235046.17 (180.59%) | 467653.21 (359.3%) | -572541.79 (-439.88%) |
| High SDI | -2496.53 | 64243.73 (-2573.32%) | 90117.31 (-3609.7%) | -156857.57 (6283.02%) |
| Central Europe, Eastern Europe, and Central Asia | -67805.39 | 22244.14 (-32.81%) | 19642.01 (-28.97%) | -109691.54 (161.77%) |
| High-income | -10943.29 | 75142.42 (-686.65%) | 83271.09 (-760.93%) | -169356.8 (1547.59%) |
| Latin America and Caribbean | 79524.76 | 33212.82 (41.76%) | 73277.58 (92.14%) | -26965.64 (-33.91%) |
| North Africa and Middle East | 153937.56 | 7875.56 (5.12%) | 128255.33 (83.32%) | 17806.67 (11.57%) |
| South Asia | 2362269.03 | 434250.93 (18.38%) | 1115274.88 (47.21%) | 812743.22 (34.41%) |
| Southeast Asia, East Asia, and Oceania | 645661.56 | 846248.11 (131.07%) | 1361748.85 (210.91%) | -1562335.4 (-241.97%) |
| Sub-Saharan Africa | 120950.87 | -5489.89 (-4.54%) | 86616.04 (71.61%) | 39824.72 (32.93%) |
| Diabetes mellitus attributed to ambient particulate matter pollution among both genders | | | | |
| Global | 6703548.73 | 769240.34 (11.48%) | 2810344.86 (41.92%) | 3123963.52 (46.6%) |
| Low SDI | 352635.79 | -6018.15 (-1.71%) | 154392.87 (43.78%) | 204261.07 (57.92%) |
| Low-middle SDI | 1558921.94 | 105015.72 (6.74%) | 520172.51 (33.37%) | 933733.71 (59.9%) |
| Middle SDI | 3153128.09 | 438421.16 (13.9%) | 1199544.94 (38.04%) | 1515162 (48.05%) |
| High-middle SDI | 1299078.6 | 225942.29 (17.39%) | 571442.74 (43.99%) | 501693.57 (38.62%) |
| High SDI | 335805.58 | 162780.57 (48.47%) | 273698.78 (81.51%) | -100673.77 (-29.98%) |
| Central Europe, Eastern Europe, and Central Asia | 241737.36 | 45005.52 (18.62%) | 51454.32 (21.29%) | 145277.53 (60.1%) |
| High-income | 239125.1 | 197617.13 (82.64%) | 257023.66 (107.49%) | -215515.68 (-90.13%) |
| Latin America and Caribbean | 686119.32 | 133679 (19.48%) | 390799.27 (56.96%) | 161641.05 (23.56%) |
| North Africa and Middle East | 782512.18 | 20399.14 (2.61%) | 450948.18 (57.63%) | 311164.86 (39.76%) |
| South Asia | 2058079.9 | 163488.56 (7.94%) | 713500.05 (34.67%) | 1181091.28 (57.39%) |
| Southeast Asia, East Asia, and Oceania | 2329281.46 | 362821.66 (15.58%) | 778337.18 (33.42%) | 1188122.61 (51.01%) |
| Sub-Saharan Africa | 366693.41 | -17457.75 (-4.76%) | 186941.15 (50.98%) | 197210 (53.78%) |
| Diabetes mellitus attributed to ambient particulate matter pollution among males | | | | |
| Global | 3648256.84 | 439618.99 (12.05%) | 1456086.47 (39.91%) | 1752551.38 (48.04%) |
| Low SDI | 203558.44 | -6258 (-3.07%) | 92593.52 (45.49%) | 117222.93 (57.59%) |
| Low-middle SDI | 867267.98 | 50391.79 (5.81%) | 285548.47 (32.93%) | 531327.72 (61.26%) |
| Middle SDI | 1646489.88 | 228039.72 (13.85%) | 606576.55 (36.84%) | 811873.6 (49.31%) |
| High-middle SDI | 694878.59 | 126671.1 (18.23%) | 288320.15 (41.49%) | 279887.34 (40.28%) |
| High SDI | 233900.23 | 96152.21 (41.11%) | 151664.06 (64.84%) | -13916.04 (-5.95%) |
| Central Europe, Eastern Europe, and Central Asia | 123334.68 | 26330.2 (21.35%) | 25002.43 (20.27%) | 72002.05 (58.38%) |
| High-income | 175967.93 | 113848.72 (64.7%) | 134343.92 (76.35%) | -72224.71 (-41.04%) |
| Latin America and Caribbean | 360936.9 | 61575.22 (17.06%) | 193919.48 (53.73%) | 105442.2 (29.21%) |
| North Africa and Middle East | 404924.47 | 7534.01 (1.86%) | 231771.55 (57.24%) | 165618.9 (40.9%) |
| South Asia | 1129450.42 | 78100.23 (6.91%) | 390555.89 (34.58%) | 660794.3 (58.51%) |
| Southeast Asia, East Asia, and Oceania | 1255508.48 | 192279.3 (15.31%) | 409066.66 (32.58%) | 654162.53 (52.1%) |
| Sub-Saharan Africa | 198133.96 | -12180.57 (-6.15%) | 100836.66 (50.89%) | 109477.87 (55.25%) |
| Diabetes mellitus attributed to ambient particulate matter pollution among females | | | | |
| Global | 3055291.89 | 335419.79 (10.98%) | 1352273.01 (44.26%) | 1367599.09 (44.76%) |
| Low SDI | 149077.35 | -566.2 (-0.38%) | 61439.86 (41.21%) | 88203.7 (59.17%) |
| Low-middle SDI | 691653.96 | 52663.63 (7.61%) | 232188.18 (33.57%) | 406802.15 (58.82%) |
| Middle SDI | 1506638.21 | 209719.45 (13.92%) | 591196.41 (39.24%) | 705722.35 (46.84%) |
| High-middle SDI | 604200.01 | 102533.39 (16.97%) | 282969.32 (46.83%) | 218697.3 (36.2%) |
| High SDI | 101905.35 | 70493.63 (69.18%) | 122916.4 (120.62%) | -91504.68 (-89.79%) |
| Central Europe, Eastern Europe, and Central Asia | 118402.68 | 20169.16 (17.03%) | 26375.95 (22.28%) | 71857.57 (60.69%) |
| High-income | 63157.17 | 87192.55 (138.06%) | 122893.05 (194.58%) | -146928.43 (-232.64%) |
| Latin America and Caribbean | 325182.42 | 71702.75 (22.05%) | 196644.54 (60.47%) | 56835.13 (17.48%) |
| North Africa and Middle East | 377587.71 | 12964.94 (3.43%) | 218841.78 (57.96%) | 145781 (38.61%) |
| South Asia | 928629.48 | 83009.54 (8.94%) | 319028.09 (34.35%) | 526591.85 (56.71%) |
| Southeast Asia, East Asia, and Oceania | 1073772.97 | 171193.19 (15.94%) | 367677.84 (34.24%) | 534901.95 (49.82%) |
| Sub-Saharan Africa | 168559.45 | -6152.44 (-3.65%) | 85501.52 (50.72%) | 89210.38 (52.93%) |
