# Supplementary material for: The global burden, trends and cross-region inequities of non-communicable diseases attributed to ambient particulate matter pollution
Source: Front Public Health. 2025 Nov 3;13:1682574. doi: 10.3389/fpubh.2025.1682574 (PMC12620374; doi:10.3389/fpubh.2025.1682574)
Supplement: Supplementary file 3 [file Supplementary_file_2.docx]

**Figure Legend**

Figure S1 ASDRs of ambient particulate matter-attributed non-communicable diseases in 2019.

Figure S2 AAPCs on ASDRs of ambient particulate matter-attributed non-communicable diseases from 1990-2019.

Figure S3 Trends of ASDR of non-communicable diseases attributed to ambient particulate matter from 1990-2019.

Figure S4 Trends of age-specific DALY rate of non-communicable diseases attributed to ambient particulate matter from 1990-2019.

Figure S5 The changes in the DALYs of ambient particulate matter-attributed non-communicable diseases driven by aging, population growth, and epidemiological change from 1990-2019.

Figure S6 The correlation between SDI and ASDR in 2019 for ambient particulate matter-attributed non-communicable diseases.

Figure S7 The correlation between SDI and ASDR from 1990-2019 for ambient particulate matter-attributed non-communicable diseases.

Figure S8 Health inequality curves for the DALYs of ambient particulate matter-attributed non-communicable diseases from 1990-2019 across the world.

Figure S9 Health concentration curves for the DALYs of ambient particulate matter-attributed non-communicable diseases from 1990-2019 across the world.

Figure S10 Age-specific death numbers and rates of ambient particulate matter-attributed non-communicable diseases in 2019.

Figure S11 Age-specific DALY numbers and rates of ambient particulate matter-attributed non-communicable diseases in 2019.


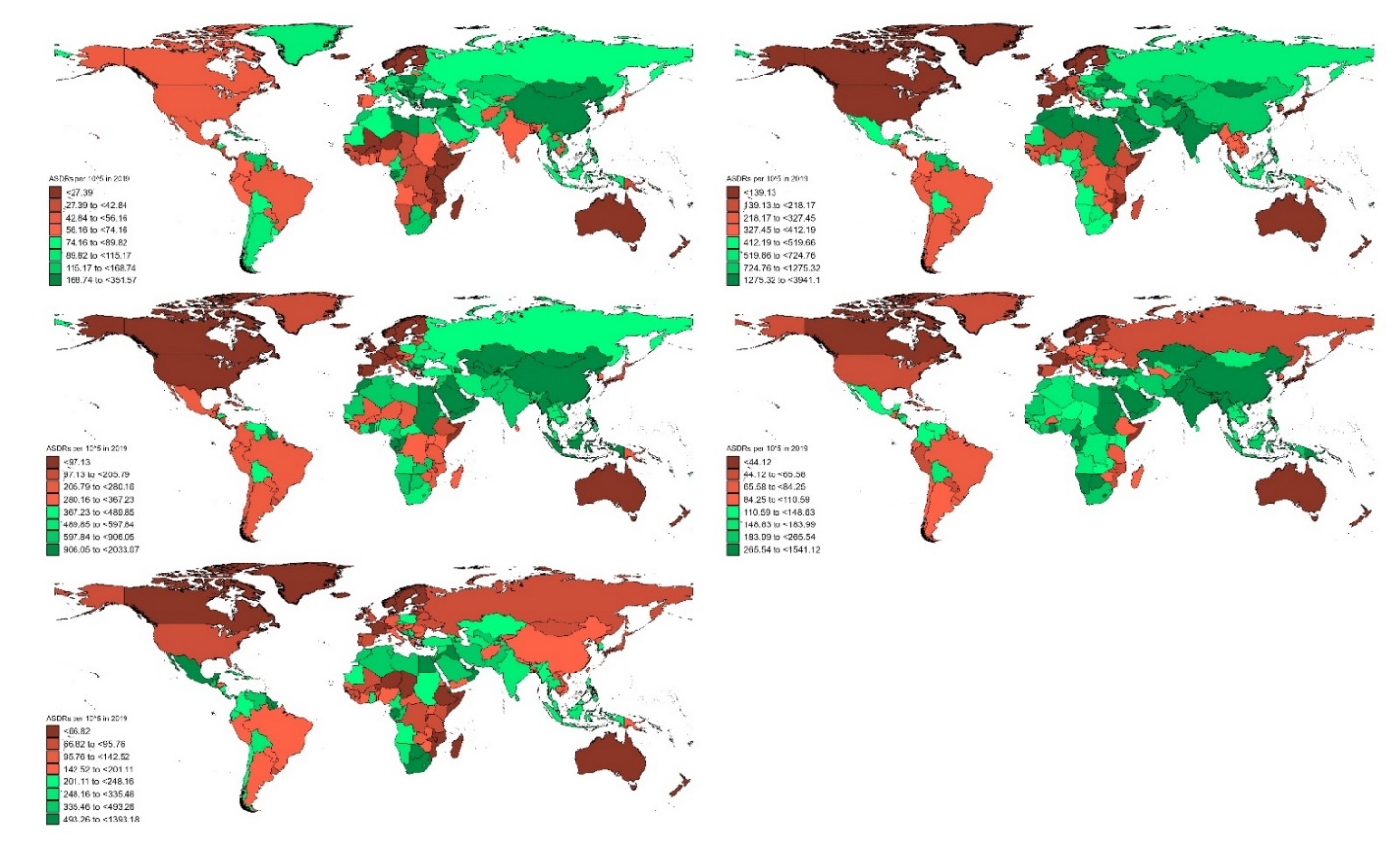


Figure S1 ASDRs of ambient particulate matter-attributed non-communicable diseases in 2019.

Figure S2 AAPCs on ASDRs of ambient particulate matter-attributed non-communicable diseases from 1990-2019.


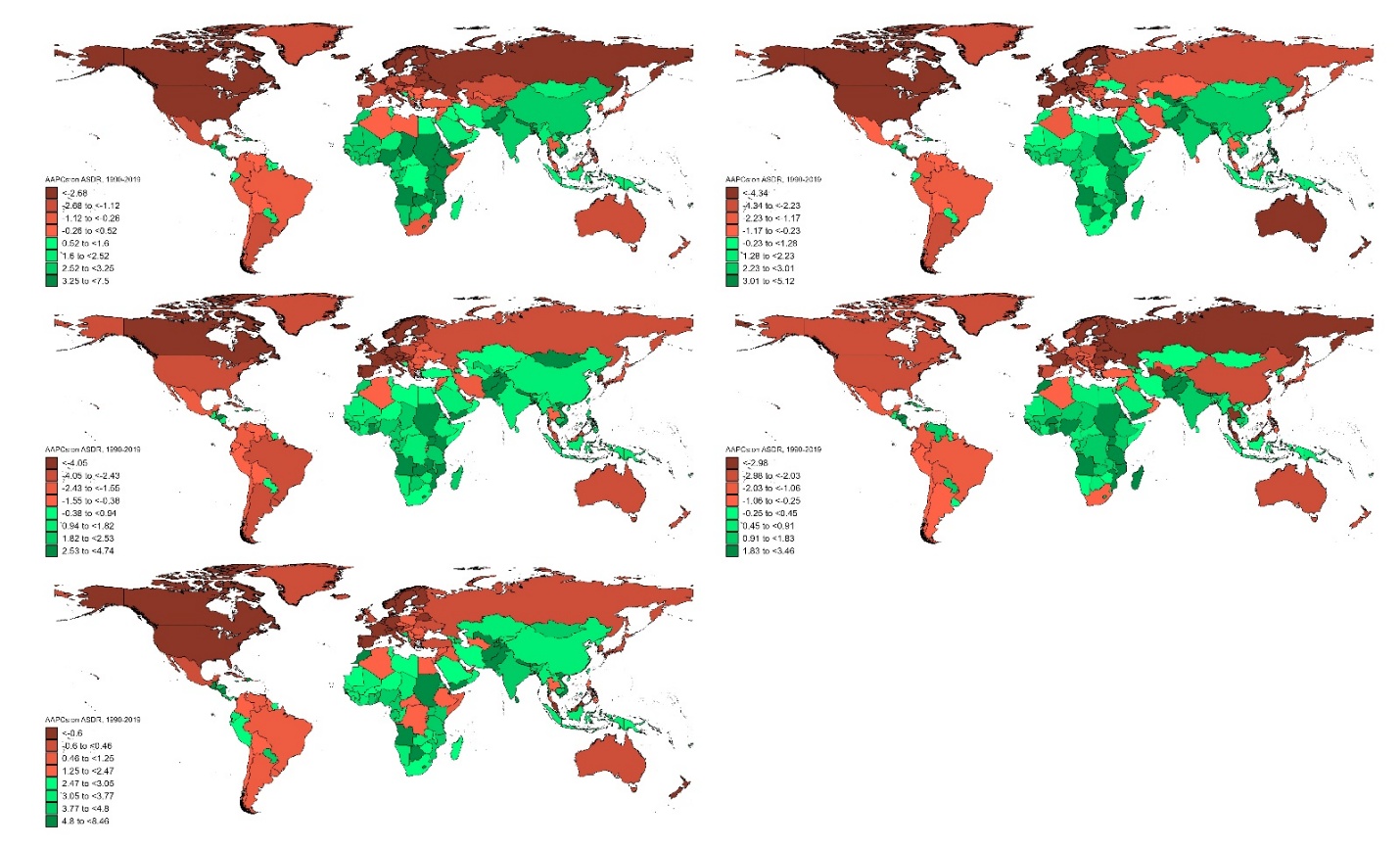


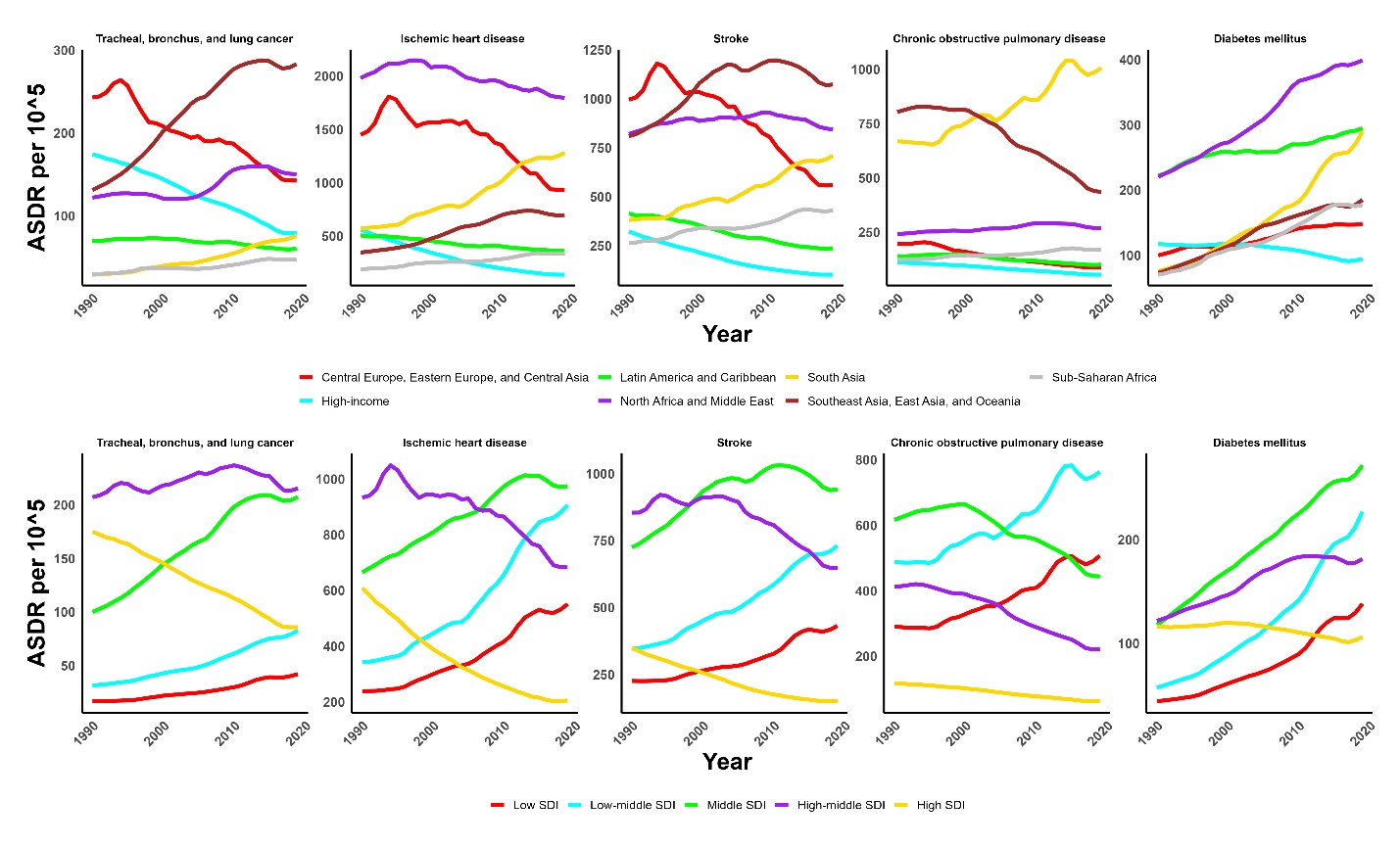


Figure S3 Trends of ASDR of non-communicable diseases attributed to ambient particulate matter from 1990-2019.


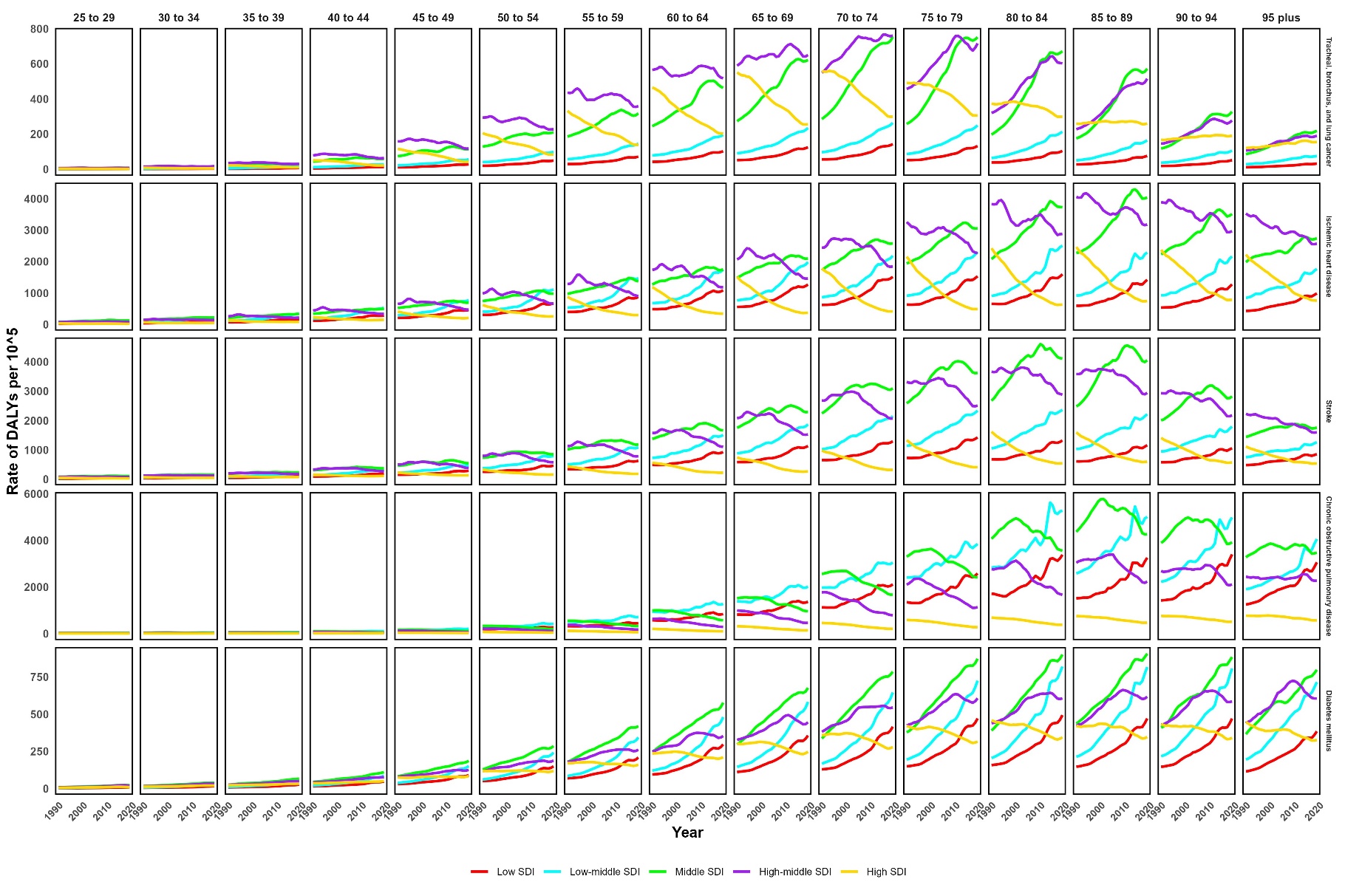


Figure S4 Trends of age-specific DALY rate of non-communicable diseases attributed to ambient particulate matter from 1990-2019.

Figure S5 The changes in the DALYs of ambient particulate matter-attributed non-communicable diseases driven by aging, population growth, and epidemiological change from 1990-2019.


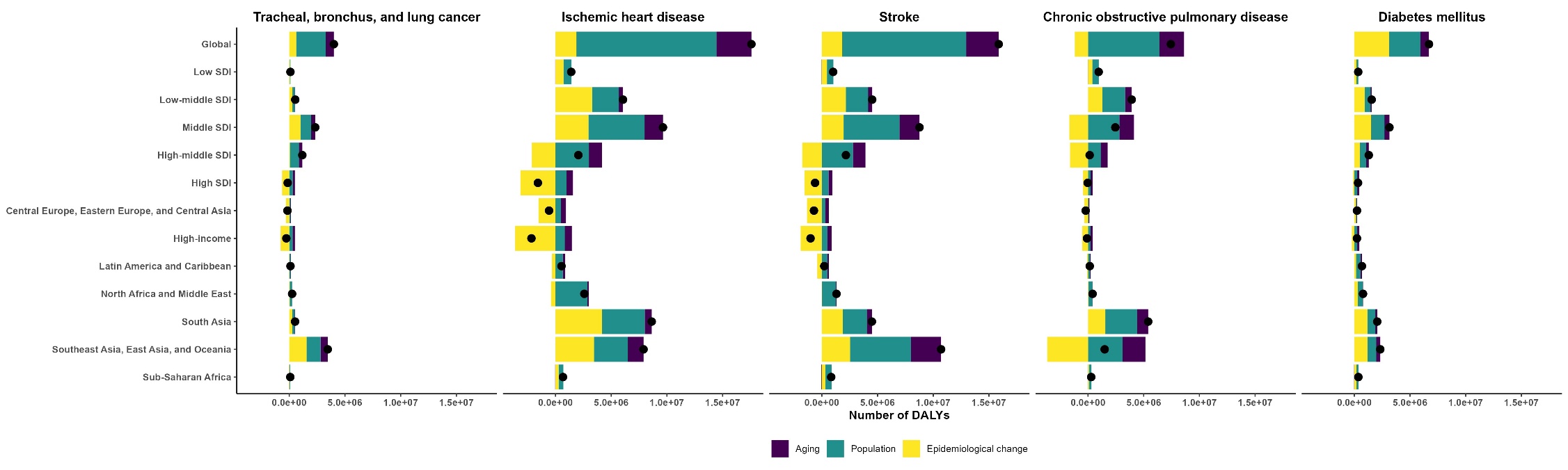


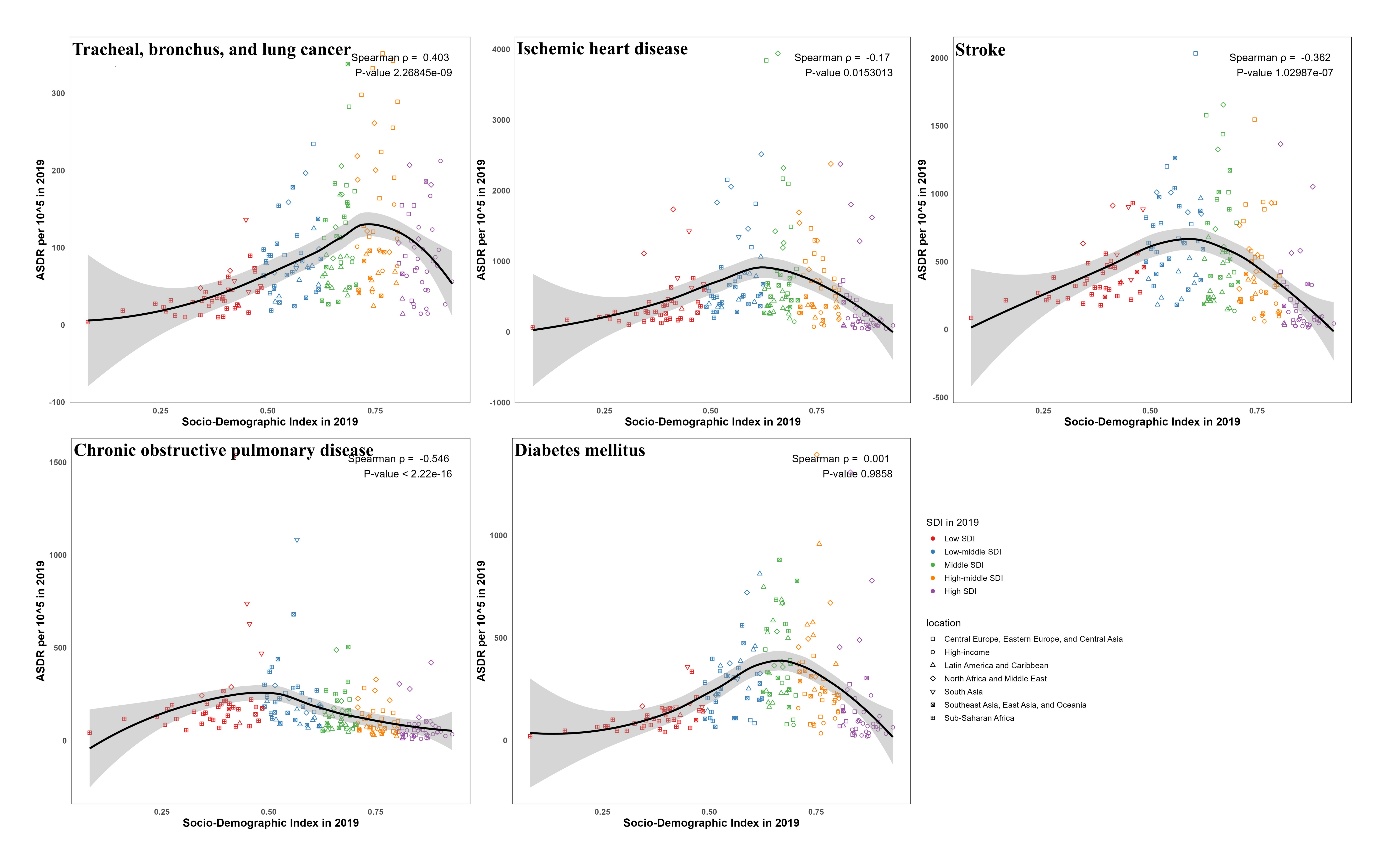


Figure S6 The correlation between SDI and ASDR in 2019 for ambient particulate matter-attributed non-communicable diseases.


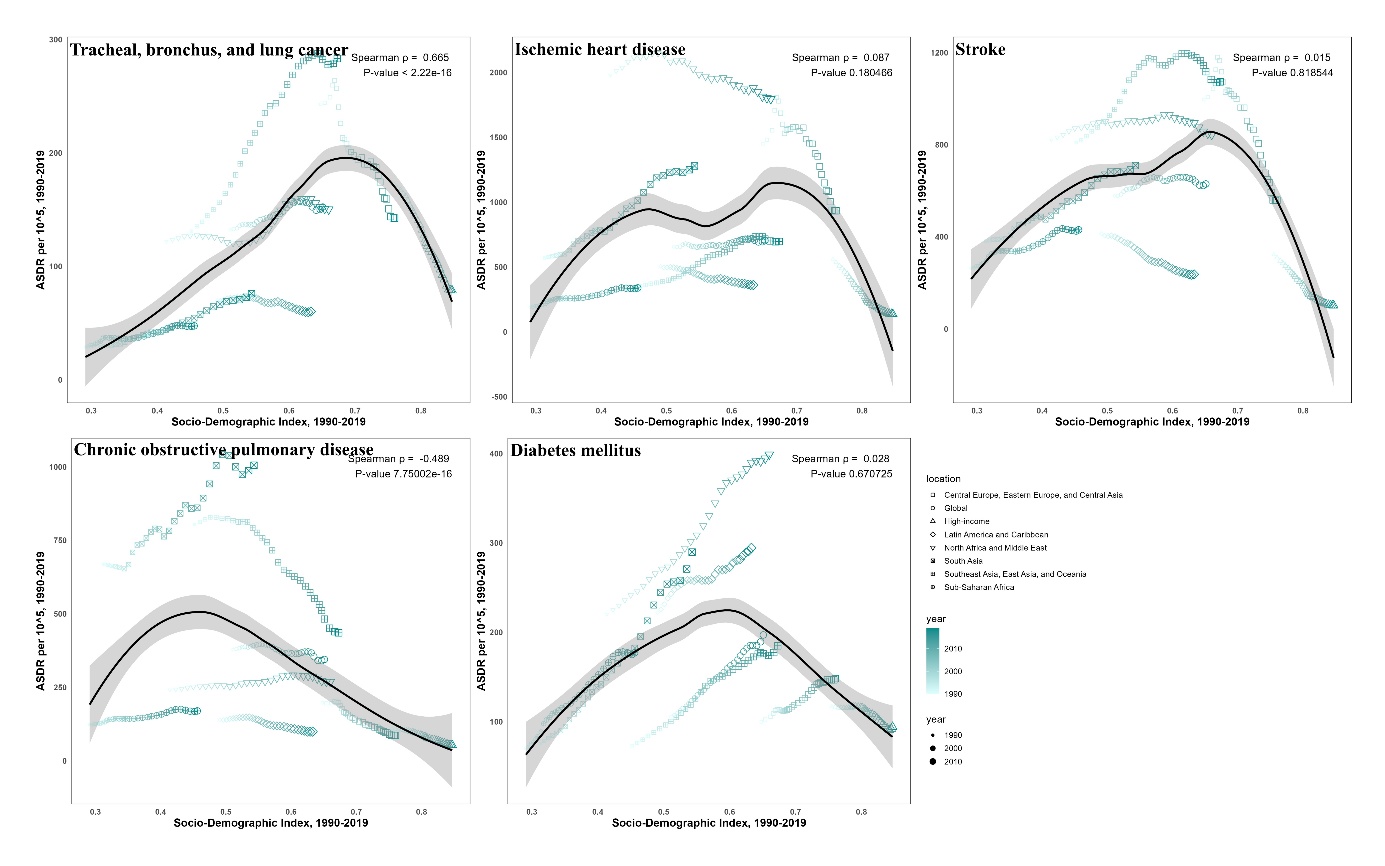


Figure S7 The correlation between SDI and ASDR from 1990-2019 for ambient particulate matter-attributed non-communicable diseases.


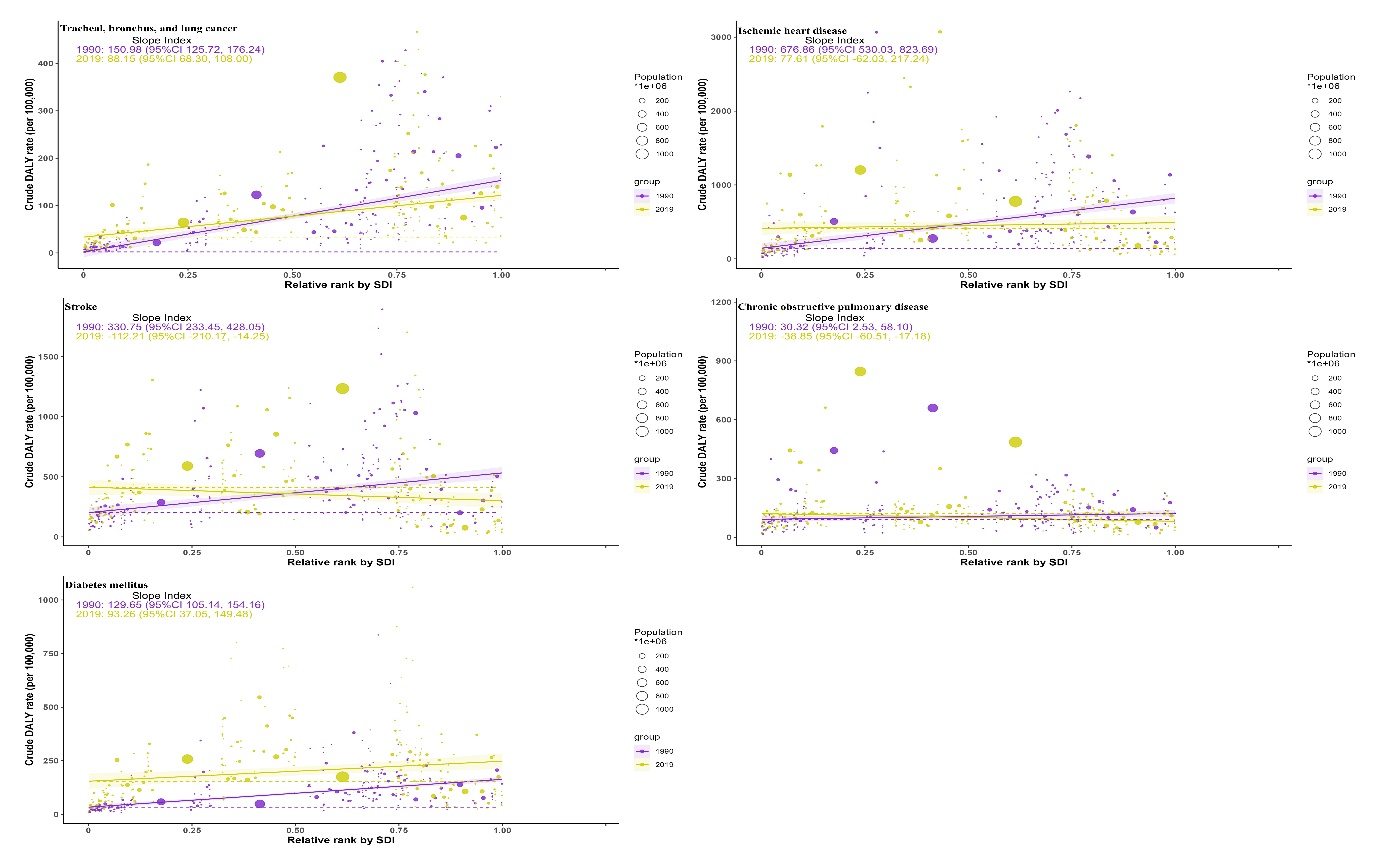


Figure S8 Health inequality curves for the DALYs of ambient particulate matter-attributed non-communicable diseases from 1990-2019 across the world.


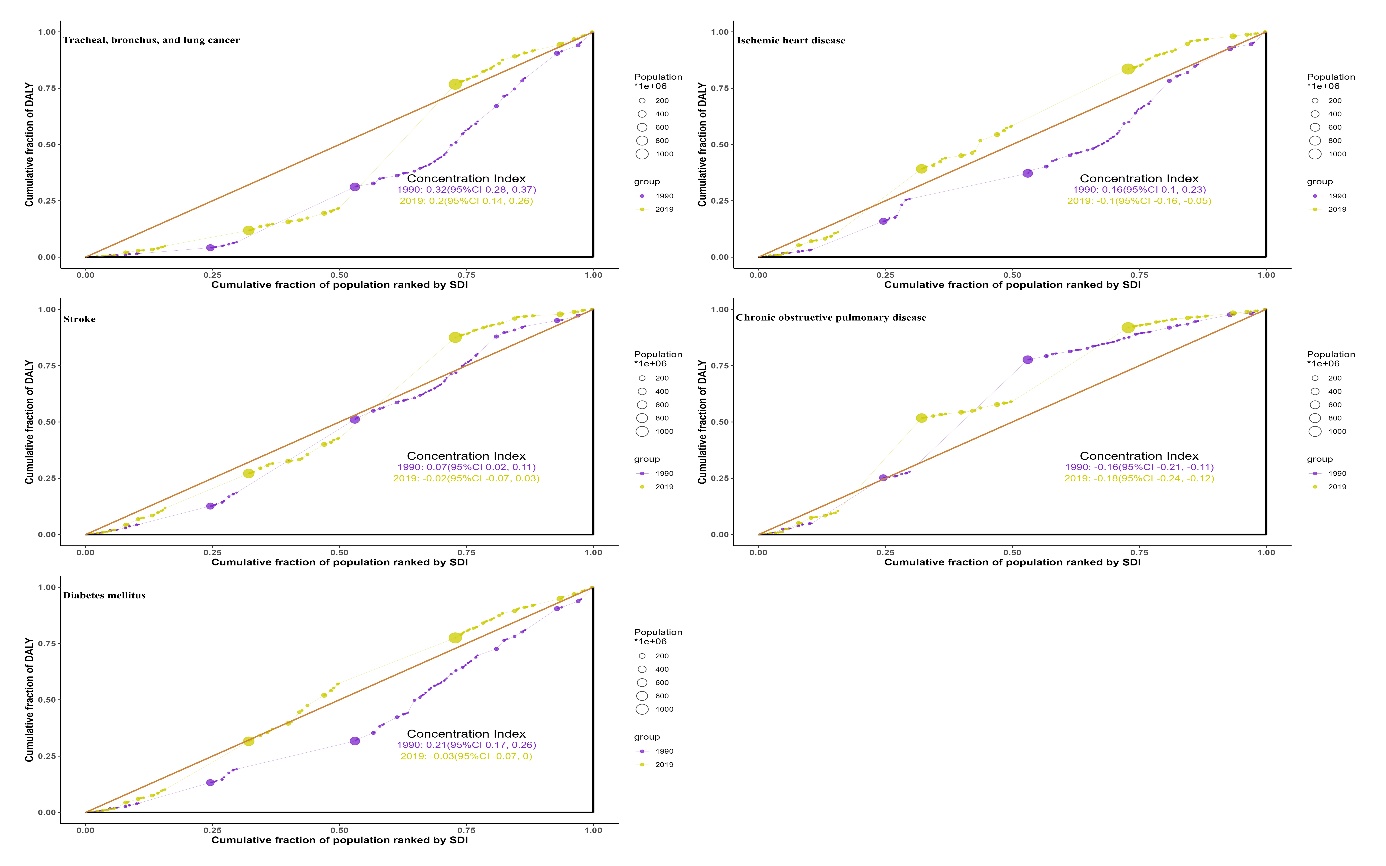


Figure S9 Health concentration curves for the DALYs of ambient particulate matter-attributed non-communicable diseases from 1990-2019 across the world.


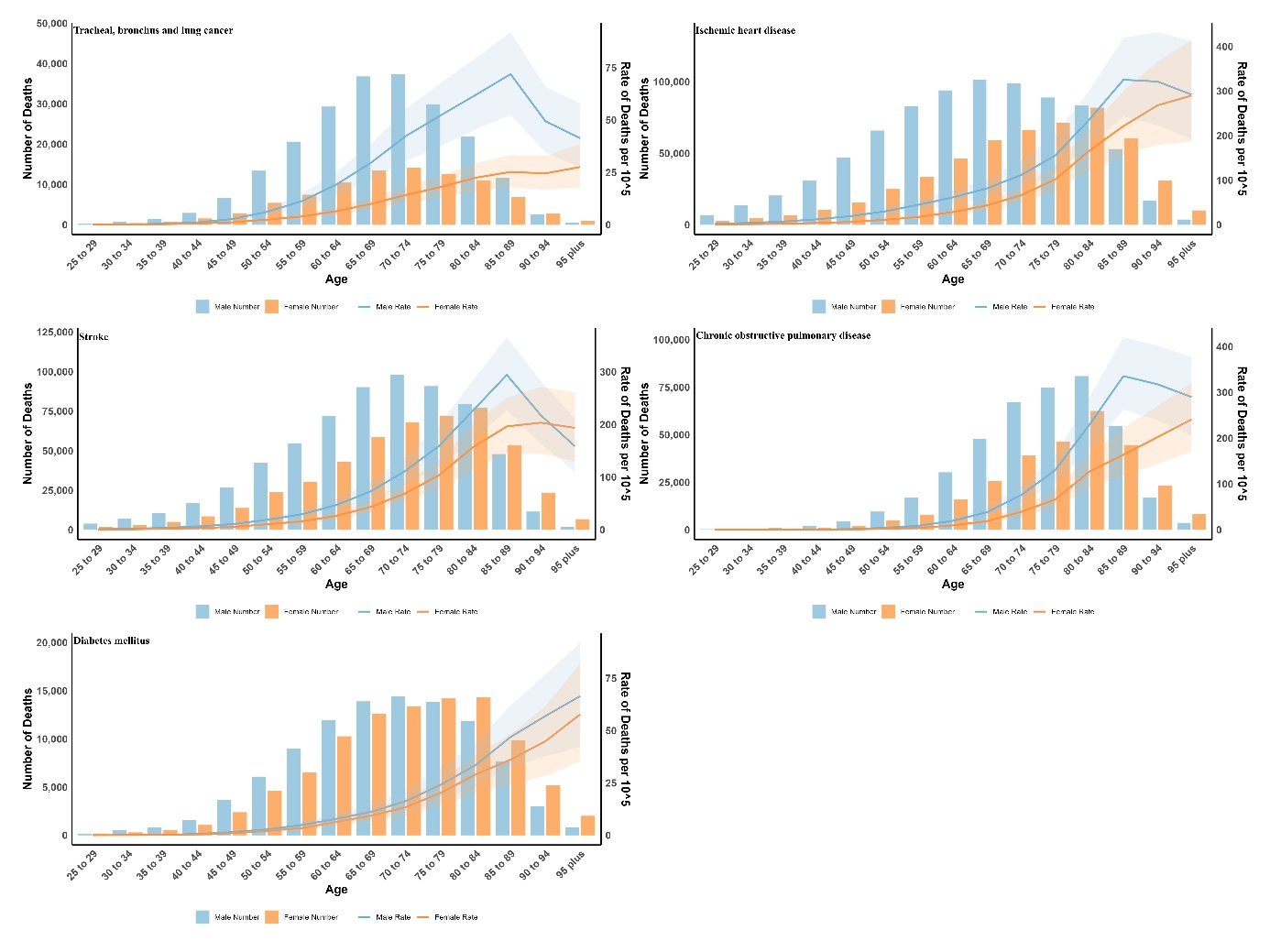


Figure S10 Age-specific death numbers and rates of ambient particulate matter-attributed non-communicable diseases in 2019.


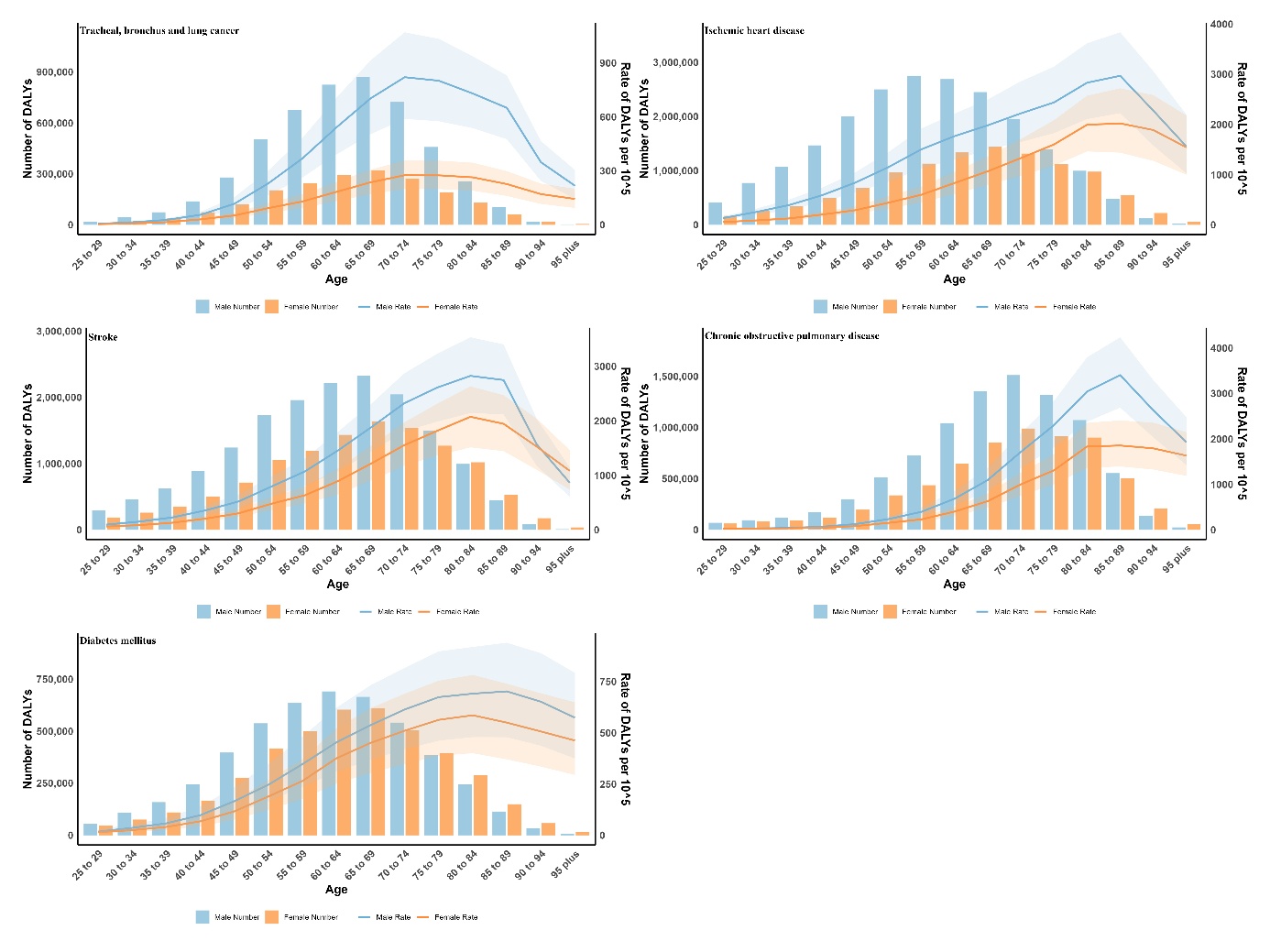


Figure S11 Age-specific DALY numbers and rates of ambient particulate matter-attributed non-communicable diseases in 2019.
