# Supplementary material for: The global burden, trends and cross-region inequities of non-communicable diseases attributed to ambient particulate matter pollution
Source: Front Public Health. 2025 Nov 3;13:1682574. doi: 10.3389/fpubh.2025.1682574 (PMC12620374; doi:10.3389/fpubh.2025.1682574)
Supplement: Supplementary file 4 [file Table_1.DOCX]

| **Chronic non-communicable diseases attributed to ambient particulate matter pollution and their corresponding ICD-10 codes in the GBD 2019** | |
| --- | --- |
| **Name of disease** | **Corresponding codes of ICD-10** |
| Tracheal, bronchus, and lung cancer | C33, C34-C34.92, Z12.2, Z80.1-Z80.2, and Z85.1-Z85.20 |
| Ischemic heart disease | I20–I21.6, I21.9–I25.9, and Z82.4–Z82.49 |
| Stroke | I60–I63.9, I65–I67.9, I69–I69.8 |
| Chronic obstructive pulmonary disease | J41-J44 |
| Diabetes mellitus | ICD-10 E10–E14 |
| **ICD-10: International Classification of Diseases, 10th Edition** | |
